# Organocatalytic stereoselective C–H diazenylation of active methylenes with arenediazonium salts

**DOI:** 10.1039/d6ra07931h

**Published:** 2026-09-08

**Authors:** Aqsa, Alaud Din, Mehtab Ahmad, Hammad Khan, Muhammad Israr, Syed Osama, Qudrat Ullah, Sumbal Saba, Zi-Sheng Chen, Syed Adnan Ali Shah, Shaukat Ali

**Affiliations:** a Organic Synthesis and Catalysis Research Laboratory, Institute of Chemical Sciences, University of Peshawar Peshawar 25120 Khyber Pakhtunkhwa Pakistan drshaukatali@uop.edu.pk; b Center for Integrative Petroleum Research (CIPR), College of Petroleum Engineering and Geosciences, King Fahd University of Petroleum & Minerals Dhahran 31261 Saudi Arabia; c Laboratório de Síntese Sustentável e Organocalcogênio (LABSO), Instituto de Química (IQ), Universidade Federal de Goiás—UFG Goiânia 74690-900 GO Brazil; d Shaanxi Key Laboratory of Natural Products & Chemical Biology, College of Chemistry and Pharmacy, Northwest A&F University Yangling 712100 Shaanxi P. R. China chenzsh@nwafu.edu.cn; e Faculty of Pharmacy, Universiti Teknologi MARA (UiTM) Cawangan Selangor Kampus Puncak Alam Bandar Puncak Alam 42300 Selangor Darul Ehsan Malaysia syedadnan@uitm.edu.my; f Atta-ur-Rahman Institute for Natural Product Discovery (AuRIns), Universiti Teknologi MARA (UiTM) Cawangan Selangor Kampus Puncak Alam Bandar Puncak Alam 42300 Selangor Darul Ehsan Malaysia

## Abstract

Herein, we report the unveiling of the first organocatalytic sp^3^ C–H diazenylation of active methylenes with arenediazonium salts under ambient conditions, affording diverse hydrazones. The reaction features mild and additive-free conditions, excellent regio- and chemoselectivity, the use of readily available substrates with a broad scope, and excellent yields up to 99%. In addition, with ethyl 4,4,4-trifluoroacetoacetate as active methylene, exclusively *Z*-selective hydrazones were isolated. Moreover, the green chemistry metrics calculated for the reaction, such as the atom economy, *E*-factor, TON, and TOF, are promising. In contrast to the classical Japp–Klingemann reaction, no removal of any molecular fragment was observed. Application to the synthesis of valuable targets was also carried out. Mechanistic experiments revealed a polar pathway for the reaction. Furthermore, the role of intramolecular H-bonding was found to be crucial not only in stabilizing the reaction product but also in determining the observed *Z*-stereoselectivity. Such a substituent-switched H-bonding-driven induction of stereoselectivity, which affords stereoselective trifluoroacetyl-substituted *Z*-hydrazones, is remarkable and unprecedented in aminocatalysis.

## Abstract 2

The simple yet effective: the aminocatalytic C–H diazenylation of active methylenes affords diverse hydrazones under ambient conditions. Remarkably, with ethyl 4,4,4-trifluoroacetoacetate, exclusively stereoselective *Z*-hydrazones were isolated.

## Graphical abstract

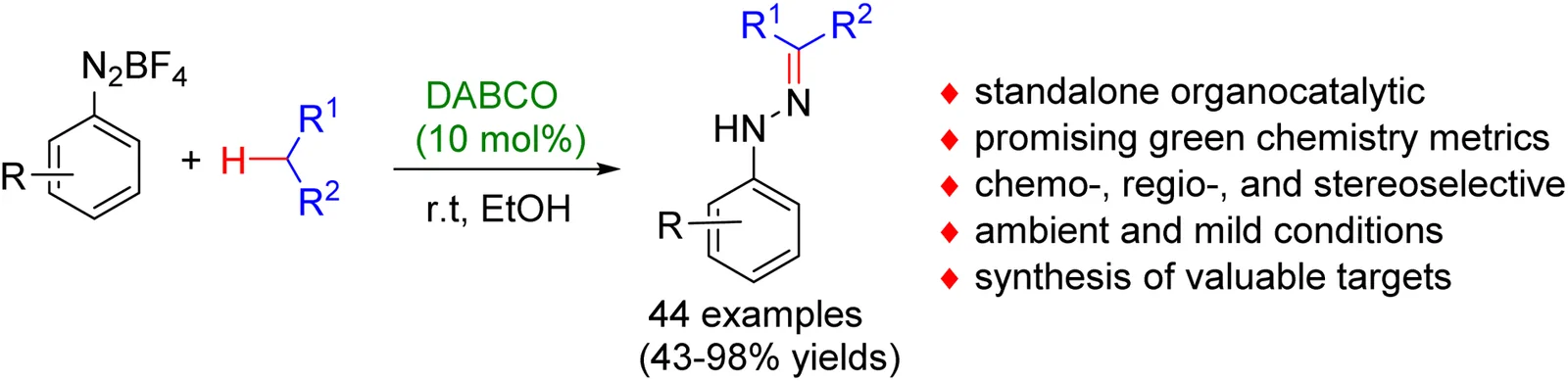

## Introduction

The efficient formation of C–N bonds supports advances in the synthesis of complex target molecules, particularly in pharmaceutical and material applications, underscoring their pivotal role in organic synthesis.^1^ The formation of these bonds *via* direct C(sp^3^)–H bond functionalization offers a highly attractive approach that can streamline synthetic pathways by eliminating the need for pre-functionalized substrates and rendering otherwise difficult disconnections possible.^2^ The selective activation of inert C(sp^3^)–H bonds necessitates the development of effective catalysts and novel strategies to achieve high chemo-, regio-, and stereoselectivity.^3^ The use of organocatalysts could help address these issues due to their low cost, high selectivity, insensitivity to air and moisture, low toxicity, and ease of preparation and recovery.^4^ However, formidable challenges exist in organocatalyzed reactions to achieve higher turnover numbers (TONs) and turnover frequencies (TOFs) comparable to transition-metal catalysts.^5^

In this context, active methylene compounds are crucial substrates for key transformations, such as Knoevenagel condensations^6^ and Michael additions.^7^ Moreover, the installation of reactive moieties on molecules allows further structural modifications as well as enhances their binding affinities, pharmacokinetics, and ligation properties. Their versatility in forming C–C and C–N bonds makes them particularly valuable in drug discovery efforts targeting complex biological systems.^8^ However, the regioselective deprotonation of the C(sp^3^)–H bond of active methylenes is an important step, often promoted by stoichiometric (or higher) amounts of metal alkoxides, hydroxides, and acetates. We argued that this deprotonation event could be carried out under mild conditions using a catalytic amine, yielding a carbanionic species which is then quenched by the electrophilic diazonium ion to give a diazo intermediate. A hydrogen-atom transfer from carbon to nitrogen (1,3-HAT), could deliver hydrazones. The conjugate acid of the amine produced can regenerate the aminocatalyst by deprotonation, thereby closing the catalytic cycle (Fig. 1e, left). However, a significant challenge was the development of a previously unexplored catalytic system to regenerate the active amine for catalytic turnovers.

**Fig. 1:**
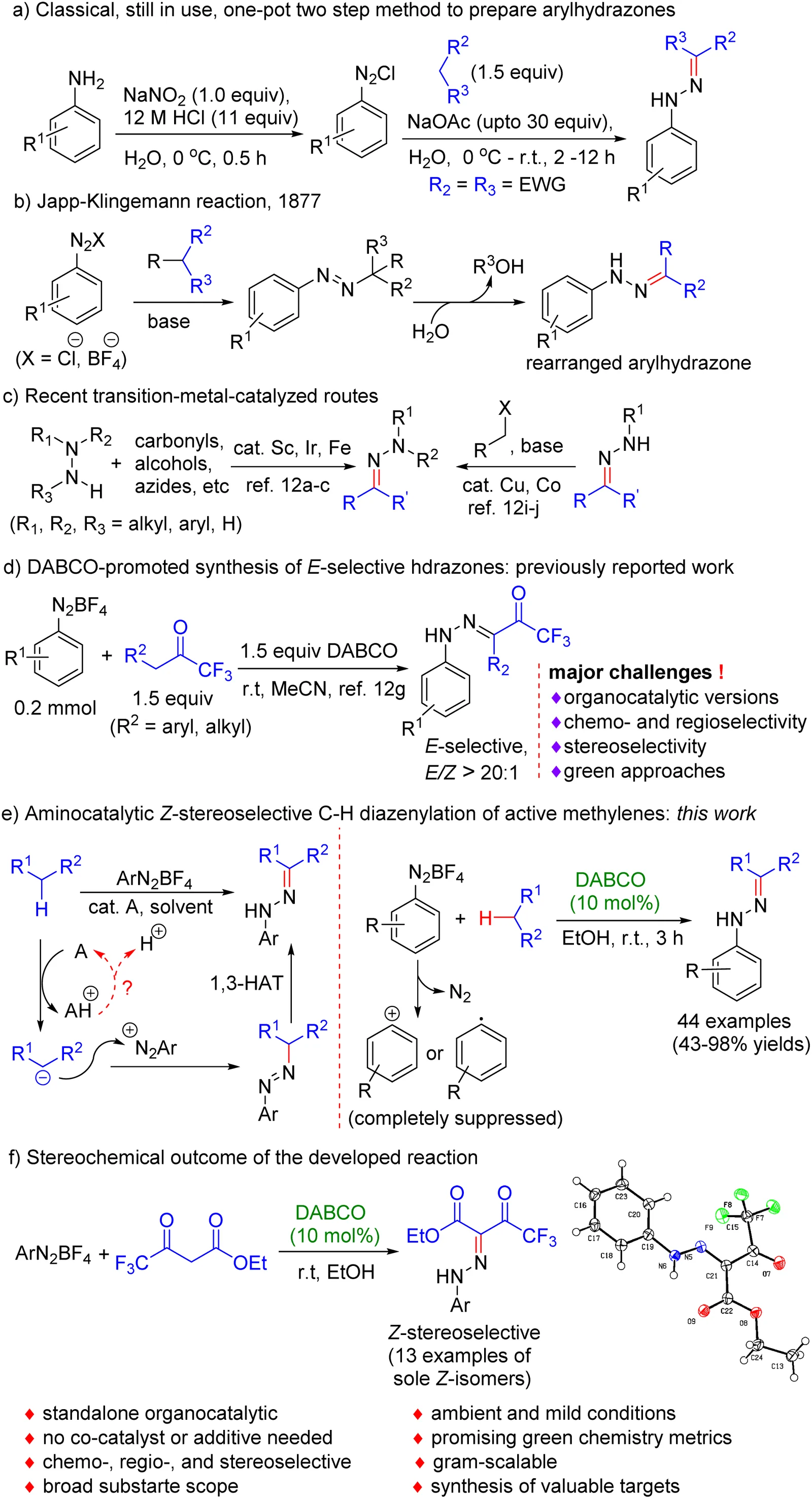
(a–d) Are previous methods to synthesize hydrazones, and the main challenges. (e) Our idea of an organocatalytic approach to hydrazones (left) and our developed method (right), A is aminocatalyst. (f) Substituent-switched stereoselective access to *Z*-hydrazones and salient features of our reaction.

Arylhydrazones are valuable building blocks in organic synthesis.^9^ For instance, they are used as substrates in Fischer indole synthesis and the enantiopure synthesis of amino acids. In addition, they are used in a variety of other fields such as material science^10^ and medicinal chemistry.^11^ Arylhydrazones are generally prepared (Fig. 1a) by a one-pot, two step method from anilines and active methylenes. The classical Japp–Klingemann reaction is also employed to prepare hydrazones from arenediazonium salts and active methylenes *via* expulsion of one electron-withdrawing moiety (Fig. 1b). Besides this, the most common strategies include the coupling of substituted hydrazines (Fig. 1c) with carbonyls,^12*a*^ alcohols,^12*b*^ azides,^12*c*^ Michael acceptors,^12*d*^ or active methylenes.^12*e*^ A gold-catalyzed hydrohydrazidation of alkynes with various hydrazides to afford *N*-acylhydrazones was developed by Kukushkin.^12*f*^ Huanfeng Jiang prepared *E*-selective trifluoroacetylated hydrazones *via* base-mediated amination with arenediazonium salts^12*g*^ (Fig. 1d). Later, Yavari prepared hydrazones by an electrooxidation of benzylic C(sp^3^)–H bonds under metal- and oxidant-free conditions in aqueous media with NaCl as a cheap electrolyte.^12*h*^ In addition, copper- and cobalt-catalyzed *N*-alkylation of NH-hydrazones (Fig. 1c, right) has also been explored.^12*i*,*j*^ Ranging from classical to recent methods, each allows access to useful hydrazone libraries. However, they suffer from drawbacks, including the use of excess amounts of strong acids and bases, the need for transition-metal catalysts, harsh reaction conditions, and the use of carcinogenic hydrazines as a diazo source, among other major challenges (Fig. 1a–d). To this end, an organocatalytic approach that delivers stereoselective *Z*-hydrazones remains elusive. It has been observed in many instances that the thermodynamic stability of the *E*-isomer often dictates product formation, favoring *E*-selectivity, especially in the absence of any opposing factor to challenge this stability. That's why synthetic methods reporting *Z*-selectivity and/or exclusive formation of *Z*-isomers are comparatively sporadic. Herein, we report a standalone amine-catalyzed chemo- and regioselective method to prepare hydrazones from readily available arenediazonium salts and active methylenes (Fig. 1e and f). Remarkably, the current aminocatalytic method unlocks access to stereoselective *Z*-hydrazones.

## Results

### Optimization of reaction parameters

Our search for reaction conditions began with the reaction of benzenediazonium salt (1a) with 1.0 equiv of 2,4-pentanedione (2a) using 5 mol% DABCO in toluene at room temperature. After 20 h, the anticipated cross-coupled product, 3-(2-phenylhydrazono)pentan-2,4-dione (3a), was obtained in an isolated yield of 33% (Table 1, entry 15). Changing the solvent nature not only improved the yield and TON of 3a (entries 9, 13, and 14) but also reduced the reaction time and increased the TOF (entries 5 and 9). The use of catalysts other than DABCO was also screened, but the results were found to be inferior (entries 6, 10, and 11). Increasing the catalyst loading to 10 mol% afforded 3a in 78% yield with high TON and TOF values (entry 1). Furthermore, changing the stoichiometry (entries 3 and 4), concentration (entries 7 and 8), and temperature (entry 16) were found to be ineffective (see SI for more details). In addition to TON and TOF, the reaction features other promising green chemistry metrics (for calculations of all these metrics, see page S6 of SI).

**Table 1:** Optimization of reaction parameters^a^

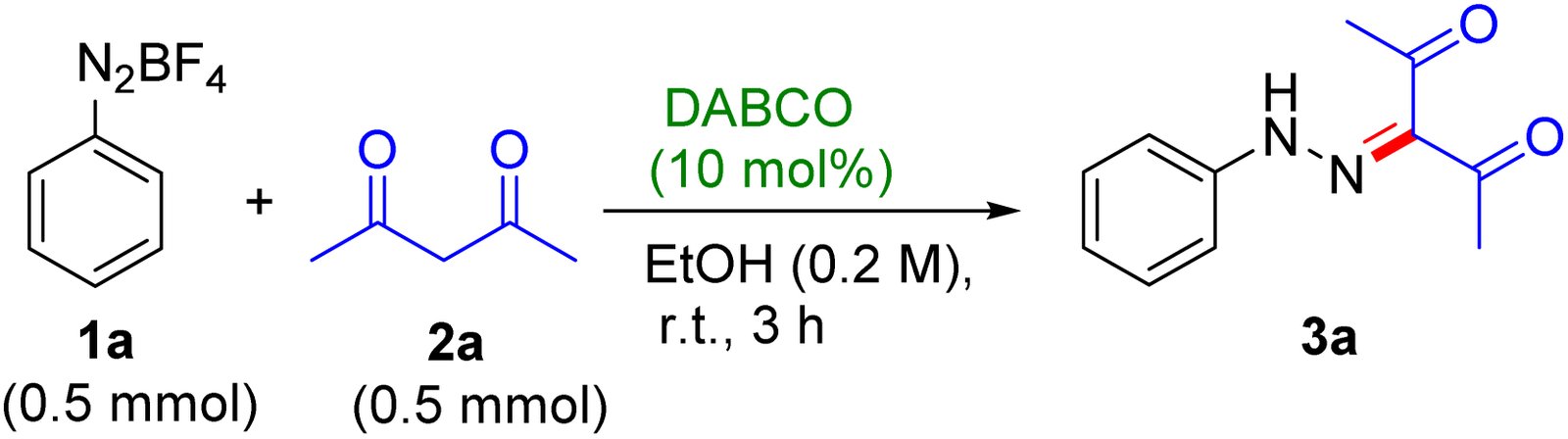				
Entry	Change in optimized conditions	3a (%)	TON	TOF (h^−1^)
**1**	**None**	**78**	**7.8**	**2.6**
2	Run for 2 h	67	6.7	3.4
3	1.2 equiv 1a	72	7.2	2.4
4	1.1 equiv 2a	61	6.1	2.0
5	5 mol% DABCO	51	5.1	1.7
6	5 mol% pyrrolidine	36	3.6	1.2
7	EtOH (0.17 M)	60	6.0	2.0
8	EtOH (0.25 M)	66	6.6	2.2
9^b^	5 mol% DABCO	88	7.0	0.4
10^b^	5 mol% DBU	65	6.5	0.3
11^b^	5 mol% Et_3_N	75	6.0	0.3
12^b^	5 mol% PPh_3_	29	2.3	0.1
13^b^	5% DABCO, H_2_O (0.2 M)	42	3.4	0.2
14^b^	5 mol% DABCO, CH_2_Cl_2_ (0.2 M)	78	6.3	0.3
15^b^	5 mol% DABCO, toluene (0.2 M)	33	2.6	0.1
16^b^	5 mol% DABCO at 40 °C	75	6.0	0.3
**17** c	**None**	**75**	**7.5**	**2.5**

a Isolated yield after column chromatography.

b Run on 0.2 mmol scale for 20 h.

c Run on 9.0 mmol scale.

### Substrate scope of arenediazonium tetrafluoroborates (1)

With the optimized conditions in hand, the substrate scope for C–H diazenylation of 2,4-pentandione (2a) with various arenediazonium tetrafluoroborates^13^ (1) was investigated (Scheme 1). A wide range of important moieties commonly employed in further synthetic transformations such as chloro (3g and 3p), bromo (3i), iodo (3j and 3v), ether (3b, 3d, and 3w), nitro (3e and 3s), keto (3f and 3t), triflouromethyl (3h), ester (3l), carboxyl (3m), and terminal alkyne (3x) were found amenable to the reaction conditions. In the case of terminal alkyne (*o*-ethynyl) substituted arenediazonium salt (1x), the chemoselective cleavage of methylene sp^3^ C–H bond was observed instead of the reactive sp C–H bond of the terminal alkyne. Generally, the reaction efficiency and product yields were found to be governed by neither electronic nor steric factors. Substituents on the *o*-positions of the arenediazonium salts caused no steric hindrance to the reaction, and the corresponding hydrazones were obtained in moderate to excellent yields (3s–3x). Both the electron-rich and electron-withdrawing moieties on the arenediazonium salts afforded moderate to excellent yields of the corresponding hydrazones. However, in the case of strong electron-donating groups such as *p*-methoxy and *p*-phenoxy, good yields of the corresponding hydrazones were obtained (3b and 3d). Whereas, in the case of weakly donating *p*- and *m*-alkyl substituents (3c, 3n, and 3o) and strong electron-withdrawing groups (3e, 3g, and 3h) on the arenediazonium salt, the corresponding hydrazones were afforded in excellent yields. The reaction was found to be insensitive to steric hindrance, allowing substituents to be installed at the *p*-, *m*-, and *o*-positions on the arenediazonium salts. Diazonium salts derived from α- and β-naphthylamines worked equally well, affording the corresponding hydrazones 3q and 3r in 69% and 79% yields, respectively. In all the examples studied, complete regio- and chemoselectivity was observed. In addition, no extrusion of nitrogen or a molecular fragment was observed in any of the cases.

**Scheme 1:**
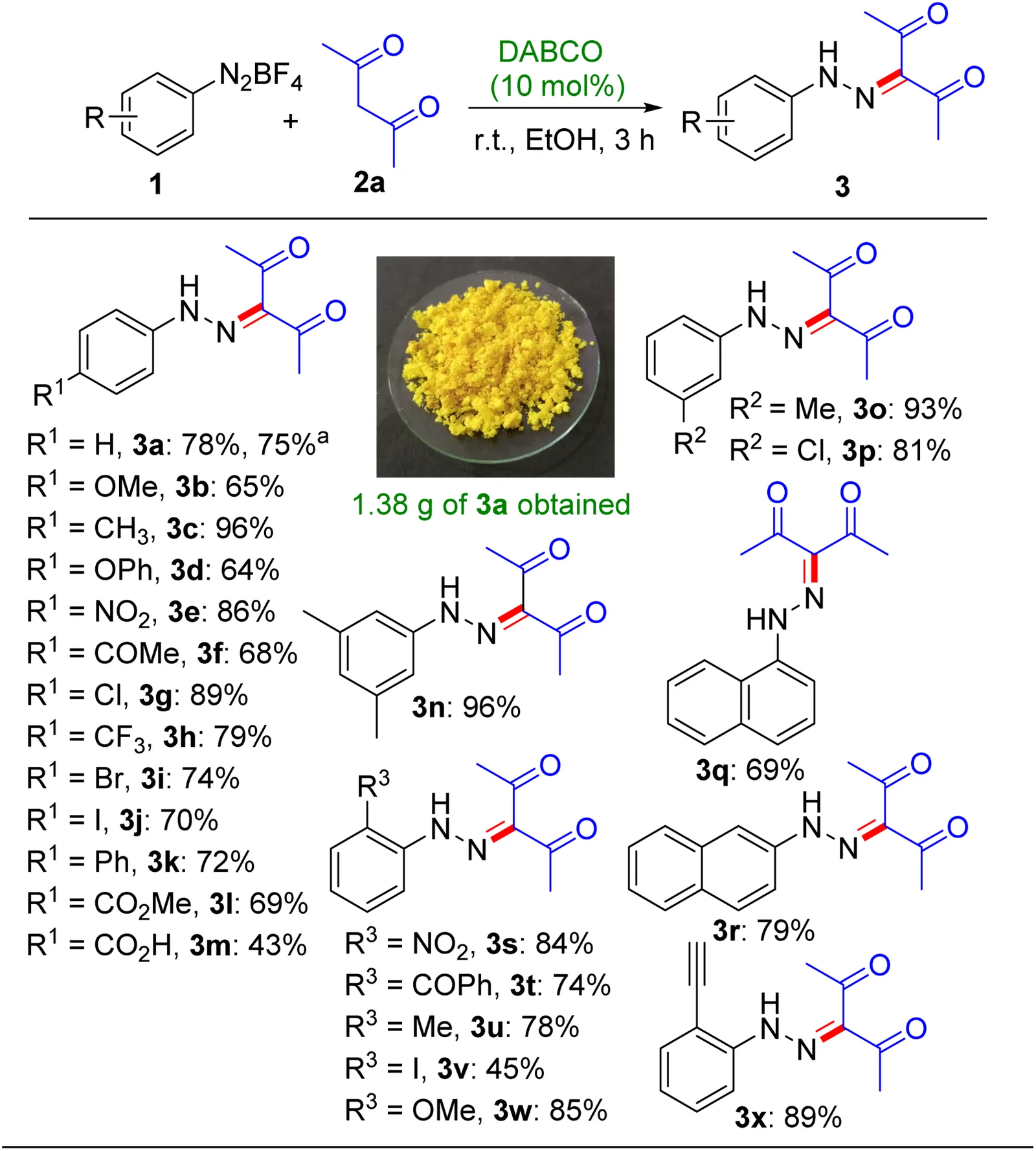
DABCO-catalyzed C–H amination of 2,4-pentandione (2a) with arenediazonium tetrafluoroborates (1): 1 (0.5 mmol), 2a (0.5 mmol), DABCO (10 mol%, 0.05 mmol), EtOH (0.2 M), r.t., 3 h. Yields shown are based on isolated products by column chromatography.^a^ reaction run on 9.0 mmol scale and 1.38 g of 3a was isolated.

### Substrate scope of active methylenes (2)

After this, we turned toward changing the structure of active methylenes under the optimized conditions (Scheme 2). A variety of active methylenes such as malononitrile 2c, trifluoroacetoacetate 2h, and benzoylacetonitrile 2g were smoothly converted to the corresponding hydrazones in good to excellent yields. The afforded hydrazones contain valuable moieties for various key structural transformations. Diketones other than 2a, such as 1,3-diphenylpropane-1,3-dione 2b, cyclohexane-1,3-dione 2d, and 1,3-indanedione 2e, were also found to be reactive under the optimized reaction conditions, giving the corresponding hydrazones (3y, 3aa, and 3ab) in 61%, 59%, and 64% yields, respectively. 3aa is used as an azo-dispersed dye.^14*a*^ Malononitrile (2c) reacted with 1a to give hydrazone 3z (used as an insecticide)^14*b*^ in 95% yield. In the case of unsymmetrical active methylenes, *E*- and *Z*-selectivity was found to depend on the substituents on the active methylenes and, to some extent, on the nature of the amine catalyst (3ad). A mixture of *E* and *Z*-isomers (3ac, 3ad) was afforded by reaction of 1a with methylacetoacetate (2f) and 3-oxo-3-phenylpropanenitrile (2g). Encouraged by these intriguing results and keeping in view the importance of trifluoroacetylated hydrazones,^15^1a was reacted with ethyl 4,4,4-trifluoroacetoacetate (2h). To our delight, a 100% stereoselective formation of the corresponding trifluoroacetylated *Z*-hydrazone, 3ae, was afforded in 60% yield. Further reactions of 2h with various substituted arenediazonium salts under optimized conditions afforded exclusively *Z*-selective hydrazones (**3af–3aq**). In all cases, the stereoselective formation of *Z*-hydrazones was confirmed by the downfield chemical shift of the N–H proton at *δ* 13 or further downfield owing to the deshielding effect of intramolecular H-bonding with the two O atoms of the ester moiety. The *Z*-configuration of 3ae was further confirmed by single-crystal X-ray crystallography (CCDC 2538913, Scheme 2). With 2h as an active methylene, the 100% *Z*-selectivity was found to be predominant and unaffected by the electronic and steric factors of the substituents attached to the arene rings of the diazonium salts (3ae–3aq). Thus, a substituent-switched control over the stereochemical outcome of the reaction was established.

**Scheme 2:**
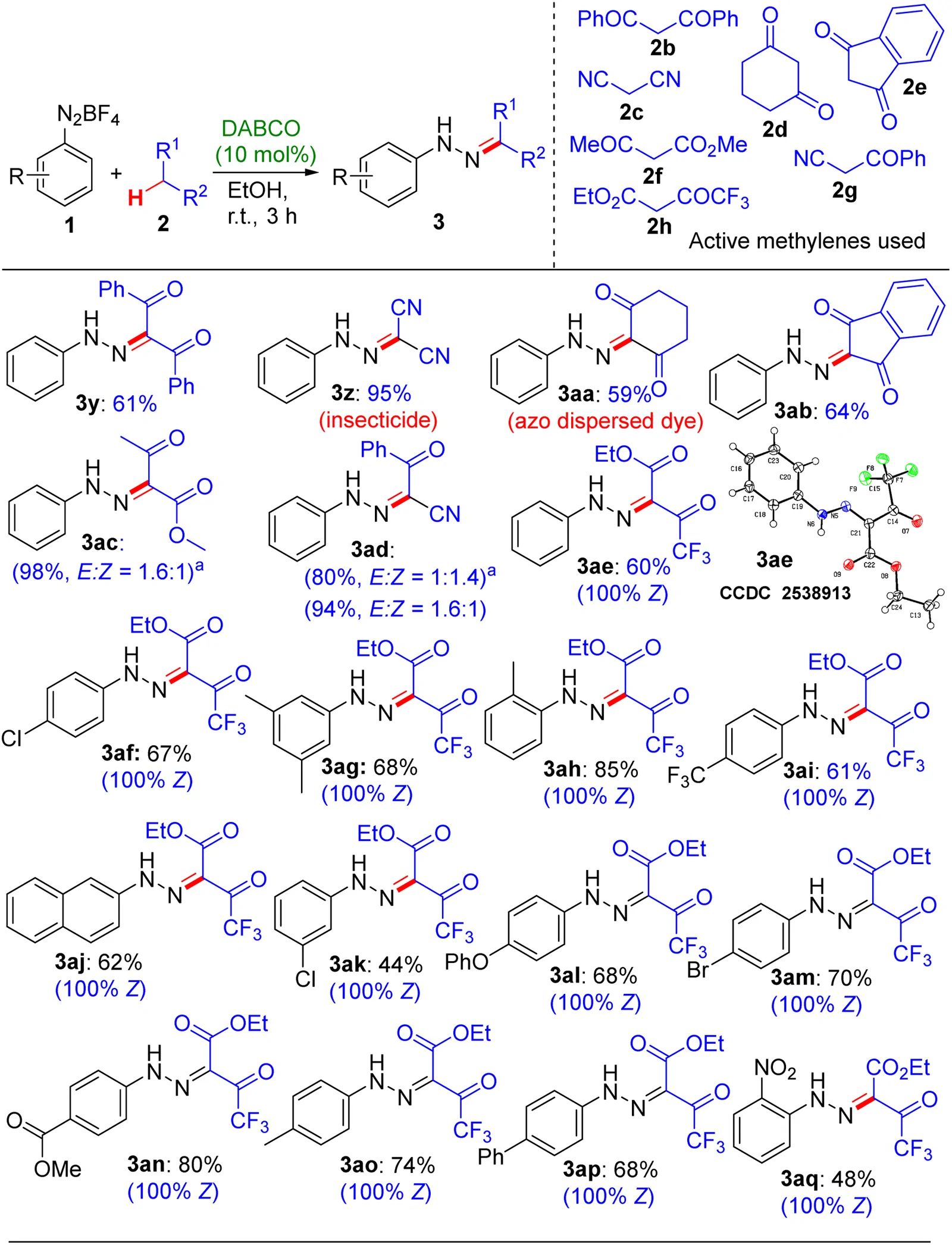
Aminocatalytic C–H amination of active methylenes with arenediazonium tetrafluoroborates: 1 (0.5 mmol), 2 (0.5 mmol), DABCO (10 mol%, 0.05 mmol), EtOH (0.2 M), r.t., 3 h. Yields shown are based on isolated products by column chromatography and *E*/*Z* ratio is shown in parentheses. ^*a*^ 20% pyrrolidine was used as catalyst.

### Application of the developed method to the synthesis of valuable targets

To further elaborate on the practical applicability of our aminocatalytic method, we applied it to synthesize 3b on a gram scale (Scheme 3a). A reaction of 10 mmol of 2a with *p*-methoxyarenediazonium salt afforded 1.54 g of 3b in 62% yield, showcasing the effectiveness and mildness of our reaction conditions for scale-up synthetic transformations. Remarkably, the scale-up reaction proceeded smoothly at room temperature. This result shows the improvement in energy usage offered by our method over previous base-mediated methods, in which cooling to 0 °C was required for scale-up reactions to dissipate the heat liberated during the stoichiometric acid–base reaction between base and an active methylene compound. After establishing this, 3b was reacted with hydroxylamine to afford *p*-methoxyphenylazoisoxazole 4a, which has applications as a photoswitchable adhesive material.^16*a*,*b*^ Finally, *N*-(4-chlorophenyl)carbonohydrazonoyl dicyanide, NCCD (Scheme 3b, product 3ar), was prepared by reacting *p*-chlorophenyl diazonium salt 1g with malononitrile 2c under optimized conditions in an excellent yield of 87%. It has been established that NCCD has high antimicrobial and anticancer activities, whereas its Zn complex has antioxidant potential.^16*c*^

**Scheme 3:**
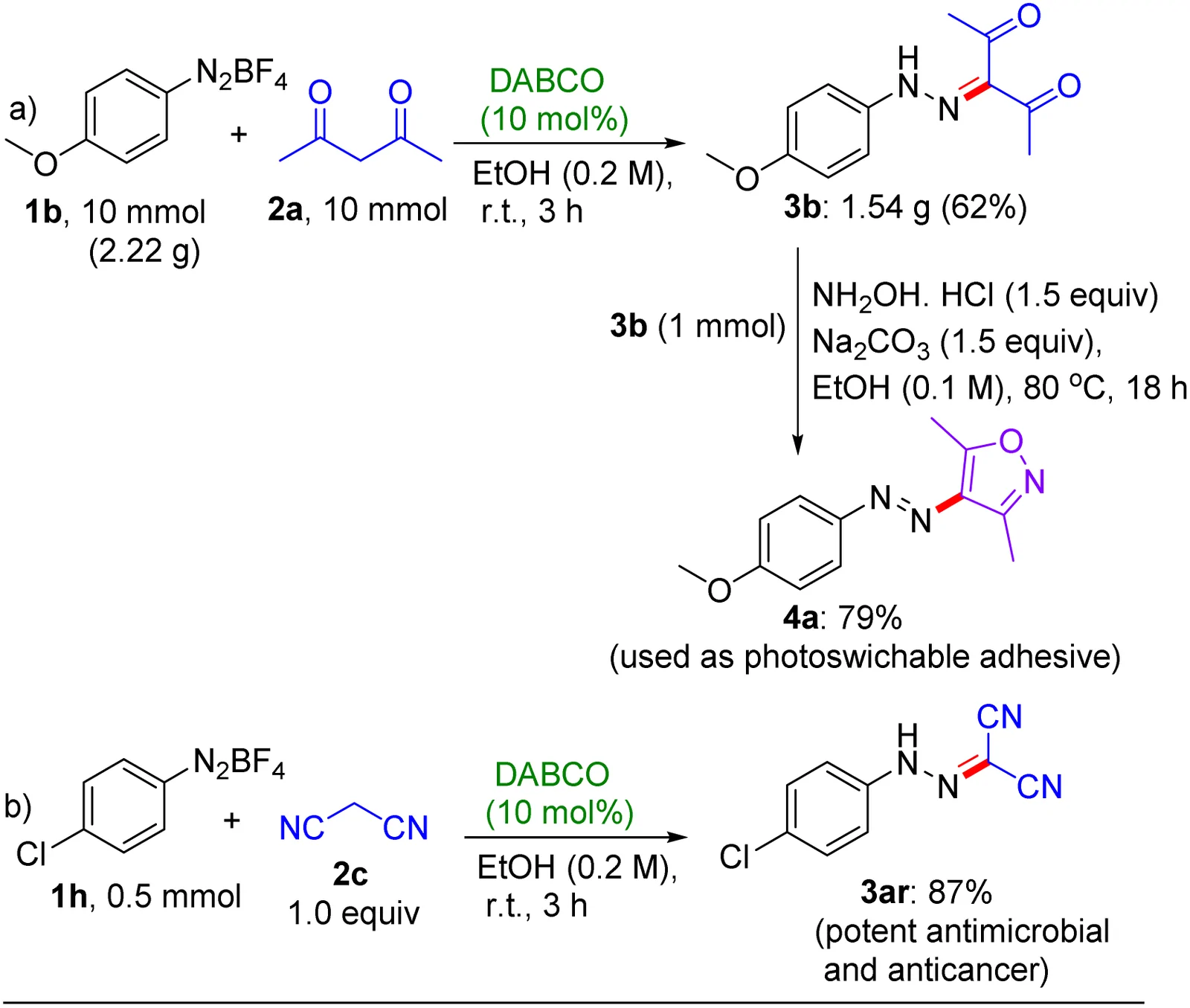
Gram-scale synthesis (a, 1^st^ step) and applications of synthesized hydrazones (a, 2^nd^ step and b).

### Mechanistic studies

To study the reaction pathway, a reaction of 1a with 2a was started without any organocatalyst. After 20 h, 3a was afforded in 24% yield (Scheme 4a). In this case, most probably the non-nucleophilic tetrafluoroborate counter anion, BF_4_^−^, acted as a catalytic species to shift the keto–enol equilibrium of active methylene (2a) to the enol side. Moreover, the role of intramolecular H-bonding cannot be ruled out. However, in the presence of a catalytic amine, a significant increase in the reaction rate was observed (Table 1, entry 1). Next, a reaction of 1a with 2a in the presence of 2 equiv of 2,2,6,6-tetramethylpiperidine-*N*-oxyl (TEMPO) under optimized conditions (Scheme 4b) afforded 82% of 3a, indicating a polar (ionic) mechanism for the reaction. Further evidences in favor of the polar pathway are: (a) the use of polar ethanol as a solvent to promote the reaction, and (b) excellent yields are afforded for strong electron-withdrawing groups (Scheme 1, products 3e, **3h**, and 3s) compared with strong electron-donating groups (products 3b and 3d). This is because the electron-withdrawing effect increases the electrophilicity of the intermediate arenediazonium ion A for nucleophilic attack by the enolate anion, and the electron-donating effect does the reverse.

**Scheme 4:**
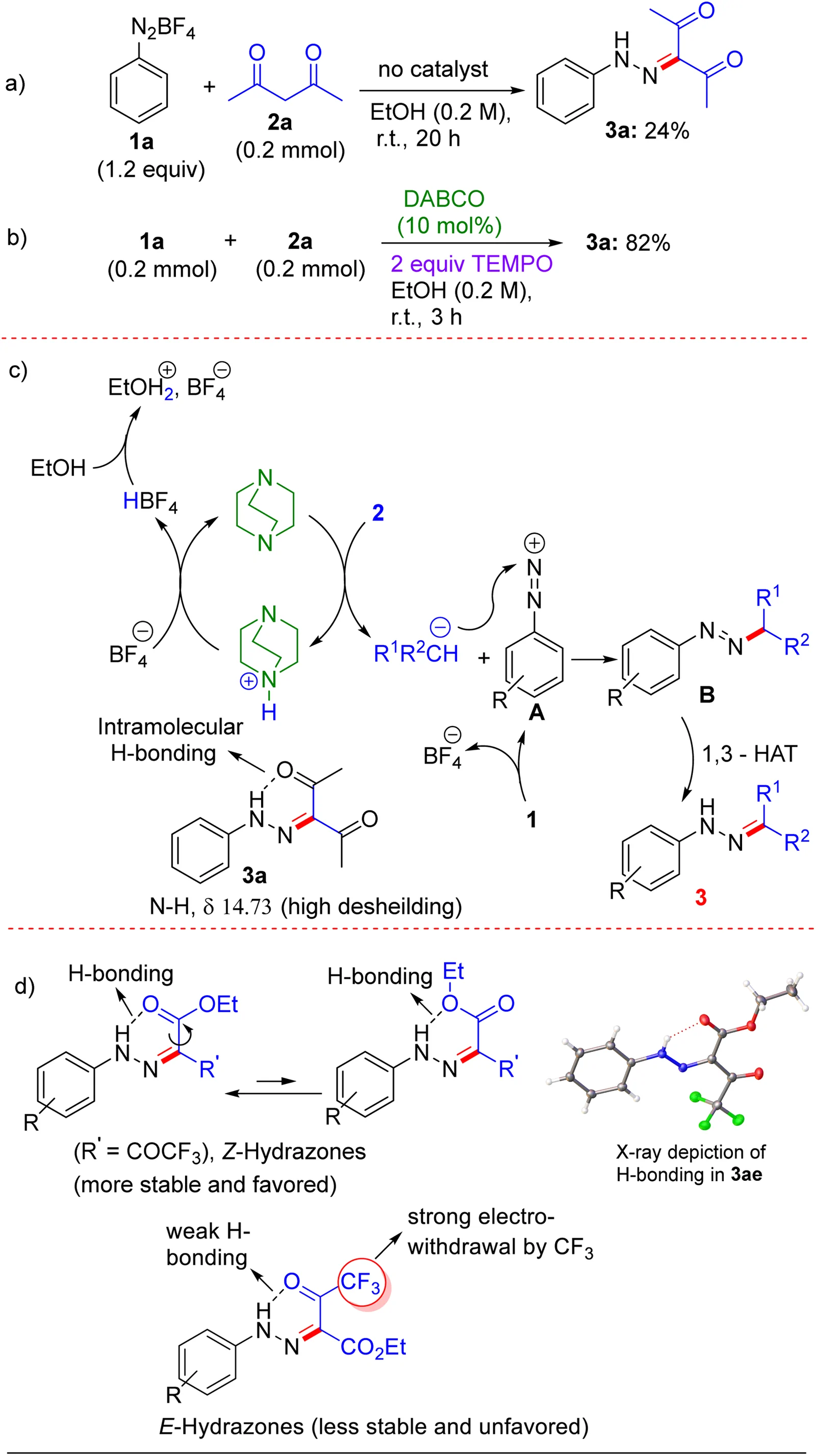
(a) and (b) Are control experiments, (c) mechanistic considerations, (d) depiction of intramolecular H-bonding-promoted exclusive Z-selectivity.

## Discussion

### Proposed mechanism

Keeping the above clues in mind, a polar mechanism, as depicted in Scheme 4c, is proposed. DABCO deprotonates the active methylene 2 to a carbanionic species, which reacts with arenediazonium ion A to produce a diazenyl intermediate B. Then, a 1,3-HAT in B produces hydrazone 3. The rearrangement of B to hydrazone 3 is highly facilitated by the intramolecular H-bonding in 3, which makes it more stable. The regeneration of DABCO occurs *via* the loss of a proton from protonated DABCO to a basic BF_4_^−^ ion present in the reaction mixture, liberating HBF_4_. This is then deprotonated by an ethanol molecule, regenerating the BF_4_^−^ anion for catalytic turnovers.

### Role of intramolecular H-bonding in determining stereoselectivity

The exclusive *Z*-selectivity of hydrazones 3ae–3aq (Scheme 2) can be explained based on intramolecular H-bonding of the N–H group with O-atoms (of both carbonyl and alkoxy) of the ester moiety, rendering *Z*-hydrazones more stable (Scheme 4d). Such an interaction is less favorable for the corresponding *E*-hydrazone because, in that case, the strong electron-withdrawing trifluoromethyl group makes the carbonyl O-atom electron-deficient and the intramolecular H-bonding weaker. Therefore, the relative energy decreases for *Z*-hydrazones compared to the corresponding *E*-isomer. Quantum mechanical calculations also support the above reasoning.^15*a*^ The evidence for intramolecular H-bonding interactions and exclusive formation of *Z*-isomers (3ae–3aq) is provided by the downfield chemical shift value of the N–H proton at *δ* 13 or even further downfield in their ^1^H NMR spectra due to deshielding caused by the *cis*-ester moiety. For *E*-isomers with relatively weak H-bonding, the N–H proton resonance is observed at about 11–13 ppm (*e.g.*, *δ* 12.75 for hydrazone 3ac).

## Conclusions

In summary, the first aminocatalytic diazenylation of readily available active methylenes has been developed, which affords diversely decorated hydrazones. The reaction allows access to several arylhydrazones that were previously inaccessible by existing methods. In addition, the use of a commercially available and inexpensive organocatalyst, along with the mild conditions, operational simplicity of the reaction, and gram scalability, makes our protocol synthetically attractive. The induction of stereoselectivity *via* making use of intramolecular H-bonding is highly intriguing for the development of new aminocatalytic stereoselective transformations that are currently underway in our laboratory. Furthermore, recycling the amine catalyst and the use of biomass/natural products-derived amines as catalysts can provide further opportunities for the development of future green approaches.

### Synthetic procedures

In a reaction tube, active methylene, 2 (0.5 mmol) was added to arenediazonium tetrafluoroborate, 1 (1.0 equiv, 0.5 mmol). Then, DABCO (10 mol%, 0.05 mmol, 5.6 mg) and EtOH (2.5 mL) were further added, and the tube was closed with a rubber septum under air. The resulting mixture was stirred at room temperature for 3 hours. On completion, the reaction mixture was transferred to a round-bottom flask with ethyl acetate, then concentrated using a rotary evaporator. The residue was purified by column chromatography on silica gel using a mixture of *n*-hexane/EA to give the desired hydrazones (3).

Alternatively, the reaction mixture was precipitated by adding water (10 mL), cooled, filtered through a sintered glass crucible, and washed with water (10 mL × 3). The residue was dried to afford 70.5 mg, 69% of 3a.

## Author contributions

Conceptualization, S. A.; methodology, S. A., A., A. D., S. O., and M. A.; investigation, S. A., A., A. D., M. A., H. K., S. S., S. O., M. I., and Q. U.; writing – original draft, A., M. I., S. A., and S. A. A. S.; writing – review & editing, S. A., A., and S. A. A. S.; funding acquisition, S. A., S. S., and F. A.; resources, S. A., H. K., and F. A.; supervision, S. A. and S. A. A. S.

## Conflicts of interest

There are no conflicts to declare.

## Supplementary Material

RA-016-D6RA07931H-s001

RA-016-D6RA07931H-s002

## Data Availability

CCDC 2538913 contains the supplementary crystallographic data for this paper.^17^ The data supporting this article have been included as part of the supplementary information (SI). Supplementary information is available. See DOI: https://doi.org/10.1039/d6ra07931h.
