# Supplementary material for: Organocatalytic stereoselective C–H diazenylation of active methylenes with arenediazonium salts
Source: RSC Adv. 2026 Sep 8;16(43):53445–53. doi: 10.1039/d6ra07931h (PMC13551556; doi:10.1039/d6ra07931h)
Supplement: Supplementary file 1 [file RA-016-D6RA07931H-s001.pdf]

Supplementary Information (SI) for RSC Advances.
This journal is © The Royal Society of Chemistry 2026

Supporting Information

Organocatalytic Stereoselective C-H diazenylation of Active Methylenes with Arenediazonium Salts

Aqsa[a], Allaud Din[a], Mehtab Ahmad[a], Hammad Khan[a], Muhammad Israr[b], Syed Osama[a], Qudrat Ullah[a], Sumbal Saba[c], Zi-Sheng Chen[d,*], Syed Adnan Ali Shah[e,f,*], and Shaukat Ali[a,*]

[a] Organic Synthesis and Catalysis Research Laboratory, Institute of Chemical Sciences, University of Peshawar, Peshawar 25120, Khyber Pakhtunkhwa, Pakistan.
[b] Center for Integrative Petroleum Research (CIPR), King Fahd University of Petroleum & Minerals, 31261, Dhahran, Saudi Arabia.
[c] Laboratório de Síntese Sustentável e Organocalcogênio (LABSO), Instituto de Química (IQ), Universidade Federal de Goiás—UFG, Goiânia 74690-900, GO, Brazil.
[d] Shaanxi Key Laboratory of Natural Products & Chemical Biology, College of Chemistry and Pharmacy, Northwest A&F University, Yangling 712100 Shaanxi, P. R. China.
[e]Faculty of Pharmacy, Universiti Teknologi MARA (UiTM) Cawangan Selangor, Kampus Puncak Alam, Bandar Puncak Alam 42300, Selangor Darul Ehsan, Malaysia.
[f]Atta-ur-Rahman Institute for Natural Product Discovery (AuRIns), Universiti Teknologi MARA (UiTM) Cawangan Selangor Kampus Puncak Alam, Bandar Puncak Alam 42300, Selangor Darul Ehsan, Malaysia.
*Correspondence: drshaukatali@uop.edu.pk (S.A), syedadnan@uitm.edu.my (S.A.A.S)

Table of Contents

1. General Information S3
2. Experimental Procedures S4
   2.1 General procedure for the preparation of arendiazonium tetrafluoroborates S4
   2.2 Search for optimum reaction conditions S3
   2.3 General procedure for the aminocatalytic synthesis of hydrazones S5
   2.4 Scale-up reaction for 3-(2-phenylhydrazono)pentane-2,4-dione (**3a**) S5
   2.5 Green chemistry metrics calculations for hydrazone **3a** S6
   2.6 Scale-up reaction for 3-(2-(4-Methoxyphenyl)hydrazono)pentane-2,4-dione (**3b**) S7
3. Characterization Data of Products S7
4. Mechanistic Studies S18
   4.1 Reaction without a catalyst S18
   4.2 Radical scavenging experiment with TEMPO S18
5. X-ray Crystallographic data of **3ae** S18
6. References S20
7. NMR Spectra of Products S22

1. General Remarks

Unless otherwise noted, all reactions were carried out in oven-dried reaction vessels with Teflon screw caps or rubber septa under air. All commercially available reagents were obtained from chemical suppliers and used as received. The starting benzenediazonium tetrafluoroborate salts (**1**) were freshly prepared from the corresponding anilines using the literature procedure.[1a-b] All the active methylenes (**2**) used in this work are commercially available. Column chromatography was performed on silica gel (70-230 mesh) with the indicated solvent mixtures. The progress of reactions was monitored using Merck aluminum TLC plates coated with 0.2 mm silica gel 60 F-254 and visualized with 254 nm UV light.

The ^{1}H NMR and ^{13}C NMR spectra were recorded on a Bruker Avance III 500, 600 Ascend, and Avance Neo 500 spectrometers. ^{1}H NMR chemical shifts (δ) were reported as parts per million (ppm) downfield from tetramethylsilane (δH = 0 ppm), and ^{13}C NMR chemical shifts were reported in ppm by considering the solvent resonance as the internal standard ($CDCl_3$, δC = 77.0 ppm; DMSO-d_6, δC = 39.50 ppm) downfield from tetramethylsilane. The following abbreviations were used to identify the multiplicities: s for singlet, d for doublet, t for triplet, q for quartet, dd for doublet of doublets , td for triplet of doublets, tt for triplet of triplets, dq for doublet of quartets, and m for multiplet, br for broad, and all combinations thereof can be explained by their integral parts.

HRMS values were measured by direct infusion of the samples using a Q-Exactive ThermoScientific mass spectrometer with an H-ESI source. LCMS values were measured with LC/MS-QTOF system which comprised of an Agilent 1200 liquid chromatography system, equipped with a binary pump, a vacuum degasser unit, an auto sampler and 6520 quadrupole time of flight mass spectrometers with Agilent electrospray ionization (ESI) source. Column used was Agilent ZORBAX Eclipse Plus C18 Rapid Resolution HT (2.1 x 100 mm) 1.8 µm. MS values were determined with JEOL JMS-600H using direct probe.

2. Experimental Procedures

2.1 General procedure for the preparation of arendiazonium tetrafluoroborates (1a-1x)

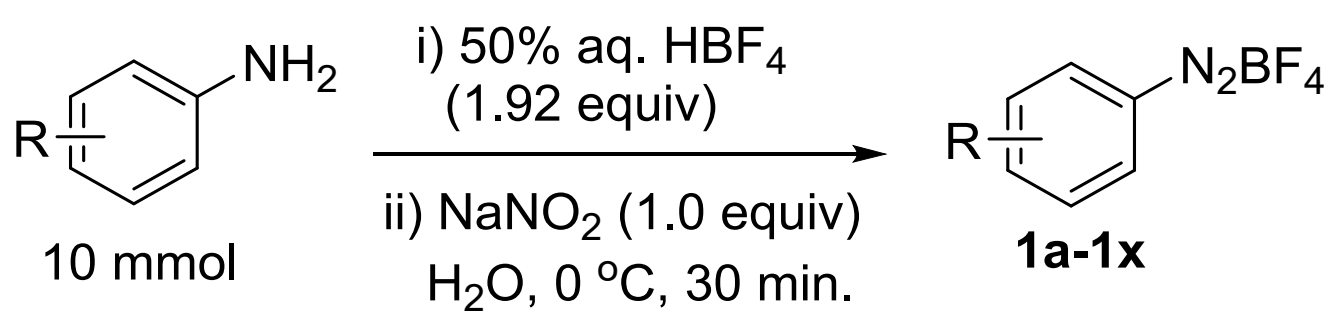

The reported protocols[1a-b] were followed for the synthesis of aryldiazonium tetrafluoroborates (**1a-1x**). In a typical procedure, 10 mmol of the corresponding aniline was mixed with water (4 mL) and 50% aqueous hydrofluoroboric acid (19.3 mmol, 1.92 equiv) at room temperature. The mixture was cooled to 0 °C, and a solution of $NaNO_2$ (10 mmol in 1.5 mL of water) was added slowly. The resulting reaction mixture was stirred at 0 °C for 30 min, and the precipitate was collected by filtration. The solid product was dissolved in minimum acetone and reprecipitated using diethyl ether to yield aryldiazonium tetrafluoroborate, which was dried under vacuum and used without further purification

2.2 Search for optimum reaction conditions

Table 1. Optimization of reaction conditions

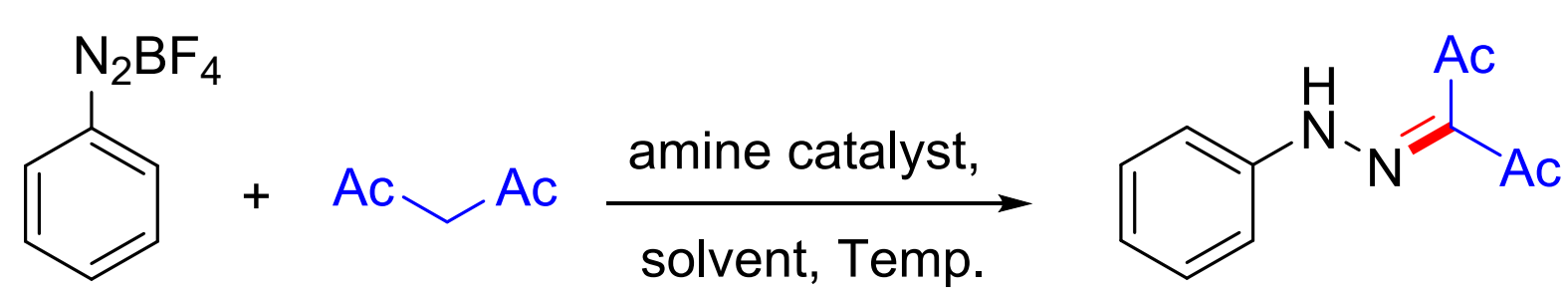

entry no.	1a (mmol)	2a (equiv)	Solvent (mL)	catalyst (mol%)	time (h)	Temp. (°C)	3a, Isolated yield (%)[a]
1	0.2	1.0	Toluene (1)	DABCO (5)	20	rt	33%
2	0.2	1.0	H_2O (1)	DABCO (5)	20	rt	42%
3	0.2	1.0	DMF (1)	DABCO (5)	20	rt	74%
4	0.2	1.0	DCM (1)	DABCO (5)	20	rt	78%
5	0.2	1.0	EtOH (1)	DABCO (5)	20	rt	88%
6	0.2	1.0	EtOH (1)	NEt_3 (5)	20	rt	75%
7	0.2	1.0	EtOH (1)	DBU (5)	20	rt	65%
8	0.2	1.0	EtOH (1)	Pyrrolidine (5)	20	rt	66%
9	0.5	1.0	EtOH (2.5)	Pyrrolidine (5)	3	rt	36%
10	1.2 equiv	0.5 mmol	EtOH (2.5)	Pyrrolidine	3	rt	80%

				(20)			
11	0.2	1.0	EtOH (1)	PPh_3 (5)	20	rt	29%
12	0.2	1.0	EtOH (1)	NaOAc (5)	20	rt	66%
13	0.2	1.0	EtOH(1.5)	DABCO (5)	20	rt	80%
14	0.2	1.0	EtOH (0.5)	DABCO (5)	20	rt	44%
15	0.2	1.0	EtOH (1)	DABCO (5)	20	40	75%
16	0.2	1.0	EtOH (1)	DABCO (5)	20	60	36%
17	0.2	1.0	EtOH (1)	DABCO (5)	20	80	13%
18	0.24	1.0	EtOH (1)	DABCO (5)	20	rt	64%
19	0.2	1.2	EtOH (1)	DABCO (5)	20	rt	48%
20	0.2	1.0	EtOH (1)	-	20	rt	24%
21	0.5	1.0	EtOH/H_2O (2.5), 1:1	DABCO (5)	20	rt	40%
22	0.5	1.0	EtOH (2.5)	DABCO (5)	3	rt	51%
23	1.2 equiv	0.5 mmol	EtOH (2.5)	DABCO (5)	6	rt	52%
24	1.2 equiv	0.5 mmol	EtOH (2.5)	DABCO (10)	3	rt	72%
**25**	**0.5 mmol**	**0.5 mmol**	**EtOH (2.5)**	**DABCO (10)**	**3**	**rt**	**78%, 69%[b]**
26	0.5 mmol	0.5 mmol	EtOH (3mL)	DABCO(10)	3	rt	60%
27	0.5 mmol	0.5 mmol	EtOH (2)	DABCO (10)	3	rt	66%
28	0.5 mmol	0.55 mmol	EtOH (2.5)	DABCO (10)	3	rt	61%
29	0.5 mmol	0.5 mmol	EtOH (2.5)	DABCO (10)	4	rt	77%
30	0.5 mmol	0.5 mmol	EtOH (2.5)	DABCO (10)	2	rt	67%
**31**	**9.0 mmol**	**9.0 mmol**	**EtOH (2.5)**	**DABCO (10)**	**3**	**rt**	**75%**

[a] via column chromatography [b] via filtration

2.3 General procedure for the aminocatalytic synthesis of hydrazones:

In a reaction tube, active methylene (**2**, 0.5 mmol) was added to arenediazonium tetrafluoroborate (**1**, 1.0 equiv., 0.5 mmol). Then, DABCO (10 mol%, 0.05 mmol, 5.6 mg) and EtOH (2.5 mL) were further added, and the tube was sealed with a rubber septum. The resulting mixture was stirred at room temperature for 3 hours under air. On completion, the reaction mixture was transferred to a round-bottom flask with ethyl acetate, then concentrated using a rotary evaporator. The residue was purified by column chromatography on silica gel using a mixture of n-hexane/EA to give the desired hydrazones **(3).**

Alternatively, the reaction mixture was precipitated by adding water (10 mL), cooled, filtered through a sintered glass crucible, and washed with water (10 mL x 3). The residue was dried to afford 70.5 mg, 69% of **3a**.

2.4 Scale-up reaction for 3-(2-phenylhydrazono)pentane-2,4-dione (3a)

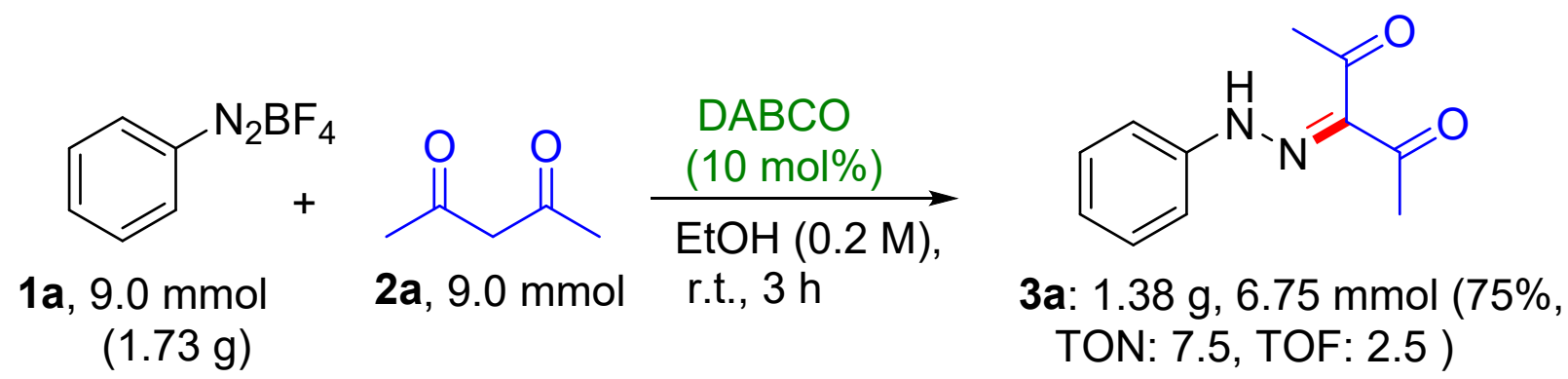

9.0 mmol, 1.73 g of benzenediazonium tetrafluoroborate (**1a**), acetylacetone (**2a**, 9.0 mmol, 0.93 mL), DABCO (10 mol%, 0.9 mmol, 101 mg), and 45 mL of EtOH were added to a 100 mL round-bottom flask fitted with a reflux condenser and stirred at room temperature for 3 h under air. The reaction mixture was transferred to a round-bottom flask with ethyl acetate, then concentrated using a rotary evaporator. The residue was purified by column chromatography on silica gel using a mixture of n-hexane/EA (30:1→20:1) to give 1.38 g (6.75 mmol, 75%) of **3a.**

2.5 Green chemistry metrics calculations[1c-e] for hydrazone 3a

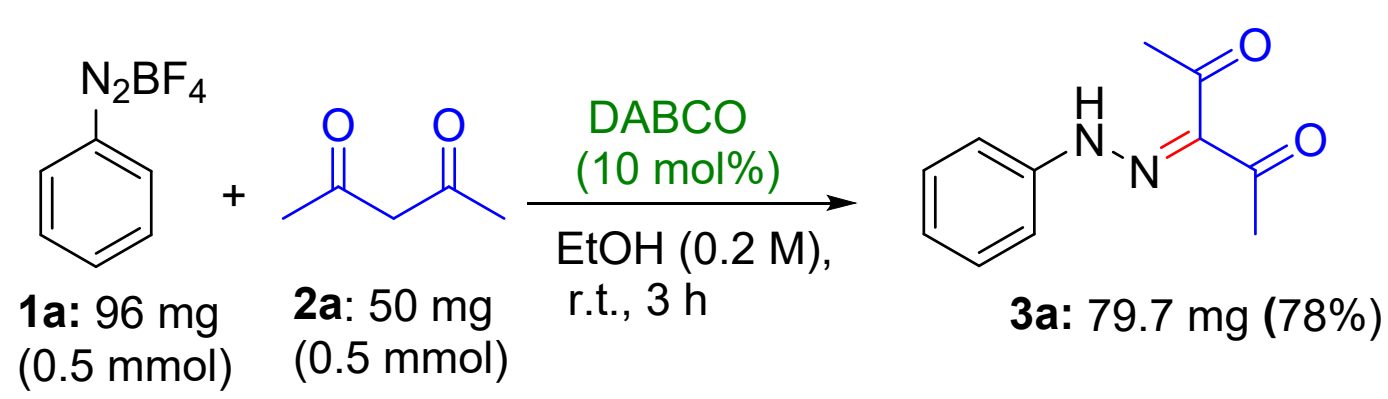

a) To calculate % atom economy, we used the above stoichiometric reaction

Atom Economy (%) =

$$\frac{Molar\ mass\ of\ product\ 3a}{\sum Molar\ masses\ of\ 1a\ and\ 2a} \times 100 = \frac{204.23}{191.92\ +\ 100.117} \times 100 = \frac{204.23}{292.037} = 70\%$$

b) % Atom efficiency (AEF) = $Atom\ Economy \times \%Yield = 70\ \times 78 = 54.6\%$

c) % Reaction mass efficiency (RME) =

$$\frac{mass\ of\ isolated\ product\ 3a}{\sum masses\ of\ 1a\ and\ 2a\ used} \times 100\ =\ \frac{79.7}{96\ + 50} \times 100 =\ 54.5\%$$

d) % Optimum efficiency (OE) = $\frac{RME}{AE} x100 = \frac{54.5}{70} x\ 100 = 78\%$

e) To calculate the environmental factor (*E*-factor), first, we calculate the total mass of all the inputs used to get 79.7 mg of product **3a** according to the above reaction

mass of 0.5 mmol of **1a** = 96 mg
mass of 0.5 mmol of **2a** = 50 mg
mass of 10 mol% DABCO = 5.6 mg
mass of 2.5 ml ethanol = 1973.63 mg
Now,

E-Factor = $$\frac{Total\ mass\ of\ inputs - Mass\ of\ product}{Mass\ of\ product} = \frac{96 + 50 + 5.6 + 1973.63 - 79.7\ mg}{79.7} = \frac{2045.53\ mg}{79.7\ mg} = 25.7$$

f) Process Mass Intensity (PMI) =
$$\frac{Total\ mass\ of\ all\ materials\ used}{Mass\ of\ product} = \frac{96 + 50 + 5.6 + 1973.63}{79.7} = \frac{2125.23}{79.7} = 26.7$$

g) Mass Intensity (MI) =
$$\frac{Total\ mass\ of\ all\ inputs\ excluding\ water}{Mass\ of\ product} = \frac{96 + 50 + 5.6 + 1973.63}{79.7} = \frac{2125.23}{79.7} = 26.7$$

h) % Mass productivity (MP) = $$\frac{1}{MI}x100 = \frac{1}{26.7}x100 = 4\%$$

i) Solvent Intensity (SI) =
$$\frac{Total\ mass\ of\ all\ solvents\ used\ excluding\ water}{Mass\ of\ product} = \frac{1973.63\ mg}{79.7\ mg} = 25$$

j) Turn over number (TON) = $$\frac{Total\ mmols\ of\ 3a\ formed}{mmols\ of\ DABCO\ used} = \frac{0.39}{0.05} = 7.8$$

k) Turn over frequency (TOF) = $$\frac{TON}{time(h)} = \frac{7.8}{3\ h} = 2.6\ (/h)$$

2.6 Scale-up reaction for 3-(2-(4-Methoxyphenyl)hydrazono)pentane-2,4-dione (3b)

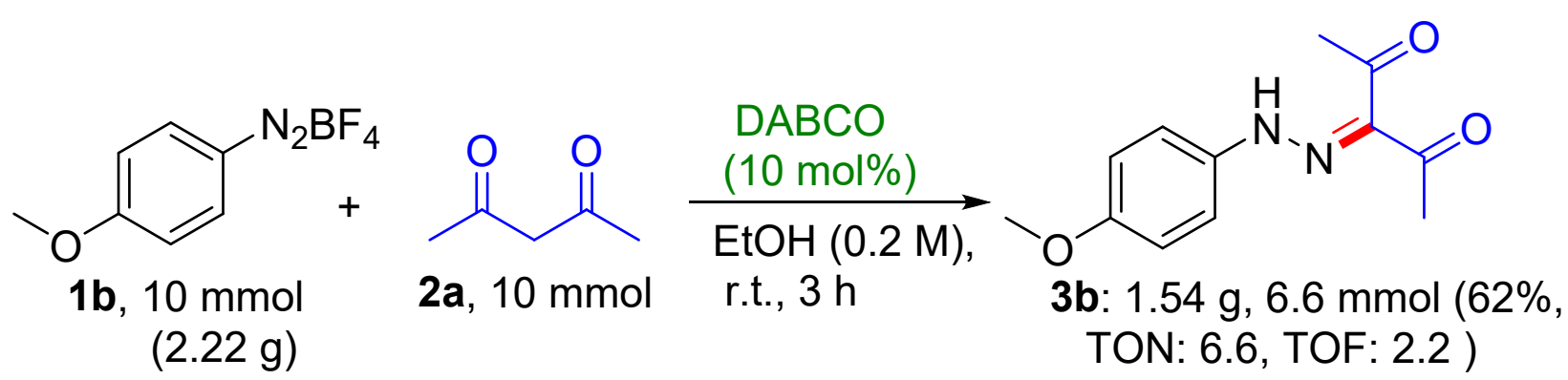

10 mmol, 2.22g of *p*-methoxyphenyldiazonium tetrafluoroborate (**1b**), acetylacetone (**2a**, 10 mmol, 1.03 mL), DABCO (10 mol%, 1 mmol, 112.2 mg), and 50 mL of EtOH were added to a 100 mL round-bottom flask fitted with a reflux condenser and stirred at room temperature for 3 h under air. The reaction mixture was transferred to a round-bottom flask with ethyl acetate, then concentrated using a rotary evaporator. The residue was purified by column chromatography on silica gel using a mixture of n-hexane/EA (30:1→10:1) to give 1.54 g, 6.6 mmol (62%) of **3b.**

3. Characterization data of products

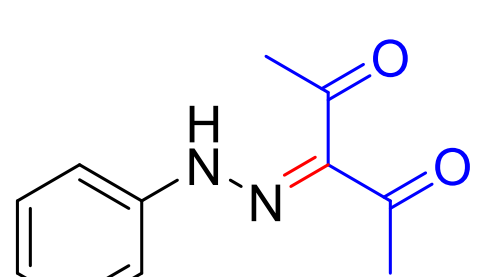

3a

3-(2-Phenylhydrazono)pentane-2,4-dione (3a)[2]**:** Prepared by reacting benzenediazonium tetrafluoroborate (**1a**, 0.5 mmol, 96 mg) and acetylacetone (**2a**, 0.5 mmol, 51.4 μL) following the above general

procedure. Purification by column chromatography on silica gel with *n*-hexane/EtOAc (50/1→20/1) yielded **3a** (79.65 mg, 0.39 mmol, 78%) as a yellow solid.

^{1}H NMR (500 MHz, $CDCl_3$) δ 14.73 (s, 1H), 7.41 (d, *J* = 4.4 Hz, 4H), 7.22-7.19 (m, 1H), 2.60 (s, 3H), 2.49 (s, 3H). **^{13}C NMR** (125 MHz, $CDCl_3$) δ 197.92, 197.08, 141.51, 133.20, 129.64, 125.87, 116.24, 31.64, 26.60.

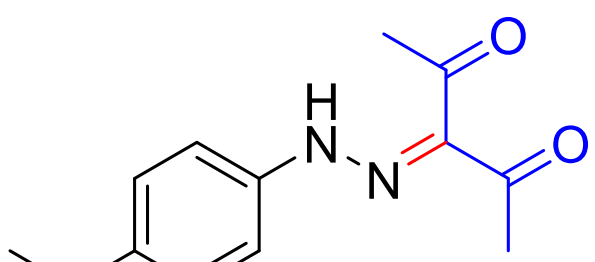

3b

3-(2-(4-Methoxyphenyl)hydrazono)pentane-2,4-dione (3b)[3,8]: Prepared by reacting *p*-methoxybenzenediazonium tetrafluoroborate salt (**1b**, 0.5 mmol, 111 mg) and acetylacetone **(2a**, 0.5 mmol, 51.4 μL) following the above general procedure. Purification by column chromatography on silica gel with n-hexane/EtOAc: 50/1→20/1 yielded **3b** (76.2 mg, 0.325 mmol, 65%) as a yellow solid.

^{1}HNMR (500 MHz, $CDCl_3$) δ 14.96 (s, 1H), 7.36 (d, *J* = 9 Hz, 2H), 6.94 (d, *J* = 9 Hz, 2H), 3.83 (s, 3H) 2.59 (s, 3H), 2.47(s, 3H). ^{13}C NMR (125 MHz, $CDCl_3$) δ 197.56, 196.93, 158.01, 135.08, 132.68, 117.62, 114.90, 55.56, 31.50, 26.57.

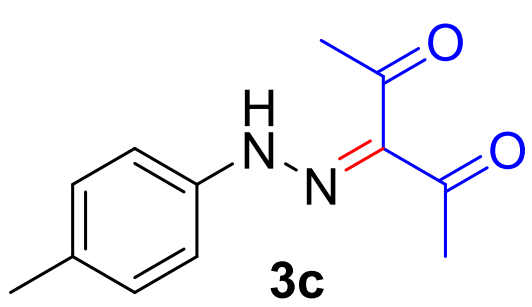

3c

3-(2-(*p*-Tolyl)hydrazono)pentane-2,4-dione (3c)[2,4]: Prepared by reacting *p*-methylbenzenediazonium tetrafluoroborate salt (**1c**, 0.5 mmol, 104 mg) and acetylacetone (**2a**, 0.5 mmol, 51.4 μL) following the above general procedure. Purification by column chromatography on silica gel with *n*-hexane/EtOAc: 50/1→20/1 yielded **3c** (104.76 mg, 0.48 mmol, 96%) as an orange-yellow solid.

^{1}H NMR (500 MHz, $CDCl_3$) δ 14.71 (s, 1H), 7.22-7.21 (m, 2H), 7.12 (d, *j* = 6.8 Hz, 2H), 2.51(s, 3H), 2.39 (s, 3H), 2.27 (s, 3H). **^{13} C NMR** (125 MHz, $CDCl_3$) δ 197.64, 196.99, 139.15, 135.87, 132.80, 130.12, 116.16, 31.51, 26.52, 20.92.

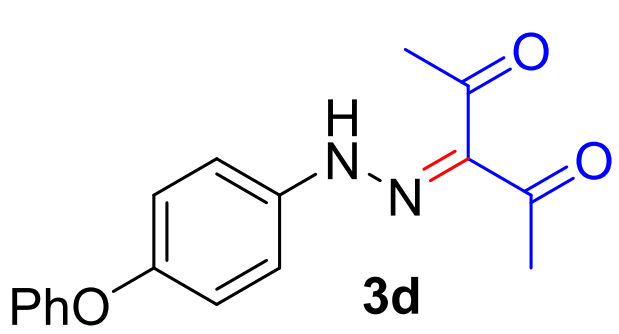

3d

3-(2-(4-phenoxyphenyl)hydrazono)pentane-2,4-dione(3d):
Prepared by reacting *p*-phenoxybenzenediazonium tetrafluoroborate salt (**1d**, 0.5 mmol, 142 mg) and acetylacetone (**2a**, 0.5 mmol, 51.4 μL) following the above general procedure. Purification by column chromatography on silica gel with *n*-hexane/EtOAc: 50/1→20/1 yielded **3d** (94.83 mg, 0.32 mmol, 64%) as a light yellow solid.

^{1}H NMR (500 MHz, $CDCl_3$) δ 14.76 (s, 1H), 7.27-7.22 (m, 4H), 7.02-6.99 (m, 1H), 6.94-6.89 (m, 4H), 2.48(s, 3H), 2.35 (s, 3H). **^{13}C NMR** (125 MHz, $CDCl_3$) δ 197.57, 196.71, 156.81, 155.21, 136.91, 132.83, 129.70, 123.42, 119.75, 118.67, 117.48, 31.42. 26.40. **LCMS (ESI)** calculated for $C_{17}H_{17}N_2O_3^+$([M+H]$^+$): 297.1234, found: 297.1268.

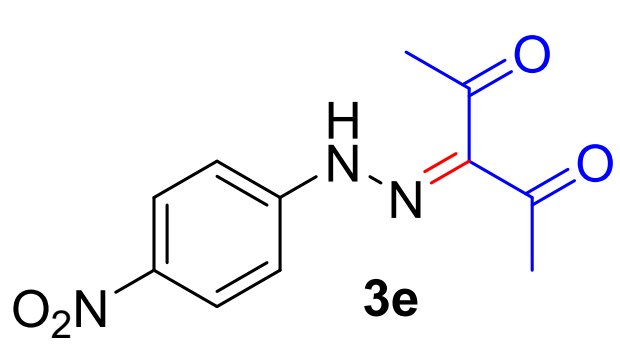

3e

3-(2-(4-nitrophenyl)hydrazono)pentane-2,4-dione (3e)[2]: Prepared by reacting 4-nitrobenzenediazonium tetrafluoroborate salt (**1e**, 0.5 mmol, 118.5 mg) and acetylacetone (**2a)** (0.5 mmol, 51.4 μL) following the above general procedure. Purification by column chromatography on silica gel with *n*-hexane/EtOAc: 50/1→10/1 yielded **3e** (107.2 mg, 0.43 mmol, 86%) as an orange solid.

^{1}**H NMR** (500 MHz, $CDCl_3$) δ 14.52 (s, 1H), 8.30 (d, *J* = 8.8 Hz, 2H), 7.50 (d, *J* = 8.7 Hz, 2H), 2.63 (s, 3H) 2.53(s, 3H). ^{13}**C NMR** (125 Hz, $CDCl_3$) δ 198.70, 196.79, 146.59, 144.55, 135.01, 125.79, 115.8031.84, 26.65.

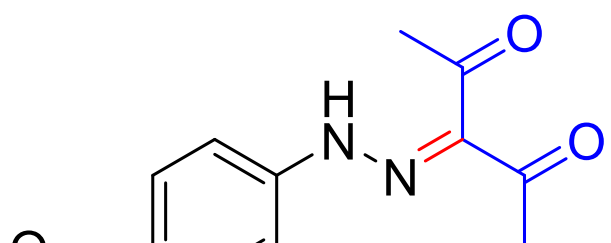
3f

3-(2-(4-Acetylphenyl)hydrazono)pentane-2,4-dione (3f)[5]: Prepared by reacting *p*-acetylbenzenediazonium tetrafluoroborate salt (**1f**, 0.5 mmol, 117 mg) and acetylacetone (**2a**, 0.5 mmol, 51.4 µL) following the above general procedure. Purification by column chromatography on silica gel with *n*-hexane/EtOAc: 50/1→20/1 yielded **3f** (83.8 mg, 0.34 mmol, 68%) as a pale yellow solid.

^{1}**H NMR** (500 MHz, $CDCl_3$) δ 14.58 (s, 1H), 8.02 (d, *J* = 8.65 Hz, 2H), 7.46 (d, *J* = 8.7 Hz, 2H), 2.62 (s, 3H), 2.0 (s, 3H), 2.51 (s, 3H). ^{13}**C NMR** (150 MHz, $CDCl_3$) δ 198.37, 196.96, 196.52, 145.18, 134.23, 134.07, 130.28, 115.72, 31.74, 26.61, 26.46.

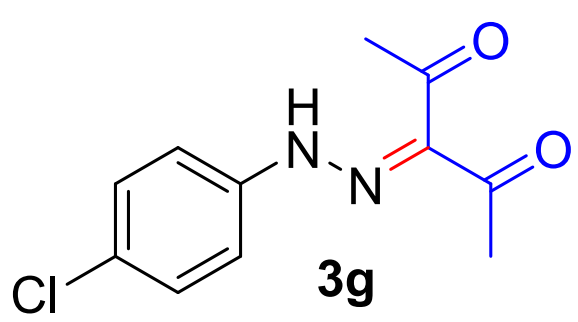
3g

3-(2-(4-Chlorophenyl)hyrazono)pentane-2,4-dione (3g)[2]: Prepared by reacting *p*-chlorobenzenediazonium tetrafluoroborate salt (**1g**, 0.5 mmol, 113.2 mg) and acetylacetone (**2a**, 0.5 mmol, 51.4 µL) following the above general procedure. Purification by column chromatography on silica gel with *n*-hexane/EtOAc: 50/1→20/1 yielded **3g** (106.21 mg, 0.45 mmol, 89%) as a curcumin-yellow solid.

^{1}**H NMR** (500 MHz, $CDCl_3$) δ 14.62 (s, 1H), 7.31-7.26 (m, 4H), 2.53 (s, 3H), 2.41 (s, 3H). ^{13} **C NMR** (150 MHz, $CDCl_3$) δ 198.15, 196.89, 140.17, 133.48, 130.99, 129.76, 117.32, 31.64, 26.57.

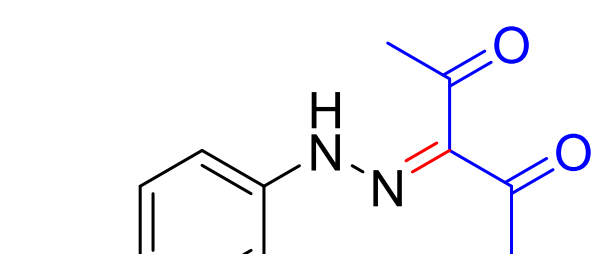
3h

3-(2-(4-(trifluoromethyl)phenyl)hydrazono)pentane-2,4-dione (3h)[2] : Prepared by reacting 4-(trifluoromethyl)benzenediazonium tetrafluoroborate salt (**1h**, 0.5 mmol, 130 mg) and acetylacetone (**2a**, 0.5 mmol, 51.4 µL) following the above general procedure. Purification by column chromatography on silica gel with *n*-hexane/EtOAc (50/1→10/1) yielded **3h** (107.5 mg, 0.394 mmol, 79%) as a light-yellow solid.

^{1}**H NMR** (500 MHz, $CDCl_3$) δ 14.58 (s, 1H), 7.66 (d, *J* = 8.35, 2H), 7.48 (d, *J* = 8.35, 2H), 2.62 (s, 3H), 2.51(s, 3H). ^{13}**C NMR** (150 MHz, $CDCl_3$) δ 198.48, 196.95, 144.32, 134.16, 129.73, 127.01, 126.98, 116.02, 31.78, 26.64.

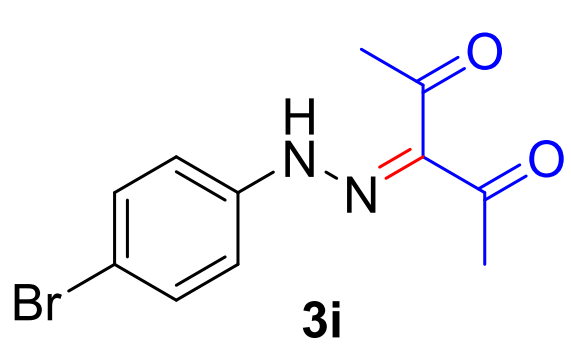
3i

3-(2-(4-bromophenyl)hydrazono)pentane-2,4-dione (3i)[2]: Prepared by reacting *p*-bromobenzenediazonium tetrafluoroborate salt (**1i**, 0.5 mmol, 135.4 mg) and acetylacetone (**2a**, 0.5 mmol, 51.4 µL) following the above general procedure. Purification by column chromatography on silica gel with *n*-hexane/EtOAc: 50/1→10/1 yielded **3i** (104.75 mg, 0.37 mmol, 74%) as a yellow solid.

^{1}**H NMR** (500 MHz, $CDCl_3$) δ 14.68 (s, 1H), 7.53-7.51 (m, 2H), 7.30-7.28 (m, 2H), 2.60 (s, 3H), 2.48 (s, 3H). ^{13}**C NMR** (125 MHz, $CDCl_3$) δ 198.35, 197.11, 140.74, 133.59, 132.81, 118.78, 117.79, 31.87, 26.78.

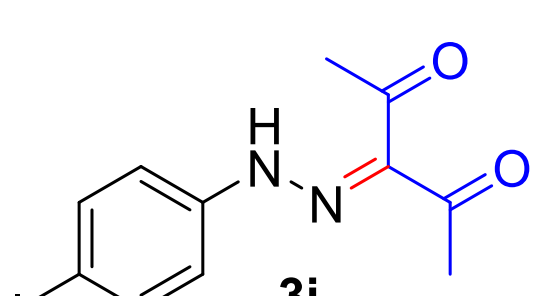
3j

3-(2-(4-iodophenyl)hydrazono)pentane-2,4-dione (3j)[6]: Prepared by reacting *p*-iodobenzenediazonium tetrafluoroborate salt (**1j**, 0.5 mmol, 159 mg) and acetylacetone (**2a**, 0.5 mmol, 51.4 µL) following the above

general procedure. Purification by column chromatography on silica gel with *n*-hexane/EtOAc: 50/1→20/1 yielded **3j** (115.55 mg, 0.35 mmol, 70%) as a yellow solid.

^{1}H NMR (500 MHz, $CDCl_3$) δ 14.63(s, 1H), 7.70 (d, *J* = 8.15 Hz, 2H), 7.16 (d, *J* = 8.2 Hz, 2H), 2.59(s, 3H), 2.46(s, 3H). **^{13}C NMR** (125 MHz, $CDCl_3$) δ 198.21, 196.91, 141.32, 138.58, 133.54, 117.94, 89.27, 31.69, 26.60.

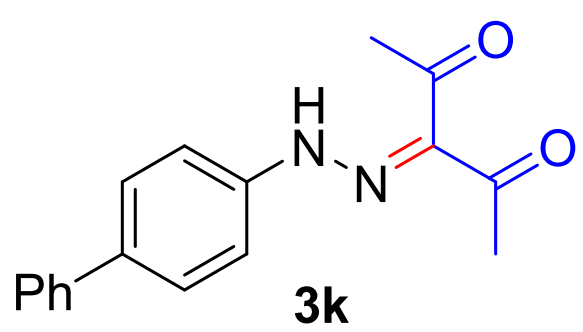

3k

3-(2-([1, 1-biphenylacetylphenyl]-4-yl)hydrazono)pentane-2,4-dione (3k): Prepared by reacting [1,1-biphenyl]-4-benzenedaizonium tetrafluoroborate salt (**1k**, 0.5 mmol, 134 mg) and acetylacetone (**2a**, 0.5 mmol, 51.4 µL) following the above general procedure. Purification by column chromatography on silica gel with *n*-hexane/EtOAc: 50/1→20/1 yielded **3k** (100.92 mg, 0.36 mmol, 72%) as a yellow solid.

^{1}H NMR (500 MHz, $CDCl_3$) δ 14.81 (s, 1H), 7.64 (d, *J* = 8.15 Hz, 2H), 7.59 (d, *J* = 7.6 Hz, 2H), 7.48-7.43 (m, 4H), 7.36 (t, *j* = 7.4 Hz, 1H), 2.61(s, 3H), 2.51(s, 3H). **^{13}C NMR** (125 MHz, $CDCl_3$) δ 198.10, 197.18, 140.89, 140.15, 138.97, 133.47, 129.04, 128.41, 127.63, 126.94, 116.78, 31.81, 26.79. **HRMS (ESI)** calculated for $C_{17}H_{17}N_2O_2^+$([M+H]$^+$): 281.1285, found: 281.1352.

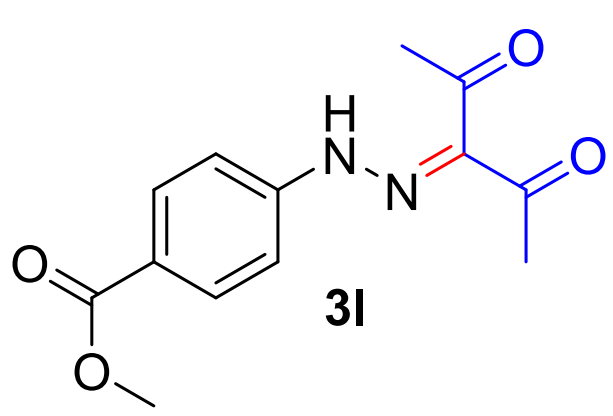

3l

Methyl 4-(2-(2,4-dioxopentan-3-ylidene)hydrazinyl)benzoate (3l): Prepared by reacting *p*-methylbenzoatediazonium tetrafluoroborate salt (**1l**, 0.5 mmol, 125 mg) and acetylacetone (**2a**, 0.5 mmol, 51.4 µl) following the above general procedure. Purification by column chromatography on silica gel with *n*-hexane/EtOAc: 50/1→20/1 yielded **3l** (90.48 mg, 0.35 mmol, 69%) as a yellow solid.

^{1}H NMR (500 MHz, $CDCl_3$) δ 14.58 (s, 1H), 8.09 (dd, *J* = 6.8, 1.95 Hz, 2H), 7.44-7.42 (m, 2H), 3.92 (s, 3H), 2.61 (s, 3H), 2.51 (s, 3H). **^{13}C NMR** (125 MHz, $CDCl_3$) δ 198.35, 196.98, 166.29, 145.11, 134.12, 131.39, 126.98, 115.61, 52.15, 31.74, 26.63. **HRMS (ESI)** calculated for $C_{13}H_{13}N_2O_4^+$([M-H]$^+$): 261.0881, found: 261.0880.

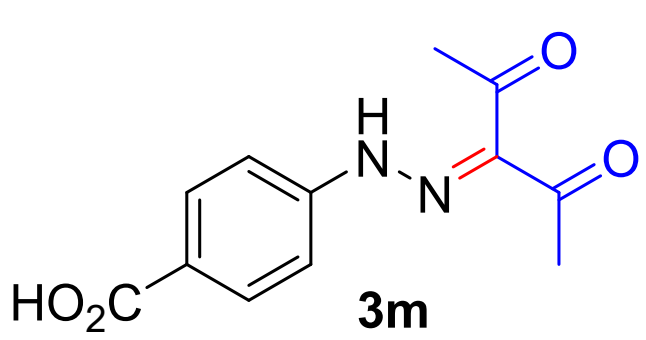

3m

4-(2-(2,4-dioxopentan-3-ylidene)hydrazinyl)benzoic acid (3m)[7]: Prepared by reacting *p*-carboxybenzenediazonium tetrafluoroborate salt (**1m**, 0.5 mmol, 118 mg) and acetylacetone (**2a**, 0.5 mmol, 51.4 µL) following the above general procedure. Purification by column chromatography on silica gel with *n*-hexane/EtOAc: 50/1→10/1 yielded **3m** (53.4 mg, 0.22 mmol, 43%) as a yellow solid.

^{1}H NMR (500 MHz, $CDCl_3$) δ 13.68 (s, 1H), 12.77 (s, 1H), 7.97-7.96 (m, 2H), 7.63-7.61 (m, 2H), 2.48 (s, 3H), 2.42 (s, 3H). **^{13}C NMR** (125 MHz, $CDCl_3$) δ 197.34, 196.40, 166.76, 145.45, 134.87, 130.96, 126.71, 115.73, 39.92, 39.78, 39.64, 39.50, 39.36, 39.22, 39.08, 31.18, 26.26.

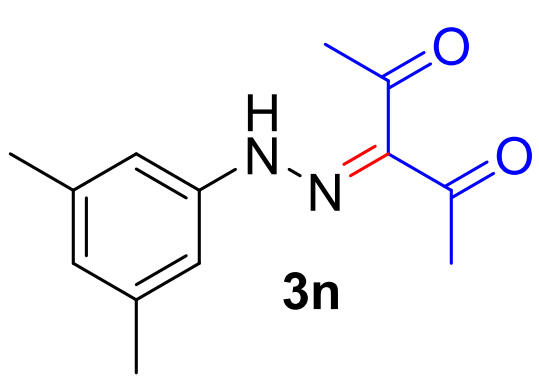

3n

3-(2-(3,5-Dimethylphenyl)hydrazono)pentane-2,4-dione (3n)[8]: Prepared by reacting 3,5-dimethylbenzenediazonium tetrafluoroborate salt **(1n**, 0.5 mmol, 110 mg) and acetylacetone **(2a**, 0.5 mmol, 51.4 µL) following the above general procedure. Purification by column chromatography on silica gel with *n*-hexane/EtOAc: 50/1→20/1 yielded **3n** (111.41 mg, 0.48 mmol, 96%) as a yellow solid.

^{1}**H NMR** (500 MHz, $CDCl_3$) δ 14.70 (s, 1H), 7.02 (s, 2H), 6.84 (s, 1H), 2.59 (s, 3H), 2.49 (s, 3H), 2.35 (s, 6H). ^{13} **CNMR** (125 MHz, $CDCl_3$) δ 197.74, 197.14, 141.46, 139.53, 127.82, 114.10, 31.62, 26.66, 21.38.

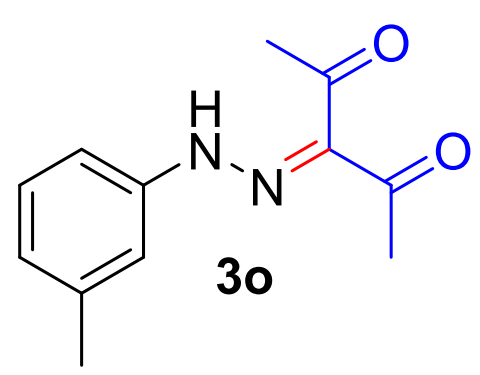

3o

3-(2-(m-tosyl))hydrazono)pentane-2,4-dione (3o)[8]: Prepared by reacting *m*-methylbenzenediazonium tetrafluoroborate salt (**1o**, 0.5 mmol, 103 mg) and acetylacetone (**2a**, 0.5 mmol, 51.4 µL) following the above general procedure. Purification by column chromatography on silica gel with *n*-hexane/EtOAc: 50/1→20/1 yielded **3o** (101.49 mg, 0.465 mmol, 93%) as an orange-yellow solid.

^{1}**H NMR** (600 MHz, $CDCl_3$) δ 14.73 (s, 1H), 7.30-7.27 (m, 1H), 7.22 (d, *j* = 6.3 Hz, 2H), 7.02 (d, *j* = 8.9 Hz, 1H) , 2.60 (s, 3H), 2.49 (s, 3H), 2.39 (s, 3H). ^{13}**C NMR** (150 MHz, $CDCl_3$) δ 197.82, 197.14, 141.43, 139.72, 133.03, 129.45, 126.79, 116.97, 113.45, 31.66, 26.64, 21.50.

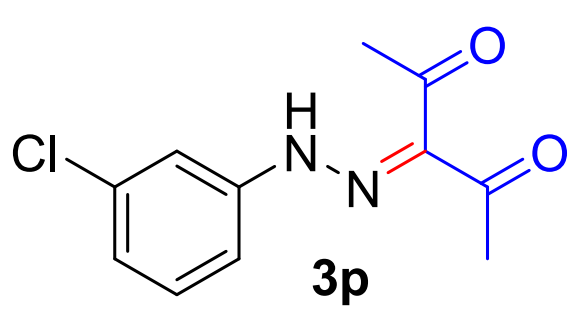

3p

3-(2-(3-chlorophenyl)hydrazono)pentane-2-3-dione (3p)[9]: Prepared by reacting *m*-chlorobenzenediazonium tetrafluoroborate salt (**1p**, 0.5 mmol, 113.2 mg) and acetylacetone (**2a**, 0.5 mmol, 51.4 µL) following the above general procedure. Purification by column chromatography on silica gel with *n*-hexane/EtOAc: 50/1→10/1 yielded **3p** (96.66 mg, 0.41 mmol, 81%) as yellow orange solid.

^{1}**H NMR** (600 MHz, $CDCl_3$) δ 14.59, (s, 1H), 7.28-7.19 (m, 4H), 2.51 (s, 3H), 2.39 (s, 3H). ^{13}**C NMR** (150 MHz, $CDCl_3$) δ 198.03, 196.79, 140.09, 133.36, 130.88, 129.66, 117.23, 31.57, 26.49.

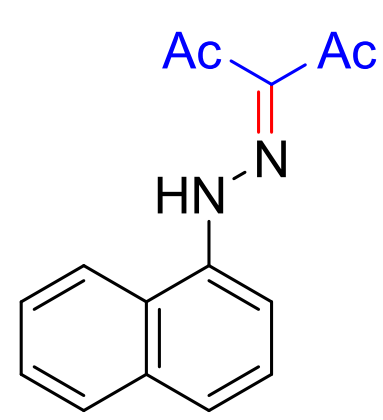

3q

3-(1-(naphthalene-1-yl)hydrazono)pentane-2,4-dione(3q)[10]: Prepared by reacting 1-napthylbenzenediazonium tetrafluoroborate salt (**1q**, 0.5 mmol, 121 mg) and acetyl acetonate (**2a**, 0.5 mmol, 51.4 µL) following general procedure. Purification by column chromatography on silica gel with *n*-hexane/EtOAc: 50/1→20/1 yielded **3q** (0.35 mmol, 87.73 mg, 69%) as light brown solid.

^{1}**H NMR** (600 MHz, $CDCl_3$) δ 15.61 (s, 1H), 7.92 (d, *J* = 8.46 Hz, 1H), 7.80 (t, *j* = Hz, 2H), 7.63 (d, *j* = 8.16 Hz, 1H), 7.53-7.43 (m, 3H), 2.59 (s, 3H), 2.45 (s, 3H). ^{13}**C NMR** (150 MHz, $CDCl_3$) δ 198.13, 197.13, 136.46, 134.26, 134.00, 128.77, 126.97, 126.52, 125.99, 125.95, 123.33, 119.50, 111.88, 31.66, 26.67. **LCMS (ESI)** calculated for $C_{15}H_{15}N_2O_2^+$([M+H]$^+$): 255.1128, found: 255.1162.

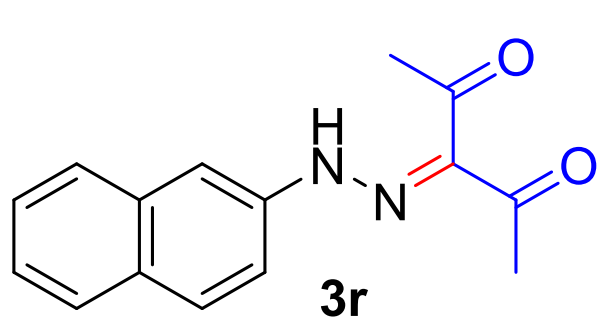

3r

3-(2-(naphthalene-2-yl)hydrazono)pentane-2,4-dione (3r)[11]: Prepared by reacting 2-napthylbenzenediazonium tetrafluoroborate salt (**1r**, 0.5 mmol, 121 mg) and acetylacetone (**2a**, 0.5 mmol, 51.3 µL) following the above general procedure. Purification by column chromatography on silica gel with *n*-hexane/EtOAc: 50/1→20/1 yielded **3r** (100.4 mg, 0.4 mmol, 79%) as a light brown solid.

^{1}**H NMR** (500 MHz, $CDCl_3$) δ 14.89 (s, 1H), 7.82 (d, *J* = 8.8 Hz, 1H), 7.76-7.74 (m, 2H), 7.64 (d, *J* = 2.2 Hz, 1H), 7.61 (dd, *J* = 8.8, 2.2 Hz, 1H), 7.45-7.42 (m, 1H), 7.39-7.36 (m, 1H), 2.56 (s, 3H), 2.48 (s, 3H). ^{13}**C NMR** (150 MHz, $CDCl_3$) δ 197.96, 197.07, 139.18, 133.70, 133.44, 131.71, 130.03, 127.99, 127.63, 127.22, 125.75, 125.73, 115.56, 115.54, 113.53, 31.65, 26.70. **LCMS (ESI)** calculated for $C_{15}H_{15}N_2O_2^+$([M+H]$^+$): 255.1128, found: 255.1134.

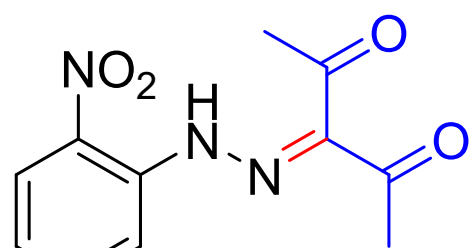
3s

3-(2-(2-nitrophenyl)hydrazono)pentane-2,4-dione (3s)[12]: Prepared by reacting 2-nitrobenzenediazonium tetrafluoroborate salt (**1s**, 0.5 mmol, 118.5 mg) and acetylacetone (**2a**, 0.5 mmol, 51.3 μL) following the above general procedure. Purification by column chromatography on silica gel with (*n*-hexane/EtOAc: 50/1→10/1) yielded **3s** (104.676 mg, 0.42 mmol, 84%) as a light yellow solid.

^{1}H NMR (600 MHz, $CDCl_3$) δ 15.49(s, 1H), 8.30 (dd, *J* = 8.46, 1.5 Hz, 1H), 8.16-8.14 (m, 1H), 7.75-7.72 (m, 1H), 7.29-7.26 (m, 1H), 2.66(s, 3H), 2.57(s, 3H), **^{13}C NMR** (150 MHz, $CDCl_3$) δ 197.30, 197.20, 138.55, 136.42, 135.95,135.90, 126.28, 124.15, 117.39, 31.84, 26.87.

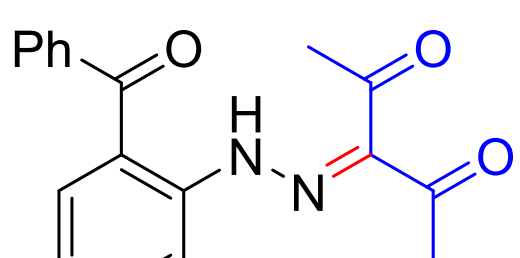
3t

3-(2-(2-benzoylphenyl)hydrazono)pentane-2,4-dione (3t): Prepared by reacting 2-benzoylbenzenediazonium tetrafluoroborate salt (**1t**, 0.5 mmol, 148 mg) and acetylacetone (**2t**, 0.5 mmol, 51.3 μL) following the above general procedure. Purification by column chromatography on silica gel with *n*-hexane/EtOAc: 50/1→20/1 yielded **3t** (0.37 mmol, 114.08 mg, 74%) light yellow solid.

^{1}H NMR (500 MHz, $CDCl_3$) δ 15.54 (s, 1H), 8.08 (dd, *J* = 8.4, 1.1 Hz, 1H), 7.79-7.77 (m, *J* = 4.56 Hz, 2H), 7.66-7.58 (m, 3H), 7.50-7.47 (m, 2H), 7.21-7.18 (m, 1H), 2.61 (s, 3H), 2.53 (s, 3H). **^{13} C NMR** (125 Hz, $CDCl_3$) δ 197.42, 197.40, 196.64, 143.58, 138.09, 134.86, 133.92, 133.30, 132.61, 130.13, 128.24, 123.47, 123.45, 116.35, 31.59, 26.72. **HRMS (ESI)** calculated for $C_{18}H_{17}N_2O_3^+$([M+H]$^+$): 309.1234, found: 309.1232.

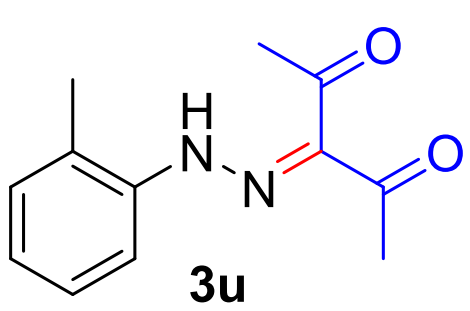
3u

3-(2-(o-tolyl)hydrazono)pentane-2,4-dione (3u)[13]: Prepared by reacting 2-methylbenzenediazonium tetrafluoroborate salt (**1u**, 0.5 mmol, 103 mg) and acetyl acetonate (**2a**, 0.5 mmol, 51.4 μL) following the above general procedure. Purification by column chromatography on silica gel with *n*-hexane/EtOAc: 50/1→20/1 yielded **3u** (85.12 mg, 0.39 mmol, 78%) as a yellow solid.

^{1}H NMR (500 MH z, $CDCl_3$) δ 14.98 (s, 1H), 7.76 (dd, *J* = 8.2, 1.2 Hz, 1H), 7.31-7.28 (m, 1H), 7.21 (d, *J* = 7.45 Hz, 1H), 7.13 (td, *j* = 7.5, 1.3 Hz, 1H), 2.63 (s, 3H), 2.51 (s, 3H), 2.40 (s, 3H). **^{13}C NMR** (125 MHz, $CDCl_3$) δ 197.84, 197.20, 139.73, 133.78, 131.06, 127.47, 125.66, 125.58, 115.98, 31.59, 26.67, 16.98.

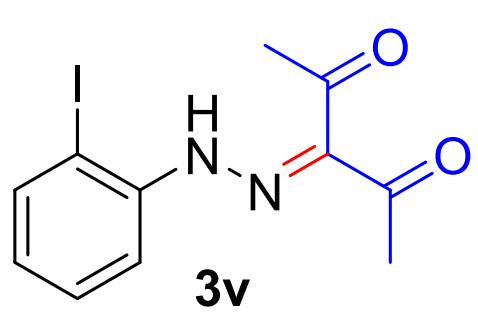
3v

3-(2-(2-iodophenyl)hydrazono)pentane-2,4-dione (3v): Prepared by reacting 2-iodobenzenediazonium tetrafluoroborate salt (**1v**, 0.5 mmol, 159 mg) and acetylacetone (**2a**, 0.5 mmol, 51.4 μL) following the above general procedure. Purification by column chromatography on silica gel with *n*-hexane/EtOAc: 50/1→20/1 yielded **3v** (0.23 mmol, 74.3 mg, 45%) as a yellow solid.

^{1}H NMR (500 MHz, $CDCl_3$) δ 14.76(s, 1H), 7.82 (d, *J* = 7.9 Hz, 1H), 7.73 (d, *J* = 8.3 Hz, 1H), 7.42 (t, *j* = 7.9 Hz, 1H), 6.93 (t, *j* = 7.75 Hz, 1H), 2.64 (s, 3H), 2.51 (s, 3H). **^{13}C NMR** (125 MHz, $CDCl_3$) δ 197.73, 197.20, 142.44, 139.68, 134.20, 129.65, 127.01, 116.84, 85.46, 31.75, 26.78. **MS (EI)**: m/z = 330.2 [M]$^+$, 297.1, 232.1, 219.1, 203.2, 149.1, 91.1, 64.0, 43.0.

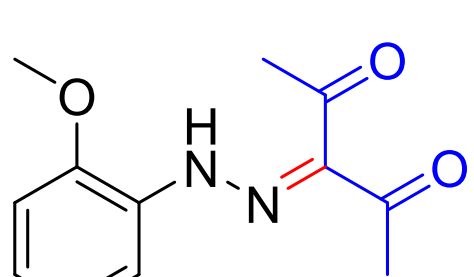
3w

3-(2-(2-methoxyphenyl)hydrazono)pentane-2,4-dione (3w)[14]: Prepared by reacting 2-methoxybenzenediazonium tetrafluoroborate salt (**1w**, 0.5 mmol, 111 mg) and acetylacetone (**2a**, 0.5 mmol, 51.4 μL) following the

above general procedure. Purification by flash column chromatography on silica gel with *n*-hexane/EtOAc: 50/1→20/1 yielded **3w** (99.56 mg, 0.43 mmol, 85%) as a yellow solid.

^{1}H NMR (500 MHz, $CDCl_3$) δ 14.80(s, 1H), 7.74 (dd, *J* = 8, 1.6 Hz, 1H), 7.16 (td, *j* = 8.1, 1.6 Hz, 1H), 7.05-7.02 (m, 1H), 6.95 (dd, *J* = 8.25,1.1 Hz, 1H), 3.96 (s, 3H), 2.61 (s, 3H), 2.50 (s, 3H). **^{13}C NMR** (125 MHz, $CDCl_3$) δ 197.25, 197.22, 148.56, 133.68, 130.82, 126.04, 121.45, 115.16, 111.09, 55.89, 31.59, 26.63.

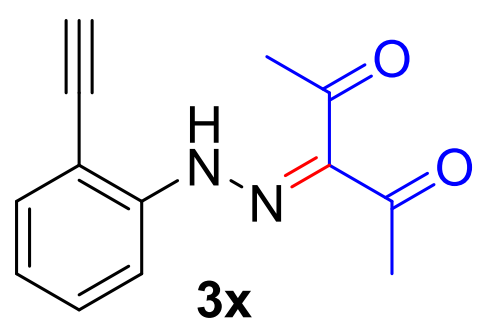

3x

3-(2-(2-ethynylphenyl)hydrazono)pentane-2,4-dione (3x): Prepared by reacting 2-ethanyl tetrafluoroborate benzenediazonium salt (**1x**, 0.5 mmol, 108 mg) and acetyl acetonate (**2a**, 0.5 mmol, 51 µL) following the above general procedure. Purification by column chromatography on silica gel with (*n*-hexane/EtOAc: 50/1→20/1) yielded **3x** (0.45 mmol, 101.571 mg, 89%) as a yellow solid.

^{1}H NMR (500 MHz, $CDCl_3$) δ 14.90 (s, 1H), 7.80-7.77 (m, 1H), 7.51-7.49 (m, 1H), 7.43(t, *J* =8.05 Hz, 1H), 7.14 (t, j = 7.7 Hz, 1H), 3.68 (s, 1H), 2.63(s, 3H), 2.51(s, 3H). **^{13}C NMR** (125 MHz, $CDCl_3$) δ 197.63, 197.30, 143.80, 143.05, 132.87, 130.51, 125.03, 114.65, 110.67, 85.62, 31.78, 26.81. **HRMS (ESI)** calculated for $C_{13}H_{13}N_2O_2^+$($[M+H]^+$): 229.0972, found: 229.0973.

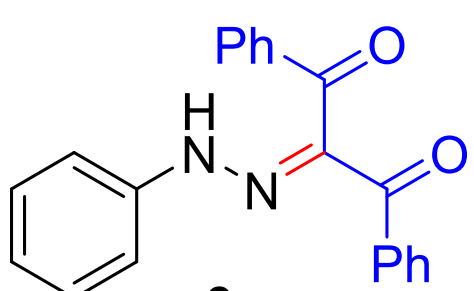

3y

1,3-diphenyl-2-(2-phenylhydrazono)propane-1,3-dione (3y)[15]: Prepared by reacting benzenediazonium tetrafluoroborate salt (**1a**, 0.5 mmol, 96 mg) and propane1,3-dione (**2b**, 0.5 mmol, 112.12 mg) following the above general procedure. Purification by column chromatography on silica gel with *n*-hexane/EtOAc: 50/1→20/1 yielded **3y** (100.1 mg, 0.31 mmol, 61%) as a yellow solid.

^{1}H NMR (500 MHz, $CDCl_3$) δ 13.57(s, 1H), 8.09-8.07 (m, 2H), 7.66-7.60 (m, 3H), 7.54-7.47 (m, 3H), 7.39-7.32 (m, 4H), 7.26 (d, *J* = 8.05 Hz, 2H), 7.13 (t, *J* = 7.35 Hz, 1H). **^{13}C NMR** (150 MHz, $CDCl_3$) δ 192.91, 191.50, 141.68, 138.19, 137.05, 133.81, 132.74, 132.54, 130.70, 129.65, 128.41, 128.34, 128.20, 125.15, 115.92.

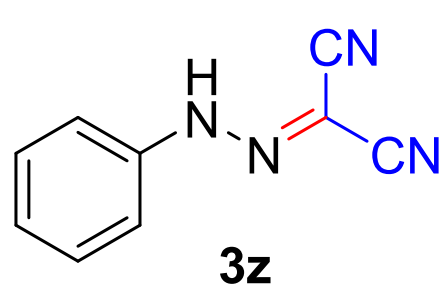

3z

***N*-phenylcarbonohydrazonyl dicyanide (3z)[16]:** Prepared by reacting benzenediazonium tetrafluoroborate salt (**1a**, 0.5 mmol, 96 mg) and malononitrile (**2c**, 0.5 mmol, 33.1 mg) following the above general procedure. Purification by column chromatography on silica gel with *n*-hexane/EtOAc: 50/1→10/1 yielded **3z** (80.7785 mg, 0.475 mmol, 95%) as a brown solid.

^{1}H NMR (500 MHz, $CDCl_3$) δ 9.73(s, 1H), 7.45-7.42 (m, 2H), 7.33-7.31 (m, 2H), 7.27 (d, *j* = 7.55 Hz, 1H). **^{13}C NMR** (150 MHz, $CDCl_3$) δ 139.63, 129.95, 126.83, 116.02, 112.12, 108.15, 86.77.

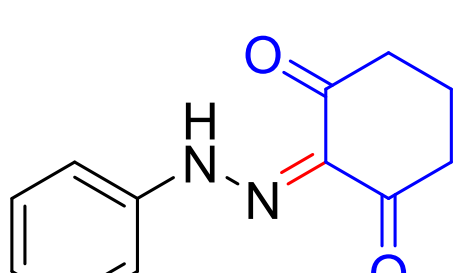

3aa

2-(2-phenylhydrazono)cyclohexane-1,3-dione (3aa)[17]: Prepared by reacting benzenediazonium tetrafluoroborate salt (**1a**, 0.5 mmol, 96 mg) and cyclohexane-1,3-dione (**2d,** 0.5 mmol, 56.1 mg) following the above general procedure. Purification by column chromatography on silica gel with *n*-hexane/EtOAc: 50/1→10/1 yielded **3aa** (63.3 mg, 0.29 mmol, 59%) as an orange-yellow solid.

^{1}H NMR (600 MHz, $CDCl_3$) δ 15.32 (s, 1H), 7.44-7.42 (m, 2H), 7.32-7.28 (m, 2H), 7.16-7.12 (m, 1H), 2.62-2.59 (s, 4H), 1.97 (p, *J* = 7.5, 2H). **^{13}C NMR** (150 MHz, CDCl3) δ 197.36, 193.71, 140.74, 131.05, 129.39, 127.00, 117.28, 38.64, 18.37.

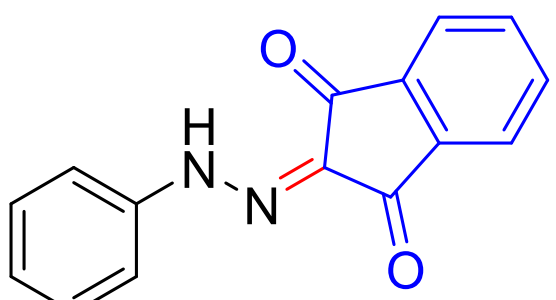

3ab

2-(2-(cyclohexa-1,5-diene-1-yl)hydrazono)naphthalene-1,3(2H, 4H)-dione (3ab)[18]: Prepared by reacting benzenediazonium tetrafluoroborate salt (**1a**, 0.5 mmol, 96 mg) and 1,3-indandione (**2e**, 0.5 mmol, 73.1 mg) following general procedure. Purification by column chromatography on silica gel with *n*-hexane/EtOAc: 50/1→10/1 yielded **3ab** (80.1 mg, 0.32 mmol, 64%) as an orange solid.

1H NMR (600 MHz, $CDCl_3$) δ 13.37 (s, 1H), 7.87-7.85 (m, 1H), 7.79-7.78 (m, 1H), 7.71-7.66 (m, 2H), 7.45-7.43 (m, 2H), 7.35-7.32 (m, 2H), 7.15-7.13 (m, 1H). **^{13}C NMR** (150 MHz, $CDCl_3$) δ 188.68, 186.14, 140.93, 140.52, 138.71, 135.25, 134.90, 130.65, 129.61, 126.23, 123.14, 122.79, 116.25.

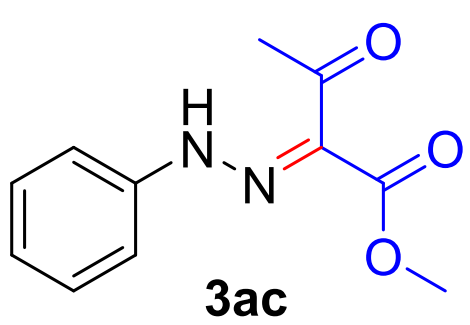

3ac

Methyl (*E*)-3-oxo-2-(2-phenylhydrazono)butanoate (3ac), *E:Z* (1.6:1): Prepared by reacting benzenediazonium tetrafluoroborate salt (**1a**, 0.5 mmol, 96 mg) and methyl 3-oxobutanoate (**2f**, 0.5 mmol, 54 µL) following the above general procedure. Purification by column chromatography on silica gel with (*n*-hexane/EtOAc: 50/1→20/1) yielded **3ac** (107.912 mg, 0.49 mmol, 98%, mixture of *E* and *Z* in 1.6:1) as a gummy brown paste.

^{1}H NMR (600 MHz, $CDCl_3$) δ 14.76 (s, 1H), 12.75 (s, 1H), 7.34-7.25 (m, 7H), 7.18-7.06 (m, 2H), 6.79-6.78 ((m, 1H), 3.81-3.78 (s, 3H), 2.51-2.41 (s, 3H). **^{13}C NMR** (150 MHz, $CDCl_3$) δ 196.95, 194.61, 165.36, 164.18, 141.45, 129.45, 129.46, 129.42, 126.49, 125.81, 125.52, 125.08, 120.00, 116.42, 115.56, 115.32, 52.99, 51.97, 30.69, 26.72. **HRMS (ESI)** calculated for $C_{11}H_{11}N_2O_3^+$([M-H]$^+$): 219.0775, found: 219.0764.

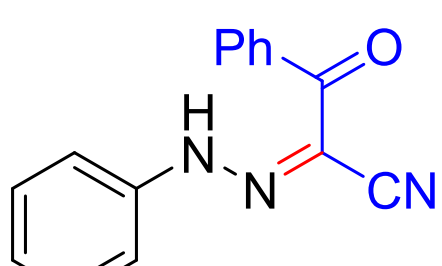

3ad

2-oxo-N, 2-diphenylacetohydrazonoyl cyanide (3ad)[19], *E:Z* (1:1.4): Prepared by reacting benzenediazonium tetrafluoroborate salt (**1a**, 0.5 mmol, 96 mg) and 2-benzoylacetonitrile (**2g**, 0.5 mmol, 72.58 mg) following general procedure. Purification by column chromatography on silica gel with *n*-hexane/EtOAc: 50/1→10/1 yielded **3ad** (99.71 mg, 0.4 mmol, 80%, mixture of *E* and *Z* in 1:1.4) as light yellow solid.

^{1}H NMR (600 MHz, $CDCl_3$) δ 15.10 (s, 1H), 9.59 (s, 1H), 7.93-7.89 (m, 3H), 7.54-7.51 (m, 2H), 7.45-7.30 (m, 9H), 7.21-7.17 (m, 2H), 7.12 (tt, *J* = 7.44, 1.2 Hz, 1H). **^{13}C NMR** (150 MHz, $CDCl_3$) δ 188.63, 186.13, 140.54, 140.50, 135.93, 135.66, 133.27, 132.80, 130.09, 129.82, 129.76, 128.60, 128.49, 128.18, 127.15, 125.72, 117.86, 116.77, 115.90, 115.09, 110.65, 110.40.

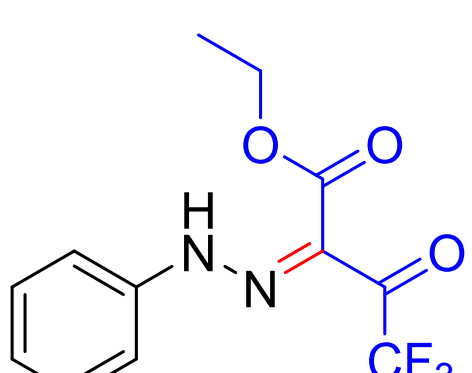

3ae

Ethyl-(*Z*)-4,4,4-trifluoro-3-oxo-2-(2-phenylhydrazono)butanoate (3ae)[20]: Prepared by reacting benzenediazonium tetrafluoroborate salt (**1a**, 0.5 mmol, 96 mg) and ethyl-4,4,4-triflouro-3-oxobutanoate (**2h**, 0.5 mmol, 73.12 µL) following the above general procedure. Purification by column chromatography on silica gel with *n*-hexane/EtOAc: 50/1→10/1 yielded **3ae** (86.5 mg, 0.3 mmol, 60%) as an orange solid.

^{1}H NMR (600 MH z, $CDCl_3$) δ 13.40(s, 1H), 7.36-7.33 (m, 4H), 7.19-7.16 (m, 1H), 4.33(q, *j* = 7.1 Hz, 2H), 1.34 (t, *j* = 7.14 Hz, 3H). **^{13} C NMR** (150 MHz, $CDCl_3$) δ 174.76, 174.50, 163.70, 140.79, 129.79, 126.68, 121.20, 118.27, 116.587, 115.94, 61.86, 61.81, 14.01.

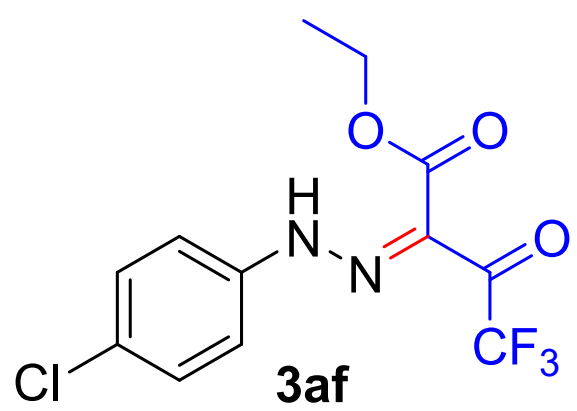

3af (exclusively *Z*)

Ethyl-(*Z*)-2(2-(4-chlorophenyl)hydrazono)-4,4,4-trifluoro-3-oxobutanoate (3af)[21]: Prepared by reacting *p*-chlorobenzenediazonium tetrafluoroborate salt (**1g**, 0.5 mmol, 113.2 mg) and ethyl-4,4,4-trifluoro-3-oxobutanoate (**2h**, 0.5 mmol, 73.12 µL) following the above general procedure. Purification by column chromatography on silica gel with *n*-hexane/EtOAc: 50/1→10/1 yielded **3af** (108.12 mg, 0.34 mmol, 67%) as an orange solid.

^{1}H NMR (600 MH z, $CDCl_3$) δ 13.35 (s, 1H), 7.32-7.30 (m, 2H), 7.27-7.25 (m, 2H), 4.32 (q, *J* = 7.14 Hz, 2H), 1.33 (t, *J* = 7.14, 3H). **^{13}C NMR** (150 MHz, $CDCl_3$) δ 174.82, 174.61, 174.39, 174.17, 163.58, 139.42, 131.95, 129.92, 121.64, 119.92, 117.98, 117.66, 117.60, 116.04, 114.10, 62.03, 13.97, 0.99.

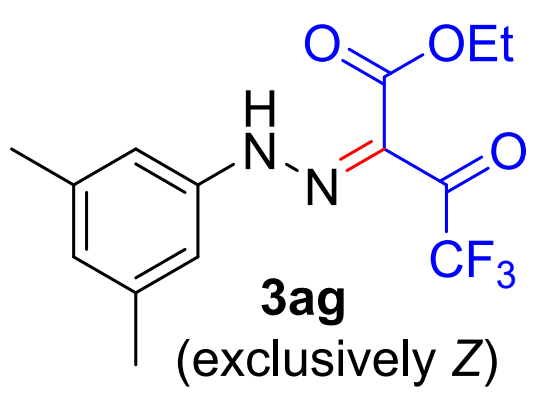

3ag (exclusively *Z*)

Ethyl (*Z*)-2-(2-3,5-dimethylphenyl)hydrazono)-4,4,4-trifluoro-3-oxobutanoate (3ag): Prepared by reacting 3,5-dimethylbenzenediazonium tetrafluoroborate salt (**1n**, 0.5 mmol, 110 mg) and ethyl-4,4,4-trifluoro-3-oxobutanoate (**2h**, 0.5 mmol, 73.12 µL) following the above general procedure. Purification by column chromatography on silica gel with *n*-hexane/EtOAc: 50/1→10/1 yielded **3ag** (107.54 mg, 0.34 mmol, 68%) as an orange solid.

^{1}H NMR (600 MH z, $CDCl_3$) δ 13.31 (s, 1H), 6.92-6.91 (m, 2H), 6.77 (s, 1H), 4.29 (t, *J* = 7.14 Hz, 2H), 2.24 (s, 6H), 1.31(t, *J* = 7.2 Hz, 3H). **^{13}C NMR** (150 MHz, $CDCl_3$) δ 174.85, 174.63, 174.42, 174.20, 163.71, 140.71, 139.68, 128.53, 120.82, 120.06, 118.12, 116.18, 114.36, 114.34, 114.30, 114.24, 61.67, 21.25, 13.95. **LCMS (ESI)** calculated for $C_{14}H_{16}F_3N_2O_3^+$([M+H]$^+$): 317.1108, found: 317.1127.

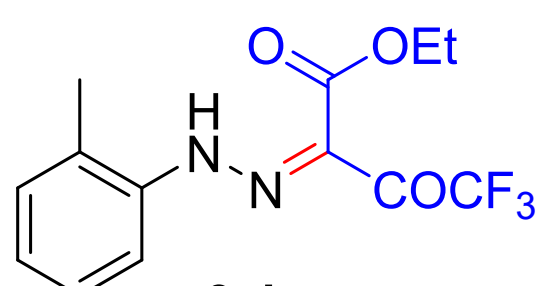

3ah

(exclusively Z)

Ethyl (*Z*)-4,4,4-trifluoro-3-oxo-2-(2-(o-tolyl)hydrazono)butanoate (3ah): Prepared by reacting 2-methylbenzenediazonium tetrafluoroborate salt (**1u**, 0.5 mmol, 103 mg) and ethyl-4,4,4-triflouro-3-oxobutanoate (**2h**, 0.5 mmol, 73.12 µL) following the above general procedure. Purification by column chromatography on silica gel with n-hexane/EtOAc: 50/1→10/1 yielded **3ah** (129 mg, 0.426 mmol, 85%) as an orange-yellow solid.

^{1}H NMR (600 MHz, $CDCl_3$) δ 13.65 (s, 1H), 7.62 (dd, *J* = 8.28, 1.2 Hz, 1H), 7.24-7.21 (m, 1H), 7.14-7.12 (m, 1H), 7.08-7.06 (tt, *J* = 7.5, 1.26 Hz, 1H), 7.34 (q, *J* = 7.14 Hz, 2H), 2.34 (s, 3H), 1.34 (d, *J* = 7.14, 3H). **^{13}C NMR** (150 MHz, $CDCl_3$) δ 175.02, 174.80, 174.58, 174.37, 164.04, 139.01, 131.05, 131.03, 127.83, 126.41, 125.67, 125.66, 121.70, 120.08, 118.14, 116.20, 115.58, 114.26, 61.86, 16.71, 14.04. **MS (EI)**: 302.2 ([M]$^+$), 233.2, 228.2, 119.1, 106.1, 91.0, 79.0, 65.0.

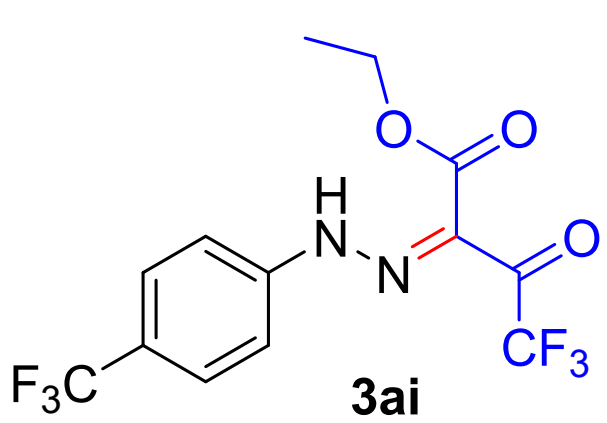

3ai

(Exclusively Z)

Ethyl(*Z*)-4,4,4-trofluoro-3-oxo-2-(2-(4-(trifluoromethyl)phenyl)hydrazono)butanoate (3ai): Prepared by reacting *p*-trifluoromethylbenzenediazonium tetrafluoroborate salt (**1h**, 0.5 mmol, 130 mg) and ethyl-4,4,4-trifluoro-3-oxobutanoate (**2h**, 0.5 mmol, 73.12 µL) following the above general procedure. Purification by column chromatography on silica gel with *n*-hexane/EtOAc: 50/1→10/1 yielded **3ai** (111.39 mg, 0.313 mmol, 61%) as a yellow solid.

^{1}H NMR (600 MHz, $CDCl_3$) δ 13.29 (s, 1H), 7.58 (d, *J* = 8.34, 2H), 7.39 (d, *J* = 8.4, 2H), 4.32 (q, *J* = 7.14, 2H), 1.31 (t, *J* = 7.08, 3H). **^{13}C NMR** (150 MHz, $CDCl_3$) δ 174.63, 174.37, 163.16, 143.39, 128.16, 127.90, 127.07, 127.03, 127.00, 126.97, 124.83, 122.67, 122.57, 118.02, 116.36, 115.67, 62.20, 13.82. **LCMS (ESI)** calculated for $C_{13}H_{10}F_6N_2O_3Na^+$([M+Na]$^+$): 379.0494, found: 379.3034.

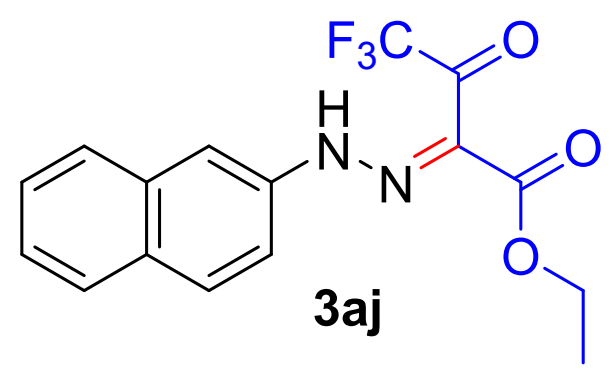

3aj

Ethyl (*Z*)-4,4,4-trifluoro-2-(2-(naphthalene-2-yl)hydrazono)-3-oxobutanoate (3aj): Prepared by reacting benzenediazonium tetrafluoroborate salt (**1r**, 0.5 mmol, 121 mg) and ethyl-4,4,4-triflouro-3-oxobutanoate (**2h**, 0.5 mmol, 73.12 µL) following the above general procedure. Purification by column chromatography on silica gel with *n*-hexane/EtOAc: 50/1→10/1 yielded **3aj** (104.1 mg, 0.31 mmol, 62%) as a yellow-orange solid.

^{1}H NMR (600 MHz, $CDCl_3$) δ 13.50 (s, 1H), 7.75 (d, *J* = 8.58 Hz, 3H), 7.68 (t, *J* = 7.86 Hz, 2H),7.53-7.51 (m, 2H), 7.39 (t, *J* = 7.68 Hz, 1H), 7.34 (t, *J* = 7.52 Hz, 1H), 4.30 (q, *J* = 7.14 Hz, 2H), 1.32 (t, *J* = 7.08 Hz, 3H). **^{13} C NMR** (150 MHz, $CDCl_3$) δ 174.79, 174.57, 174.36, 174.14, 163.68, 138.37, 133.37, 131.92, 130.22, 127.94, 127.72, 127.26, 126.11, 121.25, 118.13, 116.19, 115.26, 114.23, 61.82, 13.97. **LCMS (ESI)** calculated for $C_{16}H_{14}F_3N_2O_3^+$ ($[M+H]^+$): 339.0951, found: 339.1038.

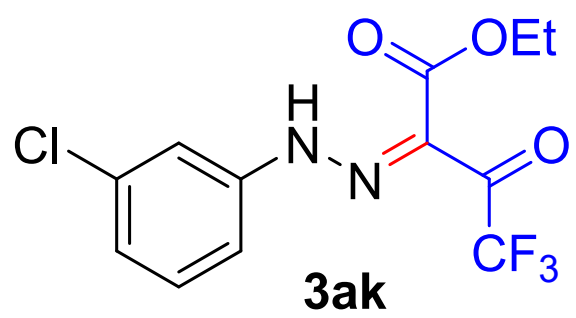

3ak
(exclusively *Z*)

Ethyl(*Z*)-2-(2-(3-chlorophenyl)hydrazono)-4,4,4-trifluoro-3-oxobutanoate(3ak): Prepared by reacting *m*-chlorobenzenediazonium tetrafluoroborate salt (**1p**, 0.5 mmol, 113.2 mg) and ethyl-4,4,4-triflouro-3-oxobutanoate (**2h**, 0.5 mmol, 73.12 µL) following the above general procedure. Purification by column chromatography on silica gel with *n*-hexane/EtOAc: 50/1→10/1 yielded **3ak** (71 mg, 0.22 mmol, 44%) as a yellow-orange solid.

^{1}H NMR (600 MHz, $CDCl_3$) δ 13.29 (s, 1H), 7.33 (t, *J* = 2.04, 1H), 7.27 (t, *J* = 8.04 Hz, 1H), 7.20 (dq, *J* = 8.22, 1.08 Hz, 1H), 7.13 (dq, *J* = 7.98, 1.02 Hz, 1H) 4.33 (q, *J* = 7.14 Hz, 2H), 1.34 (t, J = 7.14 Hz, 3H). **^{13}C NMR** (150 MHz, $CDCl_3$) δ 174.91, 174.69, 174.47, 174.25, 163.43, 141.93, 135.78, 130.83, 130.80, 126.47, 122.02, 117.91, 116.65, 115.97, 114.64, 62.11, 13.97. **LCMS (ESI)** calculated for $C_{12}H_{11}ClF_3N_2O_3^+$($[M+H]^+$): 323.0405, found: 323.0441.

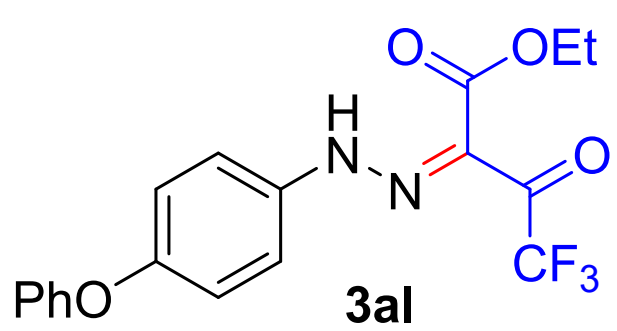

3al
(exclusively Z)

Ethyl (*Z*)-4,4,4-triflouro-3-oxo-2-(2(4-phenoxyphenyl)hydrazono) butanoate (3al): Prepared by reacting 4-phenoxybenzenediazonium tetrafluoroborate salt (**1d**, 0.5 mmol, 142 mg) and ethyl-4,4,4-trifluoro-3-oxobutanoate (**2h**, 0.5 mmol, 73.12 µL) following the above general procedure. Purification by column chromatography on silica gel with *n*-hexane/EtOAc: 50/1→10/1 yielded **3al** (129 mg, 0.34 mmol, 68%) as an orange-yellow solid.

^{1}H NMR (600 MHz, $CDCl_3$) δ 13.47 (s, 1H), 7.31-7.24 (m, 4H), 7.04 (tt, *J* = 7.38, 1.14 Hz, 1H), 6.97-6.91 (m, 4H), 4.30 (q, *J* = 7.08 Hz, 2H), 1.32(t, *J* = 7.14 Hz, 3H). **^{13}C NMR** (150 MHz, $CDCl_3$) δ 174.74, 174.52, 174.31, 174.09, 163.88, 156.62, 156.19, 136.25, 129.88, 129.87, 123.81, 119.74, 119.09, 118.12, 117.99, 116.18, 61.77, 13.99, 0.98. **MS (EI)**: m/z = 380.2 ($[M]^+$), 306.1, 183.0, 169.1, 141.0, 129.1, 77.0, 50.9.

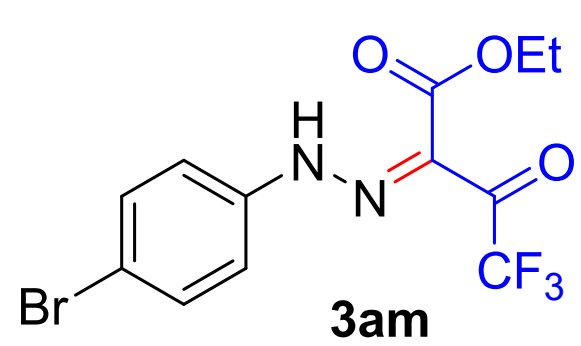

3am
(exclusively Z)

Ethyl (*Z*)-2-(2 (4-bromophenyl)hydrazono)-4,4,4-trifluoro-3-oxobutanoate (3am)[20]: Prepared by reacting reacting 4-bromobenzenediazonium tetrafluoroborate salt (**1i**, 0.5 mmol, 135.4 mg) and ethyl-4,4,4-triflouro-3-oxobutanoate (**2h**, 0.5 mmol, 73.12 µL) following the above general procedure. Purification by column chromatography on silica gel with *n*-hexane/EtOAc: 50/1→10/1 yielded **3am** (131.8 mg, 0.36 mmol, 72%) as a yellow-orange solid.

¹H NMR (600 MHz, $CDCl_3$) δ 13.30 (s, 1H), 7.44-7.42 (m, 2H), 7.19-7.17 (m, 2H), 4.30, (q, *J*=7.14, 2H), 1.31 (t, *J*=7.2, 3H). **¹³C NMR** (150 MHz, $CDCl_3$) δ 174.73, 174.51, 174.30, 174.08, 163.48, 139.84, 132.78, 131.65, 121.83, 121.66, 119.58, 117.98, 117.94, 117.90, 115.99, 61.99, 13.91, 0.95. **MS (EI):** m/z =366.1 ([M]⁺), 299.1, 292.0, 253.0, 185.0, 170.9, 154.9, 144.9, 91.0, 75.9, 62.9.

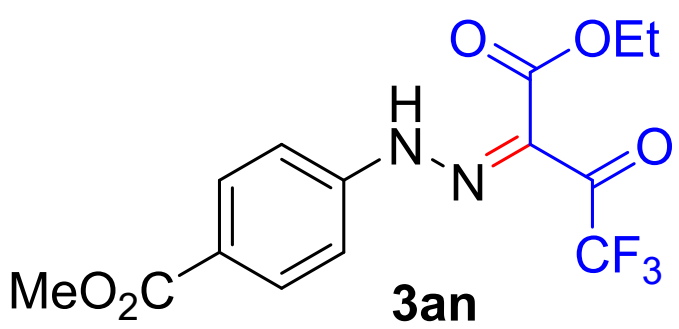

3an
(exclusively Z)

Methyl (*Z*)-4-(2-(1-ethoxy-4,4,4-trifluoro-1,3-dioxobutan-2-ylidene)hydrzinyl)benzoate (3an): Prepared by reacting *p*-methylbenzoatediazonium tetrafluoroborate salt (**1l**, 0.5 mmol, 125 mg) and ethyl-4,4,4-trifluoro-3-oxobutanoate (**2h**, 0.5 mmol, 73.12 µL) following the above general procedure. Purification by column chromatography on silica gel with *n*-hexane/EtOAc: 50/1→10/1 yielded **3an** (138.5 mg, 0.4 mmol, 80%) as a yellow-orange solid.

¹H NMR (600 MHz, $CDCl_3$) δ 13.34 (s, 1H), 8.05 (d, *J* = 8.7, 2H), 7.39 (d, *J* = 8.7, 2H), 3.89 (s, 2H), 3.86 (s, 3H), 1.18 (s, 3H). **¹³C NMR** (150 MHz, $CDCl_3$) δ 174.79, 174.57, 166.12, 163.71, 144.11, 131.53, 128.00, 122.30, 116.16, 116.08, 115.93, 52.62, 52.25, 29.68, 0.9956. **MS (EI)**: m/z = 346.2 ([M]⁺) 332.1, 301.1, 272.1, 263.1, 241.1, 149.0, 135.0, 122.0.

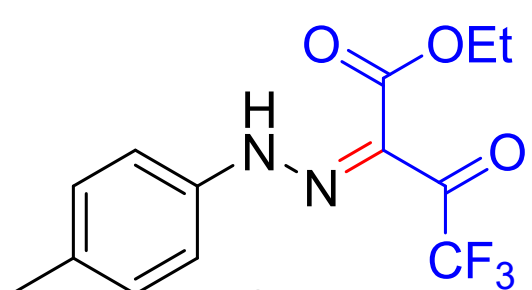

3ao
(exclusively Z)

Ethyl (*Z*)-4,4,4-triflouro-3-oxo-2-(2-(p-tolyl)hydrazono)butanoate (3ao)[20]**:** Prepared by reacting reacting 4-methylbenzenedaizonium tetrafluoroborate salt (**1c**, 0.5 mmol, 104 mg) and ethyl-4,4,4-triflouro-3-oxobutanoate (**2h**, 0.5 mmol, 73.12 µL) following the above general procedure. Purification by column chromatography on silica gel with *n*-hexane/EtOAc: 50/1→10/1) yielded **3ao** (111.78 mg, 0.37 mmol, 74%) as a yellow-orange solid.

¹H NMR (500 MHz, $CDCl_3$) δ 13.43 (s, 1H), 7.25-7.23 (m, 2H), 7.15 (d, *J* = 8.25, 2H), 4.32 (q, *J* = 7.15 Hz, 2H), 2.30 (s, 3H), 1.34 (t, *J* = 7.05 Hz, 3H). **¹³C NMR** (125 MHz, $CDCl_3$) δ 174.73, 174.46, 163.91, 138.59, 136.89, 130.35, 130.10, 120.73, 120.69, 118.36, 116.57, 113.70, 61.75, 21.03, 14.04.

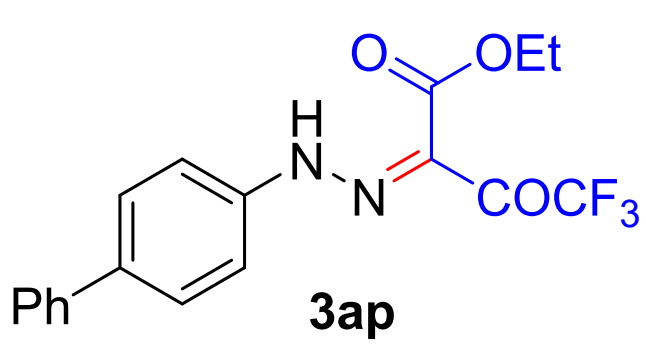

3ap
(exclusively Z)

Ethyl (*Z*)-2-(2-([1,1'-biphenyl]-4-yl)hydrazono)-4,4,4-trifluoro-3-oxobutanoate (3ap): Prepared by reacting [1, 1-biphenyl]-4 benzenediazonium tetrafluoroborate salt (**1k**, 0.5 mmol, 134 mg) and ethyl-4,4,4-triflouro-3-oxobutanoate (**2h**, 0.5 mmol, 73.12 µL) following the above general procedure. Purification by column chromatography on silica gel with *n*-hexane/EtOAc: 50/1→10/1 yielded **3ap** (124 mg, 0.34 mmol, 68%) as a yellow solid.

¹H NMR (600 MHz, $CDCl_3$) δ 13.44 (s, 1H), 7.56-7.53 (m, 2H), 7.48-7.46 (m, 2H), 7.38-7.33 (m, 4H), 7.27-7.24 (m, 1H), 4.30 (q, *J* = 7.2, 2H), 1.32 (t, *J* = 7.14, 3H). **¹³C NMR** (150 MHz, $CDCl_3$) δ 174.84, 174.63, 174.41, 174.19, 163.70, 139.97, 139.73, 139.58, 128.88, 128.33, 127.62, 126.79, 121.28, 118.11, 116.94, 116.17, 61.85, 13.97. **LCMS (ESI)** calculated for $C_{18}H_{16}F_3N_2O_3^+$([M+H]⁺): 365.1108, found: 365.1115.

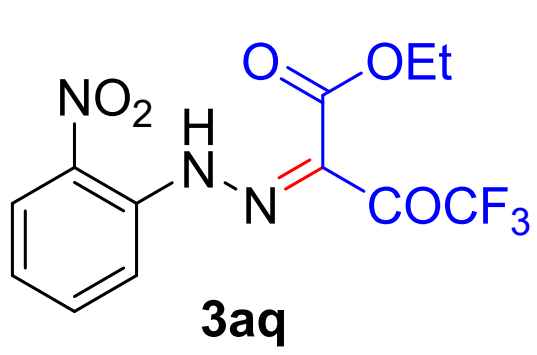

3aq
(exclusively *Z*)

Ethyl (*Z*)-4,4,4-trifluoro-2-(2-(2-nitrophenyl)hydrazono)-3 oxobutanoate (3aq)[20]: Prepared by reacting *o*-nitrobenzenediazonium tetrafluoroborate salt (**1s**, 0.5 mmol, 118.5 mg) and ethyl-4,4,4-triflouro-3-oxobutanoate (**2h**, 0.5 mmol, 73.12 µL) following the above general procedure. Purification by

column chromatography on silica gel with *n*-hexane/EtOAc: 50/1→10/1 yielded **3aq** (80 mg, 0.24 mmol, 48%) as an orange-yellow solid.

^{1}H NMR (500 MHz, $CDCl_3$) δ 14.00 (s, 1H), 8.14 (dd, *J* = 8.45, 1.55 Hz, 1H), 7.92 (dd, *J* = 8.9, 1.45 Hz, 1H), 7.54-7.50 (m, 1H), 6.97-6.93 (m, 1H), 4.29 (q, *J* = 7.1 Hz, 2H), 1.30 (t, *J* = 7.05 Hz, 3H). **^{13}C NMR** (125 MHz, $CDCl_3$) δ 162.17, 140.15, 135.77, 125.88, 124.93, 120.91, 116.12, 61.30, 14.16.

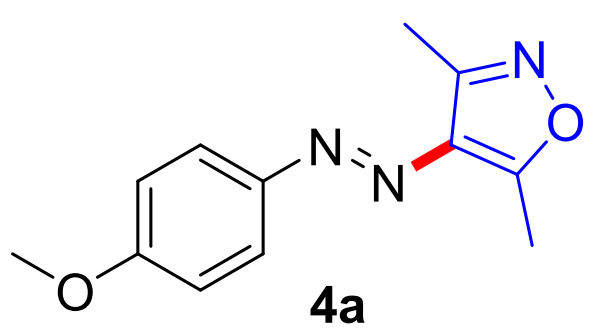

4a

(*E*)-4-((4-methoxyphenyl)diazenyl)-3,5-4H-1-isoxazole (4a)[22]: To a suspension of **3b** (1.0 equiv, 1mmol, 234.3 mg) in EtOH (10 ml) were added Na_2CO_3 (1.5 equiv, 1 mmol, 0.159 g), hydroxylamine hydrochloride (1.5 equiv, 0.1043 g), and the mixture was stirred at 80 ^{0}C for 18 h. The solvent was removed under reduce pressure and the crude product was purified by column chromatography with silica and n-hexane/EtOH (20:1) to afford 182.7 mg, 79% of **4a**.

^{1}H NMR (600 MHz, $CDCl_3$) δ 7.70 (d, *J* = 8.94, 2H), 6.89 (d, *J* = 8.94, 2H), 3.78 (s, 3H), 2.62 (s, 3H), 2.42 (s, 3H). **^{13}C NMR** (150 MHz, $CDCl_3$) δ 168.06, 161.74, 153.83, 147.20, 132.15, 123.87, 114.10, 55.49, 11.96, 11.55.

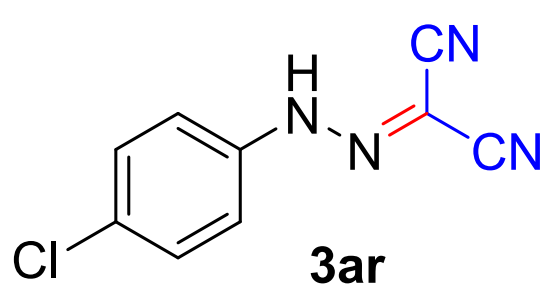

3ar

N-(4-chlorophenyl)carbonohyrazonoyl dicyanide (3ar)[23]: Prepared by reacting *p*-chlorobenzenediazonium tetrafluoroborate salt (**1g**, 0.5 mmol, 113.2 mg) and malononitrile (**2c**, 0.5 mmol, 33.1 g) following the above general procedure. Purification by column chromatography on silica gel with *n*-hexane/EtOAc: 50/1→10/1 yielded **3ar** (89 mg, 0.435 mmol, 87%) as a yellow solid.

^{1}H NMR (600 MHz, $CDCl_3$) δ 9.74 (d, *J* = 1.14 Hz, 1H), 7.31-7.30 (m, 1H), 7.29 (d, *J* = 8.1 Hz, 1H), 7.17-7.16 (dd, *J* = 8.28, 1.92 Hz, 1H), 7.12 (dd, *J* = 8.04, 2.16 Hz, 1H). **^{13}C NMR** (150 MHz, $CDCl_3$) δ 140.69, 136.05, 130.96, 126.73, 116.24, 114.13, 111.73, 107.92, 187.97.

4) Mechanistic Studies

4.1 Reaction without a catalyst

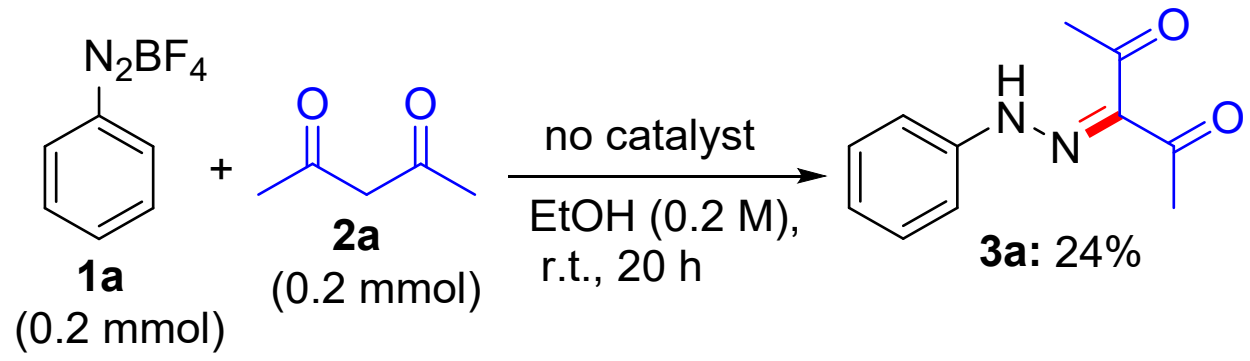

In a reaction tube, active methylene, **2a** (0.2 mmol, 21 μL), was added to arenediazonium tetrafluoroborate, **1a** (1.0 equiv, 0.2 mmol, 38.4 mg). Then, EtOH (1 mL) was further added, and the tube was closed with a rubber septum. The resulting mixture was stirred at room temperature for 20 hours under air. The reaction mixture was transferred to a round-bottom flask with ethyl acetate, then concentrated using a rotary evaporator. The residue was purified by column chromatography on silica gel using a mixture of n-hexane/EA to give 9.8 mg, 0.048 mmol (24%) of **3a.**

4.2 Radical scavenging experiment with TEMPO

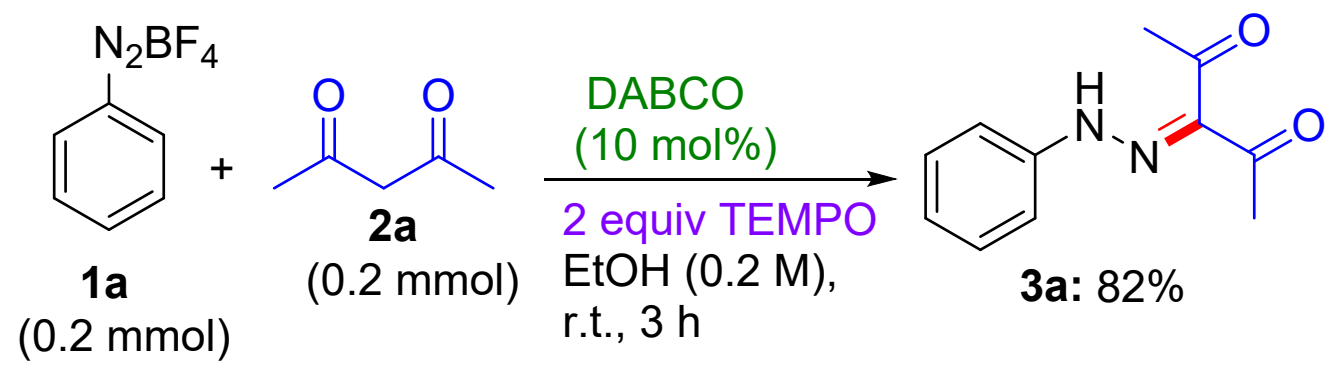

In a reaction tube, active methylene, **2a** (0.2 mmol, 21 μL), was added to arenediazonium tetrafluoroborate, **1a** (1.0 equiv, 0.2 mmol, 38.4 mg). Then, DABCO (10 mol%, 0.025 mmol, 2.8 mg), 2.0 equiv (0.4 mmol, 62.5 mg) of 2,2,6,6-Tetramethylpiperidine 1-oxyl (TEMPO), and EtOH (1 mL) were further added, and the tube was closed with a rubber septum. The resulting mixture was stirred at room temperature for 3 hours under air. The reaction mixture was transferred to a round-bottom flask with ethyl acetate, then concentrated using a rotary evaporator. The residue was purified by column chromatography on silica gel using a mixture of n-hexane/EA to give 33.5 mg, 0.164 mmol (82%) of **3a.**

5. X-ray Crystallographic data of 3ae.

CCDC	2538913
Identification code	3AC_S
Empirical formula	$C_{12}H_{11}F_3N_2O_3$
Formula weight	288.23
Temperature/K	100.00(10)
Crystal system	triclinic
Space group	P-1
a/Å	10.17090(10)
b/Å	11.45320(10)
c/Å	18.0859(2)
α/°	105.3650(10)
β/°	103.1580(10)
γ/°	96.8500(10)
Volume/Å^3	1941.66(4)
Z	6
ρ_{calc}g/cm^3	1.479
μ/mm^{-1}	1.186
F(000)	888.0
Crystal size/mm^3	0.14 × 0.13 × 0.12
Radiation	Cu Kα (λ = 1.54184)
2Θ range for data collection/°	5.264 to 150.012
Index ranges	$-12 \le h \le 12$, $-11 \le k \le 14$, $-22 \le l \le 22$
Reflections collected	22750

Independent reflections	7589 [R_{int} = 0.0165, R_{sigma} = 0.0144]
Data/restraints/parameters	7589/0/545
Goodness-of-fit on F^2	1.021
Final R indexes [I>=2σ (I)]	R_1 = 0.0304, wR_2 = 0.0827
Final R indexes [all data]	R_1 = 0.0322, wR_2 = 0.0840
Largest diff. peak/hole / e Å^{-3}	0.28/-0.28

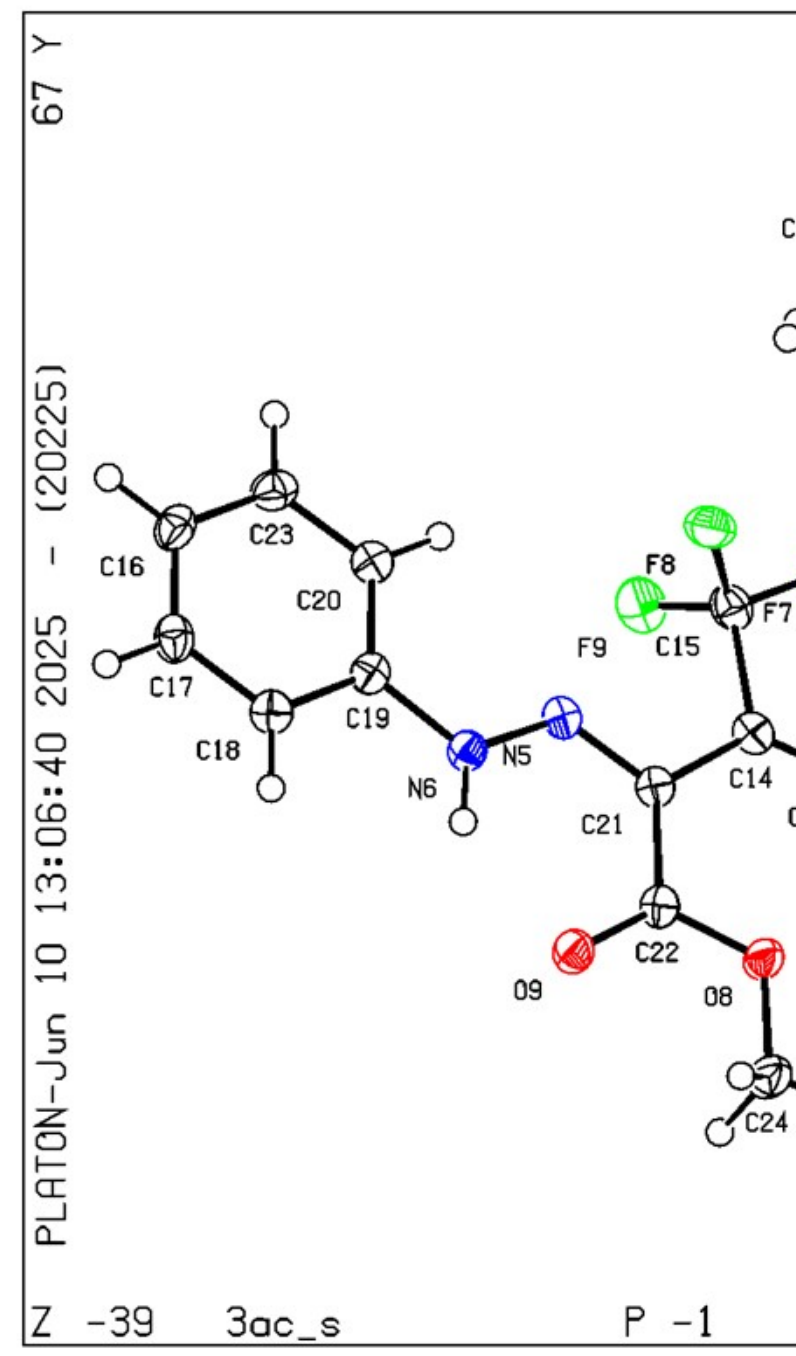

6. References:

1. a) H. P. Gemoets, I. Kalvet, A. V. Nyuchev, N. Erdmann, V. Hessel, F. Schoenebeck, T. Noël, *Chem, Sci*, 2017, **8,** 1046-1055; (b) Ramanathan, M. Liu, S. T. Preparation of Quinazolines *via* a 2+2+2 Annulation from Aryldiazonium Salts and Nitriles. *J. Org. Chem.* 2017, **82**, 8290−8295; (c) L. Wei, J. Zhang, L. Xu, *ACS Sustain. Chem. Eng.*, 2020, **8**, 13894-13899. (d) Z. Deng, Z. Liu, J. Han, T. –Y. Zhou, D. Cheng, X. Xiao, F. Chen, *Green Chem*. 2026, **28**, 5447-5456; (e) S.-L. Niu, W. Yuan, X. Gong, B. Bao, Z. -W. Wu, B. Xu, R. Zeng, Q. -W. Yang, Q. Ouyang, *ACS Sustainable Chem. Eng*. 2023, **11**, 17816-17825.

2. R. Tovtik, D. Marzin, P. Weigel, S. Crespi, N. A. Simeth, *Beilstein Arch***,** 2025*,* 20257.

3. B. A. Salah, A. T. Kandil, M. G. A. El-Nasser, *JRRAS*, 2019, **12**, 413-422.

4. S. Abdelal, A. Gheath, N. Alarafi, G. Hasim, *ASRJET*, 2017, **27,** 73-84.

5. T. Kamran Mahmudov, A. M. Maharramov, R. A. Aliyeva , I. A. Aliyev, R. K. Askerov, R. Batmaz, M. N. Kopylovich, A. J. L. Pombeiro, *J. Photochem. Photobiol. A: Chemistry,* 2011, **219**, 159-165.

6. C. E. Weston, A. Krämer, F. Colin, O. Yildiz, M. G. Baud, F. J. Meyer-Almes, M. J. Fuchter, *ACS Infect. Dis*, 2017, **3**, 152-161.
7. R.N. Sharma, K.P. Sharma, S.N. Dixit, *Orient. J. Chem.*, 2010, **26**, 283-286.
8. L. Stricker, M. Böckmann, T. M. Kirse, N. L. Doltsinis, B. J. Ravoo, *Chem. Eur. J*, 2018, **24**, 8639-8647.
9. A. Sethukumar, B. A. Prakasam, *J. Mol. Struct.* 2010, **963**, 250–257.
10. K. Krishnankutty, M. B. Ummathur, P. Ummer, *J. Serb. Chem. Soc.* 2009, **74**, 1273–1282.
11. B. C. Enache, A. Hanganu, C. Tablet, C. C. Anghel, C. C. Popescu, A. Paun, M. Matache, *ACS omega*, 2022, **7**, 39122-39135.
12. J. Marten, W. Seichter, E. Weber, U. Böhme, *J. Phys. Org. Chem.*, 2007, **20**, 716-731.
13. C. J. Patil, D. S. Talele, S. P. Talele, P. R. Pohekar, D. S. Kolhe, *J. Pharm. Sci. Res.* 2019, **11**, 2213-2219.
14. C. J. Patil, D. S. Talele, S. P. Talele, P. R. Pohekar, A. S. Patil, *Int. J. Pharm. Sci. Rev. Res*, 2017, **45**, 64-73.
15. N. Amri, A. N. M. Alaghaz, N. S. Bedowr, A. A. El-Bindary, *Appl. Organomet. Chem*, 2024, **38**, e7551.
16. H. C. Berk, J. E. Franz, *Synth Commun*, 1980, **10**, 189-193.
17. A Hossan, *Am. J. Org. Chem*, 2013, **3**, 9-15.
18. E. Evangelista, Y. P. V. de Carvalho, I. S. de Jesus, M. E. S. Rodrigues, E. P. Hayashi, K. N. de Andrade, R. G. Fiorot, L. da S. M. Forezi, *J. Org. Chem.* 2025, **90**, 300−308.
19. U. Fathy, A. A. Abu-Hashem, R. S. Gouhar, H. M. Awad, A. M. Elgamal, *Rasayan J. Chem*, 2021, **14**, 741-750.
20. G. F. Makhaeva, S. V. Lushchekina, N. P. Boltneva, O. G. Serebryakova, N. V. Kovaleva, ,E. V. Rudakova, R. J. Richardson, *Eur. J. of Med. Chem*, 2021, **218**, 113385.
21. S. Muralikrishna, P. Jagadeeswararao, P. Ravisankarareddy, HETEROCYCL LETT. 2017, **7**, 1097-1105.
22. L. Kortekaas, J. Simke, D. W. Kurka, B. J. Ravoo, *ACS Appl. Mater. Interfaces* 2020, ***12***, 32054–32060
23. H. A. Elbadawy, A. E. D. Ali, A. A. Elkashef, S. Foro, D. S El-Sayed, *Appl. Organomet. Chem*. 2022**, 36**, e6793.

7. NMR Spectra of products

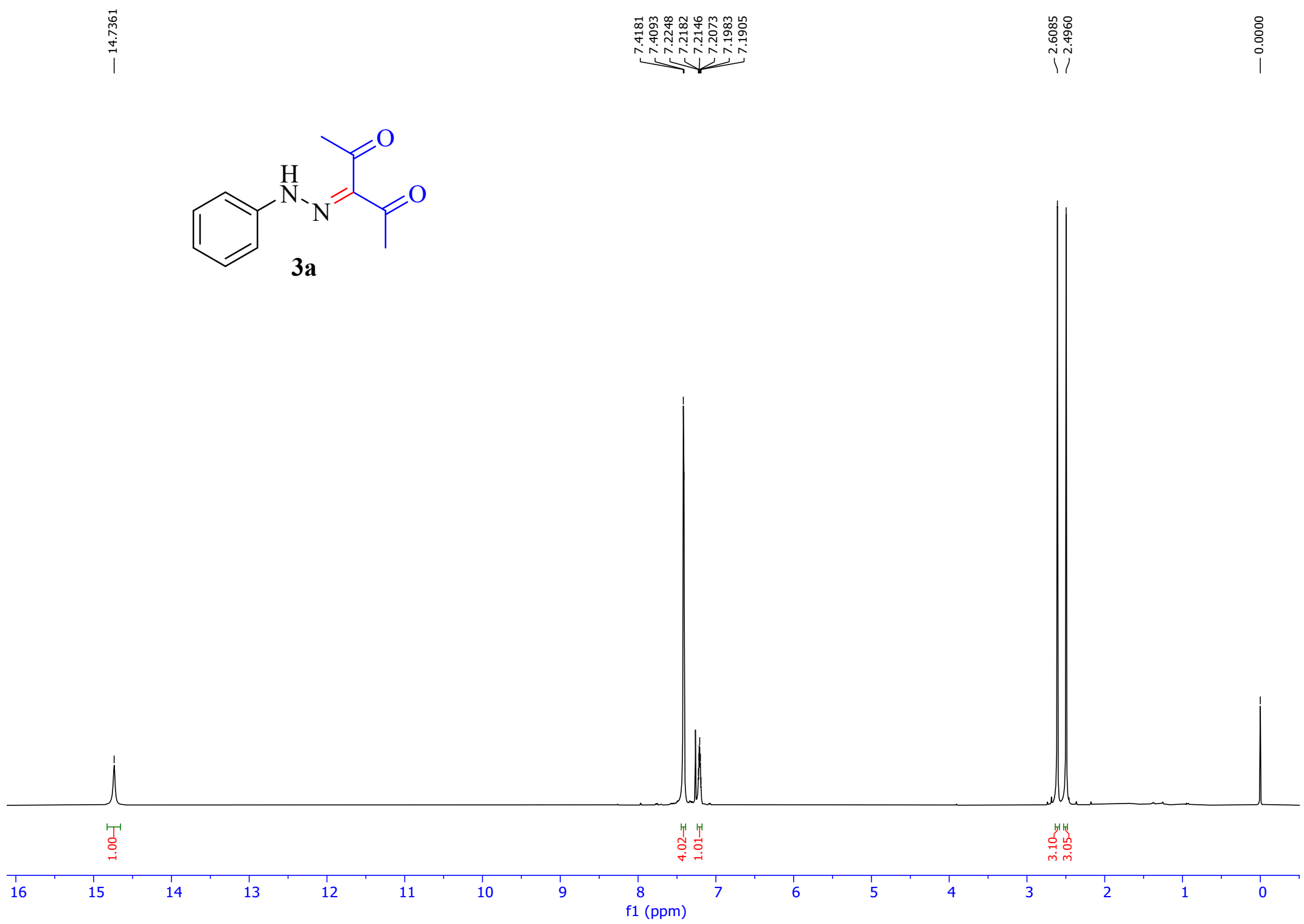
14.7361
7.4181
7.4093
7.2248
7.2182
7.2146
7.2073
7.1983
7.1905
2.6085
2.4960
0.0000
3a
1.00
4.02
1.01
3.10
3.05
f1 (ppm)

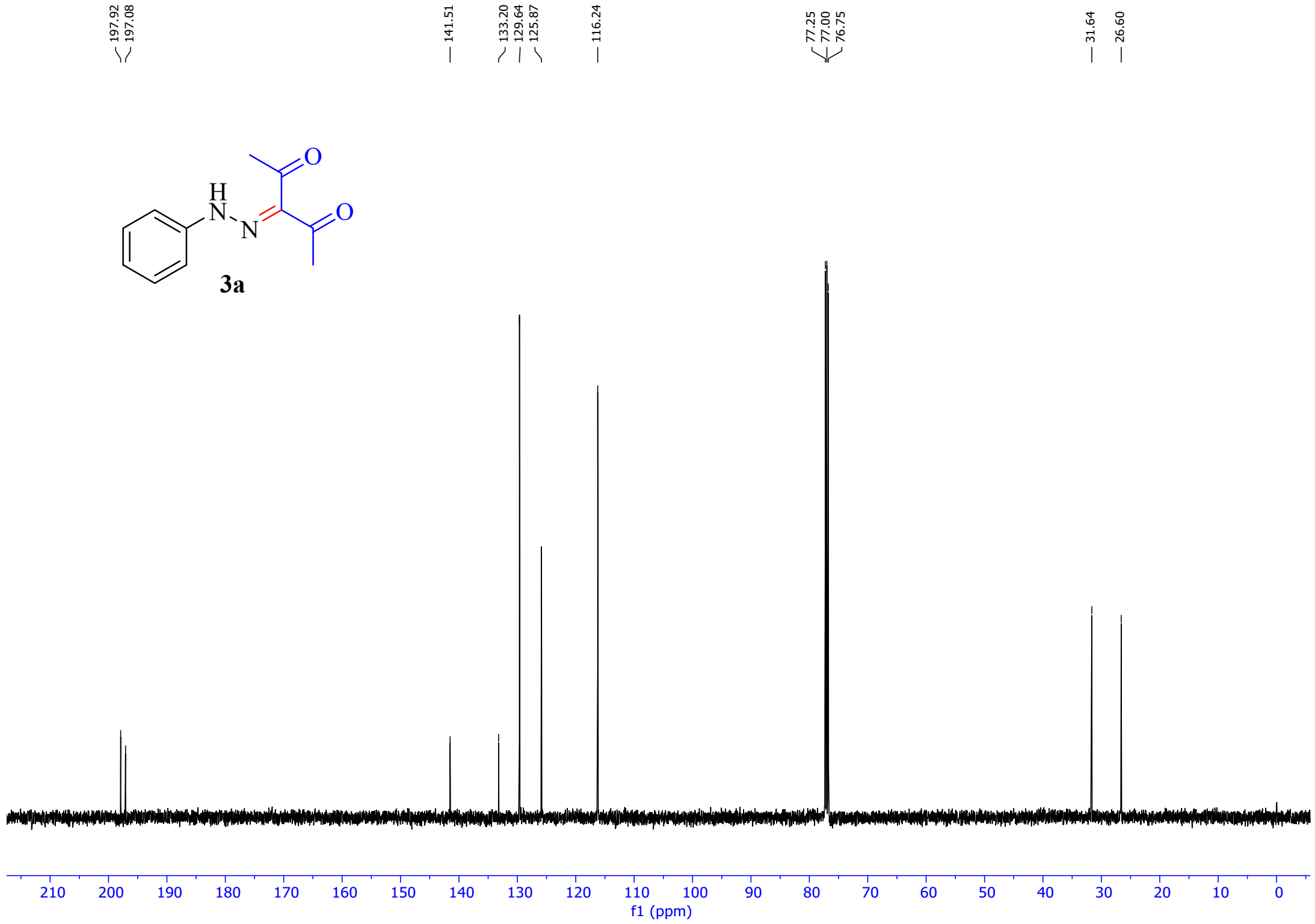
197.92
197.08
141.51
133.20
129.64
125.87
116.24
77.25
77.00
76.75
31.64
26.60
3a
f1 (ppm)

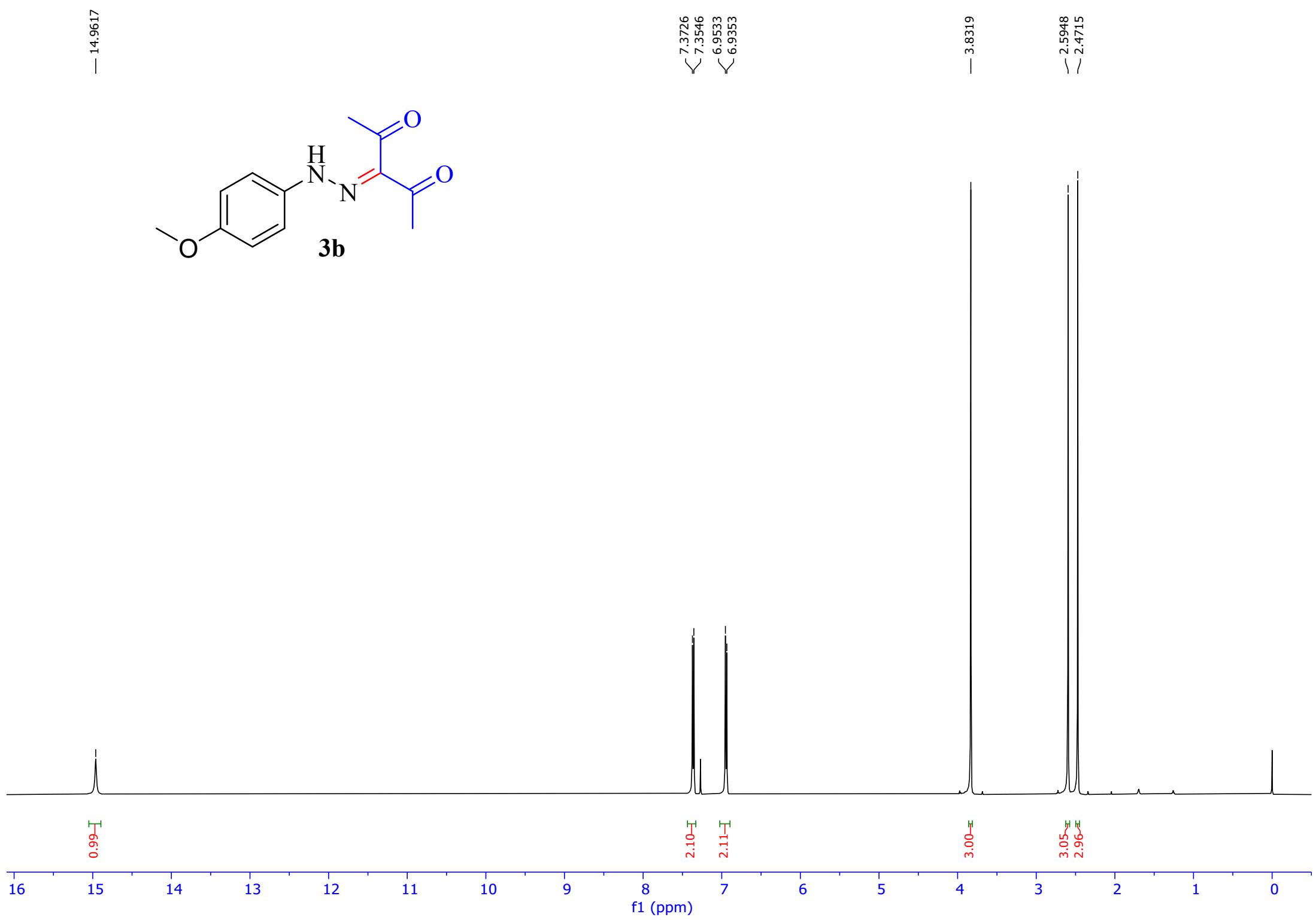

14.9617
7.3726
7.3546
6.9533
6.9353
3.8319
2.5948
2.4715
3b
0.99
2.10
2.11
3.00
3.05
2.96
16 15 14 13 12 11 10 9 8 7 6 5 4 3 2 1 0
f1 (ppm)

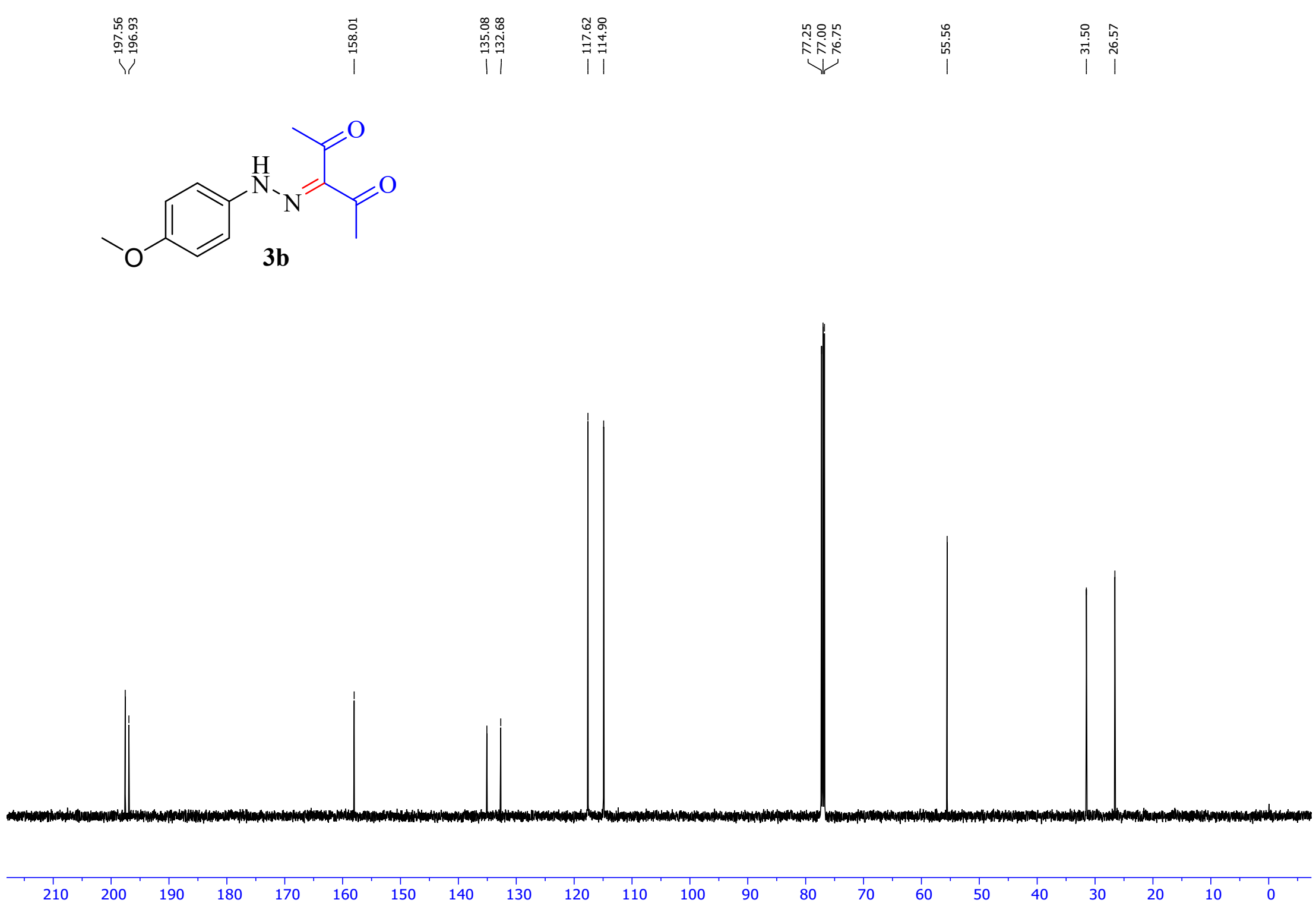

197.56
196.93
158.01
135.08
132.68
117.62
114.90
77.25
77.00
76.75
55.56
31.50
26.57
3b
210 200 190 180 170 160 150 140 130 120 110 100 90 80 70 60 50 40 30 20 10 0
f1 (ppm)

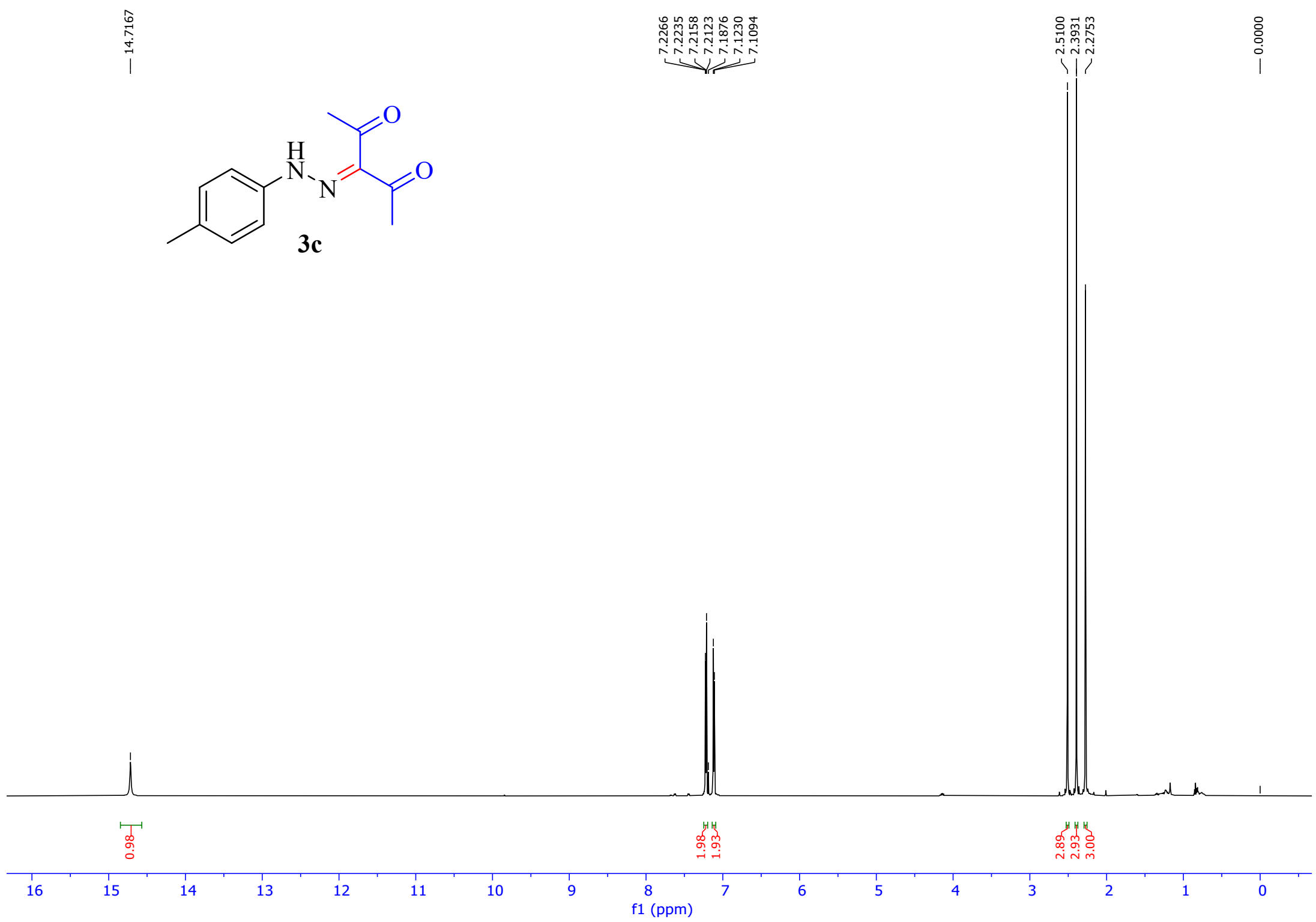
14.7167
7.2266
7.2235
7.2158
7.2123
7.1876
7.1230
7.1094
2.5100
2.3931
2.2753
0.0000
3c
0.98
1.98
1.93
2.89
2.93
3.00
f1 (ppm)

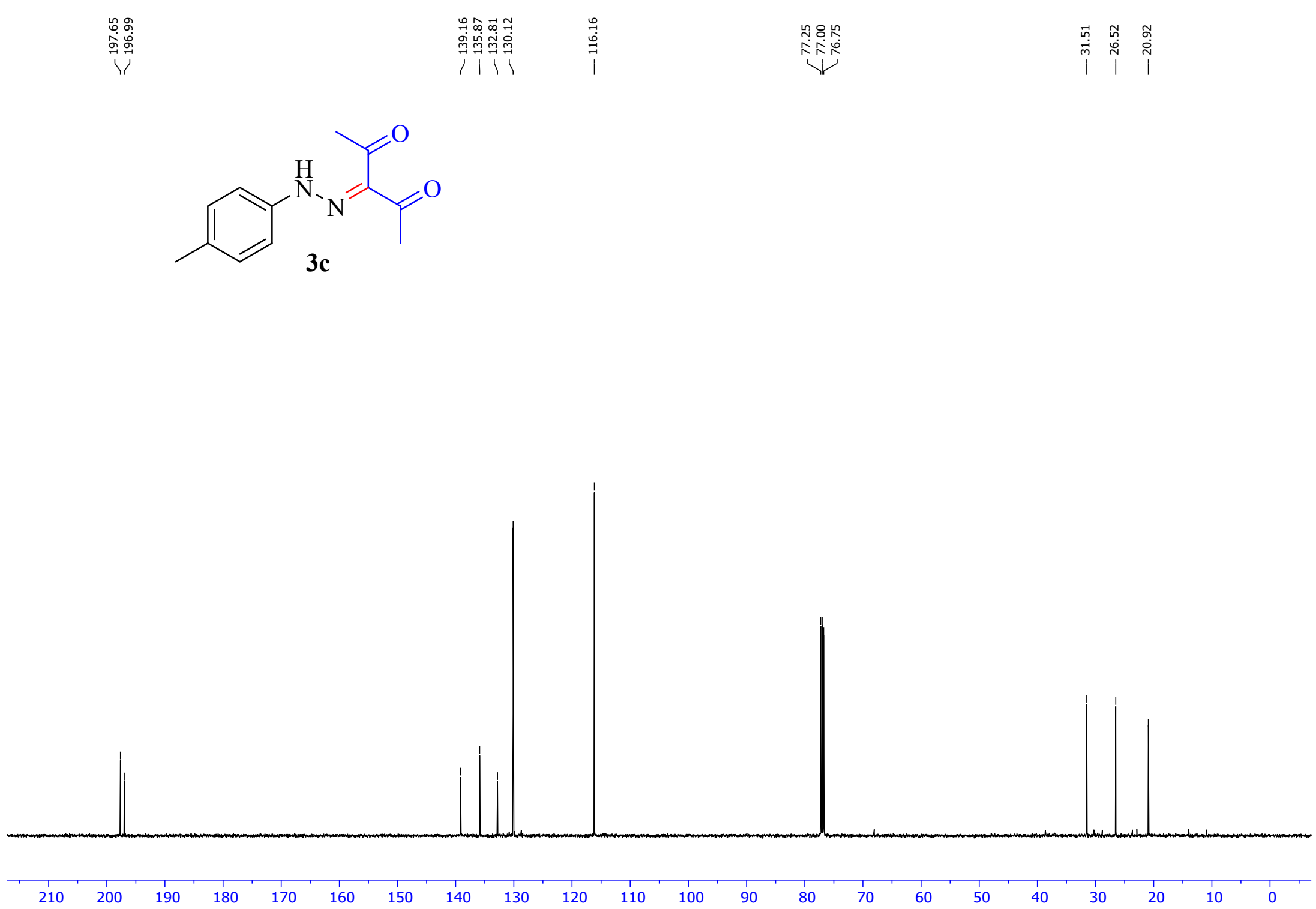
197.65
196.99
139.16
135.87
132.81
130.12
116.16
77.25
77.00
76.75
31.51
26.52
20.92
3c
f1 (ppm)

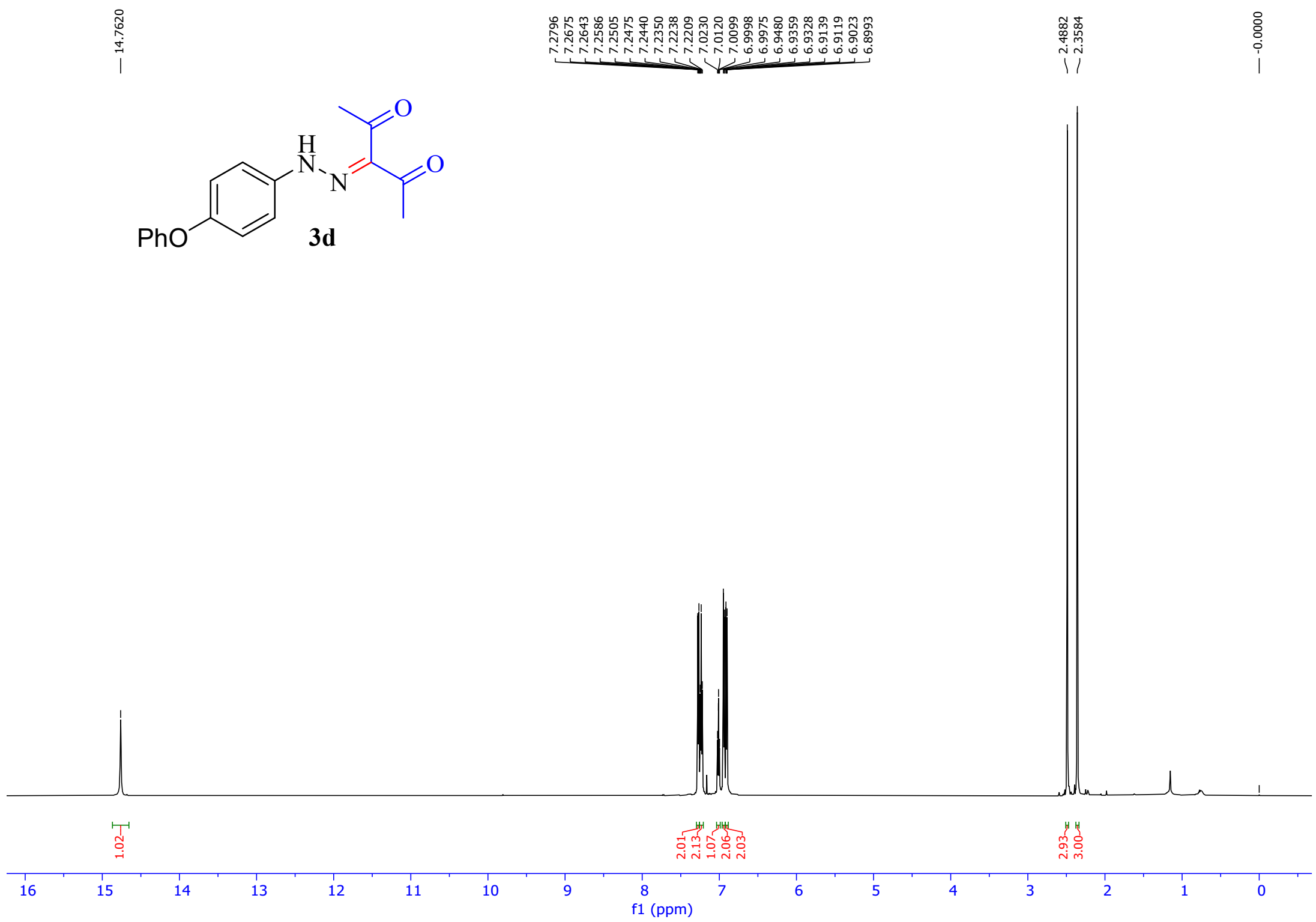
14.7620
7.2796
7.2675
7.2643
7.2586
7.2505
7.2475
7.2440
7.2350
7.2238
7.2209
7.0230
7.0120
7.0099
6.9998
6.9975
6.9480
6.9359
6.9328
6.9139
6.9119
6.9023
6.8993
2.4882
2.3584
-0.0000
PhO
3d
1.02
2.01
2.13
1.07
2.06
2.03
2.93
3.00
f1 (ppm)

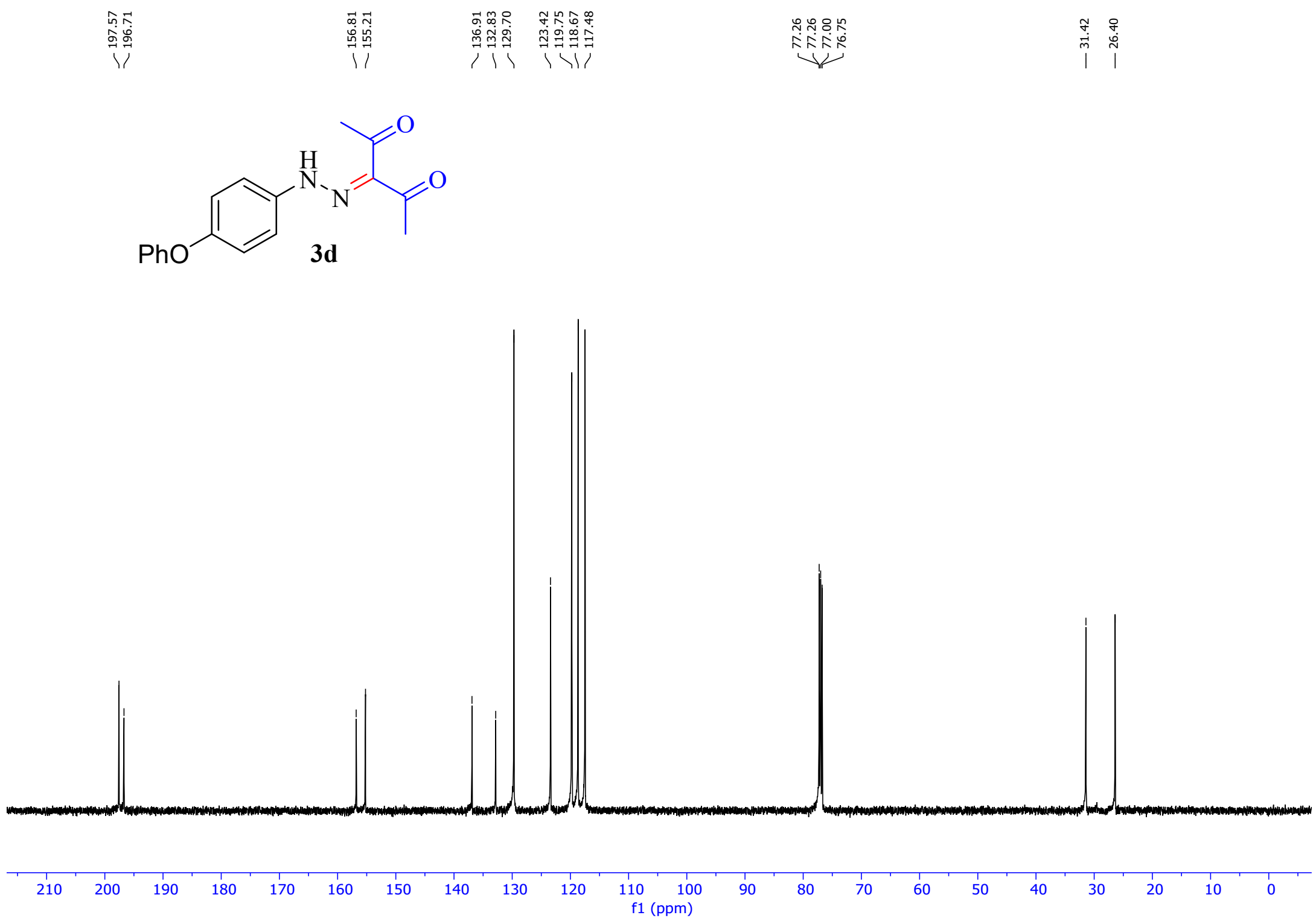
197.57
196.71
156.81
155.21
136.91
132.83
129.70
123.42
119.75
118.67
117.48
77.26
77.26
77.00
76.75
31.42
26.40
PhO
3d
f1 (ppm)

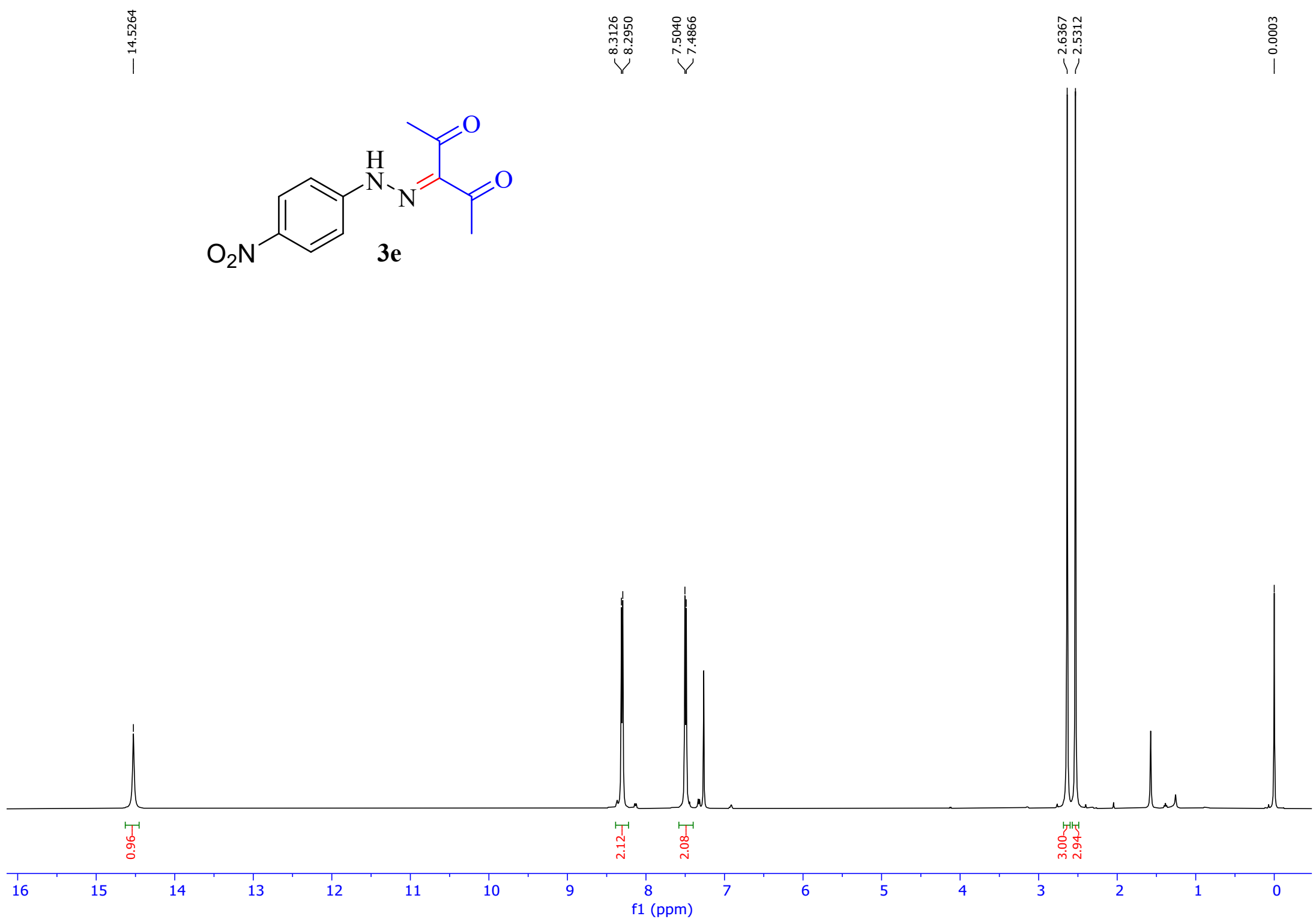
14.5264
8.3126
8.2950
7.5040
7.4866
2.6367
2.5312
0.0003
H
N
N
O
O
O_2N
3e
0.96
2.12
2.08
3.00
2.94
16 15 14 13 12 11 10 9 8 7 6 5 4 3 2 1 0
f1 (ppm)

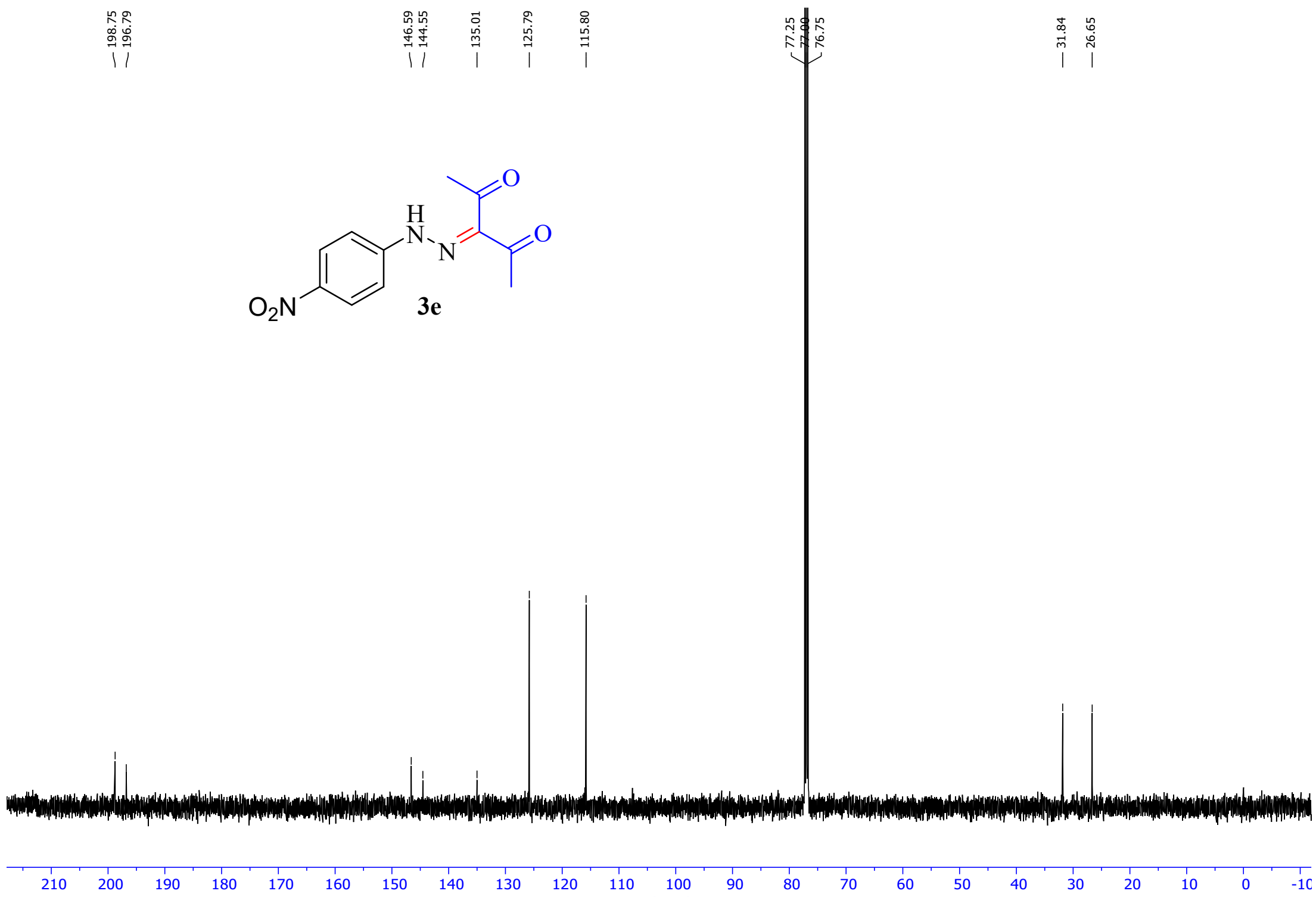
198.75
196.79
146.59
144.55
135.01
125.79
115.80
77.25
77.00
76.75
31.84
26.65
H
N
N
O
O
O_2N
3e
210 200 190 180 170 160 150 140 130 120 110 100 90 80 70 60 50 40 30 20 10 0 -10
f1 (ppm)

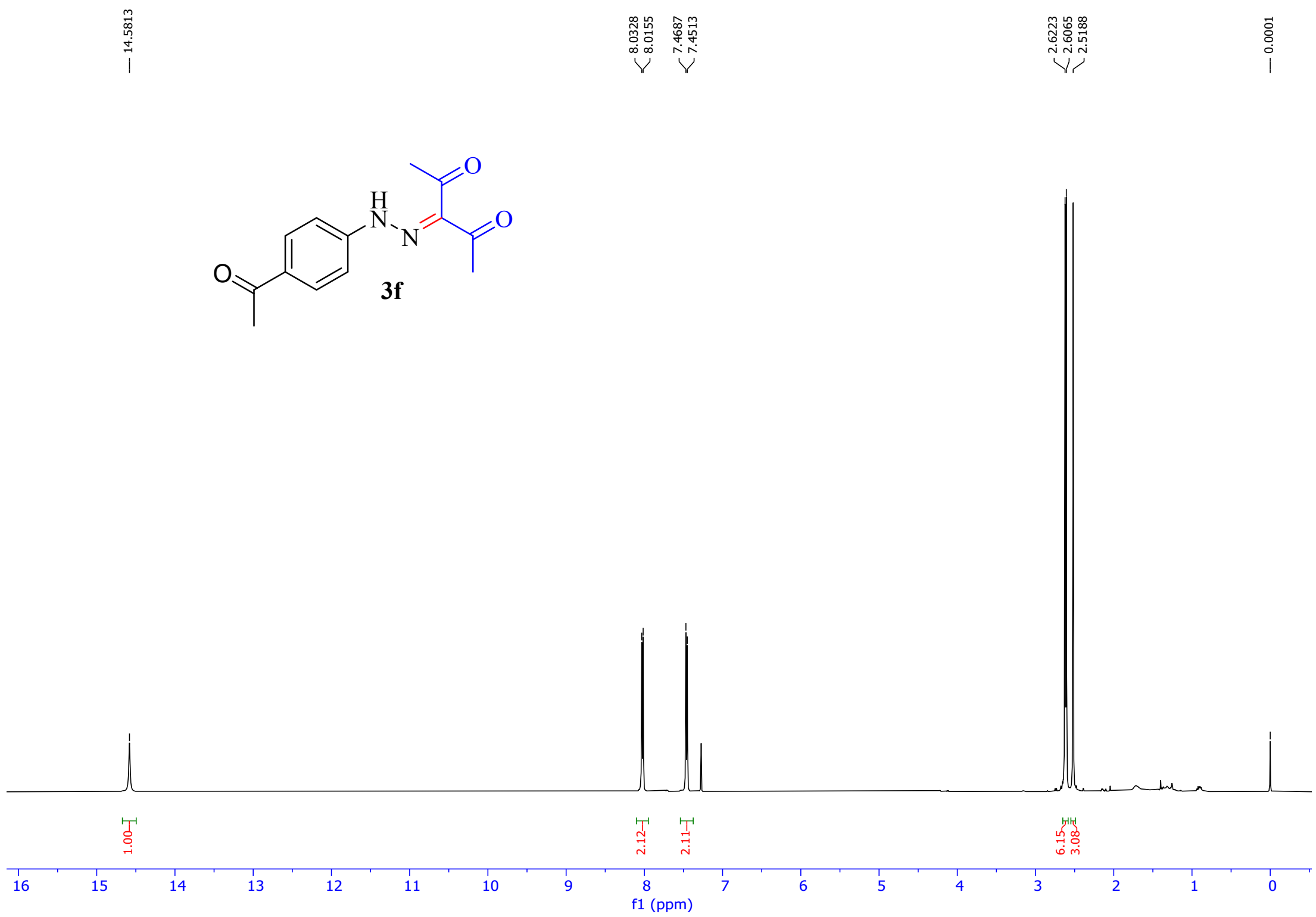
14.5813
8.0328
8.0155
7.4687
7.4513
2.6223
2.6065
2.5188
0.0001
3f
1.00
2.12
2.11
6.15
3.08
16 15 14 13 12 11 10 9 8 7 6 5 4 3 2 1 0
f1 (ppm)

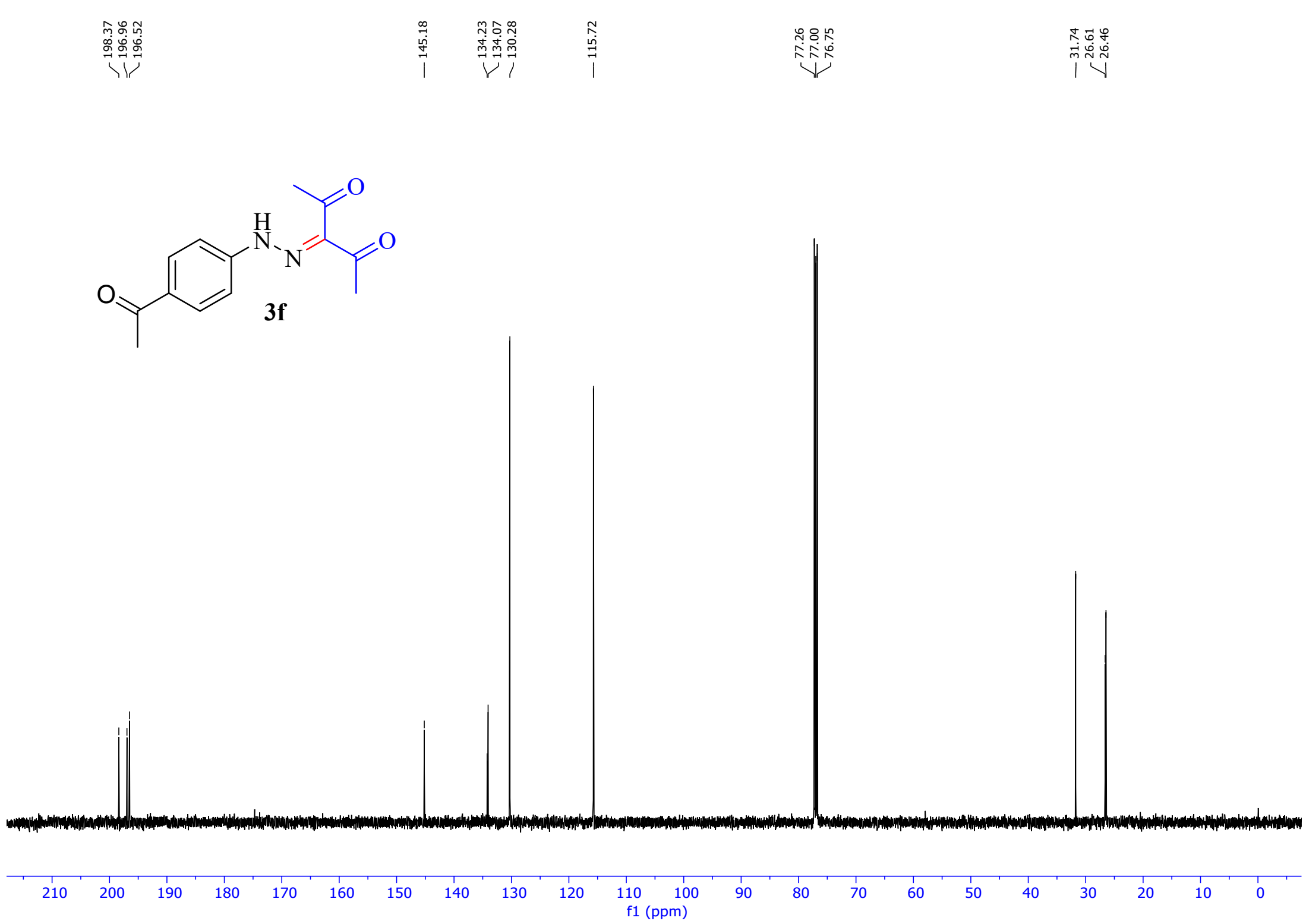
198.37
196.96
196.52
145.18
134.23
134.07
130.28
115.72
77.26
77.00
76.75
31.74
26.61
26.46
3f
210 200 190 180 170 160 150 140 130 120 110 100 90 80 70 60 50 40 30 20 10 0
f1 (ppm)

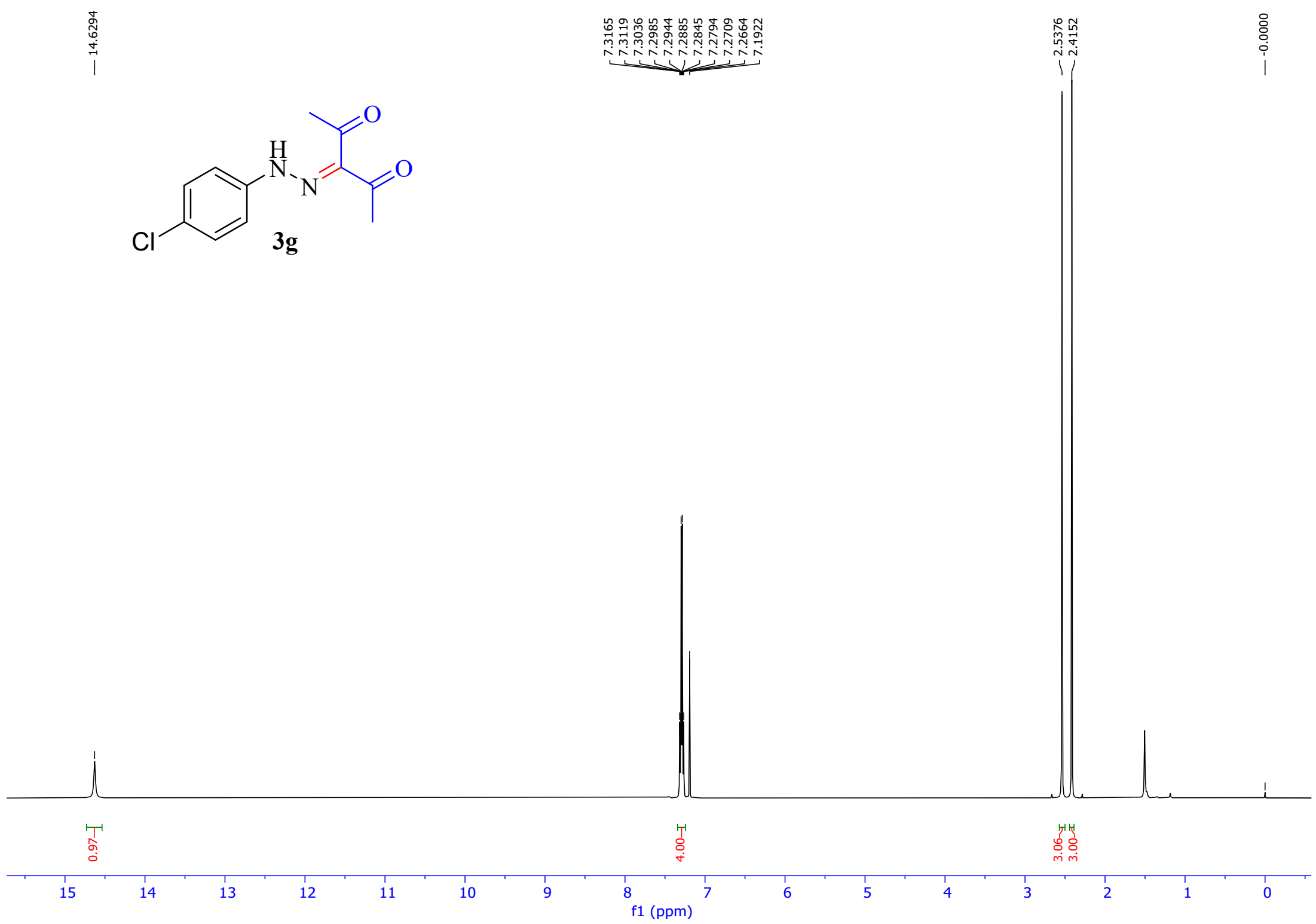
14.6294
7.3165
7.3119
7.3036
7.2985
7.2944
7.2885
7.2845
7.2794
7.2709
7.2664
7.1922
2.5376
2.4152
-0.0000
3g
Cl
H
N
O
0.97
4.00
3.06
3.00
15 14 13 12 11 10 9 8 7 6 5 4 3 2 1 0
f1 (ppm)

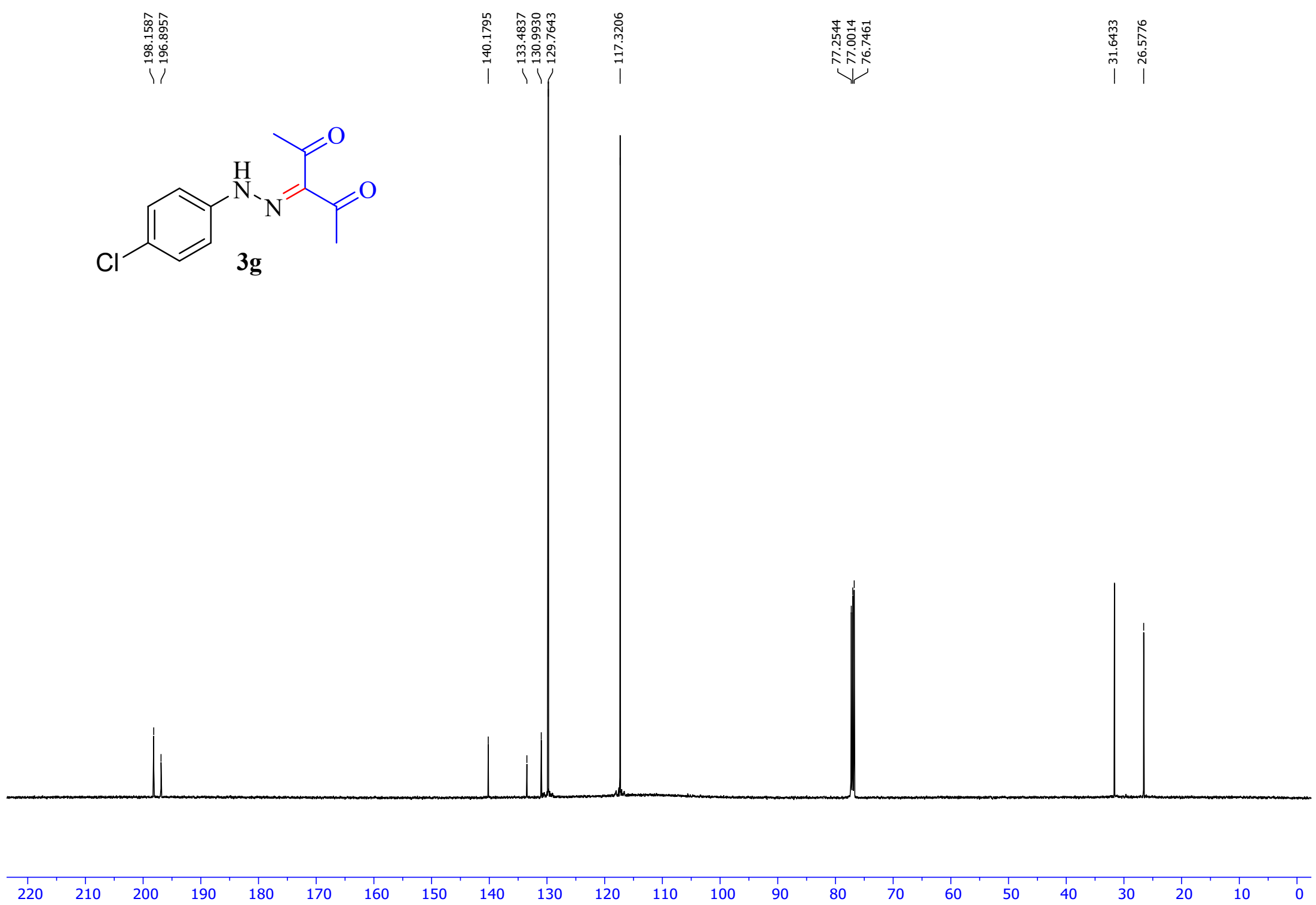
198.1587
196.8957
140.1795
133.4837
130.9930
129.7643
117.3206
77.2544
77.0014
76.7461
31.6433
26.5776
3g
Cl
H
N
O
220 210 200 190 180 170 160 150 140 130 120 110 100 90 80 70 60 50 40 30 20 10 0
f1 (ppm)

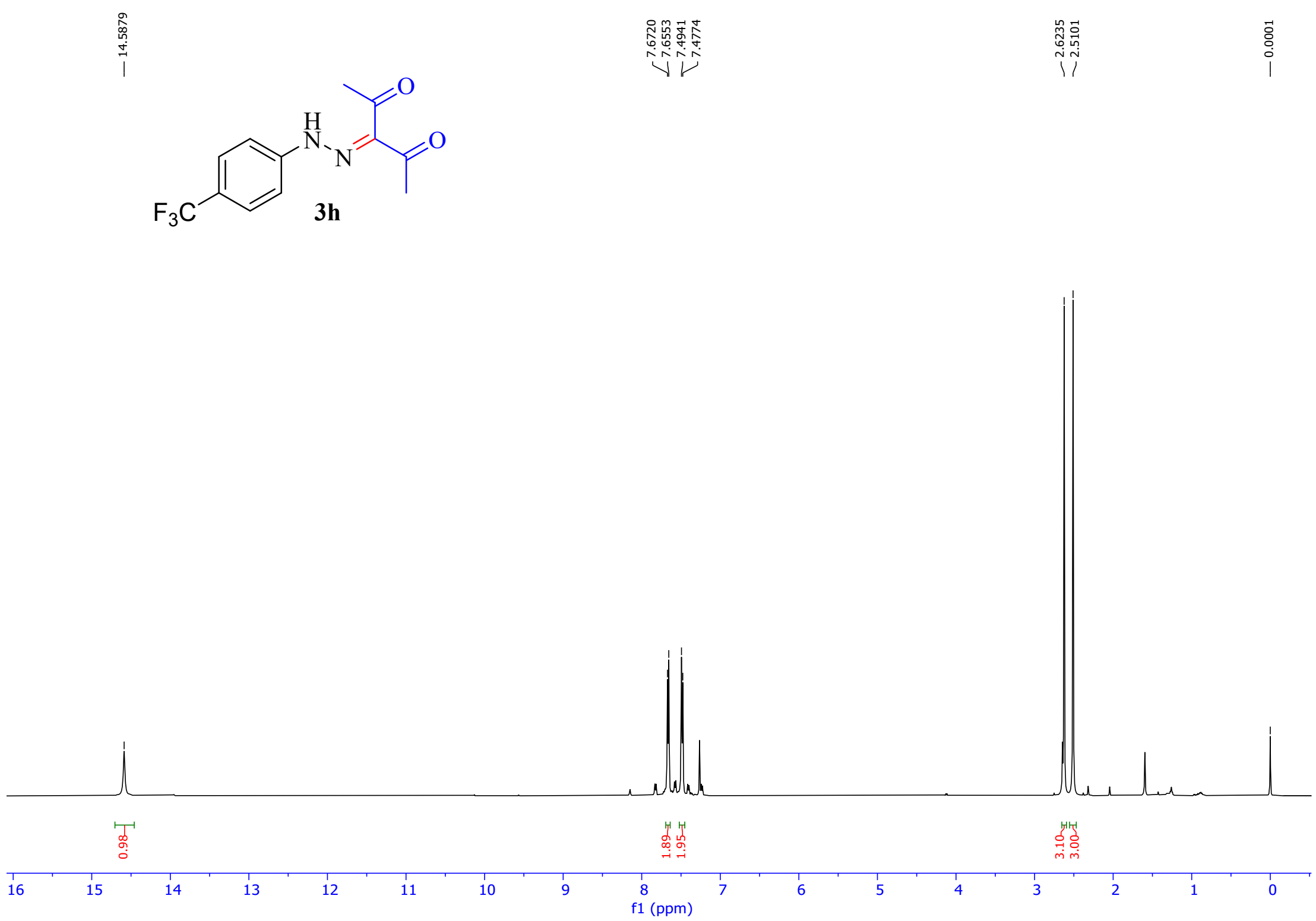
14.5879
7.6720
7.6553
7.4941
7.4774
2.6235
2.5101
0.0001
F3C
3h
0.98
1.89
1.95
3.10
3.00
16 15 14 13 12 11 10 9 8 7 6 5 4 3 2 1 0
f1 (ppm)

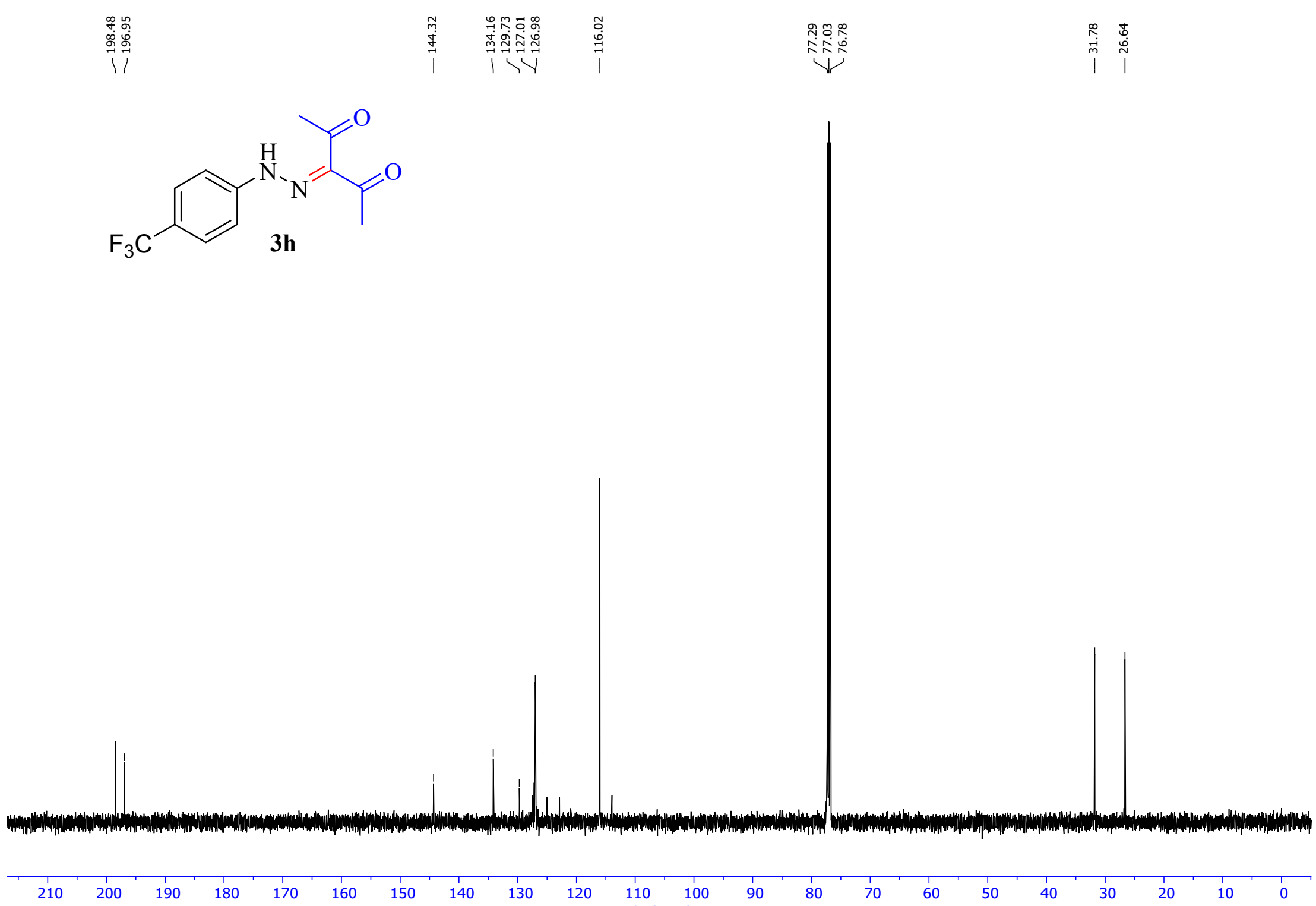
198.48
196.95
144.32
134.16
129.73
127.01
126.98
116.02
77.29
77.03
76.78
31.78
26.64
F3C
3h
210 200 190 180 170 160 150 140 130 120 110 100 90 80 70 60 50 40 30 20 10 0
f1 (ppm)

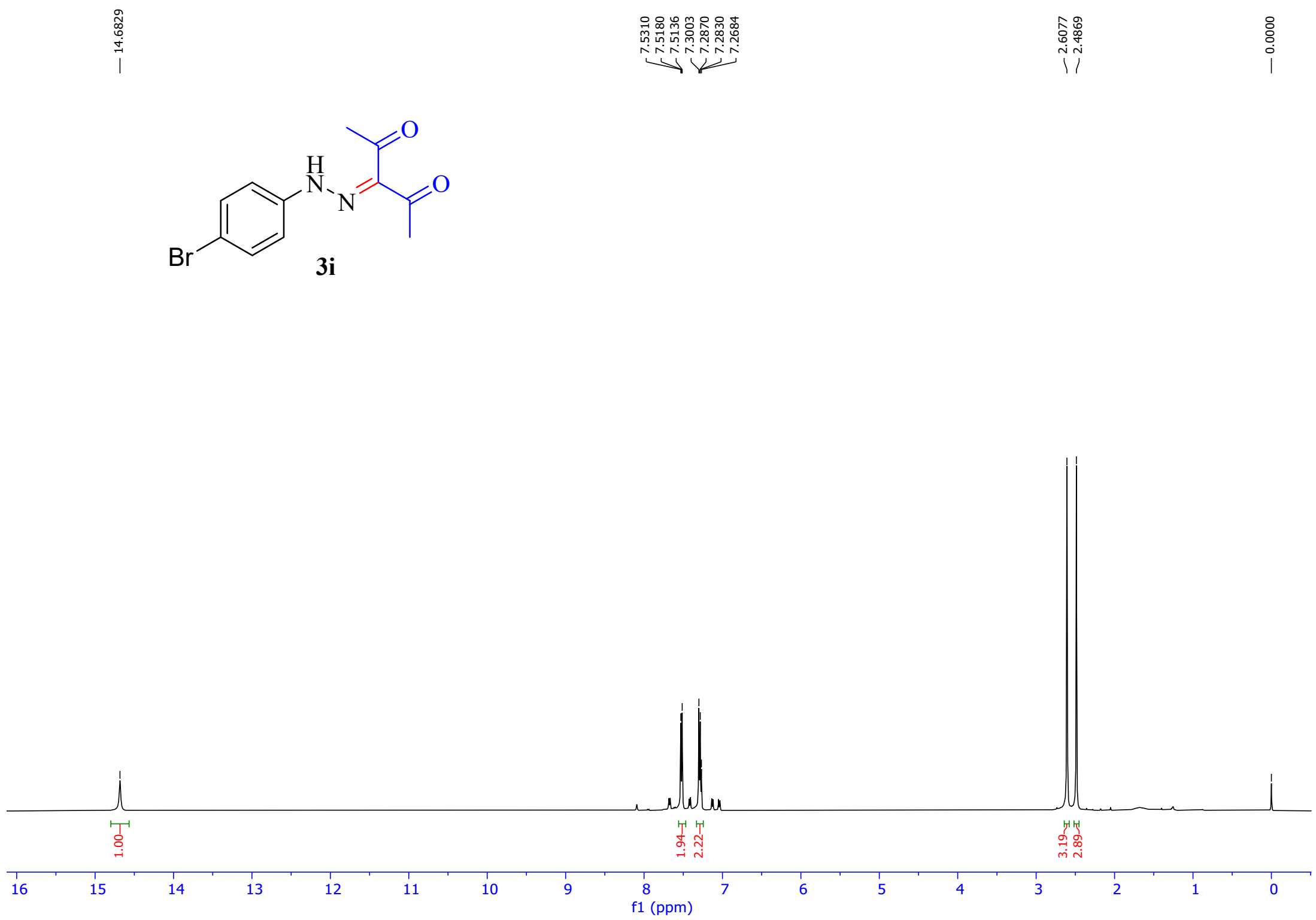
14.6829
7.5310
7.5180
7.5136
7.3003
7.2870
7.2830
7.2684
2.6077
2.4869
0.0000
H
N
N
O
O
Br
3i
1.00
1.94
2.22
3.19
2.89
16 15 14 13 12 11 10 9 8 7 6 5 4 3 2 1 0
f1 (ppm)

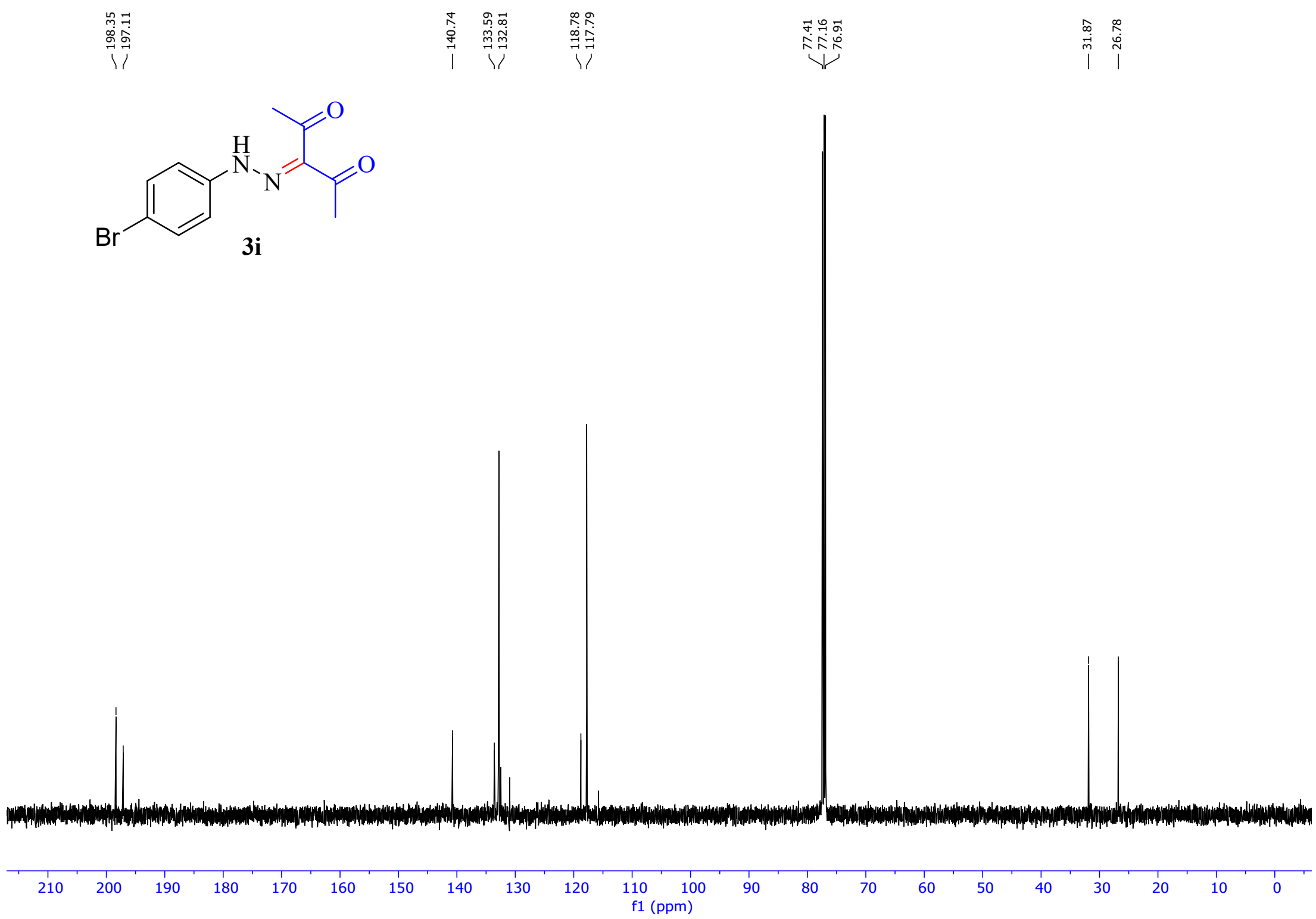
198.35
197.11
140.74
133.59
132.81
118.78
117.79
77.41
77.16
76.91
31.87
26.78
H
N
N
O
O
Br
3i
210 200 190 180 170 160 150 140 130 120 110 100 90 80 70 60 50 40 30 20 10 0
f1 (ppm)

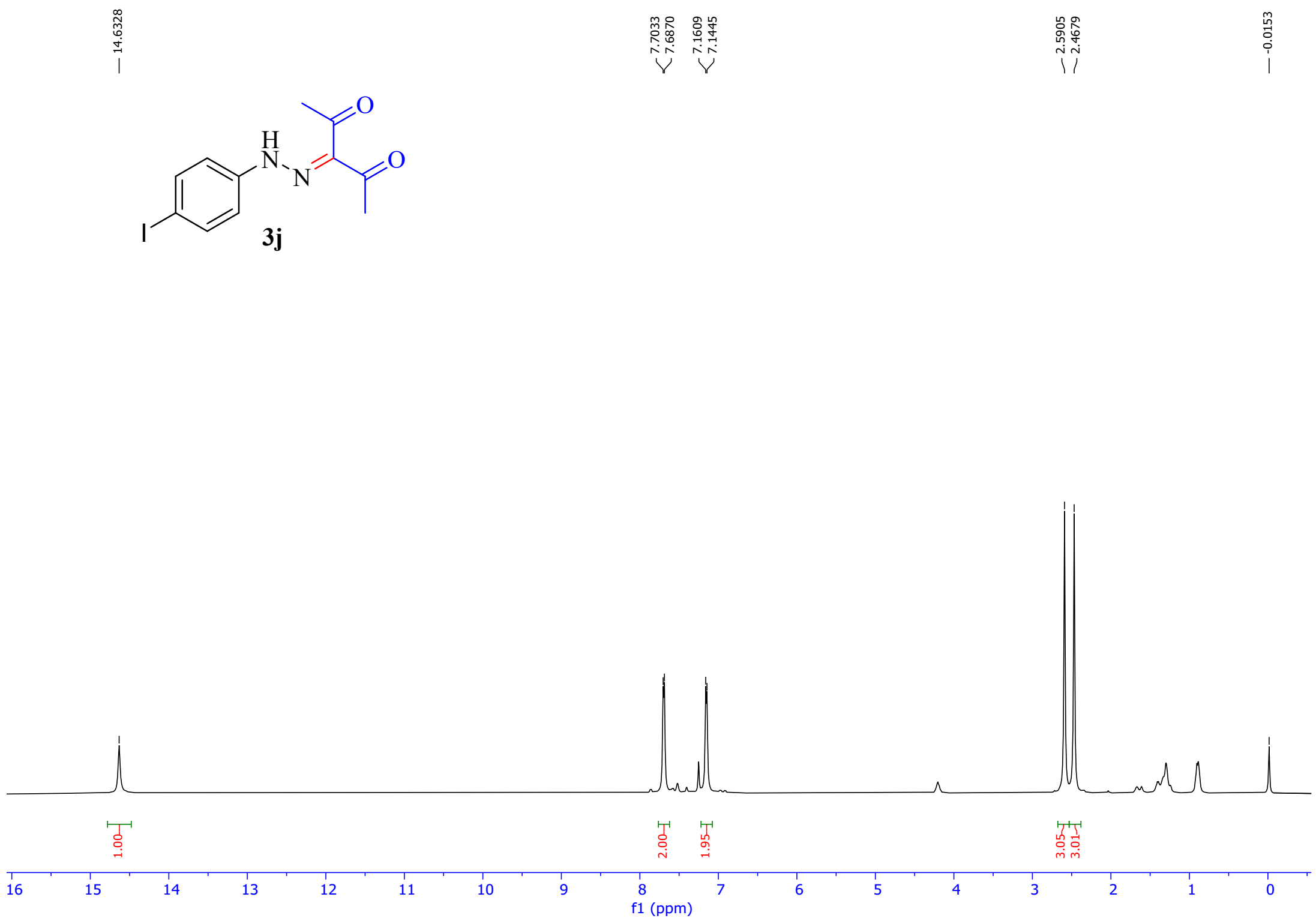
14.6328
7.7033
7.6870
7.1609
7.1445
2.5905
2.4679
-0.0153
H
N
N
O
O
I
3j
1.00
2.00
1.95
3.05
3.01
16 15 14 13 12 11 10 9 8 7 6 5 4 3 2 1 0
f1 (ppm)

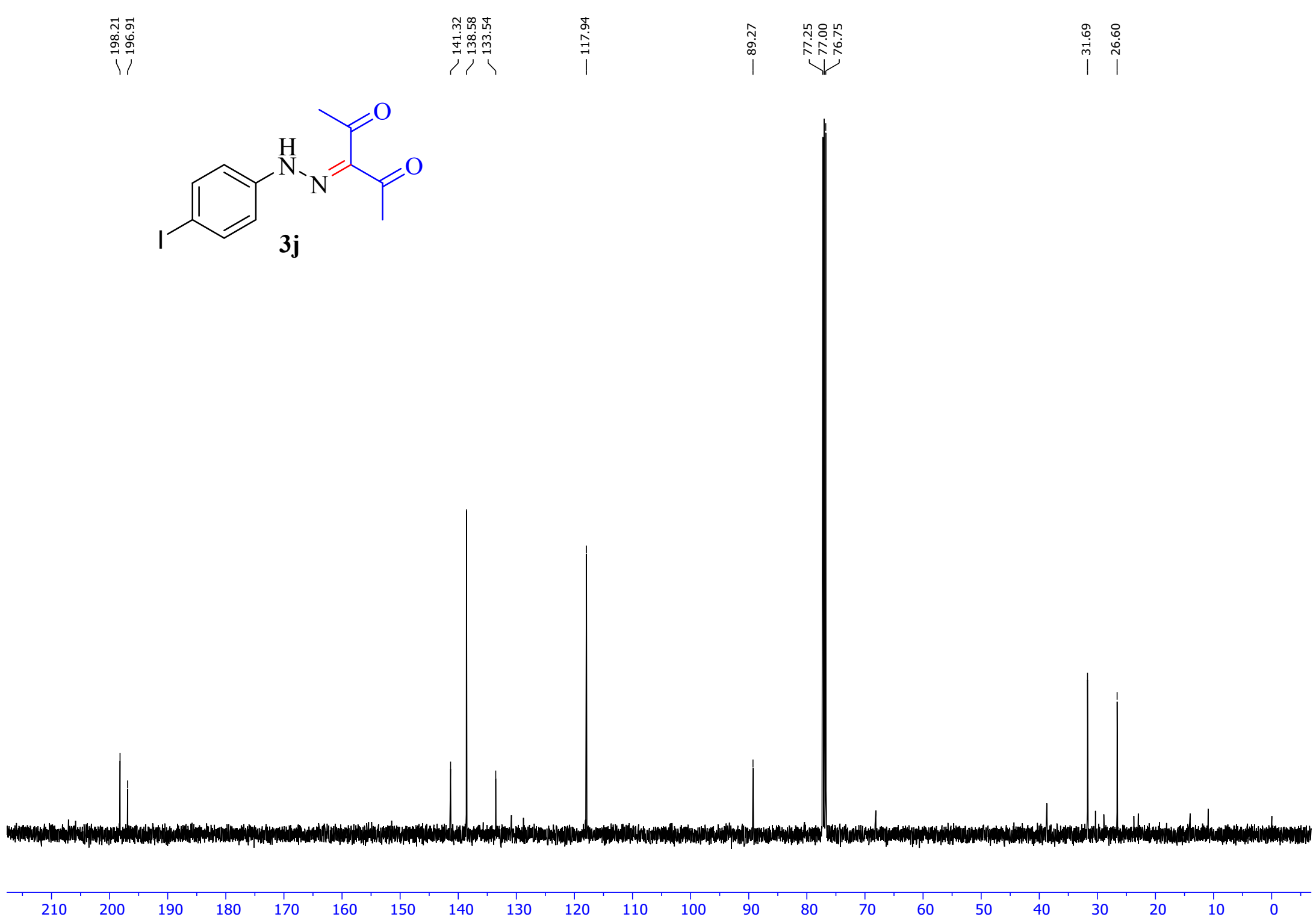
198.21
196.91
141.32
138.58
133.54
117.94
89.27
77.25
77.00
76.75
31.69
26.60
H
N
N
O
O
I
3j
210 200 190 180 170 160 150 140 130 120 110 100 90 80 70 60 50 40 30 20 10 0
f1 (ppm)

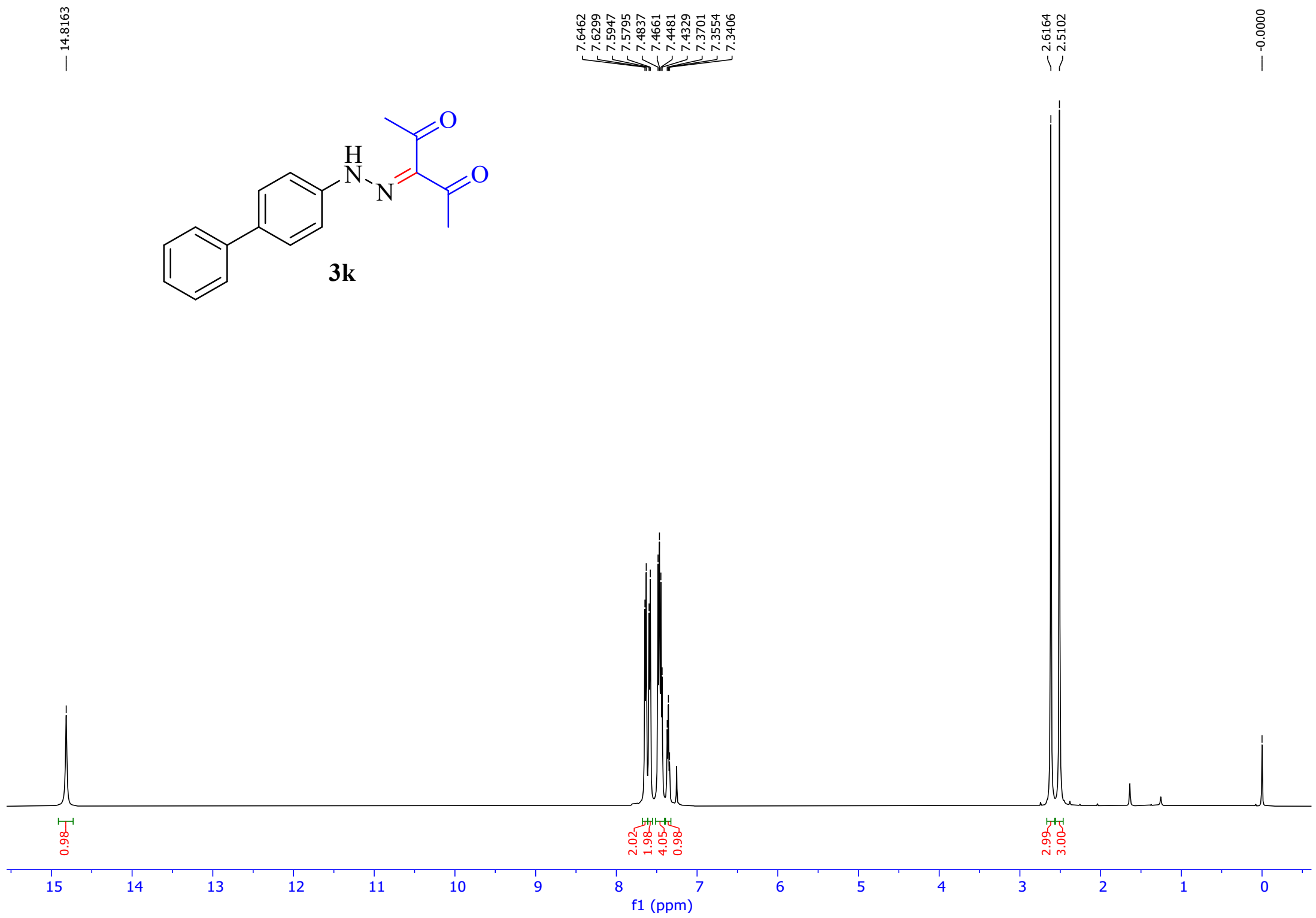
14.8163
7.6462
7.6299
7.5947
7.5795
7.4837
7.4661
7.4481
7.4329
7.3701
7.3554
7.3406
2.6164
2.5102
-0.0000
H
N
N
O
O
3k
0.98
2.02
1.98
4.05
0.98
2.99
3.00
f1 (ppm)

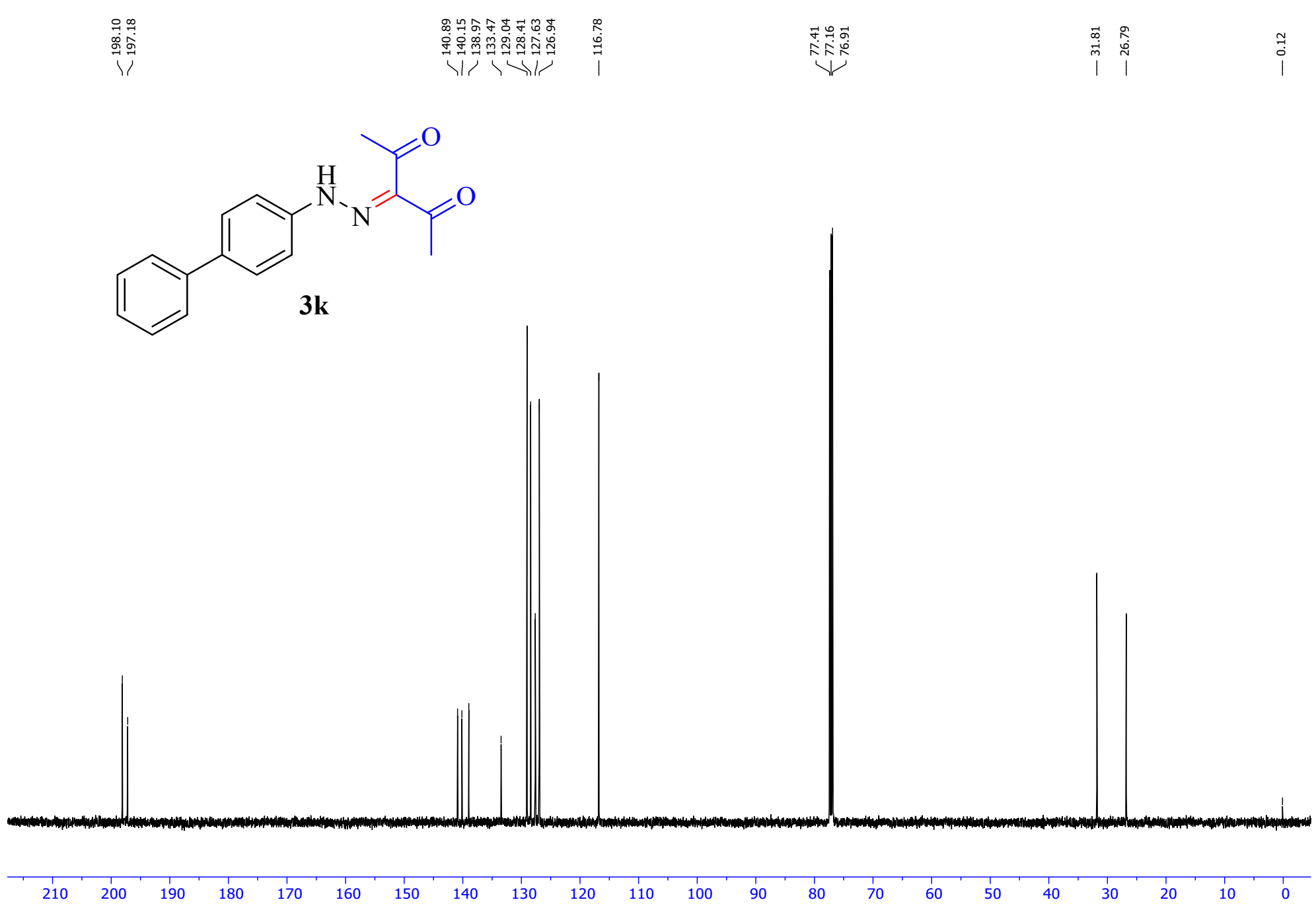
198.10
197.18
140.89
140.15
138.97
133.47
129.04
128.41
127.63
126.94
116.78
77.41
77.16
76.91
31.81
26.79
0.12
H
N
N
O
O
3k
f1 (ppm)

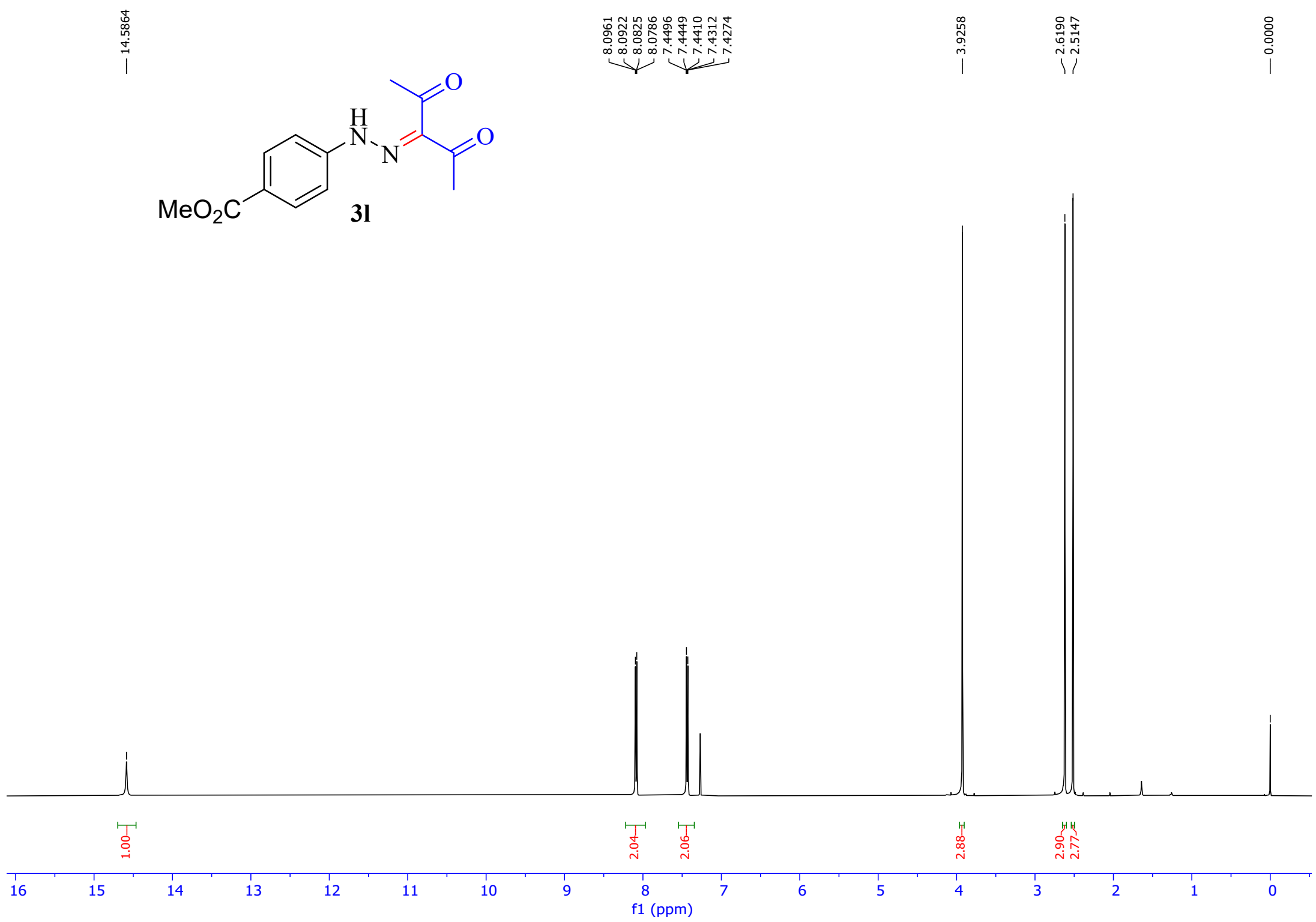
14.5864
8.0961
8.0922
8.0825
8.0786
7.4496
7.4449
7.4410
7.4312
7.4274
3.9258
2.6190
2.5147
0.0000
H
N
N
O
O
MeO2C
3l
1.00
2.04
2.06
2.88
2.90
2.77
16
15
14
13
12
11
10
9
8
7
6
5
4
3
2
1
0
f1 (ppm)

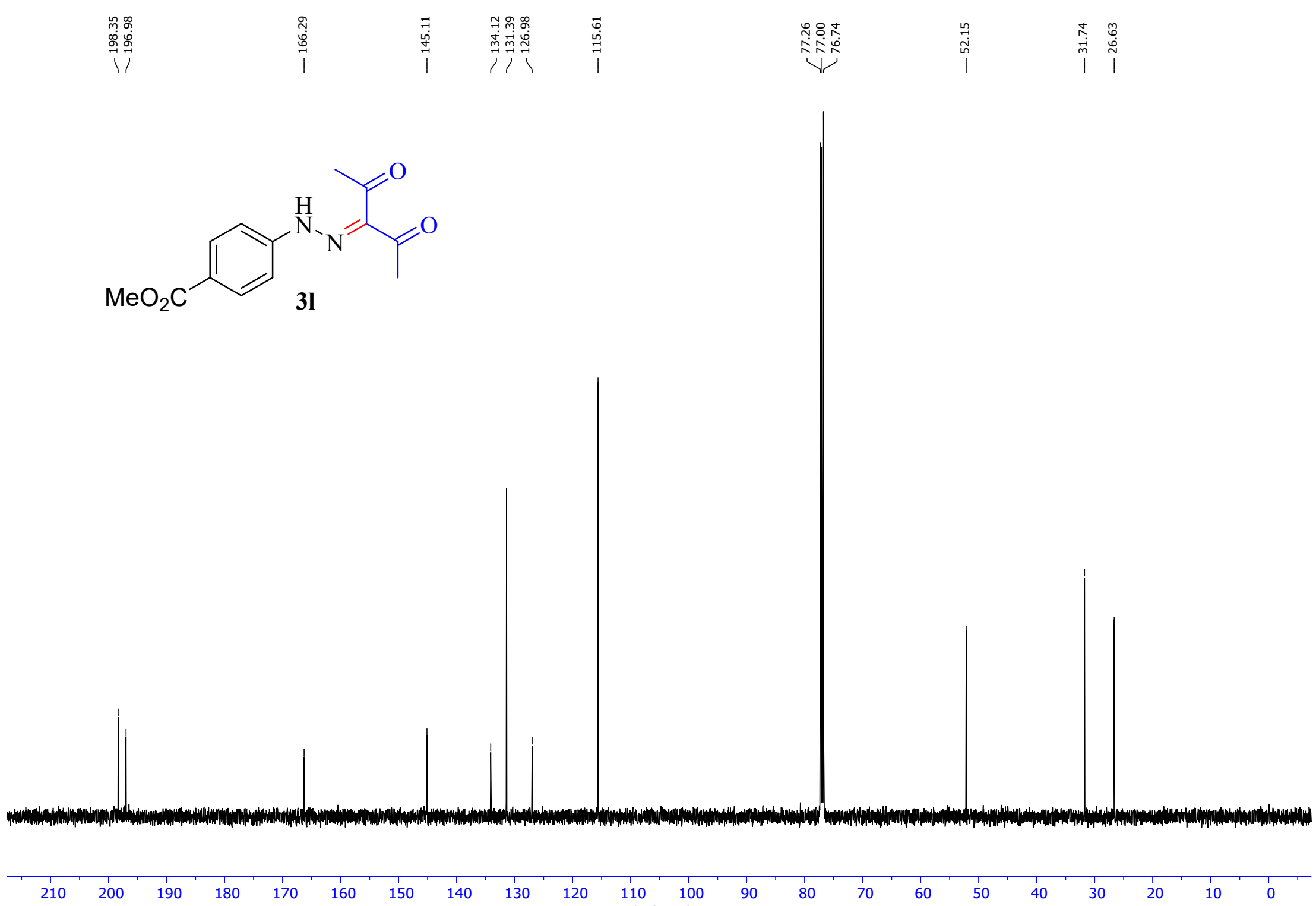
198.35
196.98
166.29
145.11
134.12
131.39
126.98
115.61
77.26
77.00
76.74
52.15
31.74
26.63
H
N
N
O
O
MeO2C
3l
210
200
190
180
170
160
150
140
130
120
110
100
90
80
70
60
50
40
30
20
10
0
f1 (ppm)

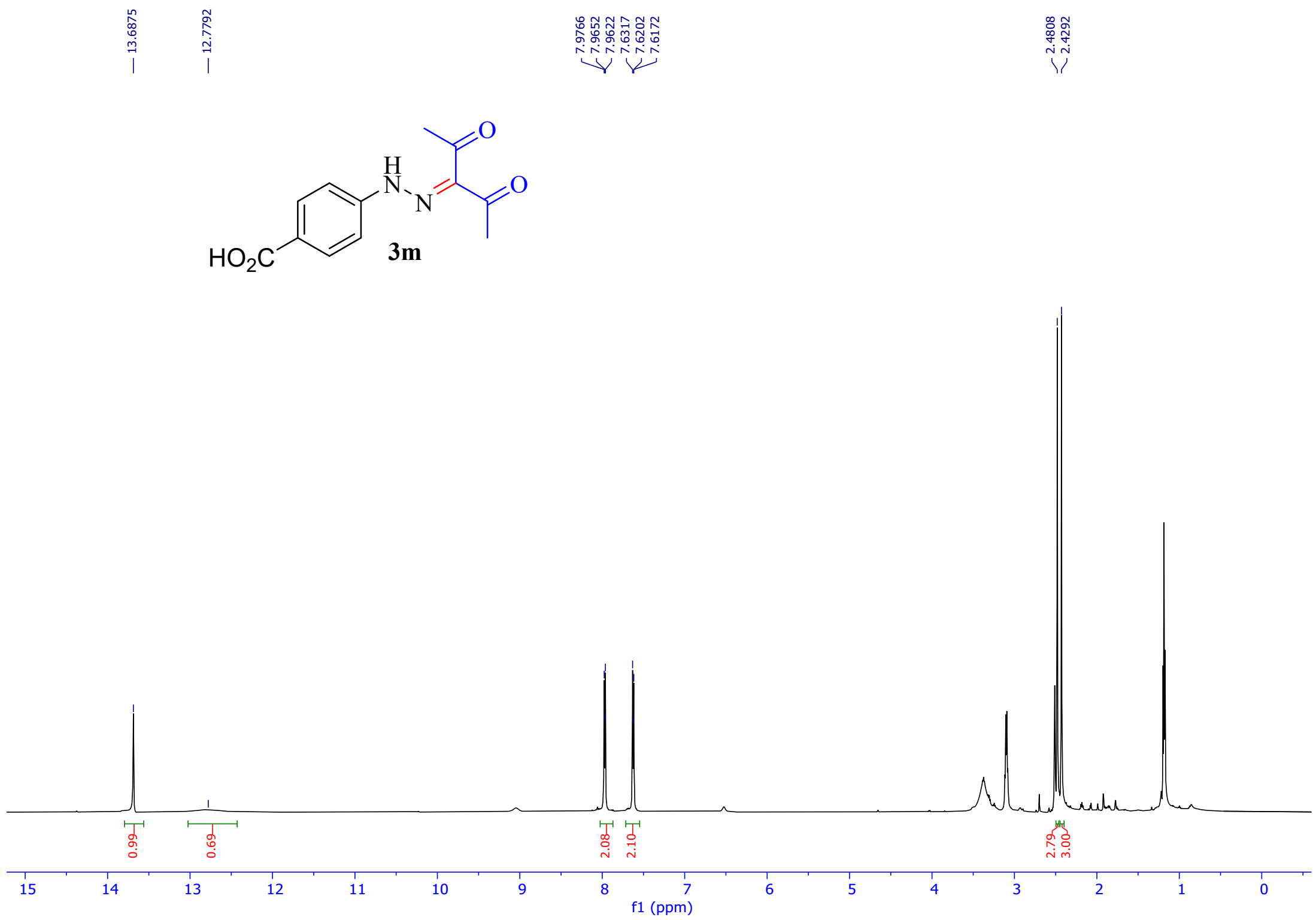
13.6875
12.7792
7.9766
7.9652
7.9622
7.6317
7.6202
7.6172
2.4808
2.4292
HO2C
3m
0.99
0.69
2.08
2.10
2.79
3.00
f1 (ppm)

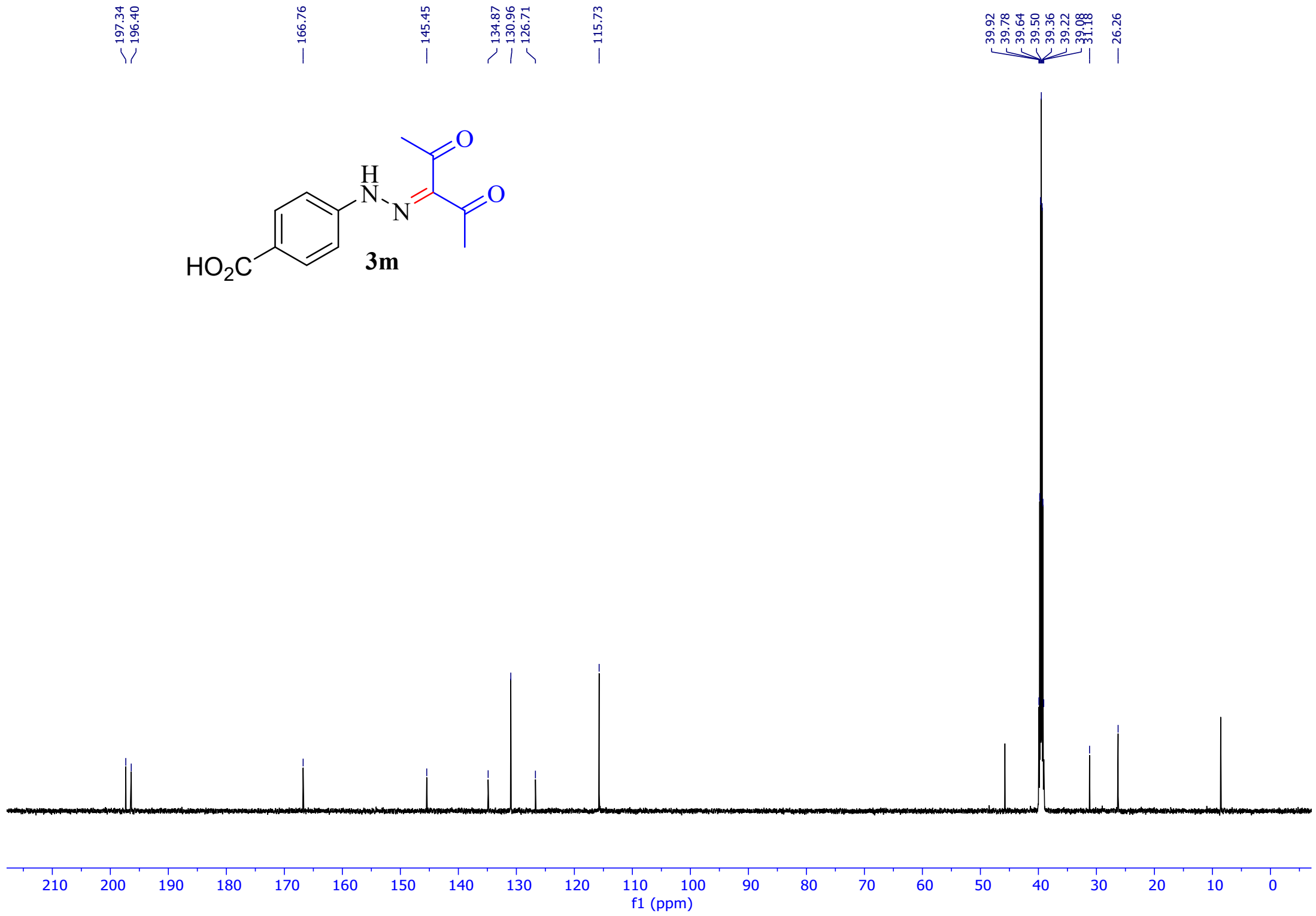
197.34
196.40
166.76
145.45
134.87
130.96
126.71
115.73
39.92
39.78
39.64
39.50
39.36
39.22
39.08
31.18
26.26
HO2C
3m
f1 (ppm)

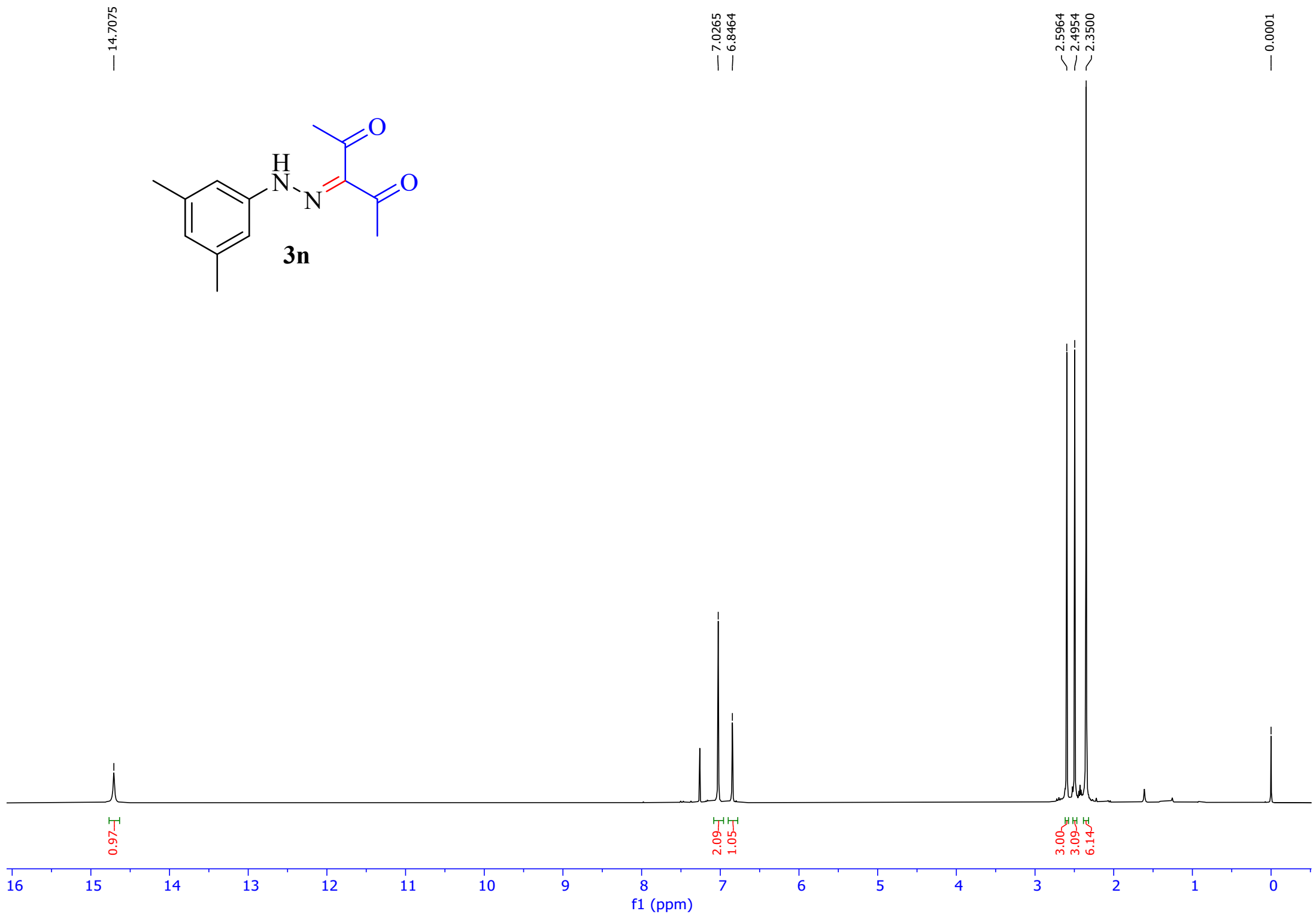
14.7075
7.0265
6.8464
2.5964
2.4954
2.3500
0.0001
3n
0.97
2.09
1.05
3.00
3.09
6.14
16 15 14 13 12 11 10 9 8 7 6 5 4 3 2 1 0
f1 (ppm)

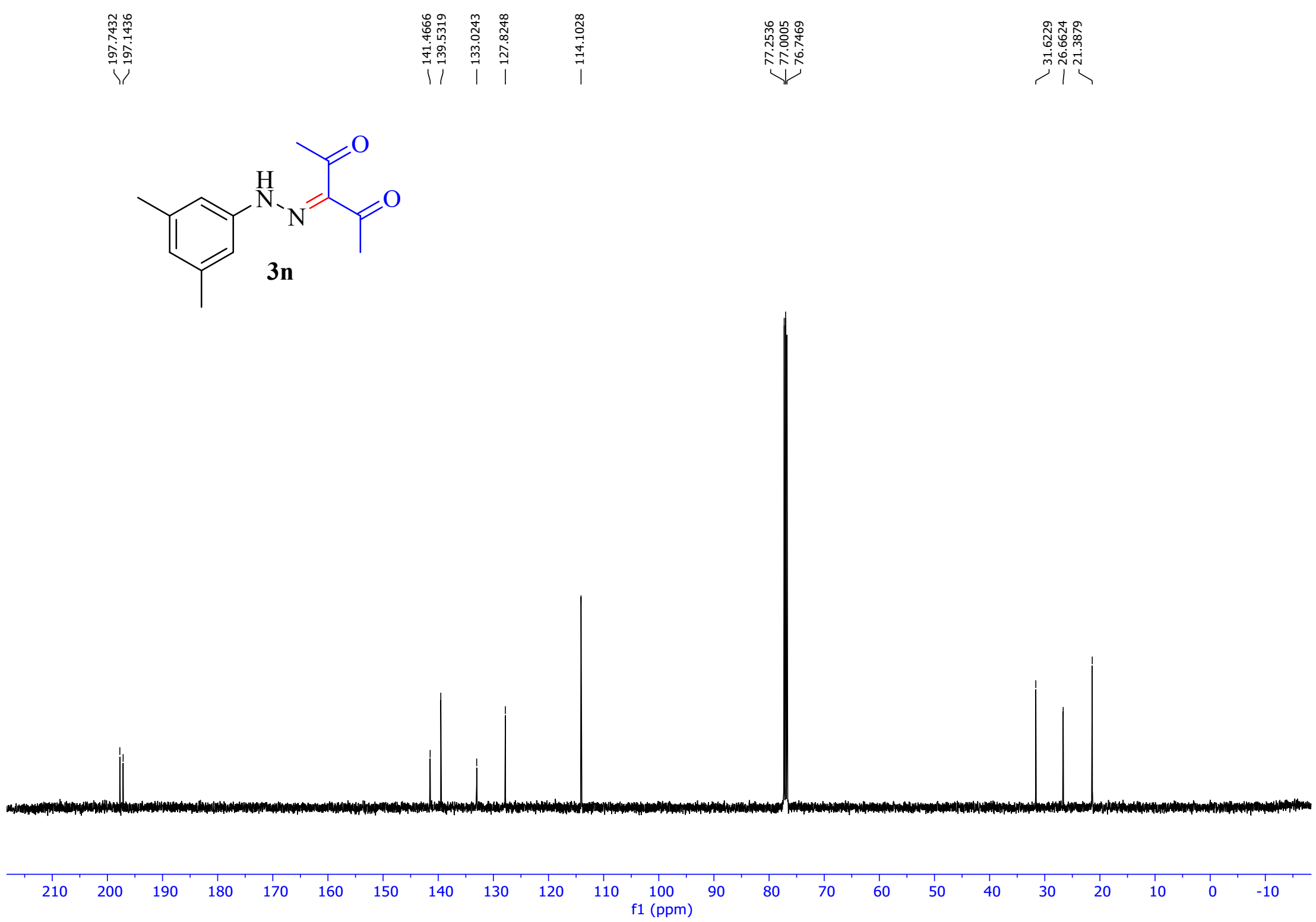
197.7432
197.1436
141.4666
139.5319
133.0243
127.8248
114.1028
77.2536
77.0005
76.7469
31.6229
26.6624
21.3879
3n
210 200 190 180 170 160 150 140 130 120 110 100 90 80 70 60 50 40 30 20 10 0 -10
f1 (ppm)

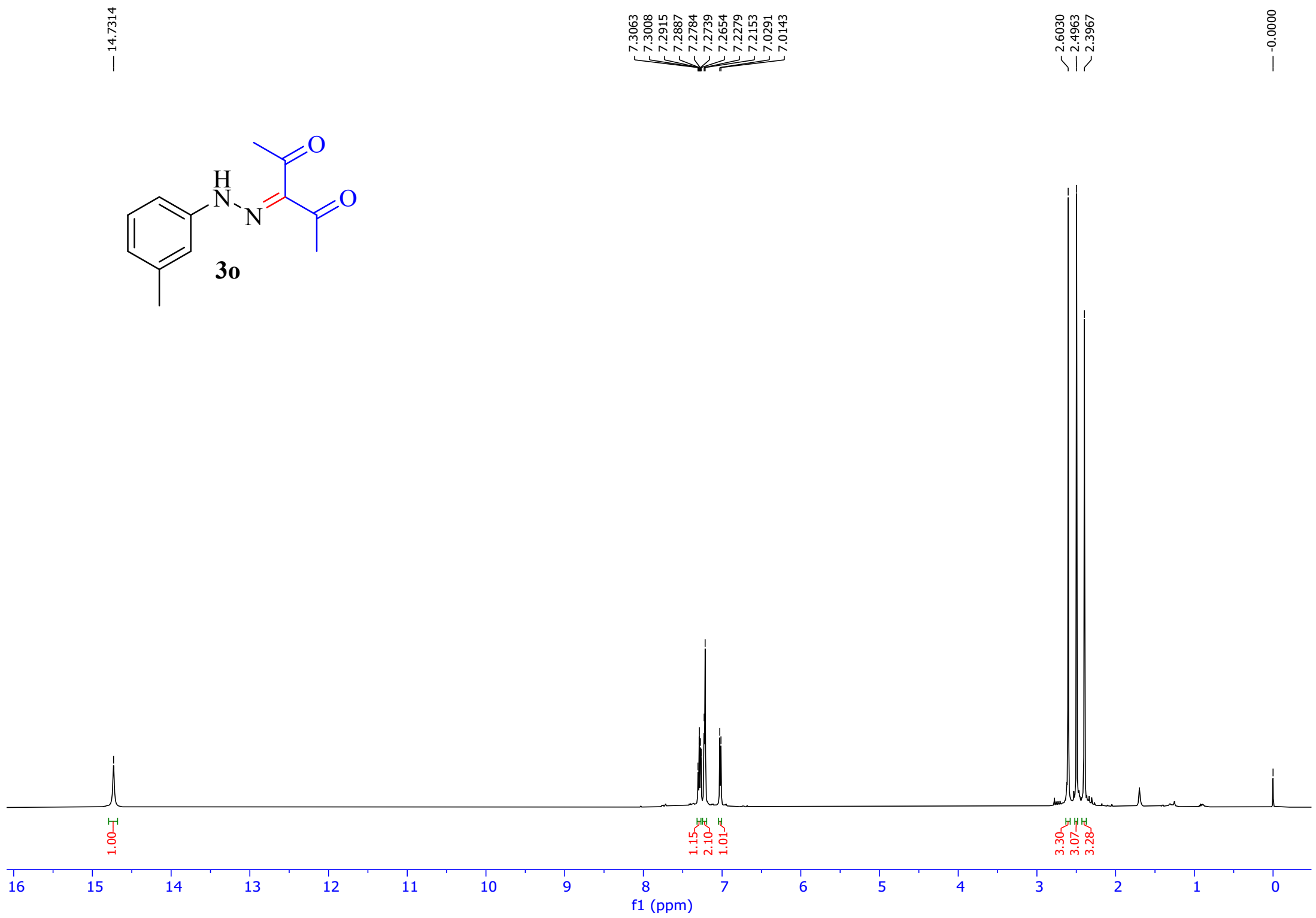
14.7314
7.3063
7.3008
7.2915
7.2887
7.2784
7.2739
7.2654
7.2279
7.2153
7.0291
7.0143
2.6030
2.4963
2.3967
-0.0000
3o
1.00
1.15
2.10
1.01
3.30
3.07
3.28
f1 (ppm)

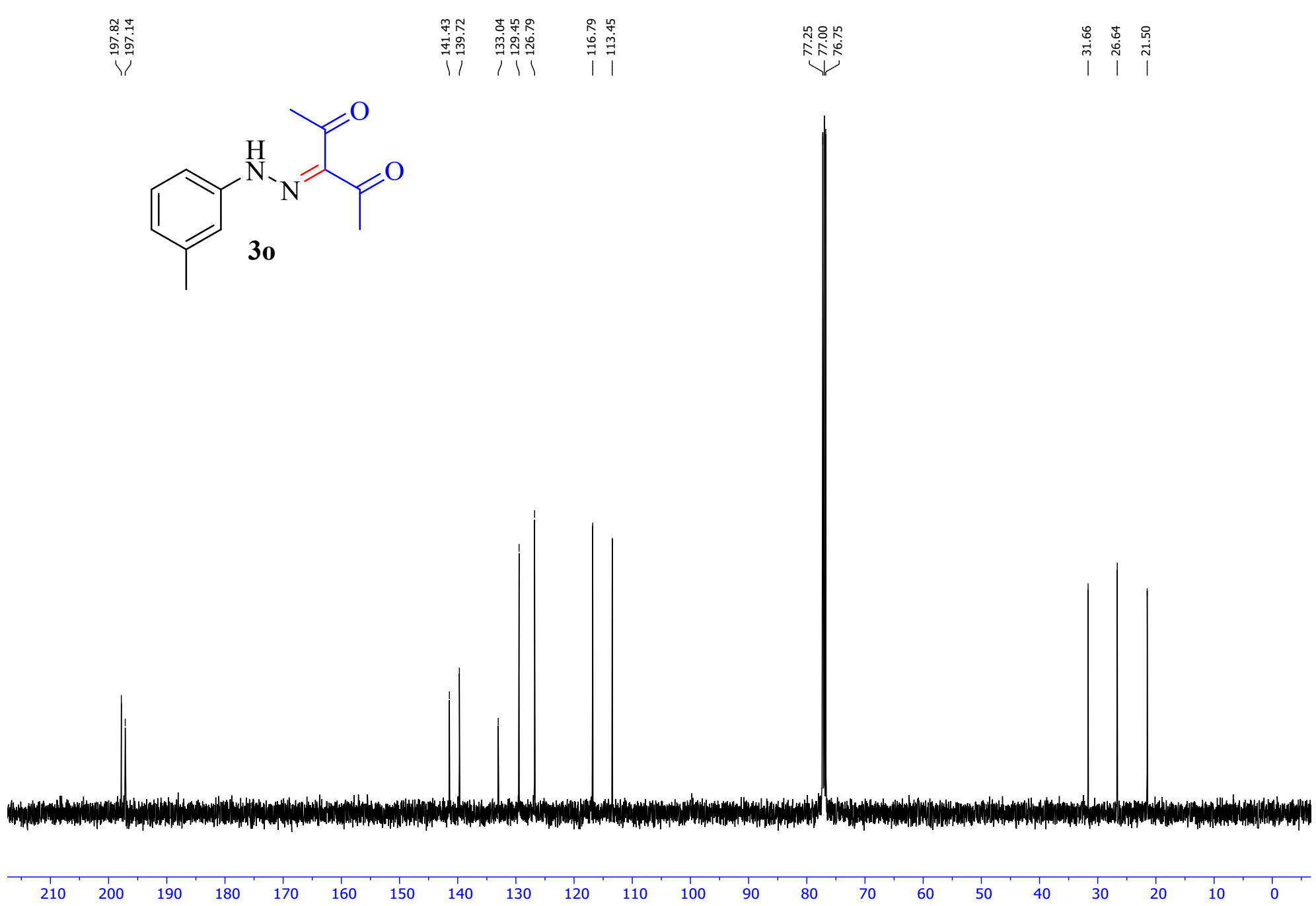
197.82
197.14
141.43
139.72
133.04
129.45
126.79
116.79
113.45
77.25
77.00
76.75
31.66
26.64
21.50
3o
f1 (ppm)

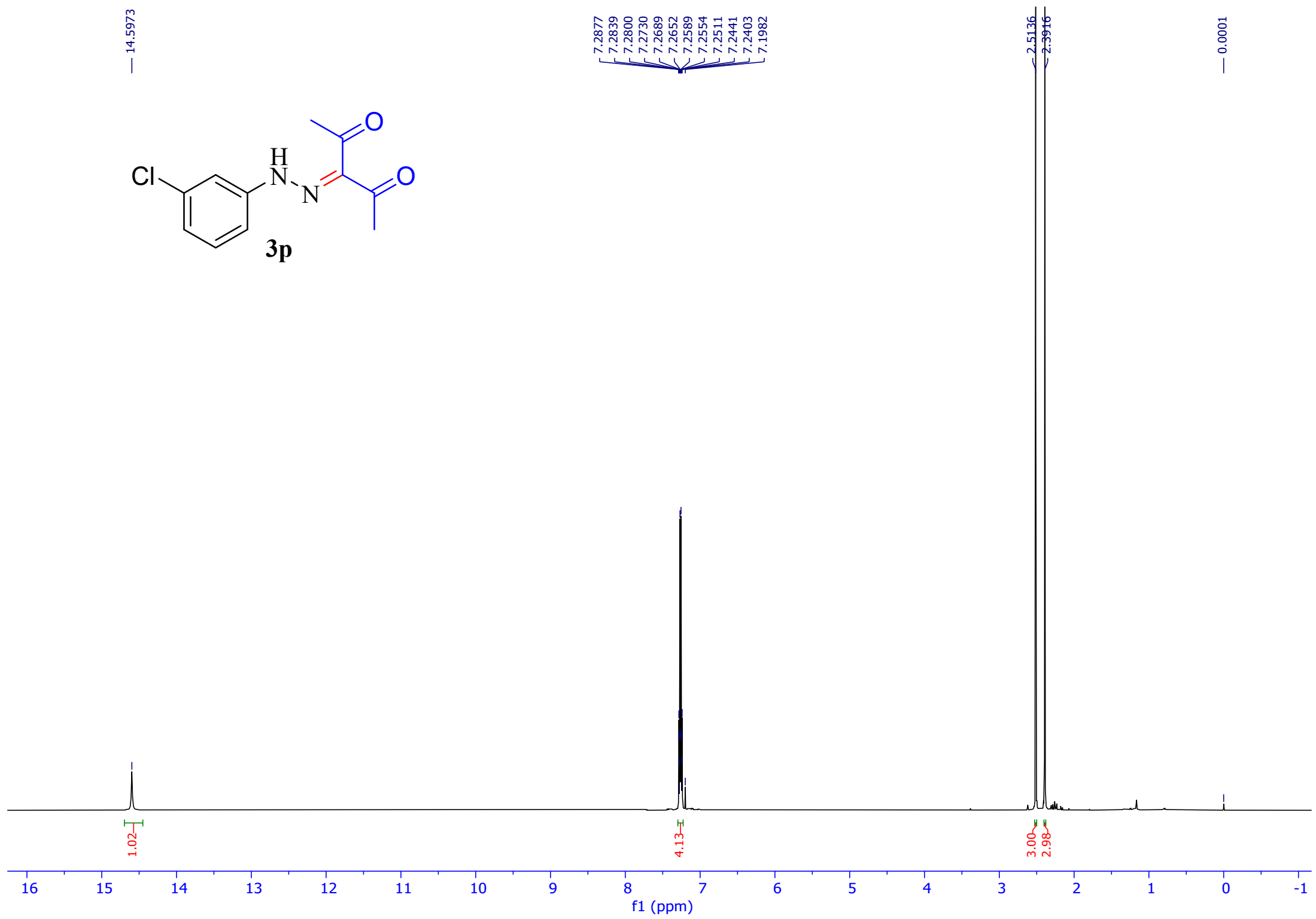
14.5973
7.2877
7.2839
7.2800
7.2730
7.2689
7.2652
7.2589
7.2554
7.2511
7.2441
7.2403
7.1982
2.5136
2.3916
0.0001
Cl
H
N
N
O
O
3p
1.02
4.13
3.00
2.98
16 15 14 13 12 11 10 9 8 7 6 5 4 3 2 1 0 -1
f1 (ppm)

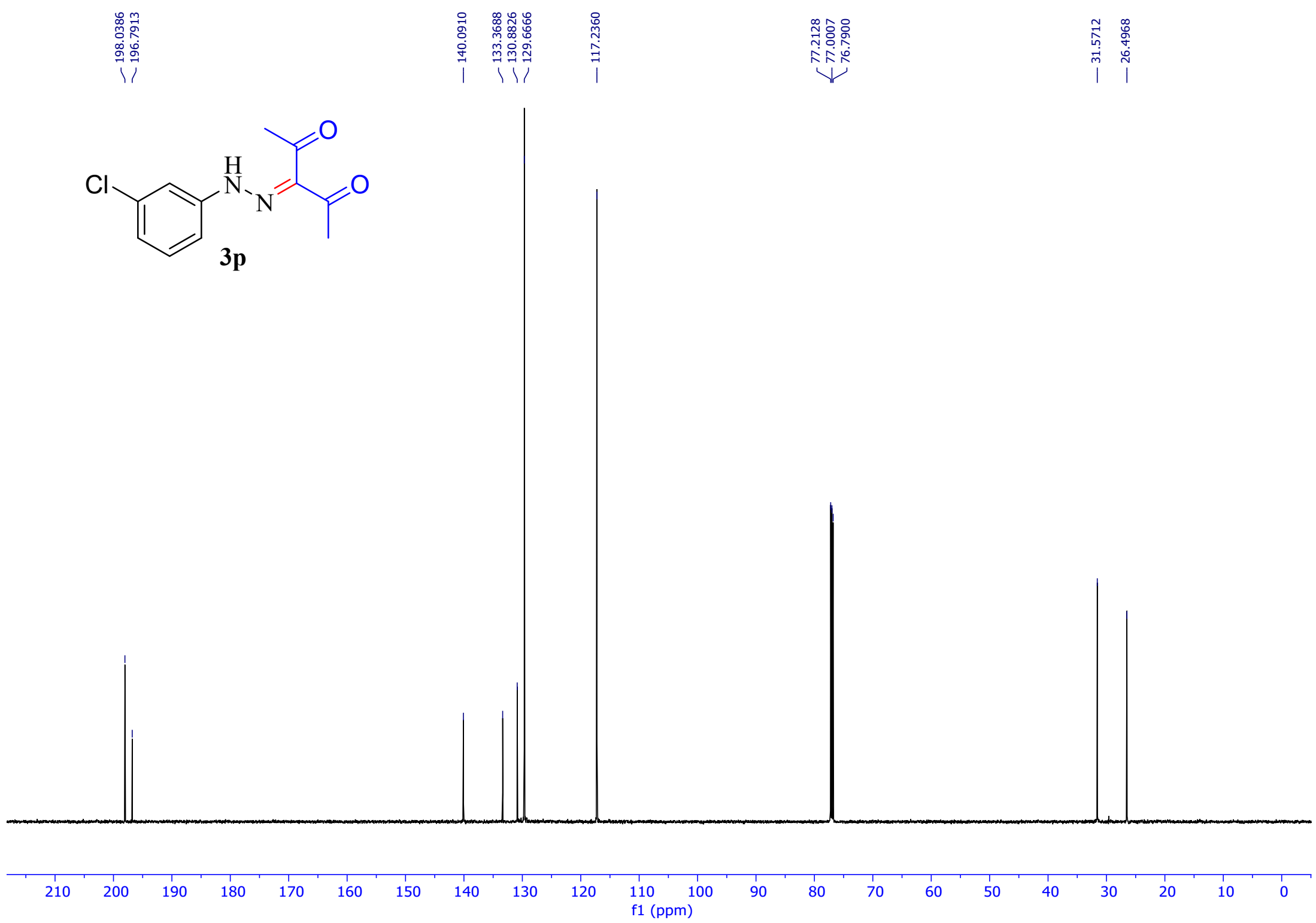
198.0386
196.7913
140.0910
133.3688
130.8826
129.6666
117.2360
77.2128
77.0007
76.7900
31.5712
26.4968
Cl
H
N
N
O
O
3p
210 200 190 180 170 160 150 140 130 120 110 100 90 80 70 60 50 40 30 20 10 0
f1 (ppm)

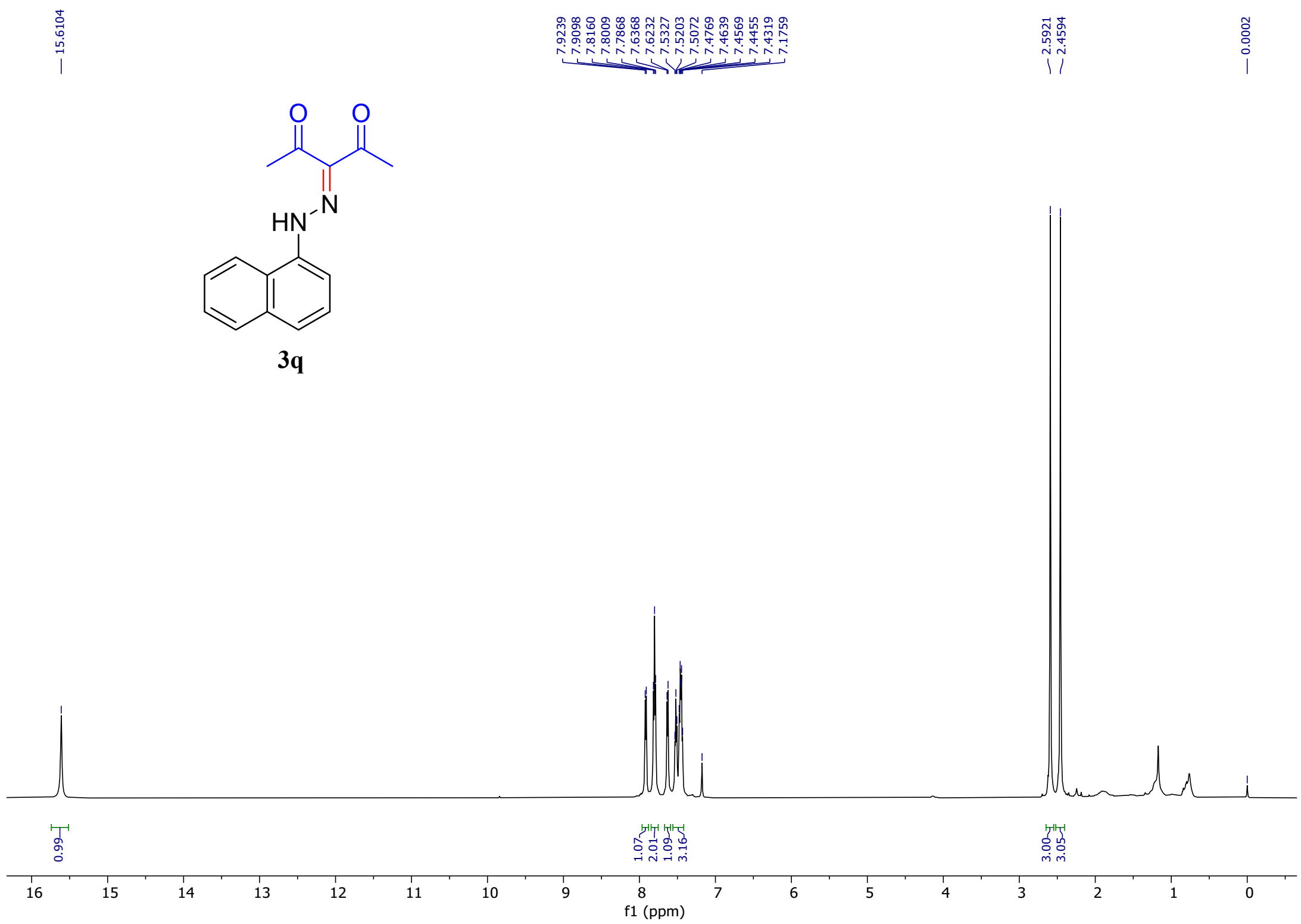
15.6104
7.9239
7.9098
7.8160
7.8009
7.7868
7.6368
7.6232
7.5327
7.5203
7.5072
7.4769
7.4639
7.4569
7.4455
7.4319
7.1759
2.5921
2.4594
0.0002
O
O
HN
N
3q
0.99
1.07
2.01
1.09
3.16
3.00
3.05
f1 (ppm)

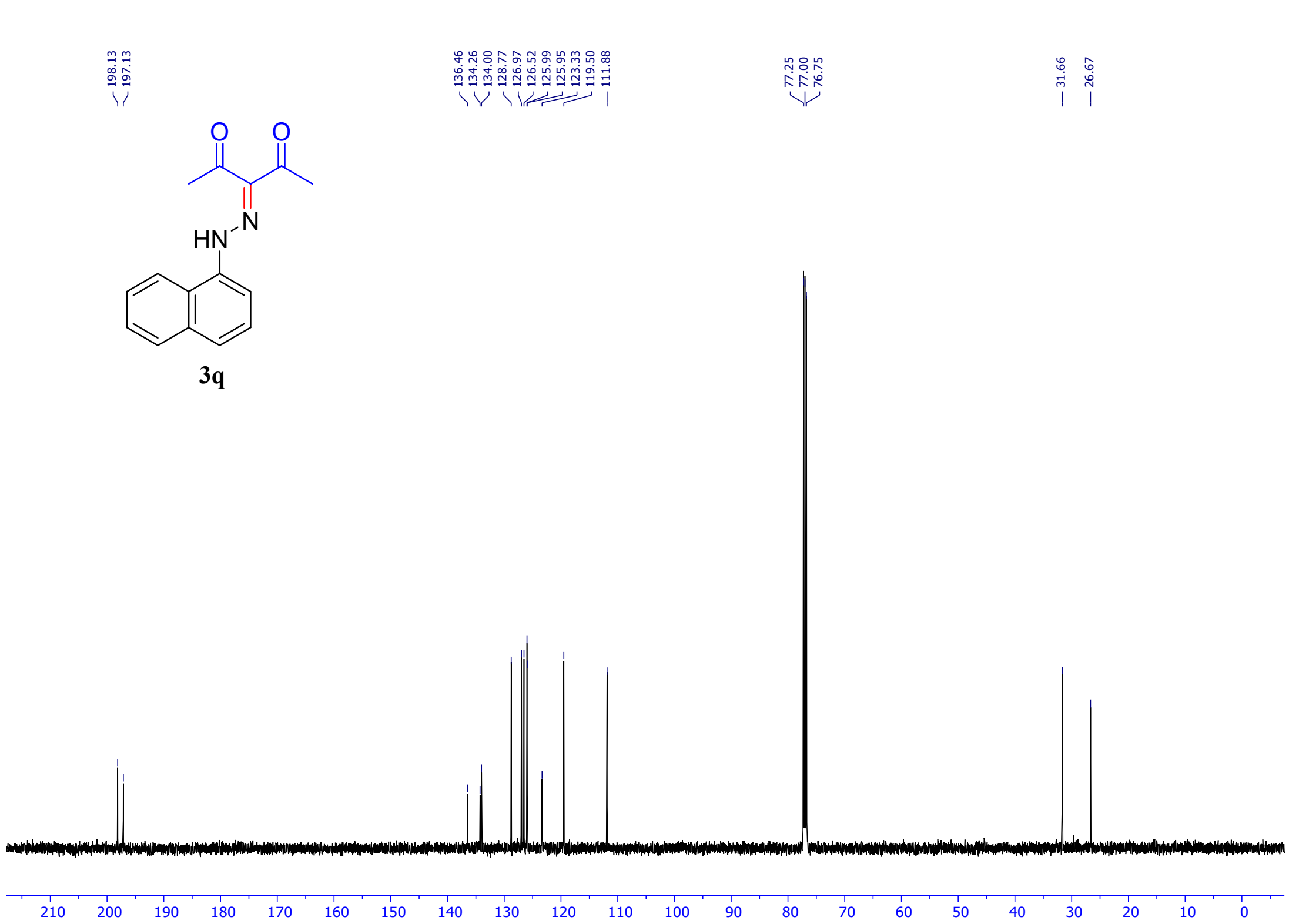
198.13
197.13
136.46
134.26
134.00
128.77
126.97
126.52
125.99
125.95
123.33
119.50
111.88
77.25
77.00
76.75
31.66
26.67
O
O
HN
N
3q
f1 (ppm)

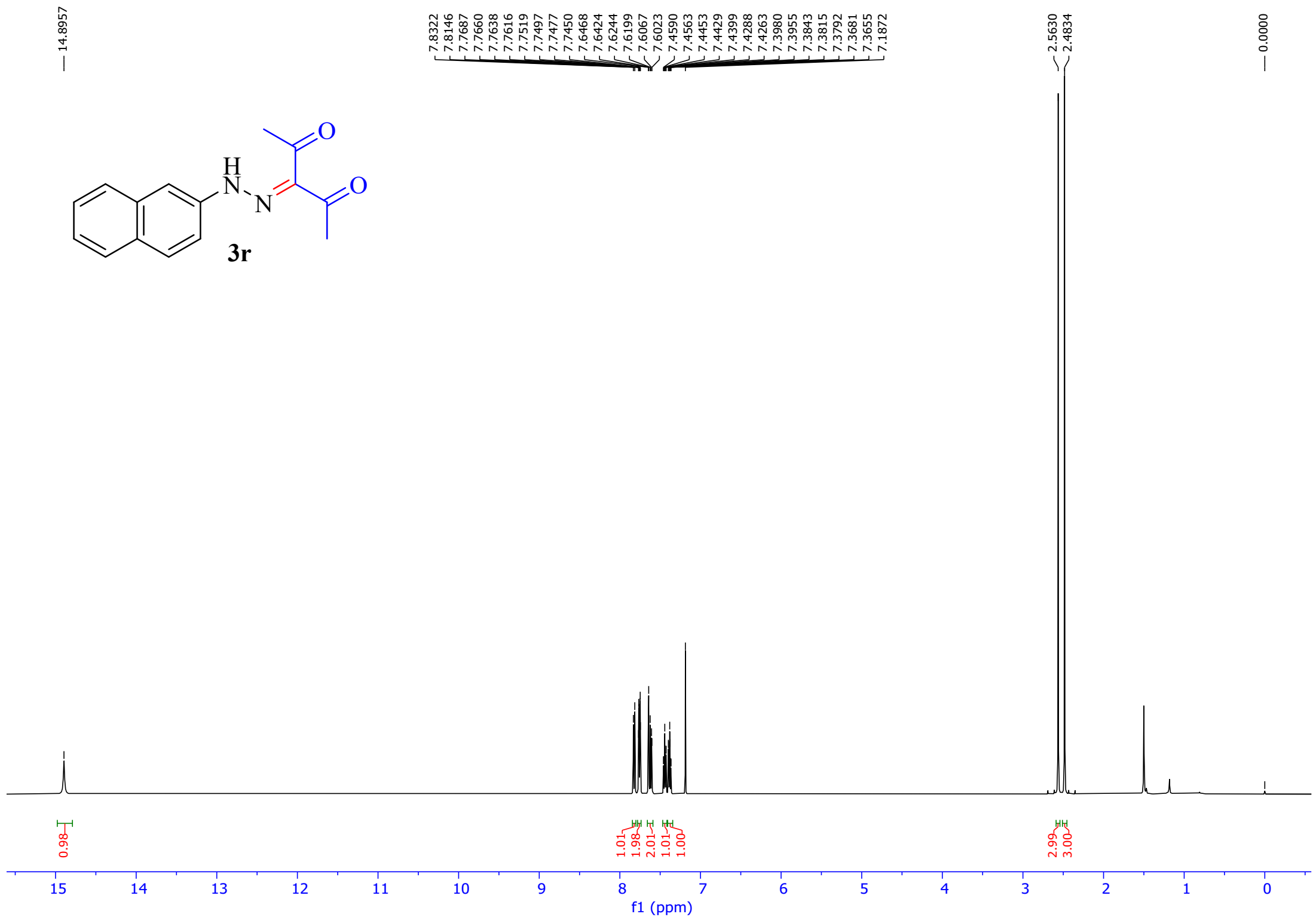

14.8957
7.8322 7.8146 7.7687 7.7660 7.7638 7.7616 7.7519 7.7497 7.7477 7.7450 7.6468 7.6424 7.6244 7.6199 7.6067 7.6023 7.4590 7.4563 7.4453 7.4429 7.4399 7.4288 7.4263 7.3980 7.3955 7.3843 7.3815 7.3792 7.3681 7.3655 7.1872
2.5630
2.4834
0.0000
3r
0.98
1.01 1.98 2.01 1.01 1.00
2.99 3.00
f1 (ppm)

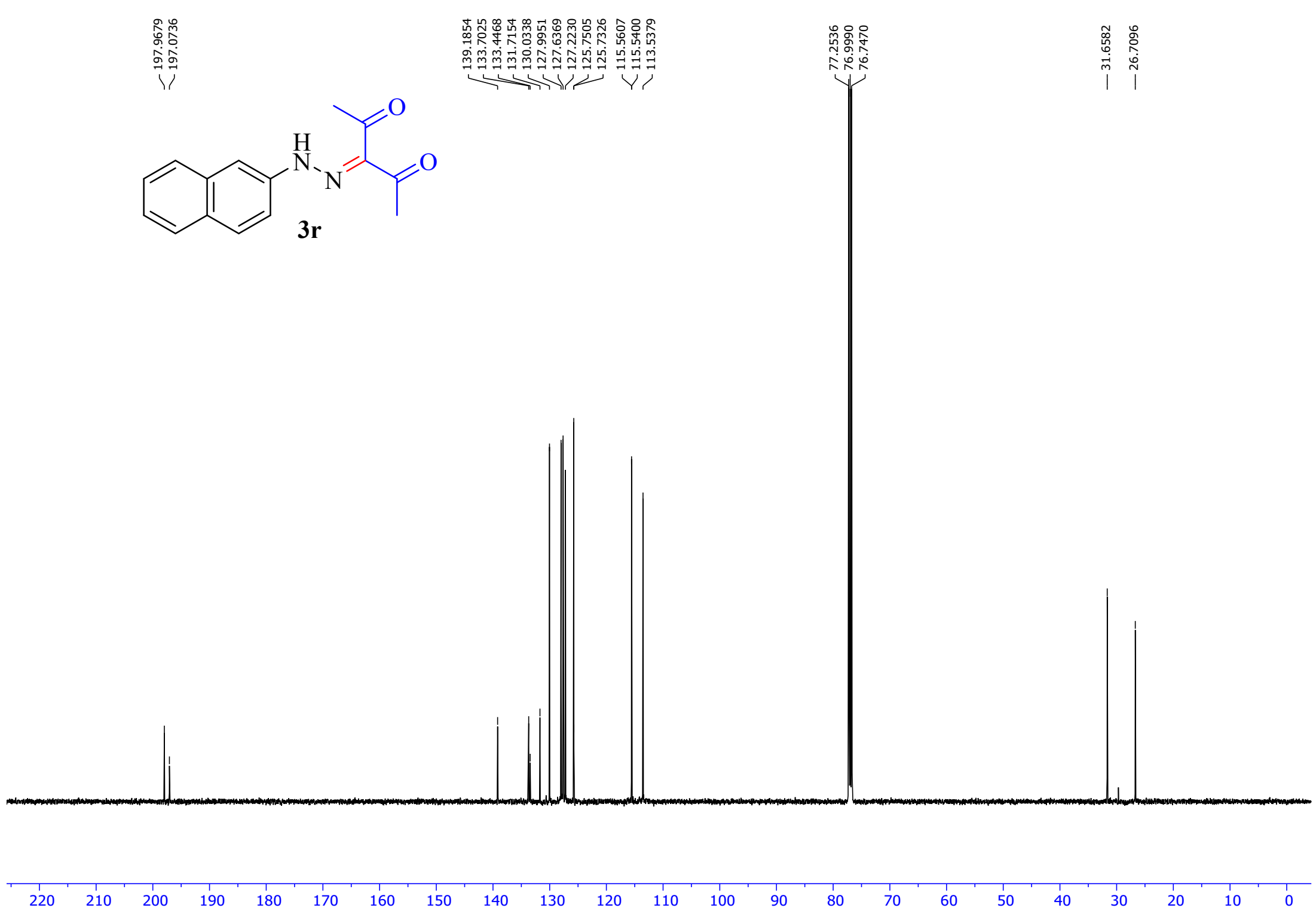

197.9679
197.0736
139.1854 133.7025 133.4468 131.7154 130.0338 127.9951 127.6369 127.2230 125.7505 125.7326 115.5607 115.5400 113.5379
77.2536 76.9990 76.7470
31.6582
26.7096
3r
f1 (ppm)

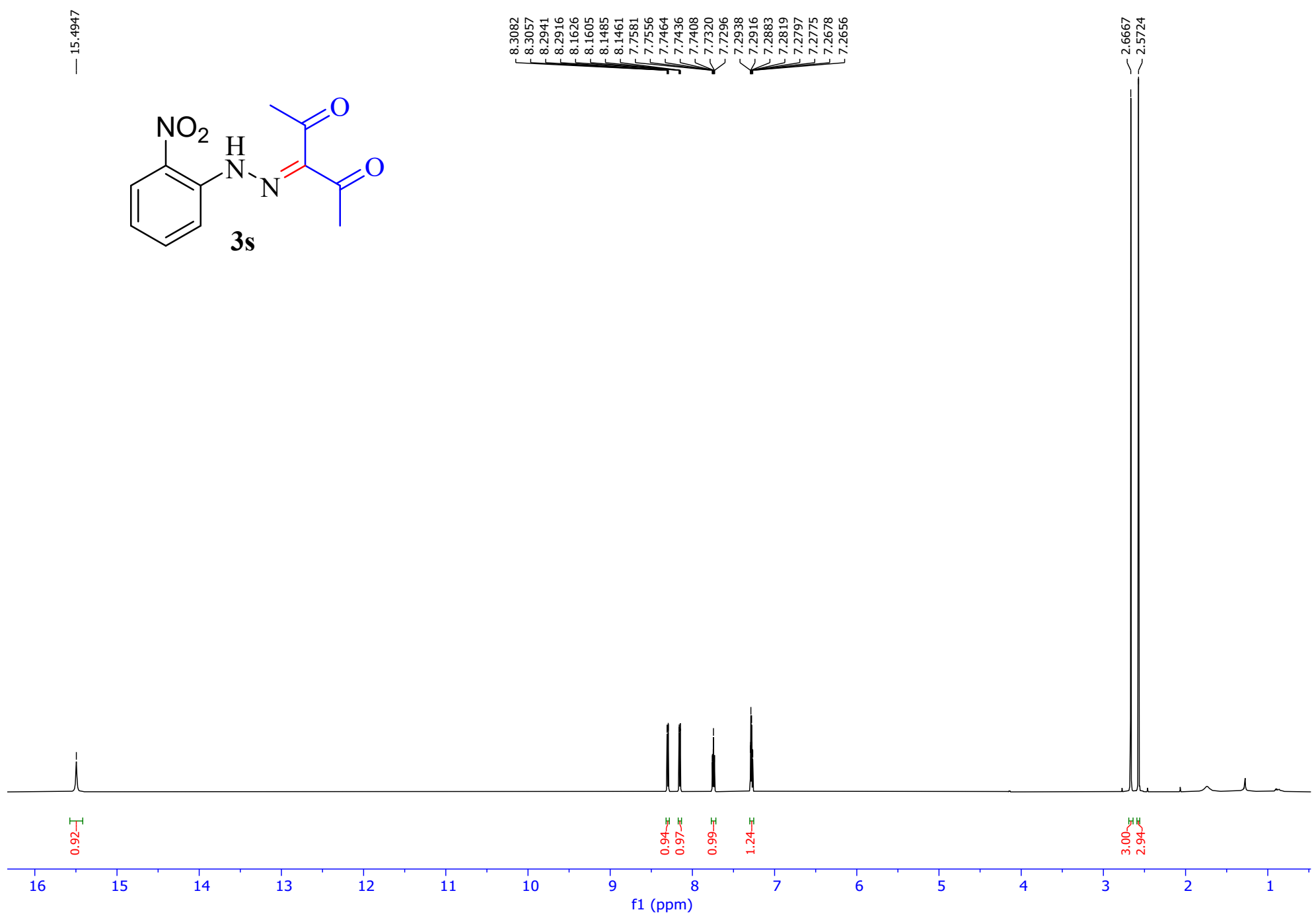
15.4947
8.3082
8.3057
8.2941
8.2916
8.1626
8.1605
8.1485
8.1461
7.7581
7.7556
7.7464
7.7436
7.7408
7.7320
7.7296
7.2938
7.2916
7.2883
7.2819
7.2797
7.2775
7.2678
7.2656
2.6667
2.5724
NO2
H
N
N
O
O
3s
0.92
0.94
0.97
0.99
1.24
3.00
2.94
16 15 14 13 12 11 10 9 8 7 6 5 4 3 2 1
f1 (ppm)

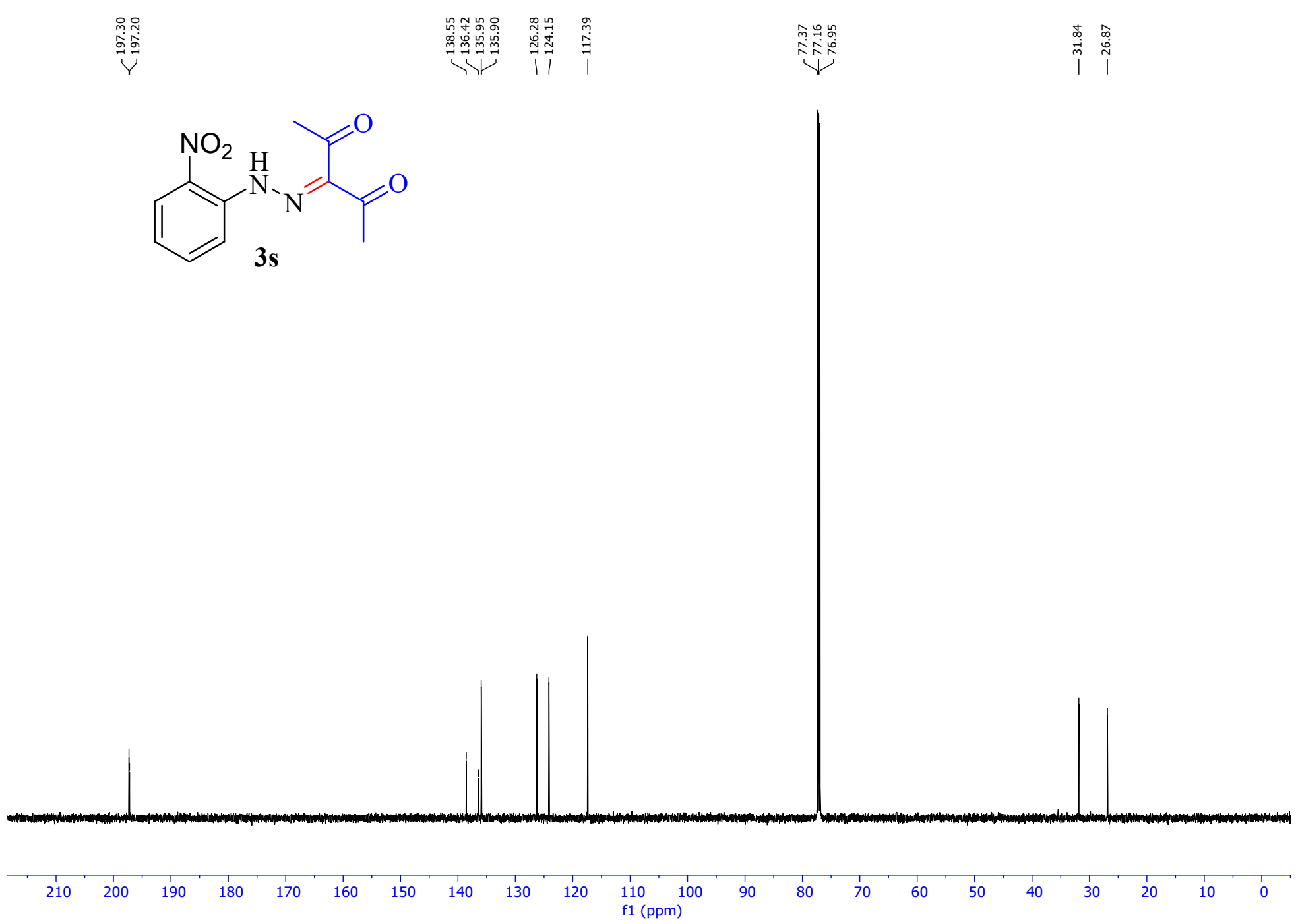
197.30
197.20
138.55
136.42
135.95
135.90
126.28
124.15
117.39
77.37
77.16
76.95
31.84
26.87
NO2
H
N
N
O
O
3s
210 200 190 180 170 160 150 140 130 120 110 100 90 80 70 60 50 40 30 20 10 0
f1 (ppm)

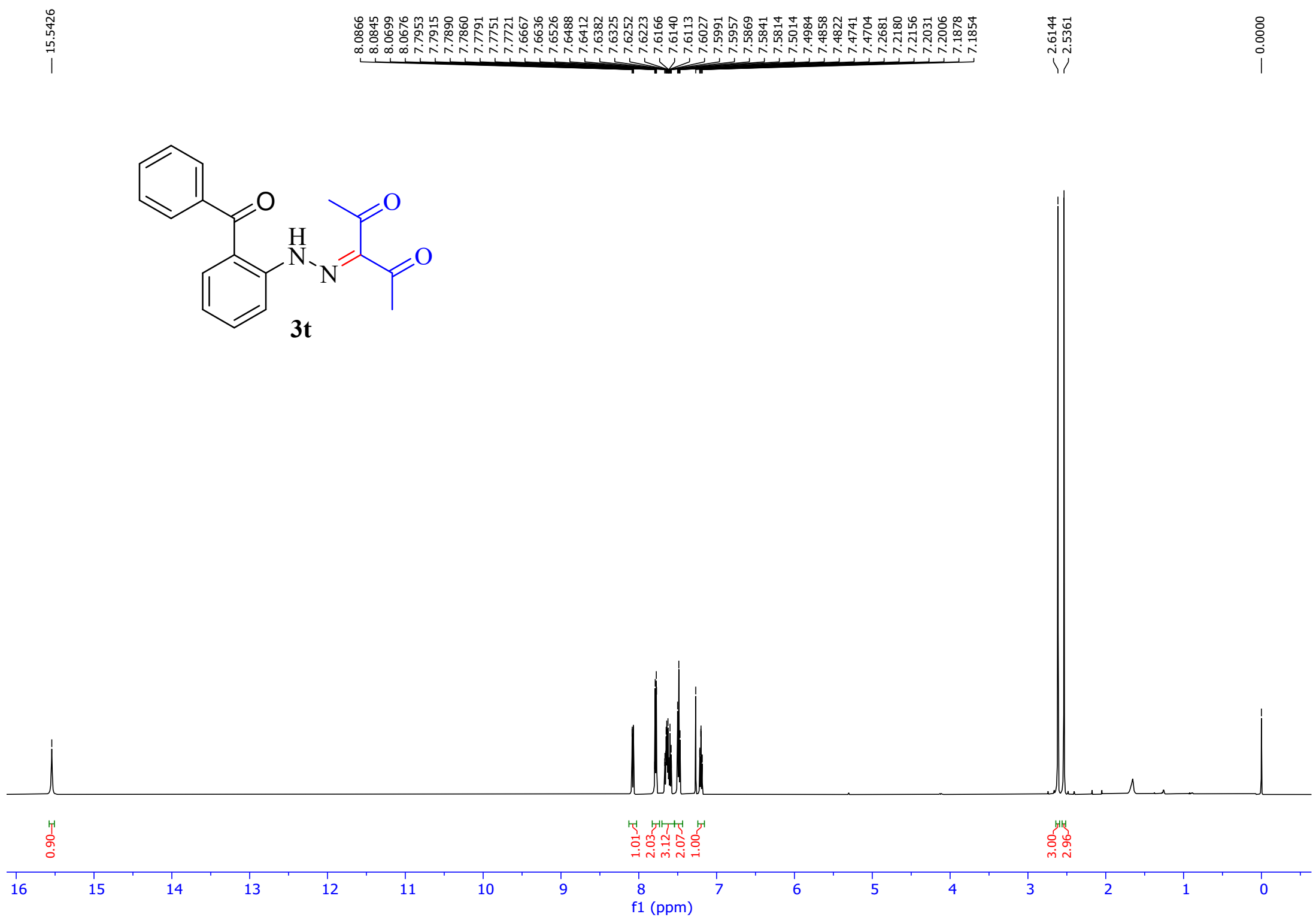

15.5426
8.0866 8.0845 8.0699 8.0676 7.7953 7.7915 7.7890 7.7860 7.7791 7.7751 7.7721 7.6667 7.6636 7.6526 7.6488 7.6412 7.6382 7.6325 7.6252 7.6223 7.6166 7.6140 7.6113 7.6027 7.5991 7.5957 7.5869 7.5841 7.5814 7.5014 7.4984 7.4858 7.4822 7.4741 7.4704 7.2681 7.2180 7.2156 7.2031 7.2006 7.1878 7.1854
2.6144 2.5361
0.0000
O
H
N
N
O
O
3t
0.90
1.01 2.03 3.12 2.07 1.00
3.00 2.96
f1 (ppm)

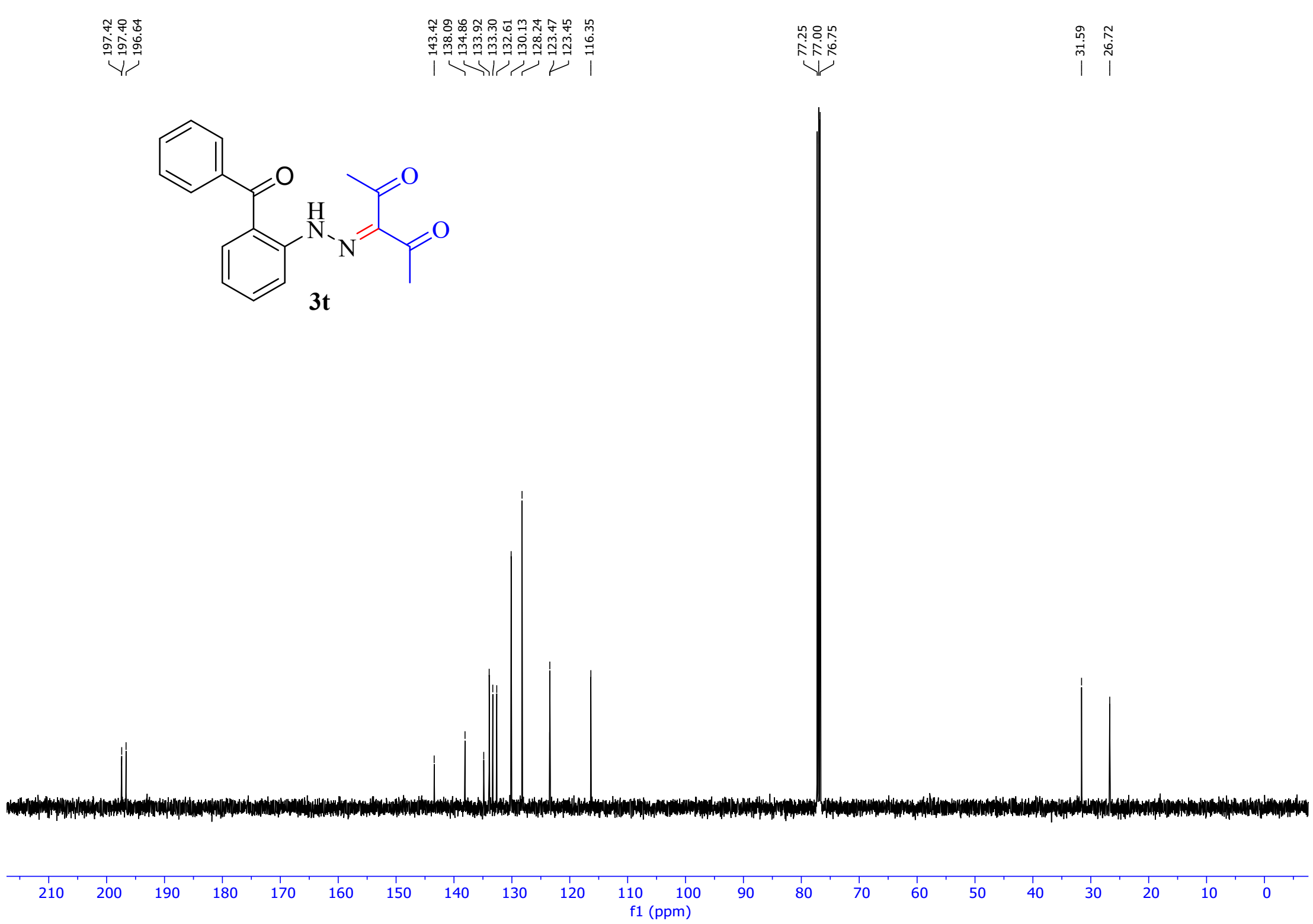

197.42 197.40 196.64
143.42 138.09 134.86 133.92 133.30 132.61 130.13 128.24 123.47 123.45 116.35
77.25 77.00 76.75
31.59 26.72
O
H
N
N
O
O
3t
f1 (ppm)

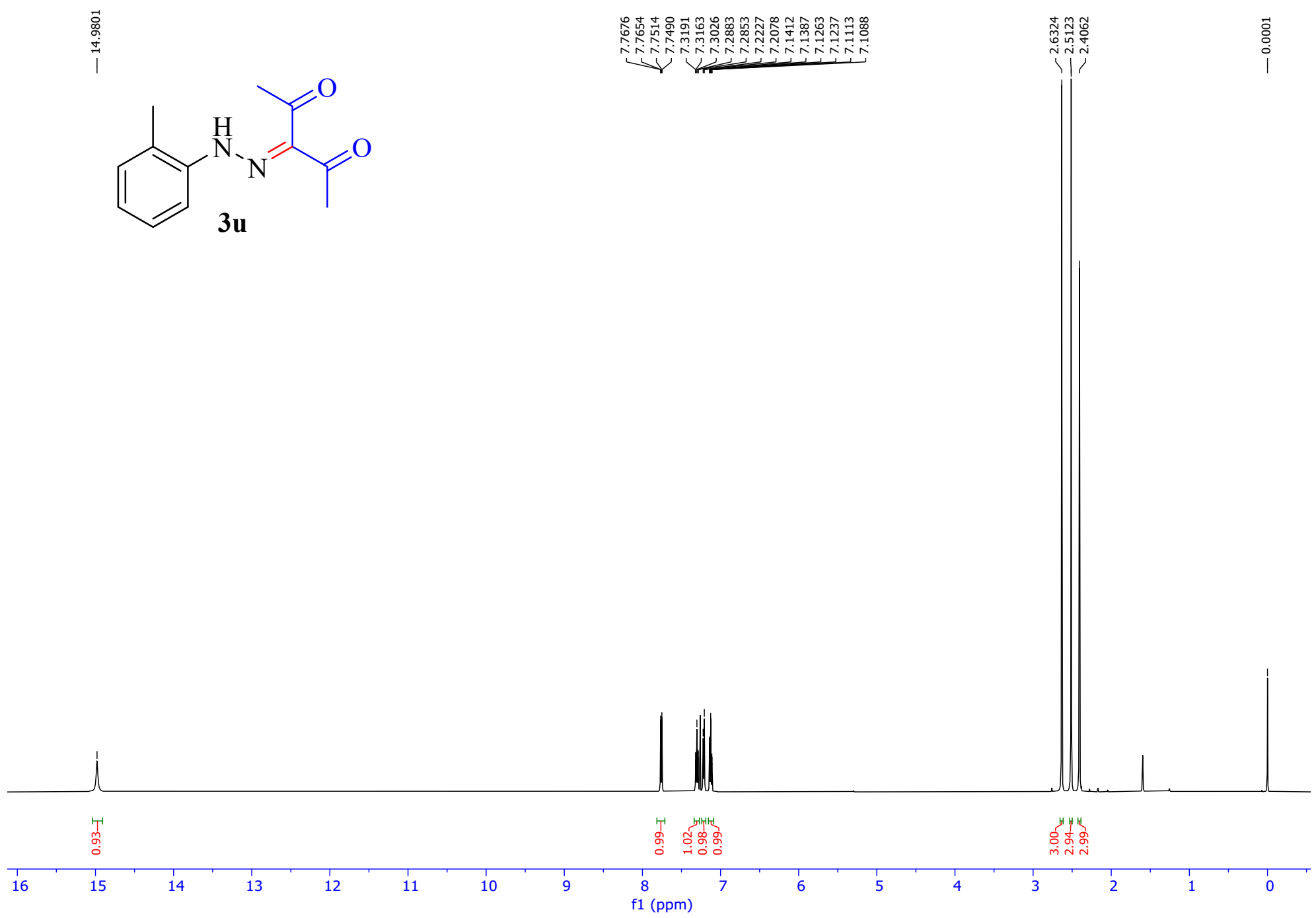
14.9801
7.7676
7.7654
7.7514
7.7490
7.3191
7.3163
7.3026
7.2883
7.2853
7.2227
7.2078
7.1412
7.1387
7.1263
7.1237
7.1113
7.1088
2.6324
2.5123
2.4062
0.0001
H
N
N
O
O
3u
0.93
0.99
1.02
0.98
0.99
3.00
2.94
2.99
16 15 14 13 12 11 10 9 8 7 6 5 4 3 2 1 0
f1 (ppm)

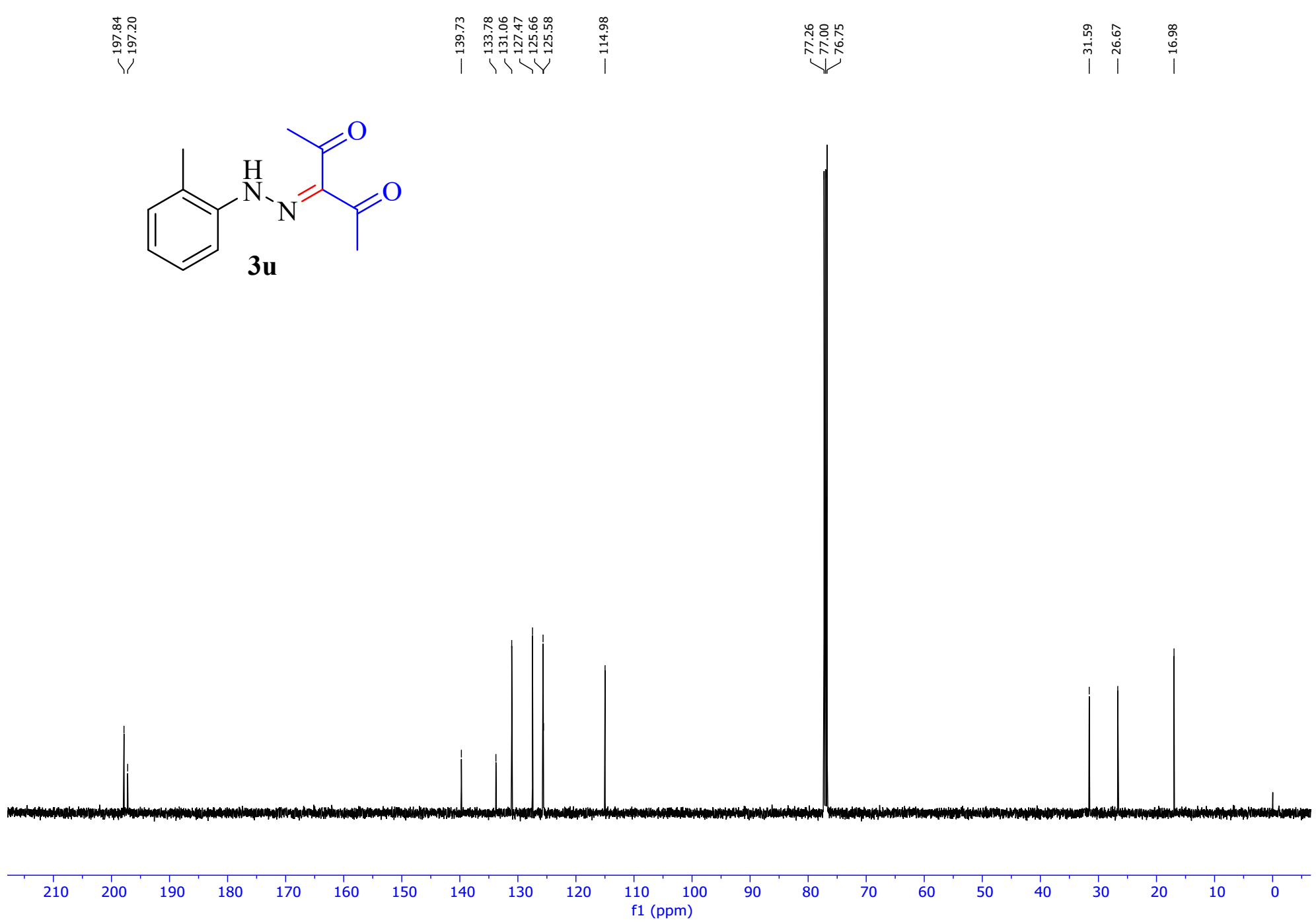
197.84
197.20
139.73
133.78
131.06
127.47
125.66
125.58
114.98
77.26
77.00
76.75
31.59
26.67
16.98
H
N
N
O
O
3u
210 200 190 180 170 160 150 140 130 120 110 100 90 80 70 60 50 40 30 20 10 0
f1 (ppm)

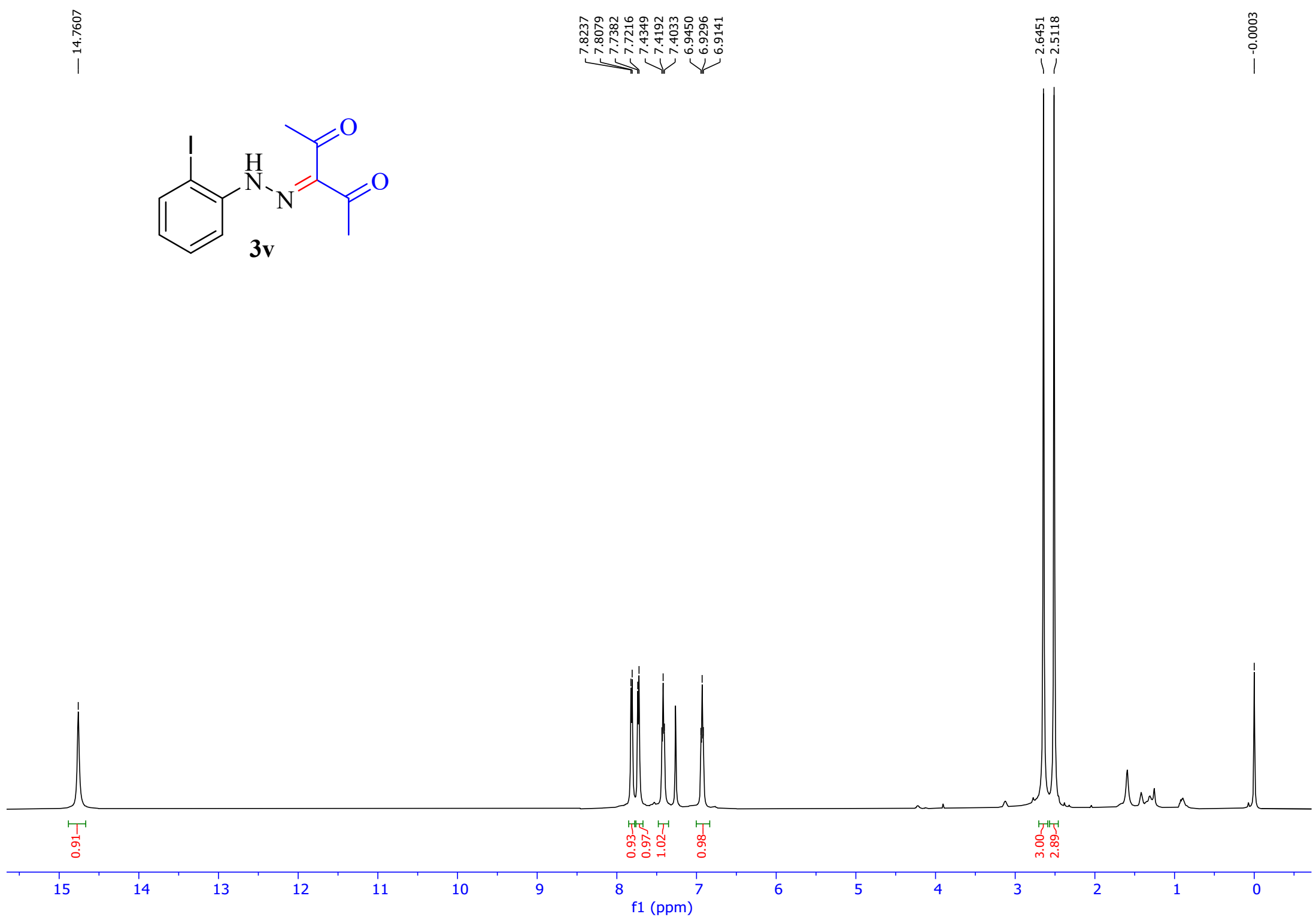
14.7607
7.8237
7.8079
7.7382
7.7216
7.4349
7.4192
7.4033
6.9450
6.9296
6.9141
2.6451
2.5118
-0.0003
0.91
0.93
0.97
1.02
0.98
3.00
2.89
f1 (ppm)
3v

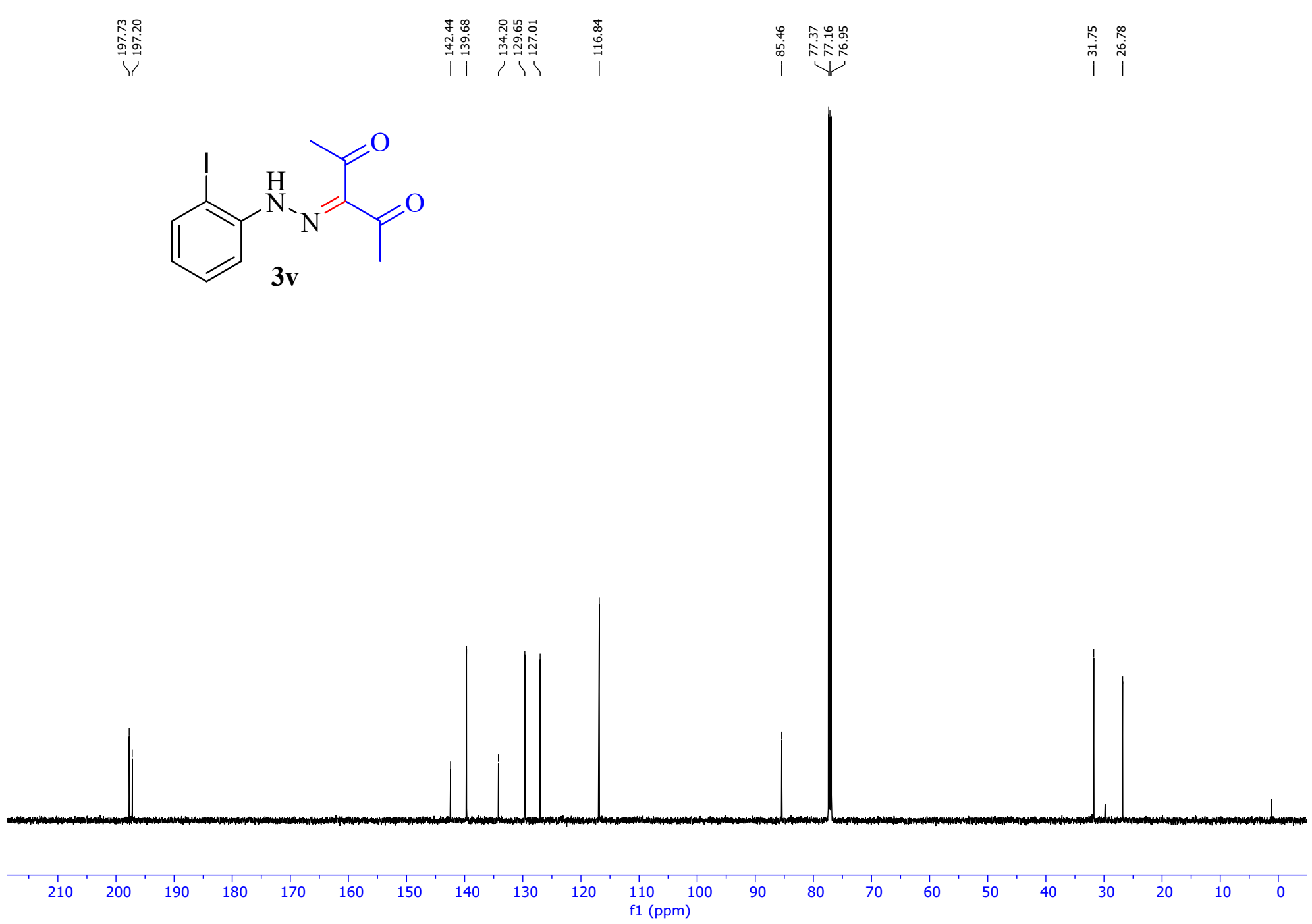
197.73
197.20
142.44
139.68
134.20
129.65
127.01
116.84
85.46
77.37
77.16
76.95
31.75
26.78
f1 (ppm)
3v

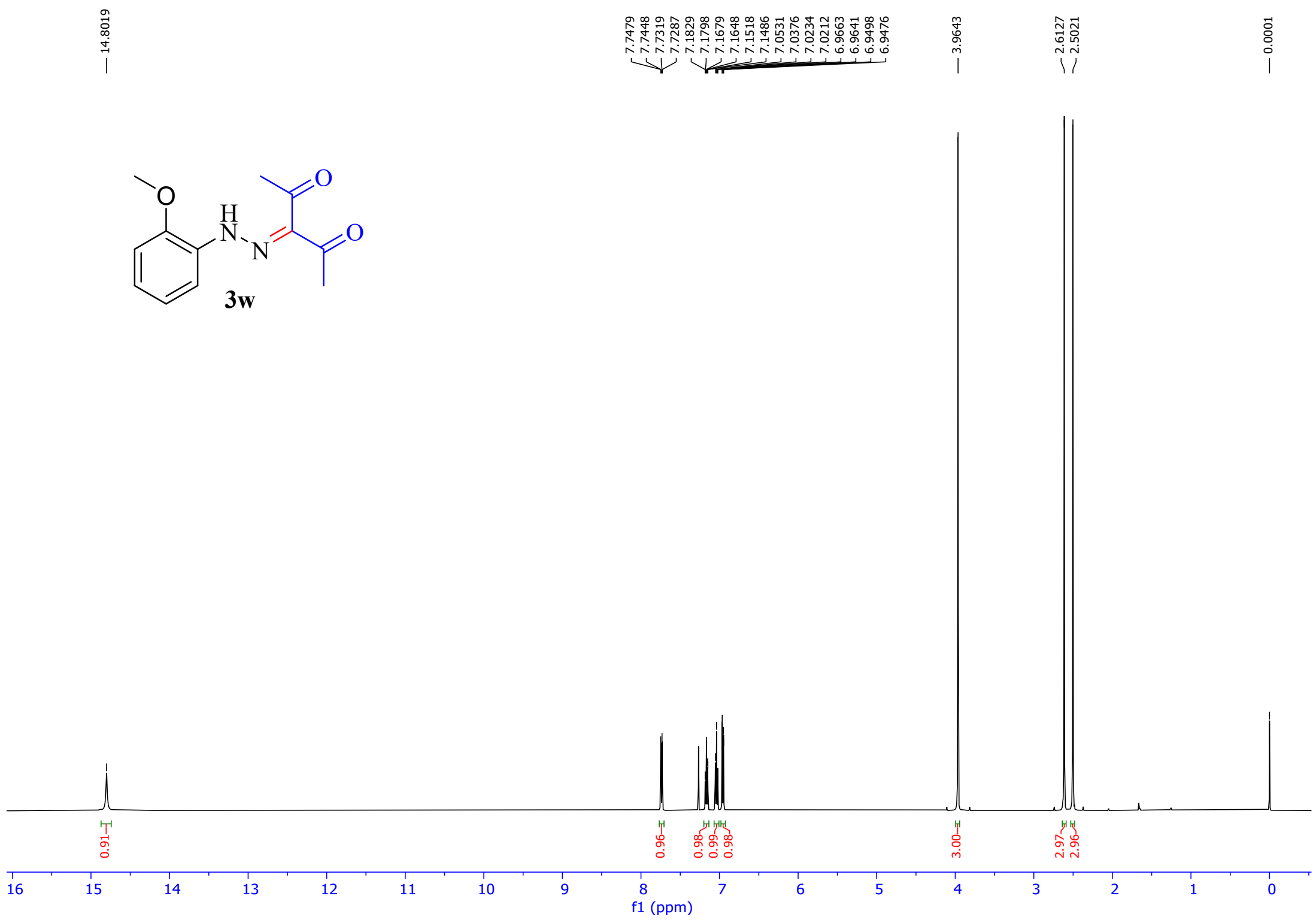
14.8019
7.7479
7.7448
7.7319
7.7287
7.1829
7.1798
7.1679
7.1648
7.1518
7.1486
7.0531
7.0376
7.0234
7.0212
6.9663
6.9641
6.9498
6.9476
3.9643
2.6127
2.5021
0.0001
H
N
N
O
O
O
3w
0.91
0.96
0.98
0.99
0.98
3.00
2.97
2.96
f1 (ppm)

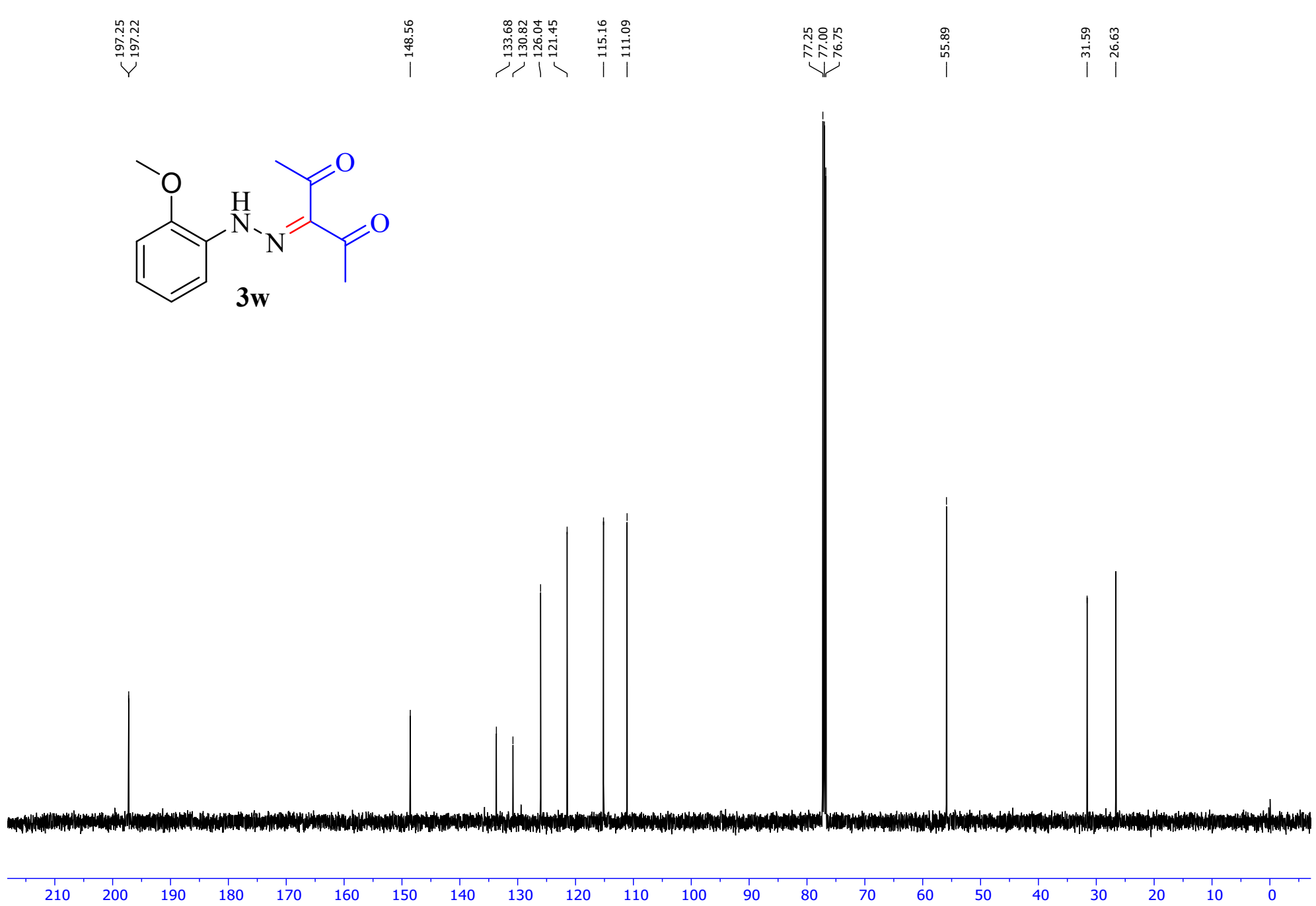
197.25
197.22
148.56
133.68
130.82
126.04
121.45
115.16
111.09
77.25
77.00
76.75
55.89
31.59
26.63
H
N
N
O
O
O
3w
f1 (ppm)

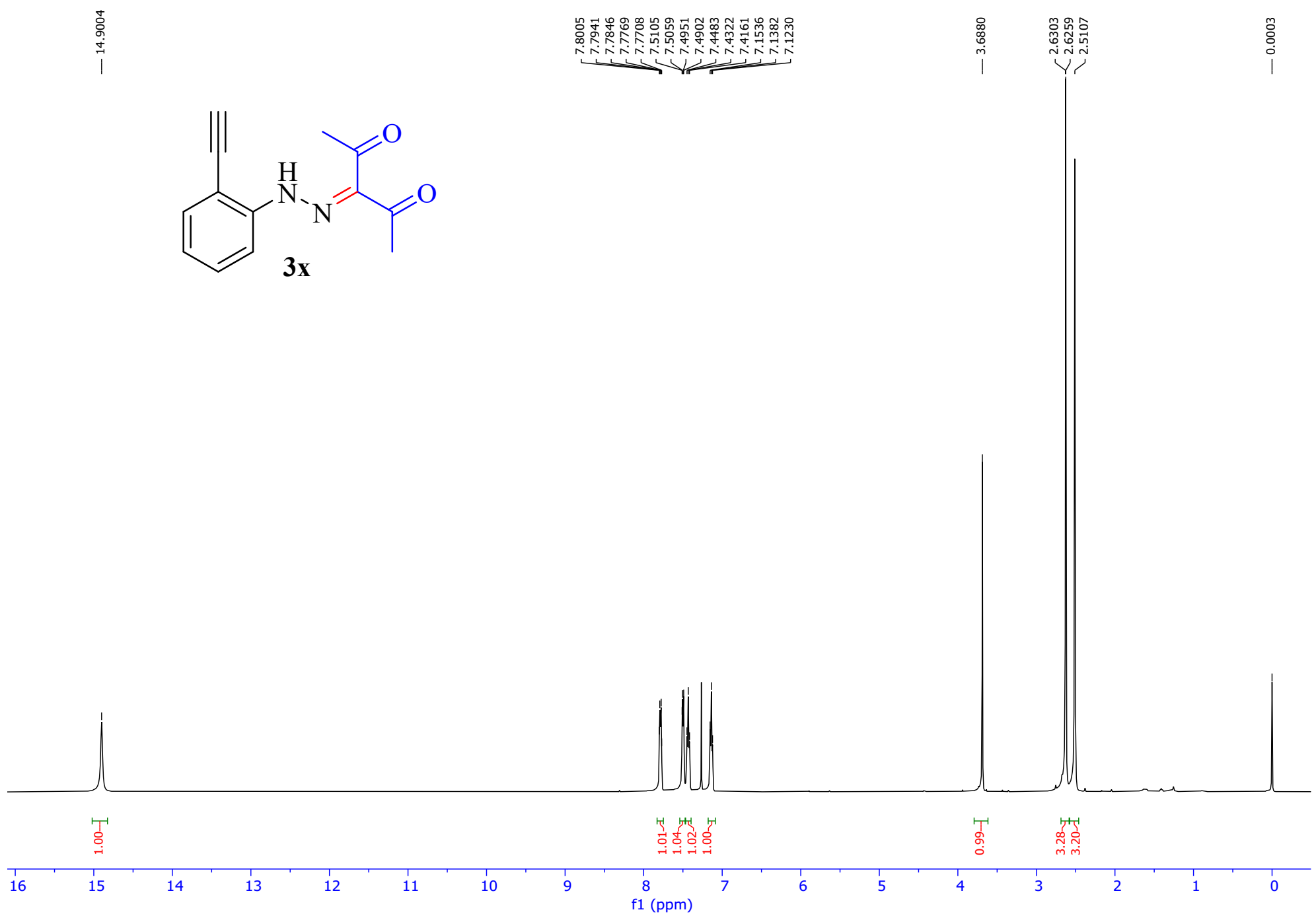
14.9004
7.8005
7.7941
7.7846
7.7769
7.7708
7.5105
7.5059
7.4951
7.4902
7.4483
7.4322
7.4161
7.1536
7.1382
7.1230
3.6880
2.6303
2.6259
2.5107
0.0003
H
N
N
O
O
3x
1.00
1.01
1.04
1.02
1.00
0.99
3.28
3.20
f1 (ppm)

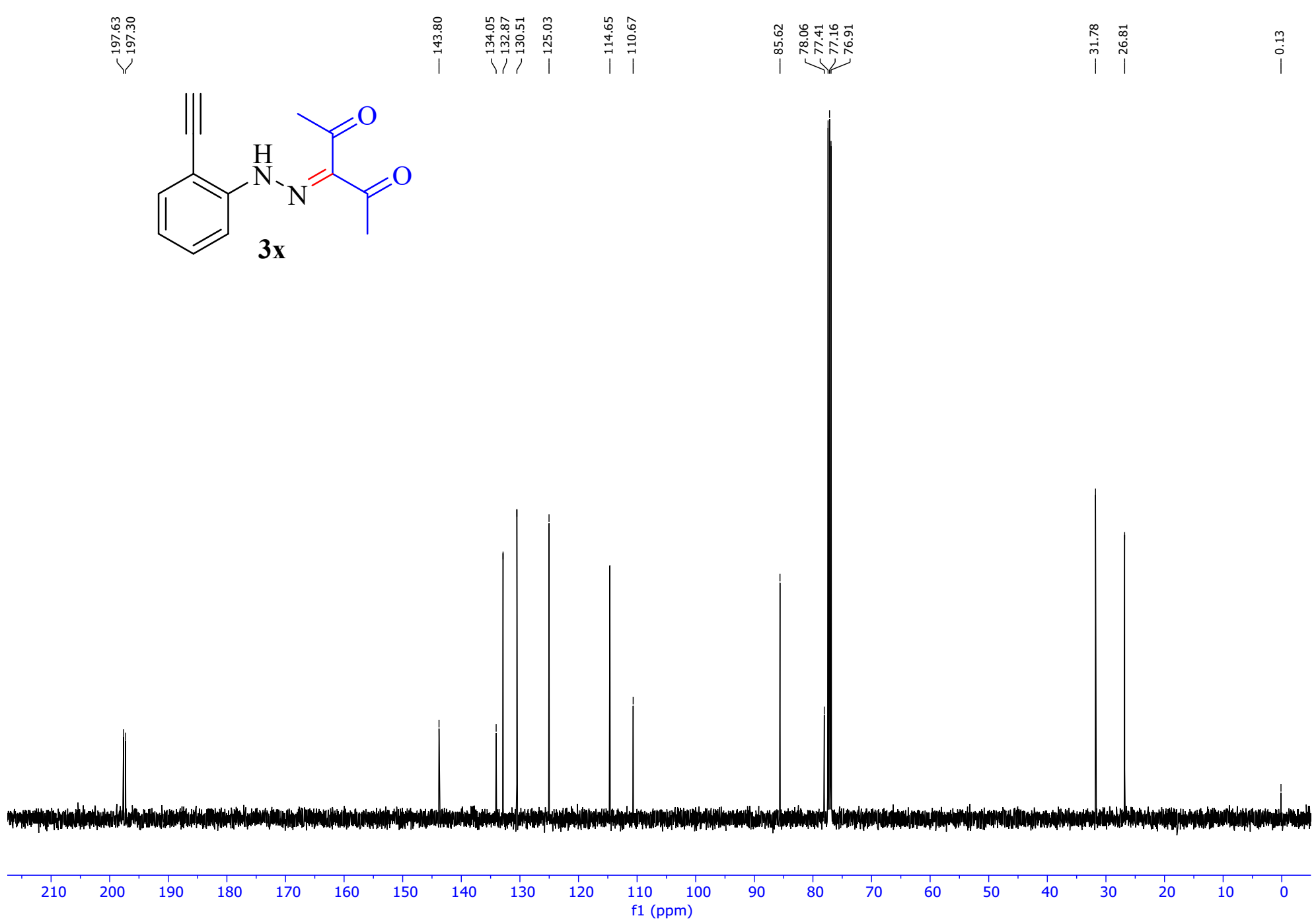
197.63
197.30
143.80
134.05
132.87
130.51
125.03
114.65
110.67
85.62
78.06
77.41
77.16
76.91
31.78
26.81
0.13
H
N
N
O
O
3x
f1 (ppm)

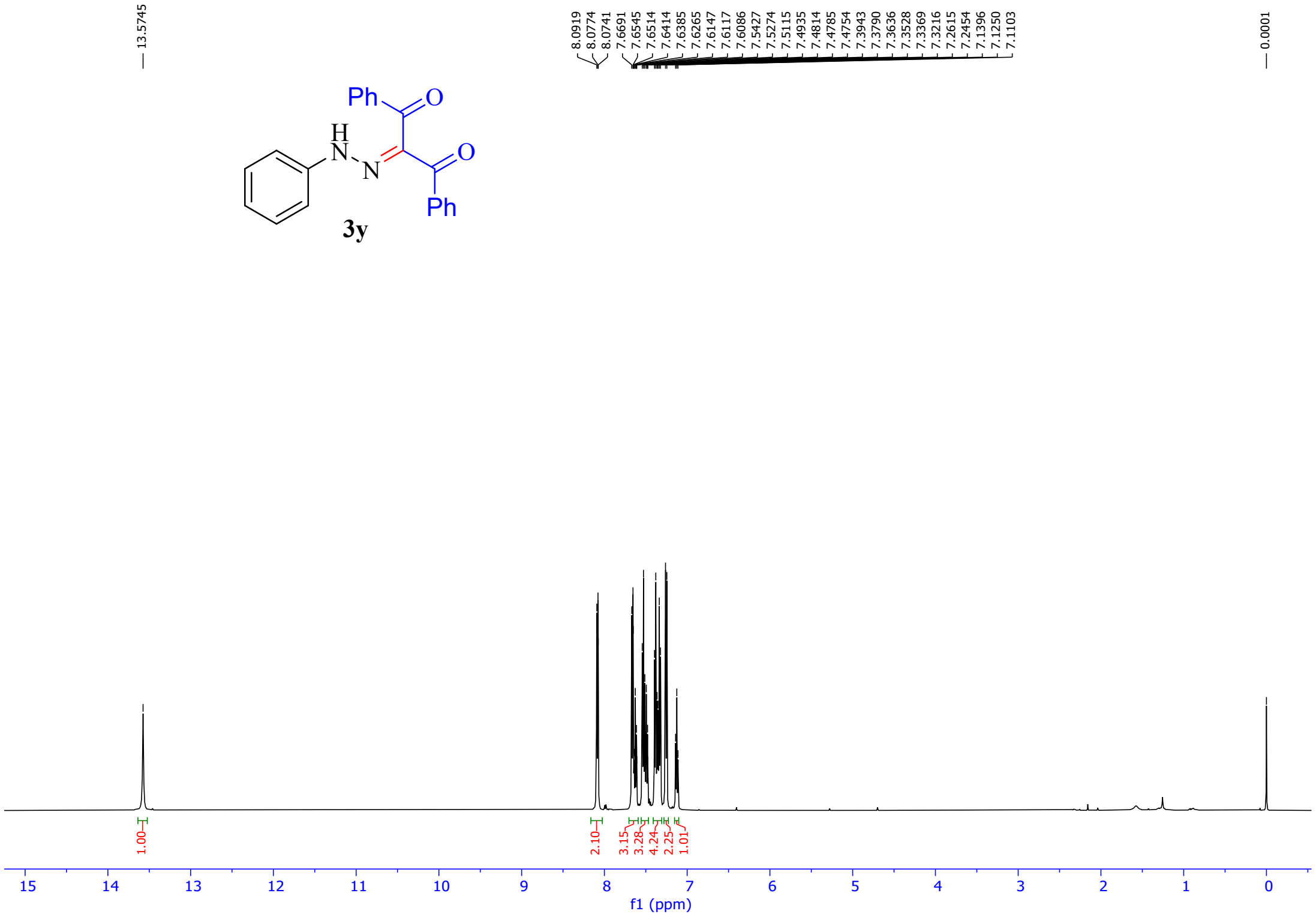
13.5745
8.0919
8.0774
8.0741
7.6691
7.6545
7.6514
7.6414
7.6385
7.6265
7.6147
7.6117
7.6086
7.5427
7.5274
7.5115
7.4935
7.4814
7.4785
7.4754
7.3943
7.3790
7.3636
7.3528
7.3369
7.3216
7.2615
7.2454
7.1396
7.1250
7.1103
0.0001
Ph
O
H
N
N
O
Ph
3y
1.00
2.10
3.15
3.28
4.24
2.25
1.01
f1 (ppm)

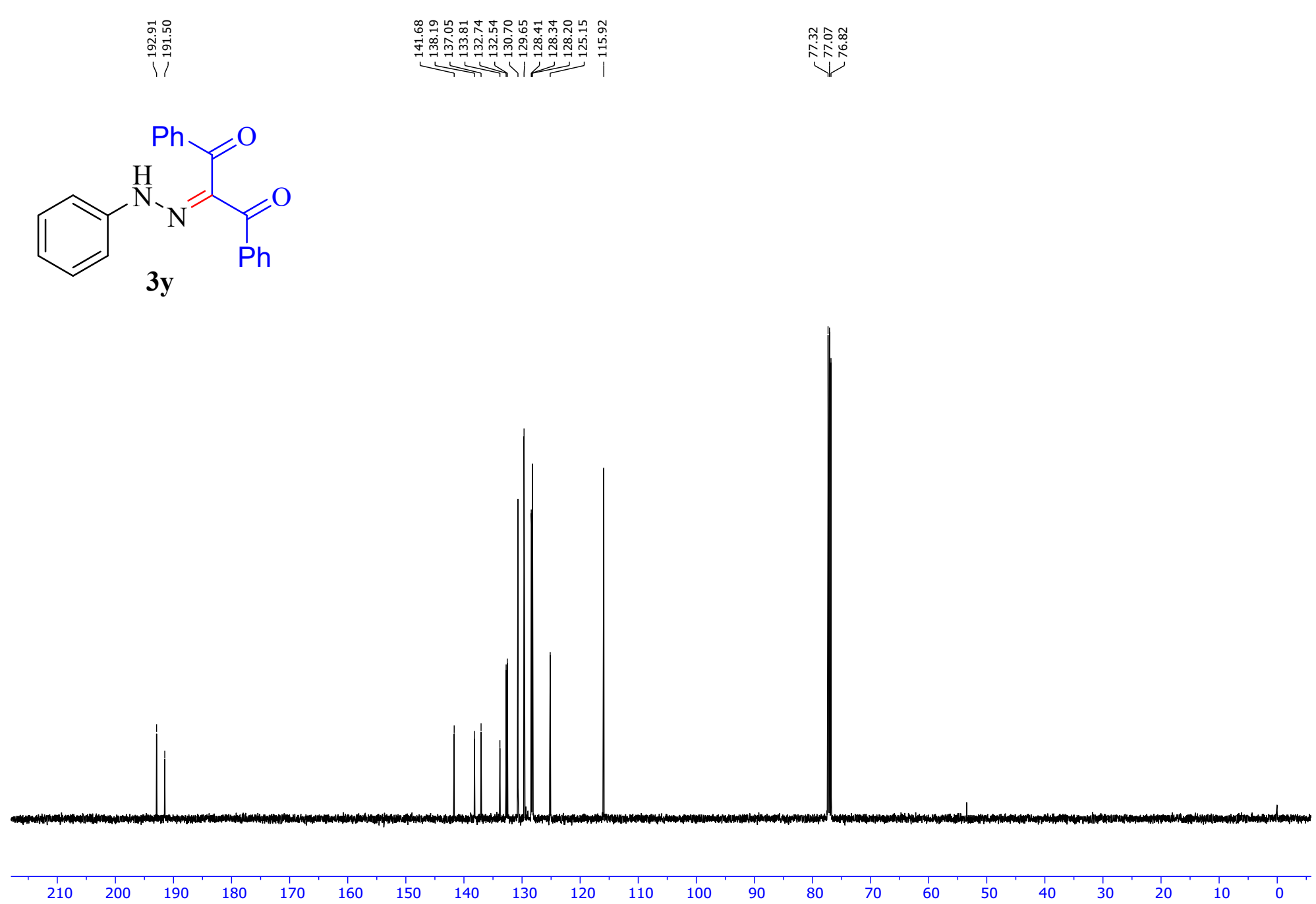
192.91
191.50
141.68
138.19
137.05
133.81
132.74
132.54
130.70
129.65
128.41
128.34
128.20
125.15
115.92
77.32
77.07
76.82
Ph
O
H
N
N
O
Ph
3y
f1 (ppm)

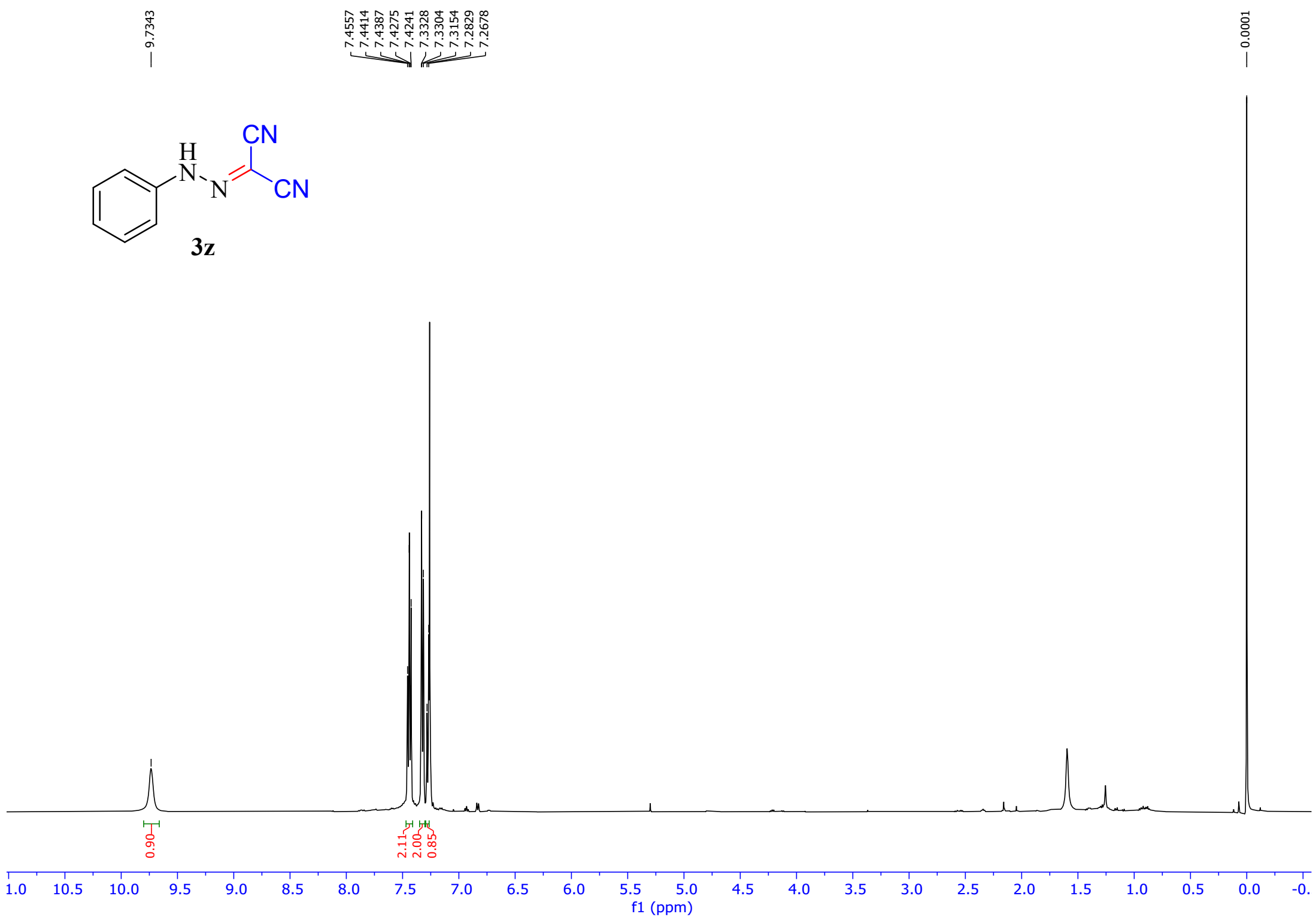
9.7343
7.4557
7.4414
7.4387
7.4275
7.4241
7.3328
7.3304
7.3154
7.2829
7.2678
0.0001
H
N
N
CN
CN
3z
0.90
2.11
2.00
0.85
f1 (ppm)

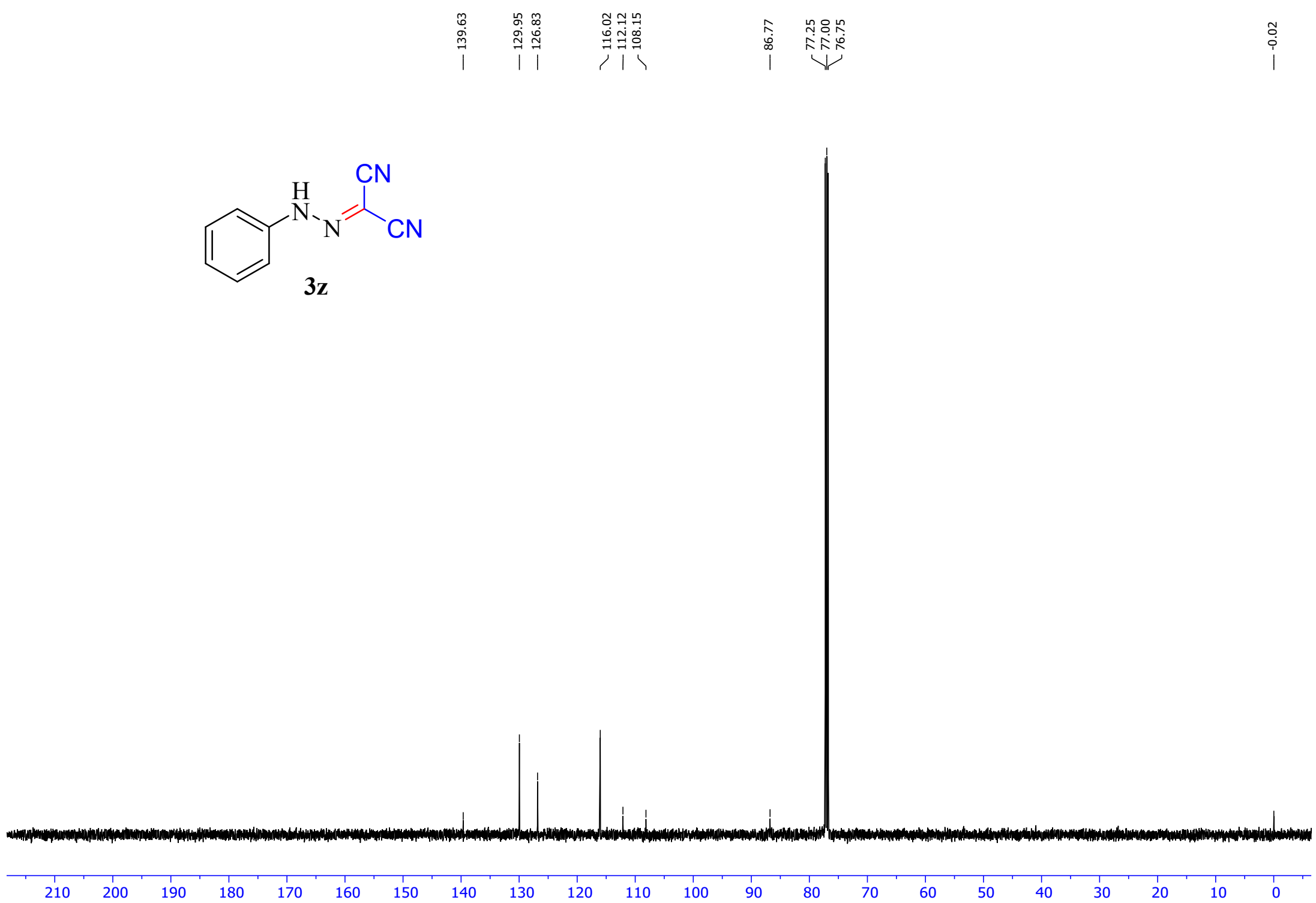
139.63
129.95
126.83
116.02
112.12
108.15
86.77
77.25
77.00
76.75
-0.02
H
N
N
CN
CN
3z
f1 (ppm)

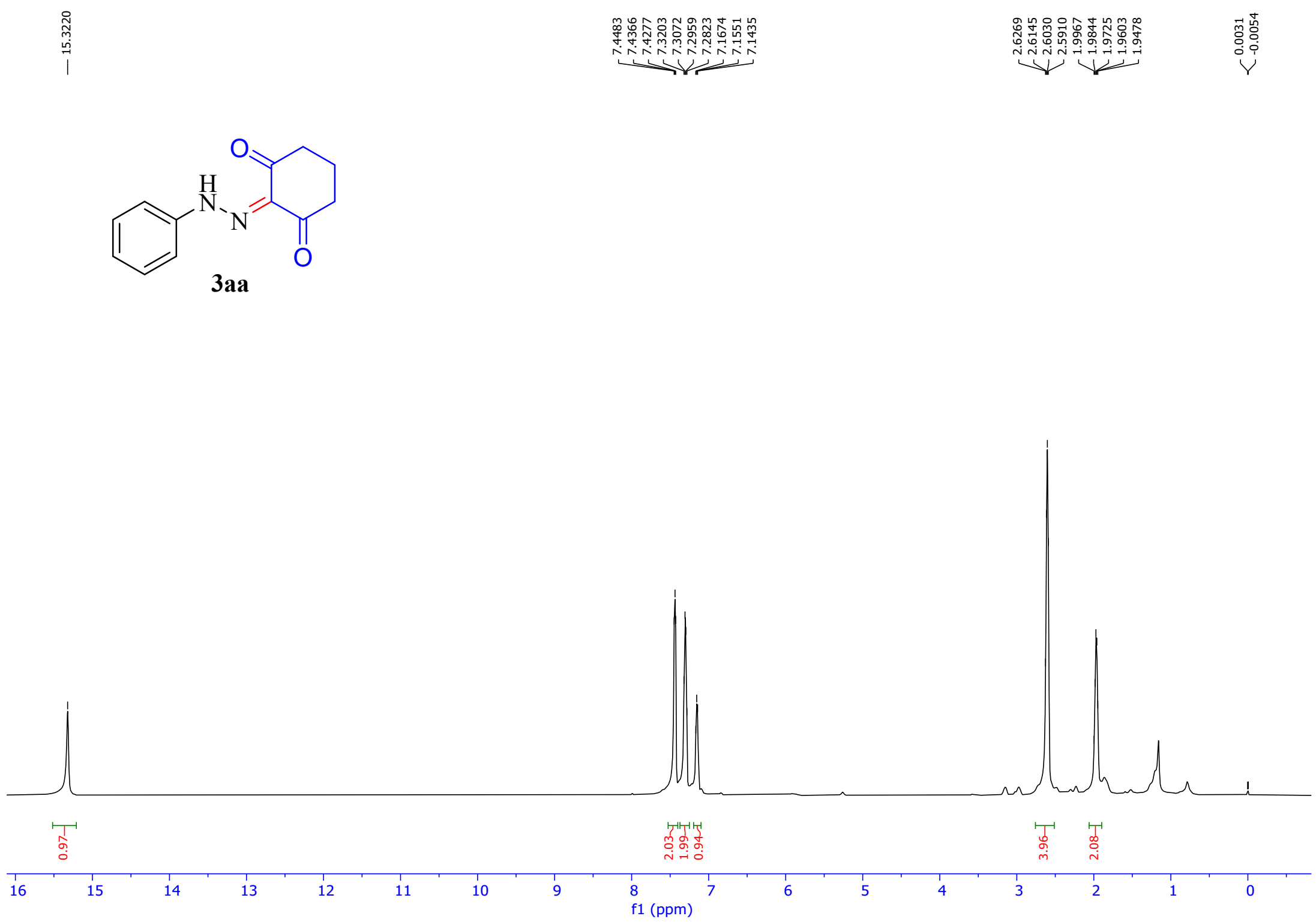

3aa

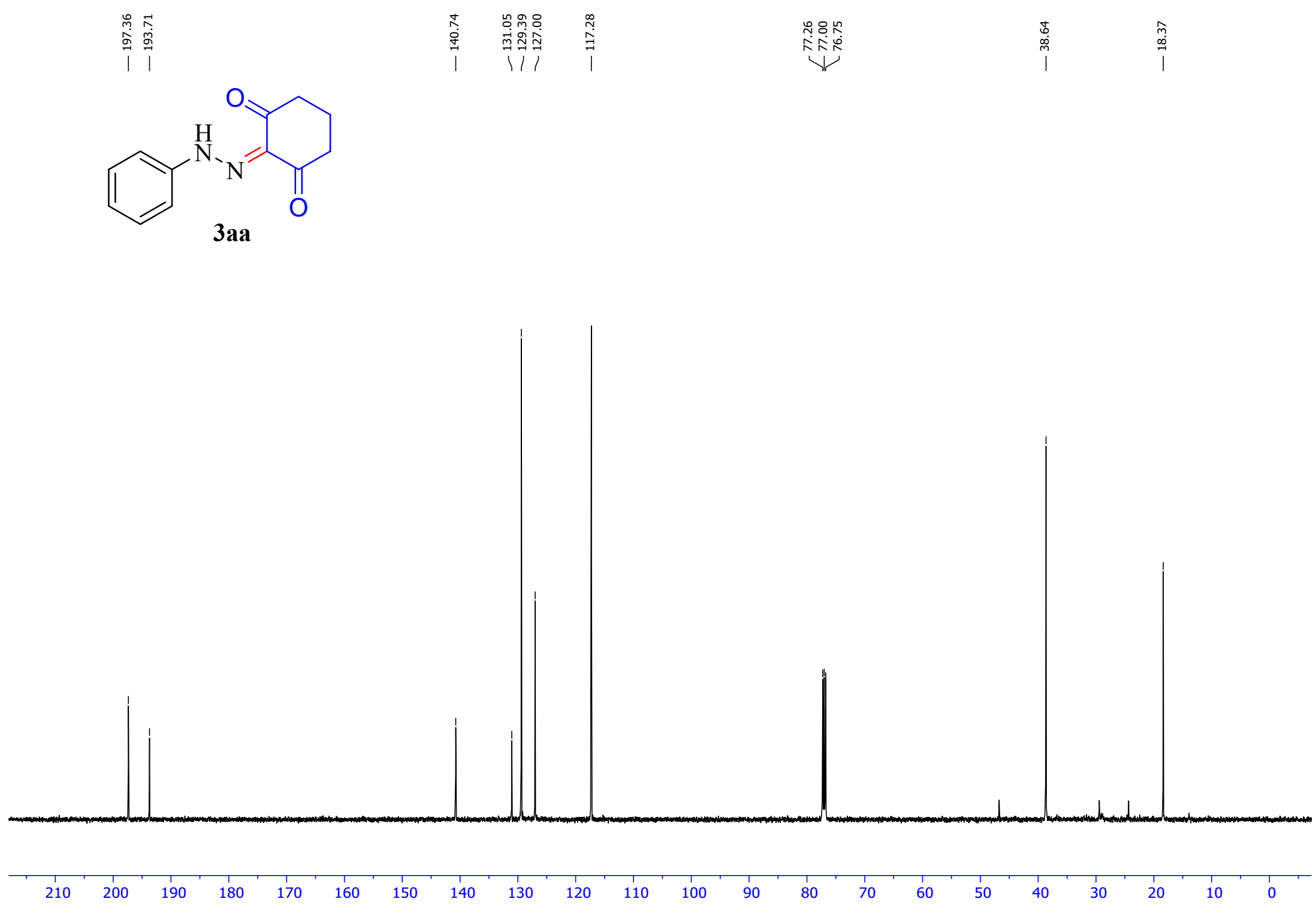

3aa

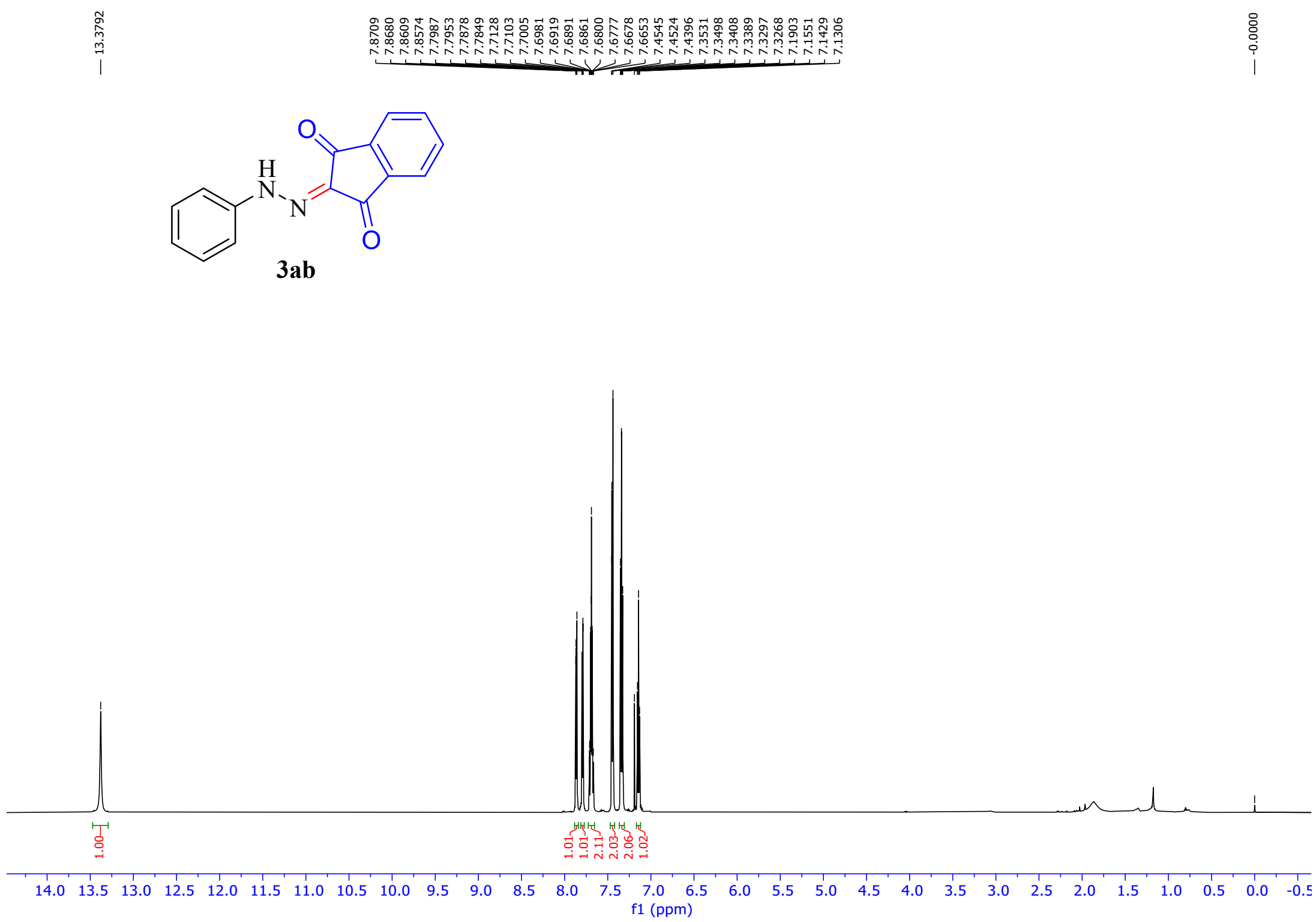
13.3792
7.8709
7.8680
7.8609
7.8574
7.7987
7.7953
7.7878
7.7849
7.7128
7.7103
7.7005
7.6981
7.6919
7.6891
7.6861
7.6800
7.6777
7.6678
7.6653
7.4545
7.4524
7.4396
7.3531
7.3498
7.3408
7.3389
7.3297
7.3268
7.1903
7.1551
7.1429
7.1306
-0.0000
3ab
1.00
1.01
1.01
2.11
2.03
2.06
1.02
f1 (ppm)

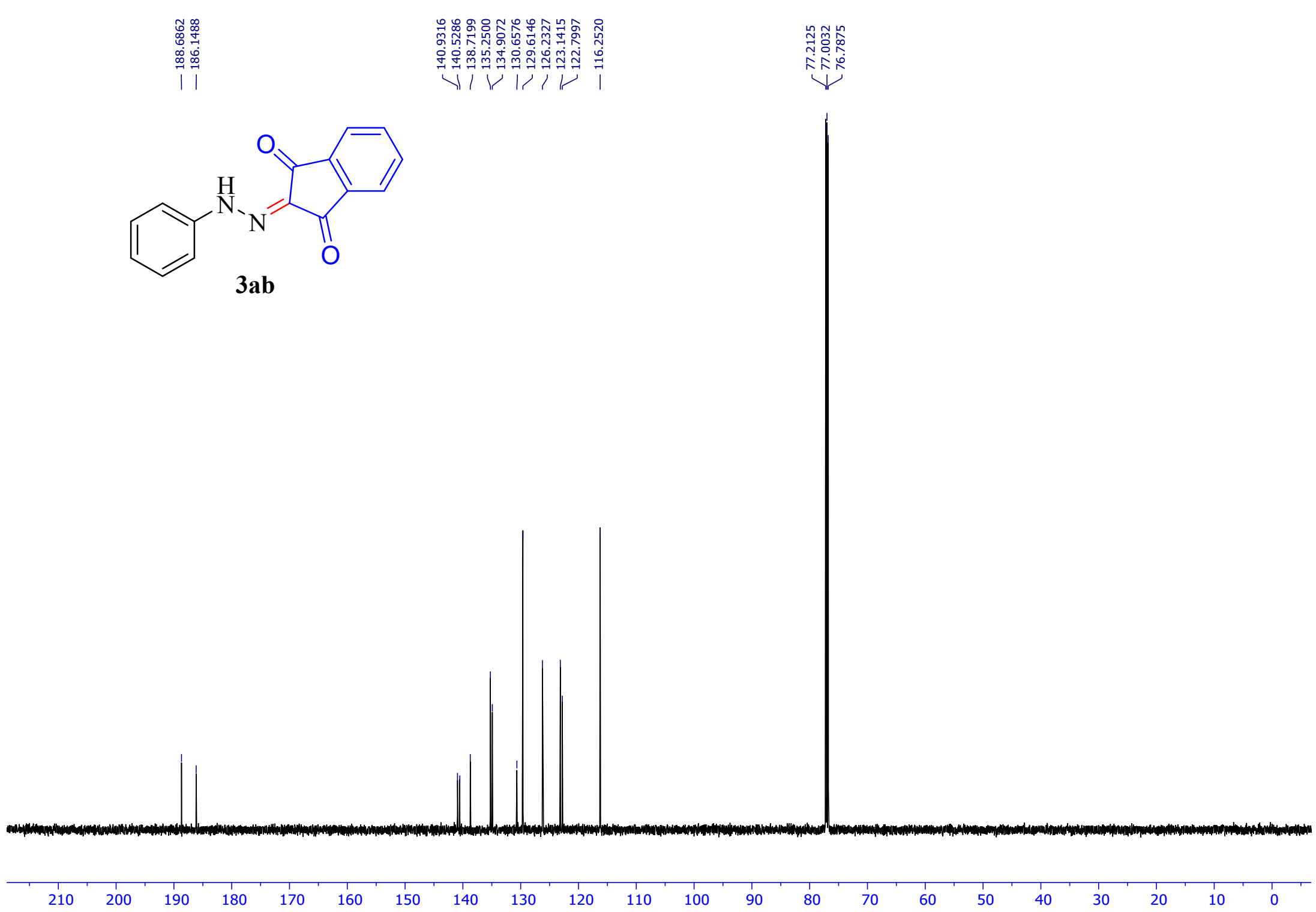
188.6862
186.1488
140.9316
140.5286
138.7199
135.2500
134.9072
130.6576
129.6146
126.2327
123.1415
122.7997
116.2520
77.2125
77.0032
76.7875
3ab
f1 (ppm)

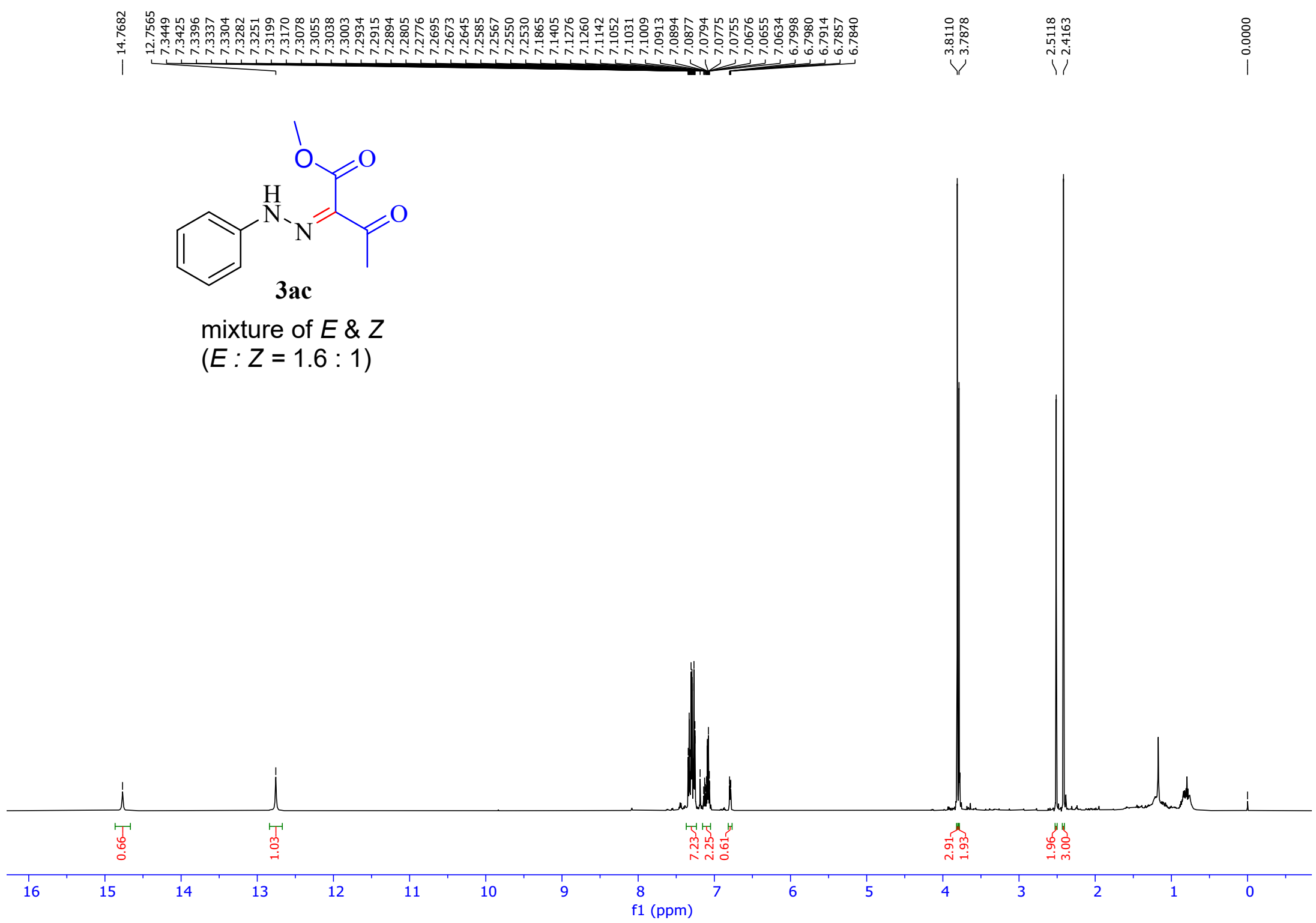
14.7682
12.7565
7.3449
7.3425
7.3396
7.3337
7.3304
7.3282
7.3251
7.3199
7.3170
7.3078
7.3055
7.3038
7.3003
7.2934
7.2915
7.2894
7.2805
7.2776
7.2695
7.2673
7.2645
7.2585
7.2567
7.2550
7.2530
7.1865
7.1405
7.1276
7.1260
7.1142
7.1052
7.1031
7.1009
7.0913
7.0894
7.0877
7.0794
7.0775
7.0755
7.0676
7.0655
7.0634
6.7998
6.7980
6.7914
6.7857
6.7840
3.8110
3.7878
2.5118
2.4163
0.0000
H
N
N
O
O
O
3ac
mixture of E & Z
(E : Z = 1.6 : 1)
0.66
1.03
7.23
2.25
0.61
2.91
1.93
1.96
3.00
16 15 14 13 12 11 10 9 8 7 6 5 4 3 2 1 0
f1 (ppm)

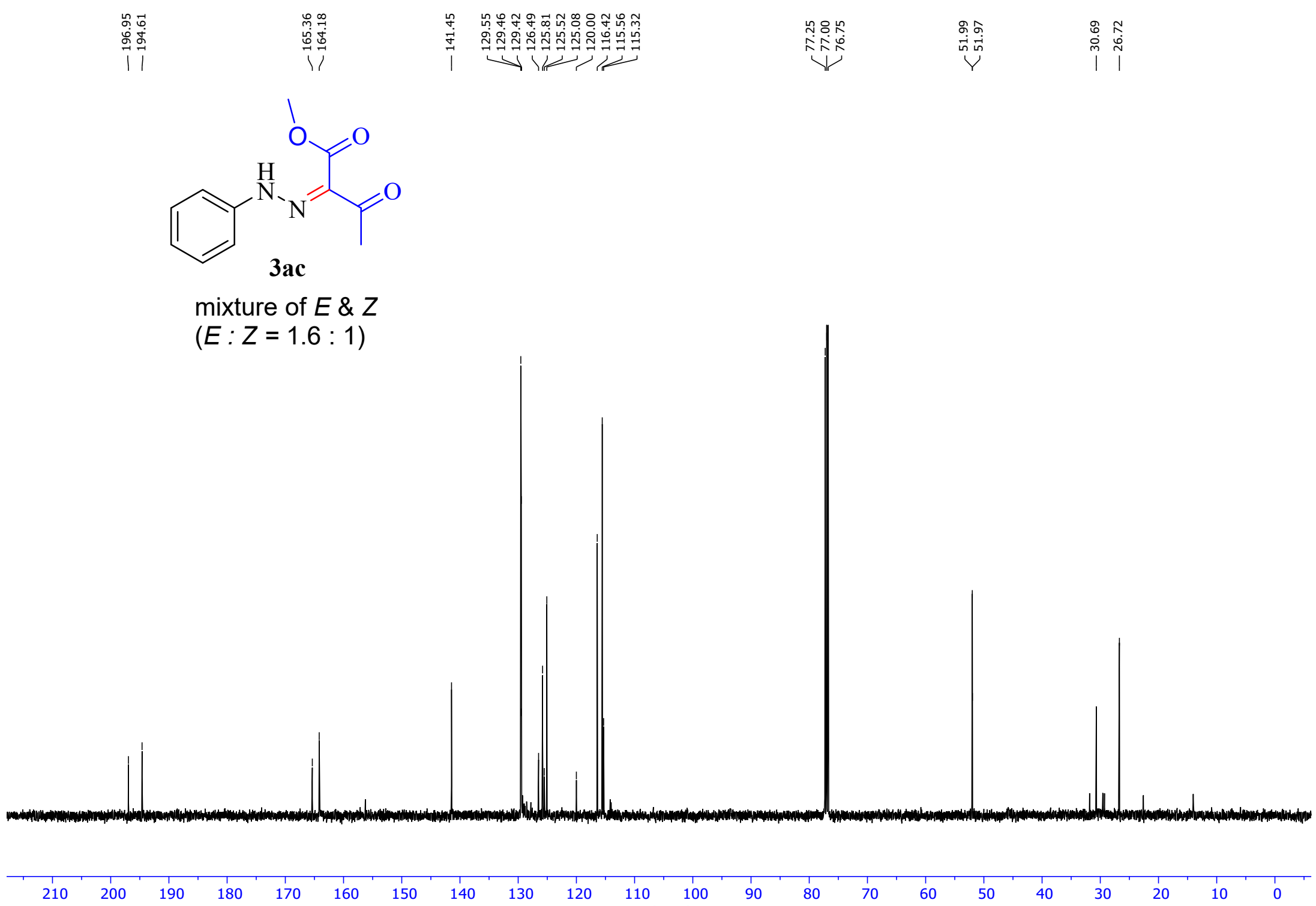
196.95
194.61
165.36
164.18
141.45
129.55
129.46
129.42
126.49
125.81
125.52
125.08
120.00
116.42
115.56
115.32
77.25
77.00
76.75
51.99
51.97
30.69
26.72
H
N
N
O
O
O
3ac
mixture of E & Z
(E : Z = 1.6 : 1)
210 200 190 180 170 160 150 140 130 120 110 100 90 80 70 60 50 40 30 20 10 0
f1 (ppm)

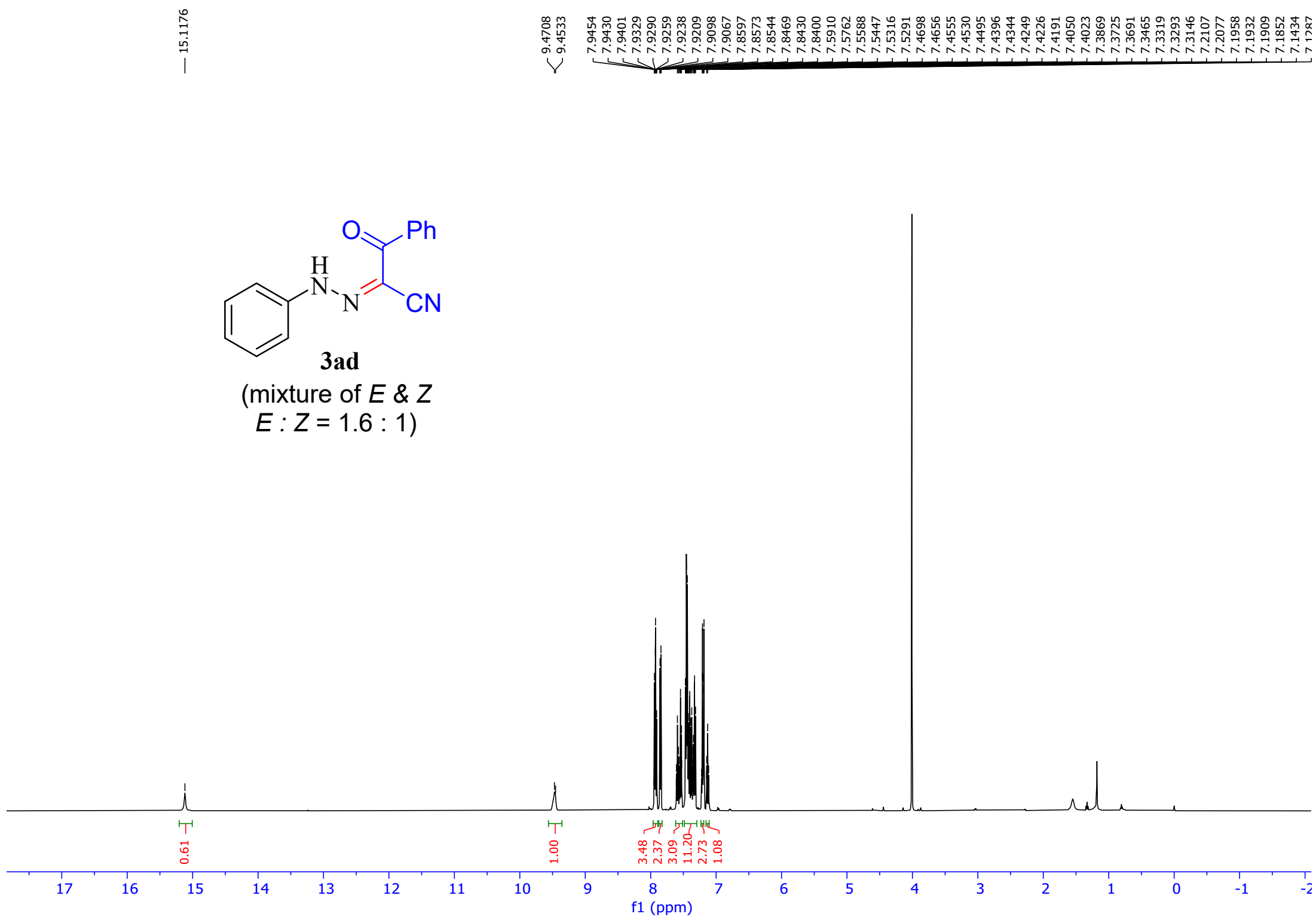

15.1176
9.4708
9.4533
7.9454 7.9430 7.9401 7.9329 7.9290 7.9259 7.9238 7.9209 7.9098 7.9067 7.8597 7.8573 7.8544 7.8469 7.8430 7.8400 7.5910 7.5762 7.5588 7.5447 7.5316 7.5291 7.4698 7.4656 7.4555 7.4530 7.4495 7.4396 7.4344 7.4249 7.4226 7.4191 7.4050 7.4023 7.3869 7.3725 7.3691 7.3465 7.3319 7.3293 7.3146 7.2107 7.2077 7.1958 7.1932 7.1909 7.1852 7.1434
O
Ph
H
N
N
CN
3ad
(mixture of E & Z
E : Z = 1.6 : 1)
0.61
1.00
3.48
2.37
3.09
11.20
2.73
1.08
f1 (ppm)

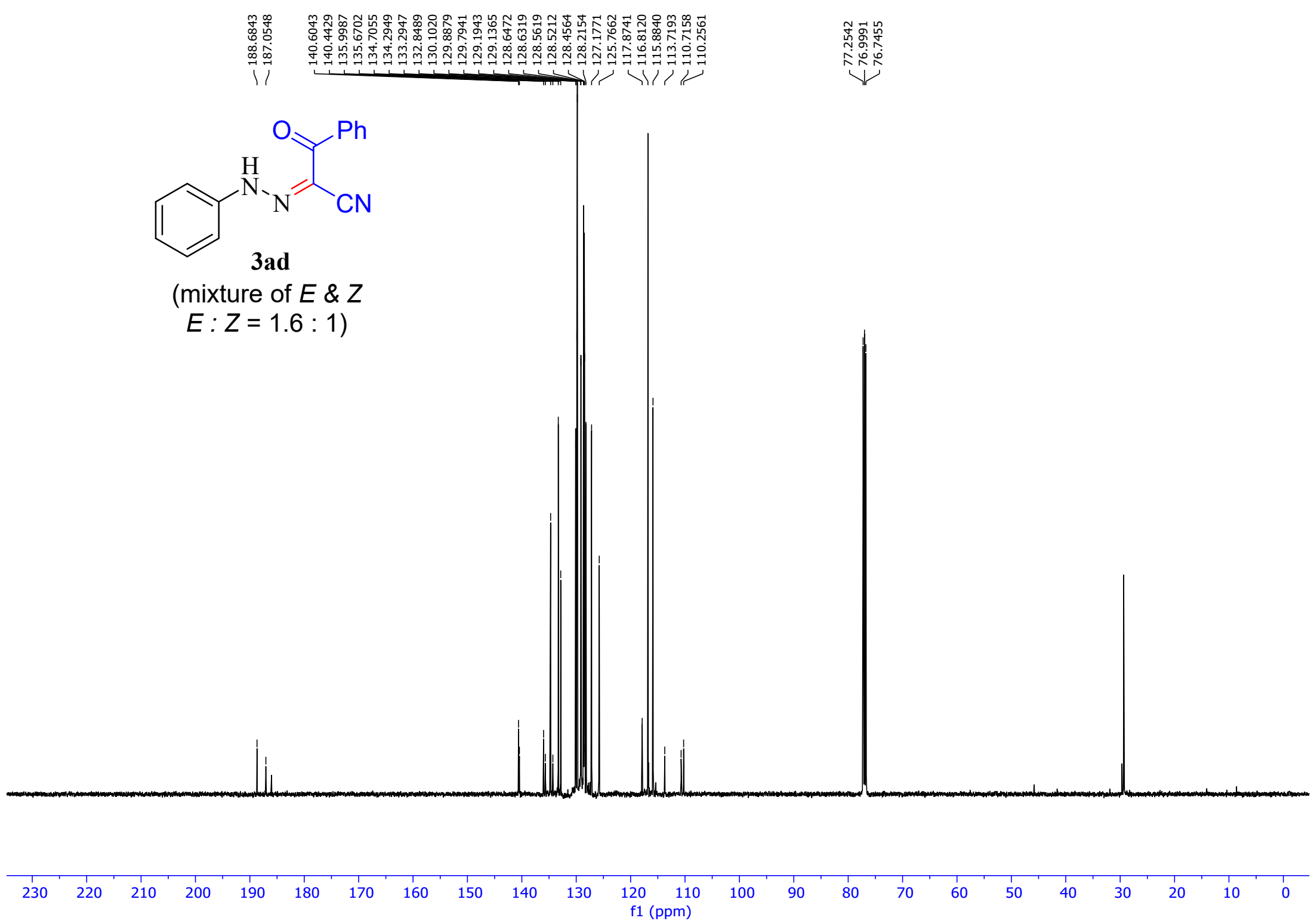

188.6843
187.0548
140.6043 140.4429 135.9987 135.6702 134.7055 134.2949 133.2947 132.8489 130.1020 129.8879 129.7941 129.1943 129.1365 128.6472 128.6319 128.5619 128.5212 128.4564 128.2154 127.1771 125.7662 117.8741 116.8120 115.8840 113.7193 110.7158 110.2561
77.2542
76.9991
76.7455
O
Ph
H
N
N
CN
3ad
(mixture of E & Z
E : Z = 1.6 : 1)
f1 (ppm)

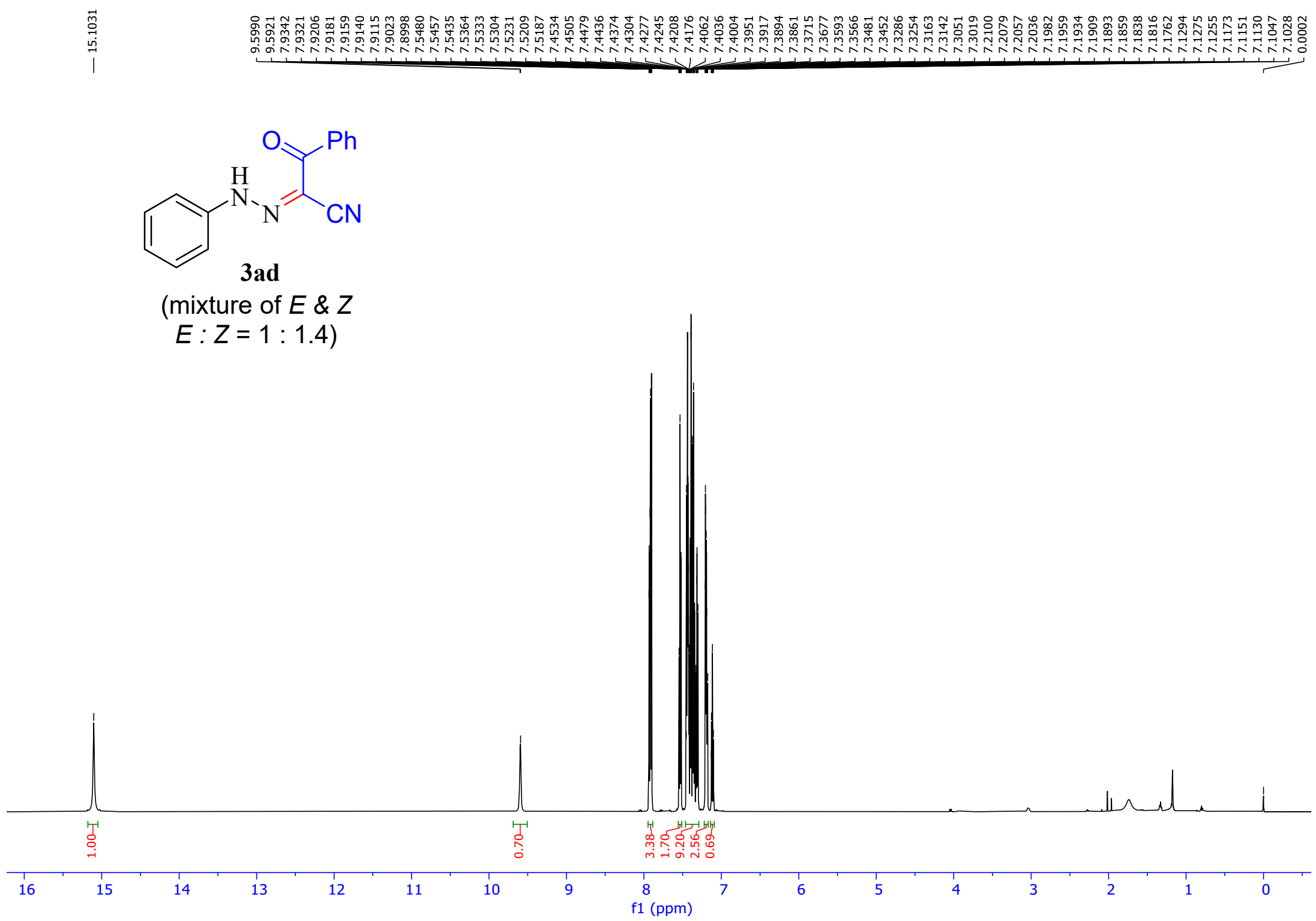
15.1031
9.5990 9.5921 7.9342 7.9321 7.9206 7.9181 7.9159 7.9140 7.9115 7.9023 7.8998 7.5480 7.5457 7.5435 7.5364 7.5333 7.5304 7.5231 7.5209 7.5187 7.4534 7.4505 7.4479 7.4436 7.4374 7.4304 7.4277 7.4245 7.4208 7.4176 7.4062 7.4036 7.4004 7.3951 7.3917 7.3894 7.3861 7.3715 7.3677 7.3593 7.3566 7.3481 7.3452 7.3286 7.3254 7.3163 7.3142 7.3051 7.3019 7.2100 7.2079 7.2057 7.2036 7.1982 7.1959 7.1934 7.1909 7.1893 7.1859 7.1838 7.1816 7.1762 7.1294 7.1275 7.1255 7.1173 7.1151 7.1130 7.1047 7.1028 0.0002
O
Ph
H
N
N
CN
3ad
(mixture of E & Z
E : Z = 1 : 1.4)
1.00
0.70
3.38
1.70
9.20
2.56
0.69
f1 (ppm)

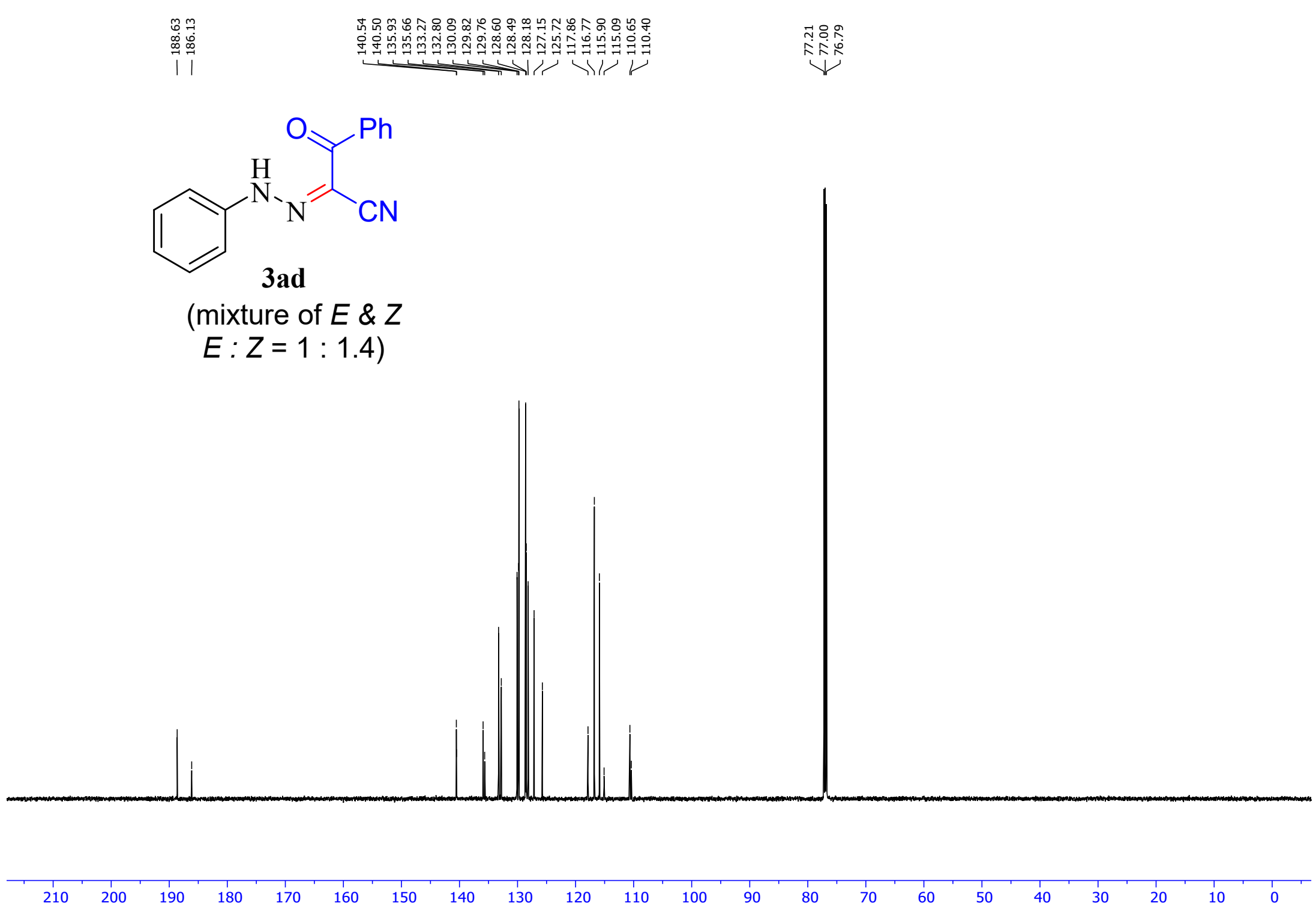
188.63
186.13
140.54 140.50 135.93 135.66 133.27 132.80 130.09 129.82 129.76 128.60 128.49 128.18 127.15 125.72 117.86 116.77 115.90 115.09 110.65 110.40
77.21 77.00 76.79
O
Ph
H
N
N
CN
3ad
(mixture of E & Z
E : Z = 1 : 1.4)
f1 (ppm)

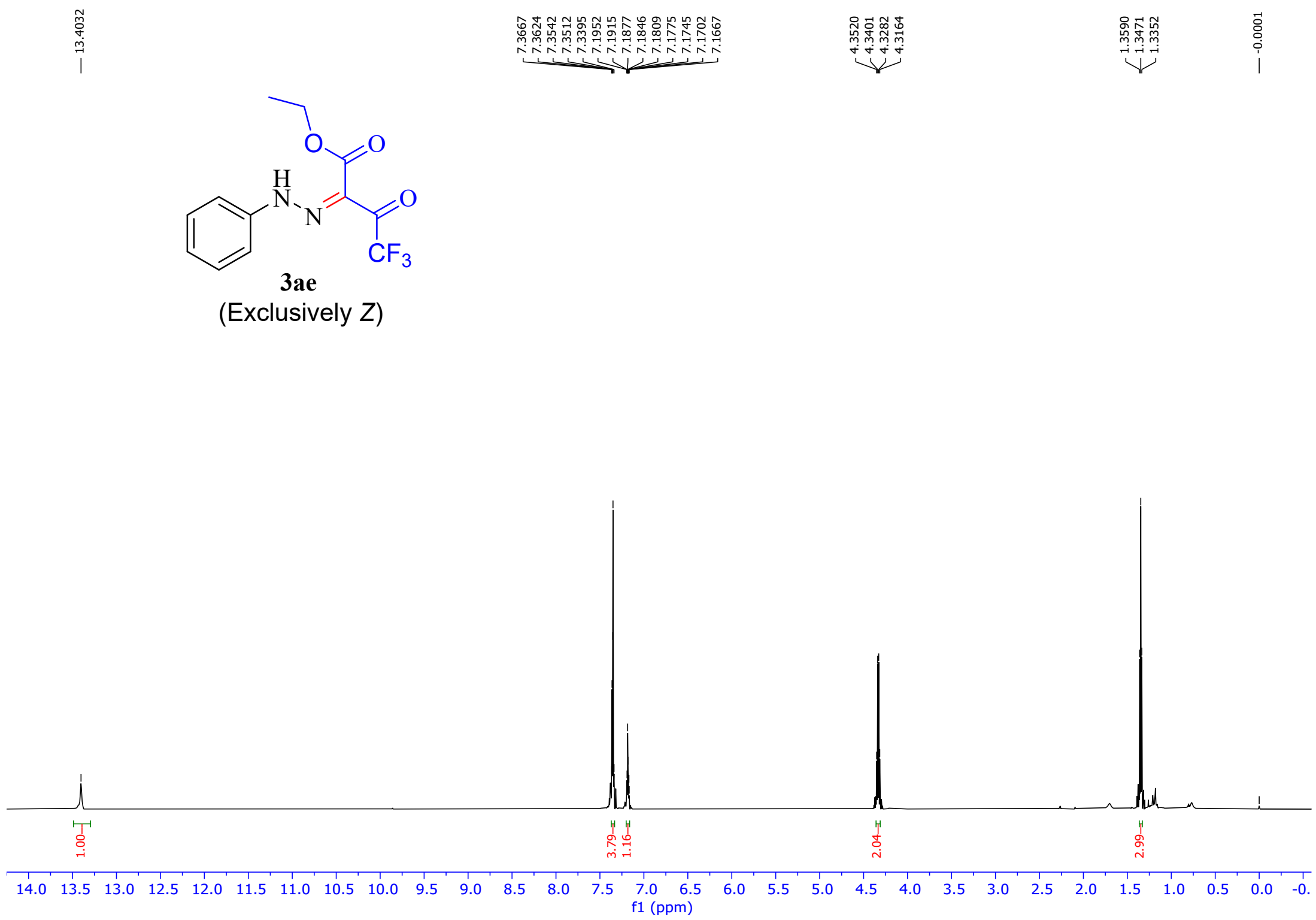
13.4032
7.3667
7.3624
7.3542
7.3512
7.3395
7.1952
7.1915
7.1877
7.1846
7.1809
7.1775
7.1745
7.1702
7.1667
4.3520
4.3401
4.3282
4.3164
1.3590
1.3471
1.3352
-0.0001
3ae
(Exclusively Z)
1.00
3.79
1.16
2.04
2.99
f1 (ppm)

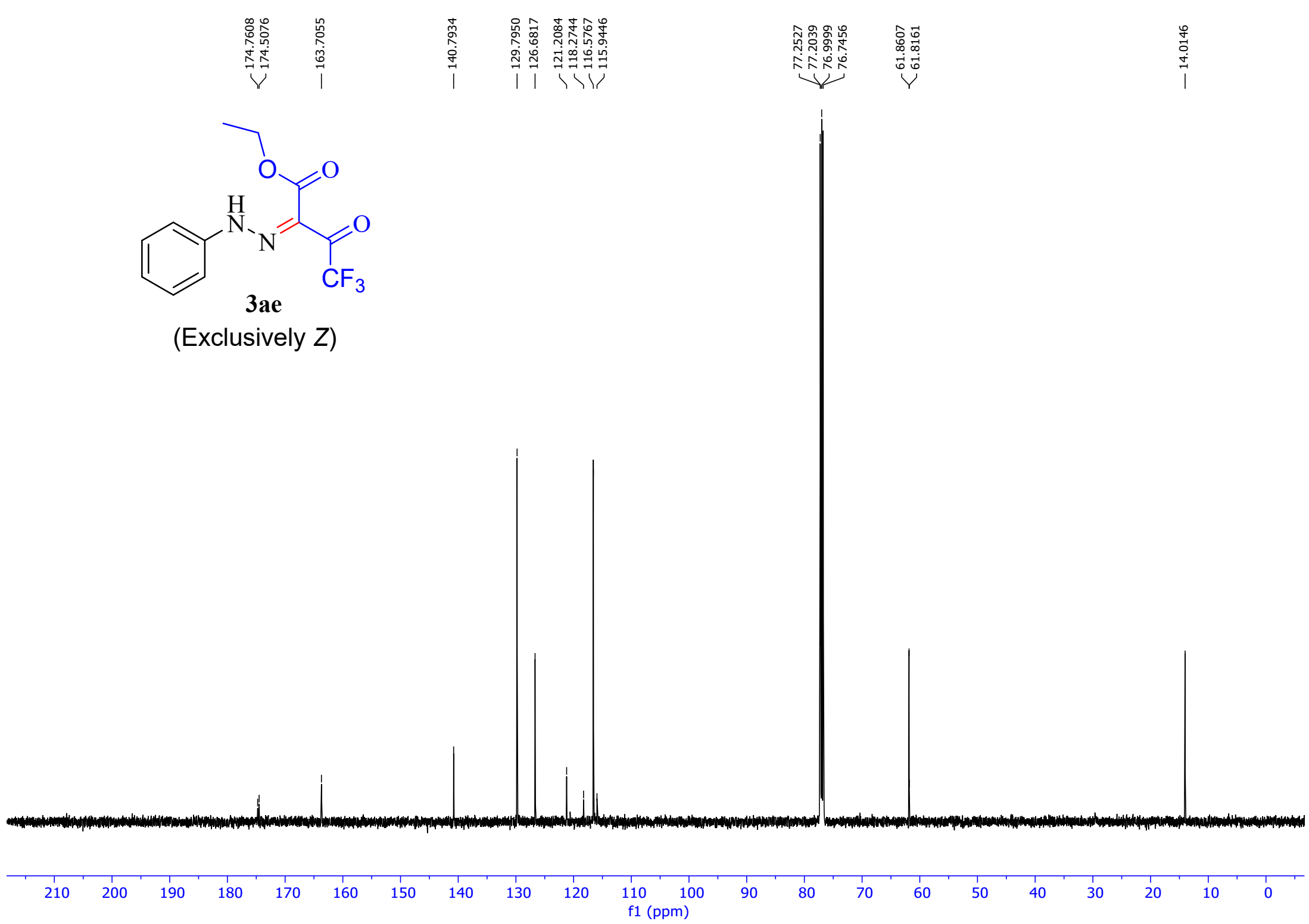
174.7608
174.5076
163.7055
140.7934
129.7950
126.6817
121.2084
118.2744
116.5767
115.9446
77.2527
77.2039
76.9999
76.7456
61.8607
61.8161
14.0146
3ae
(Exclusively Z)
f1 (ppm)

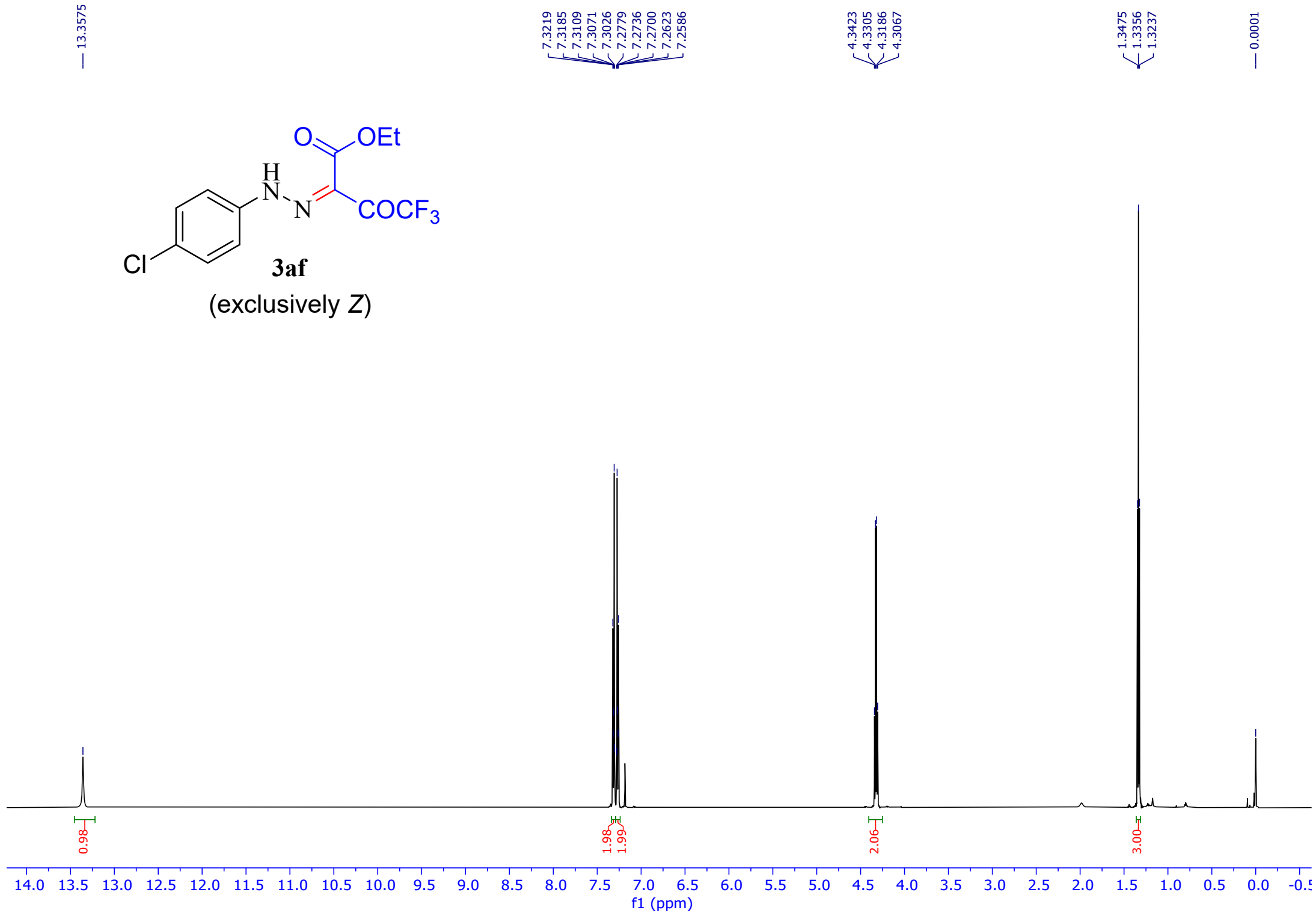
13.3575
7.3219
7.3185
7.3109
7.3071
7.3026
7.2779
7.2736
7.2700
7.2623
7.2586
4.3423
4.3305
4.3186
4.3067
1.3475
1.3356
1.3237
0.0001
O
OEt
H
N
N
COCF3
Cl
3af
(exclusively Z)
0.98
1.98
1.99
2.06
3.00
f1 (ppm)

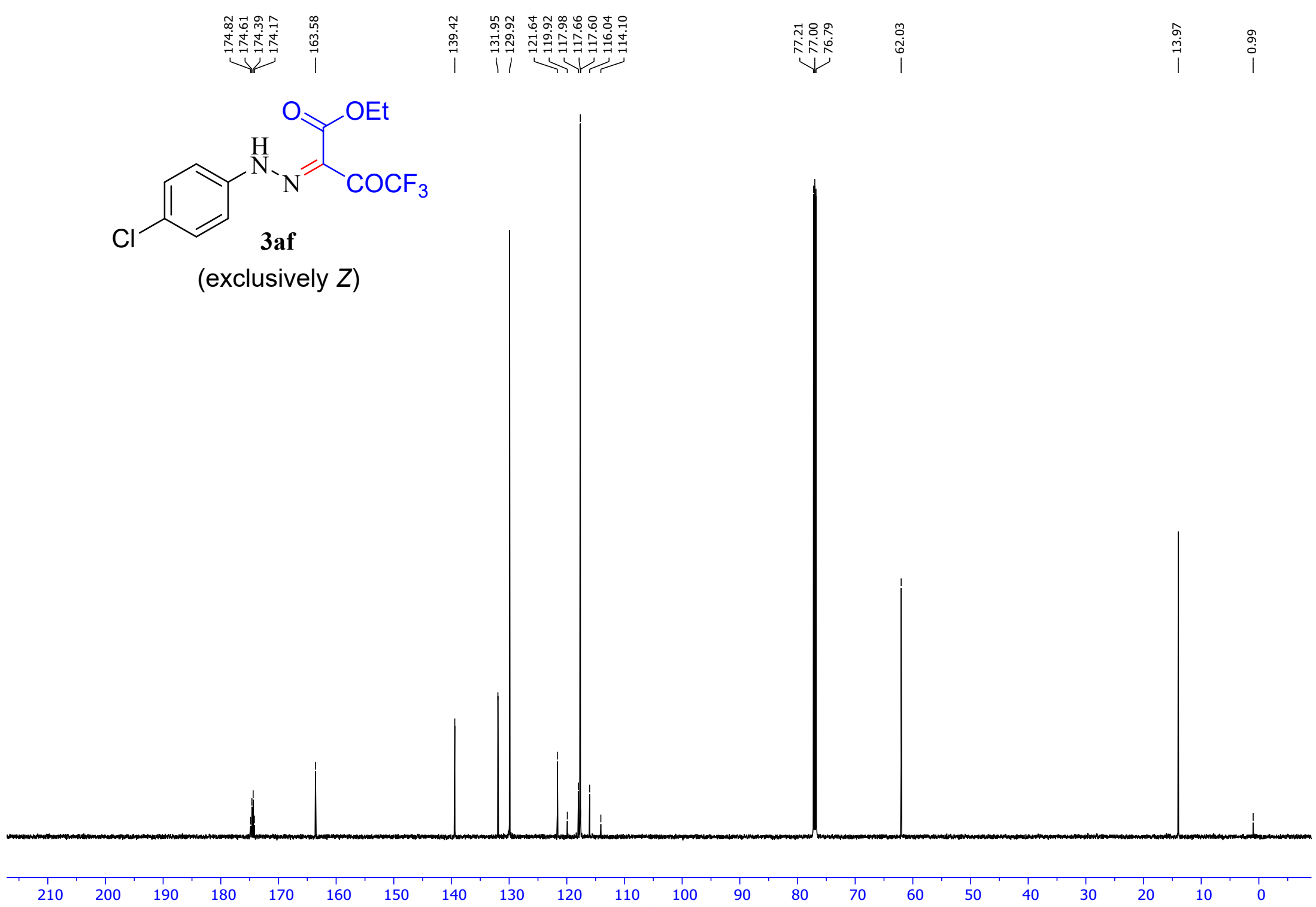
174.82
174.61
174.39
174.17
163.58
139.42
131.95
129.92
121.64
119.92
117.98
117.66
117.60
116.04
114.10
77.21
77.00
76.79
62.03
13.97
0.99
O
OEt
H
N
N
COCF3
Cl
3af
(exclusively Z)
f1 (ppm)

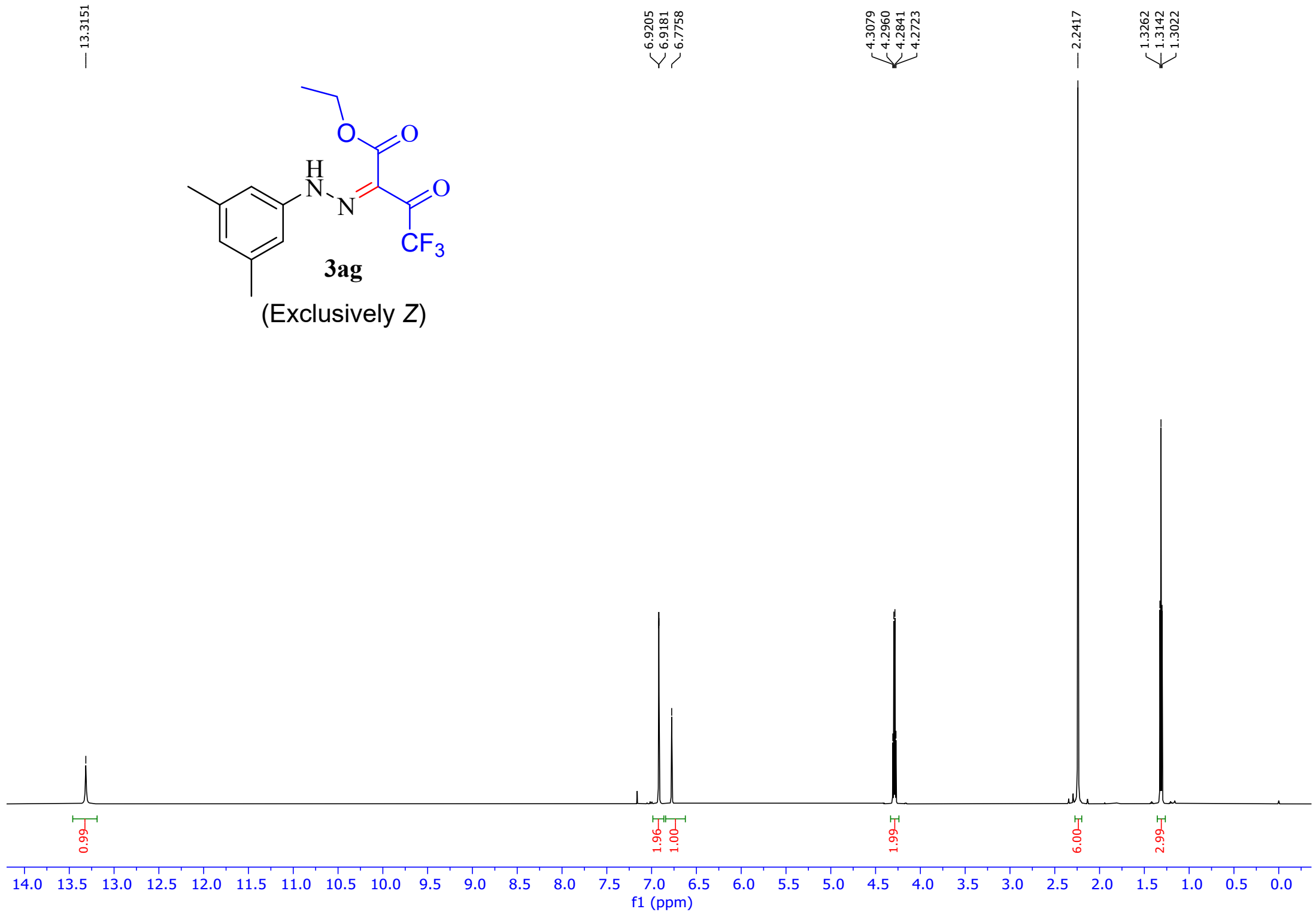

13.3151
6.9205
6.9181
6.7758
4.3079
4.2960
4.2841
4.2723
2.2417
1.3262
1.3142
1.3022
3ag
(Exclusively Z)
0.99
1.96
1.00
1.99
6.00
2.99
14.0 13.5 13.0 12.5 12.0 11.5 11.0 10.5 10.0 9.5 9.0 8.5 8.0 7.5 7.0 6.5 6.0 5.5 5.0 4.5 4.0 3.5 3.0 2.5 2.0 1.5 1.0 0.5 0.0
f1 (ppm)

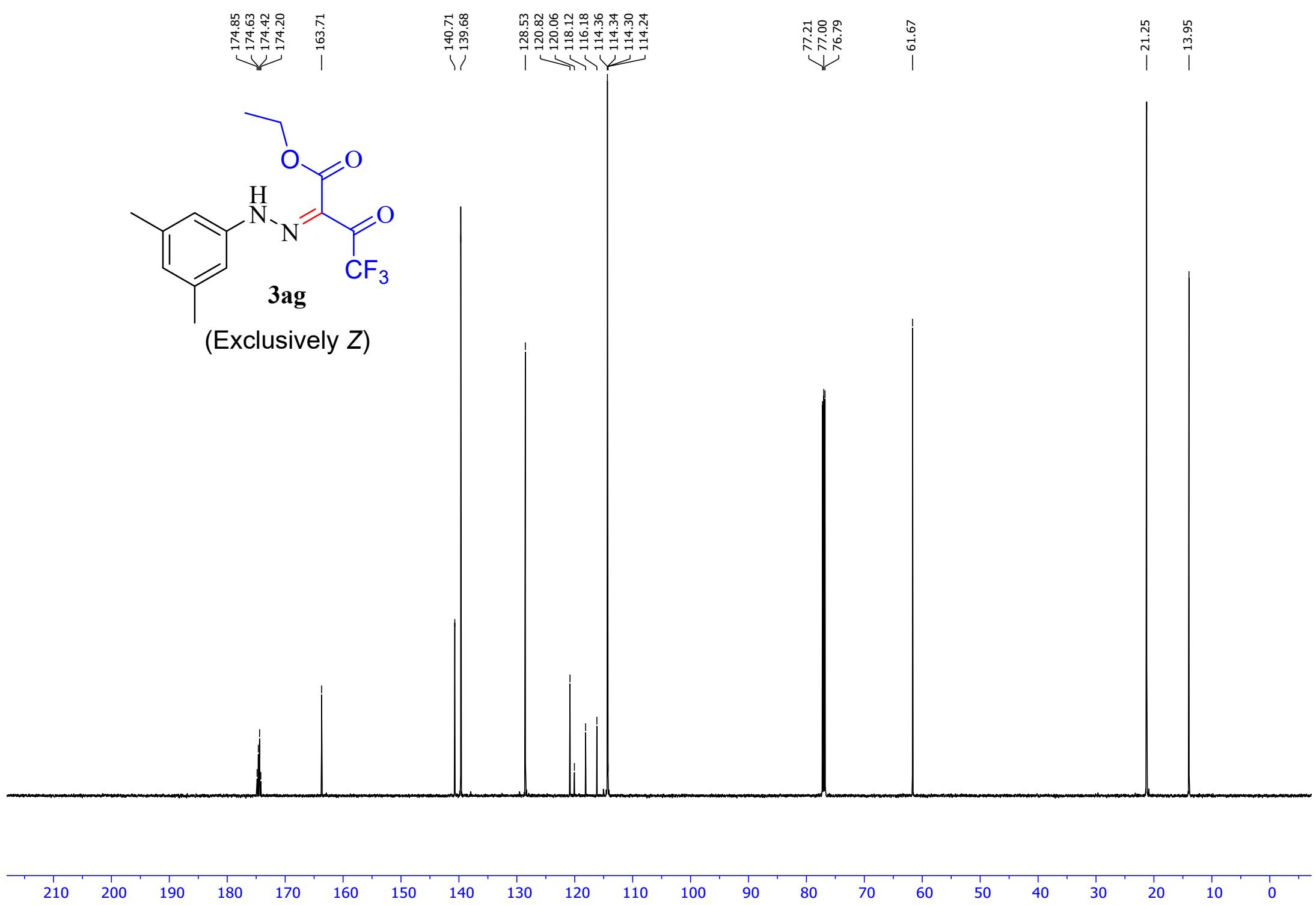

174.85
174.63
174.42
174.20
163.71
140.71
139.68
128.53
120.82
120.06
118.12
116.18
114.36
114.34
114.30
114.24
77.21
77.00
76.79
61.67
21.25
13.95
3ag
(Exclusively Z)
210 200 190 180 170 160 150 140 130 120 110 100 90 80 70 60 50 40 30 20 10 0
f1 (ppm)

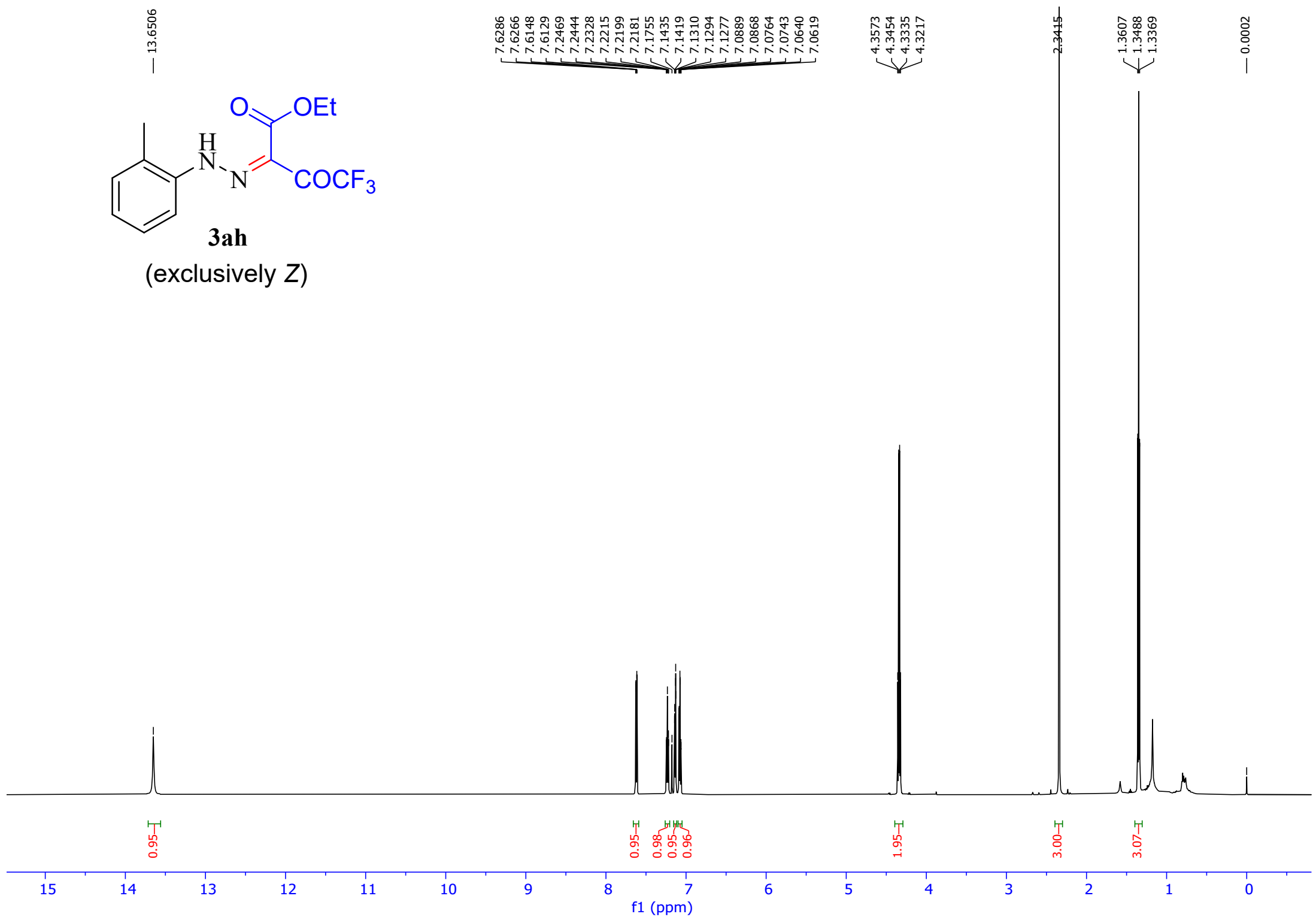

13.6506
7.6286
7.6266
7.6148
7.6129
7.2469
7.2444
7.2328
7.2215
7.2199
7.2181
7.1755
7.1435
7.1419
7.1310
7.1294
7.1277
7.0889
7.0868
7.0764
7.0743
7.0640
7.0619
4.3573
4.3454
4.3335
4.3217
2.3415
1.3607
1.3488
1.3369
0.0002
OEt
COCF3
3ah
(exclusively Z)
f1 (ppm)

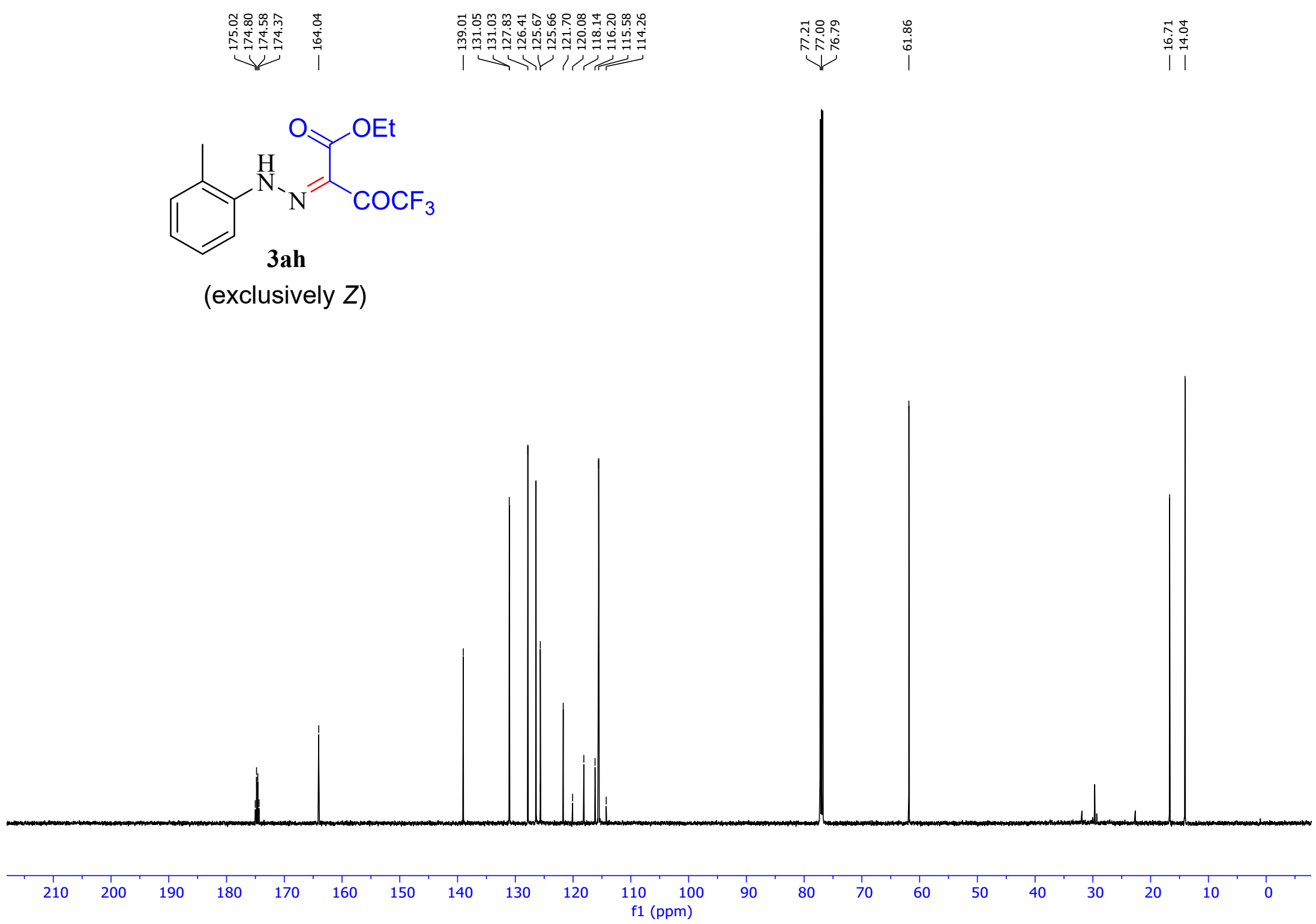

175.02
174.80
174.58
174.37
164.04
139.01
131.05
131.03
127.83
126.41
125.67
125.66
121.70
120.08
118.14
116.20
115.58
114.26
77.21
77.00
76.79
61.86
16.71
14.04
OEt
COCF3
3ah
(exclusively Z)
f1 (ppm)

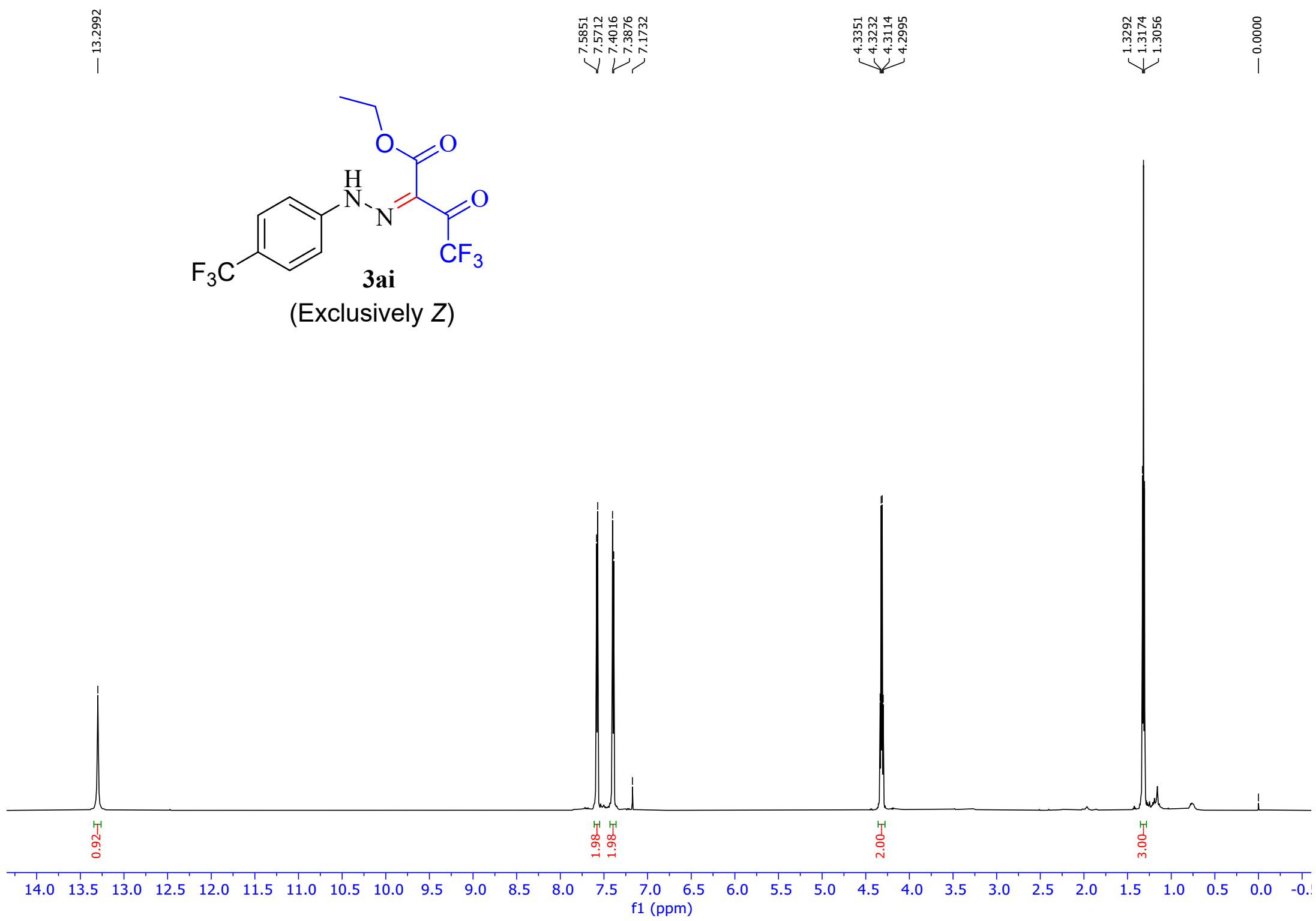
13.2992
7.5851
7.5712
7.4016
7.3876
7.1732
4.3351
4.3232
4.3114
4.2995
1.3292
1.3174
1.3056
0.0000
3ai
(Exclusively Z)
0.92
1.98
1.98
2.00
3.00
f1 (ppm)

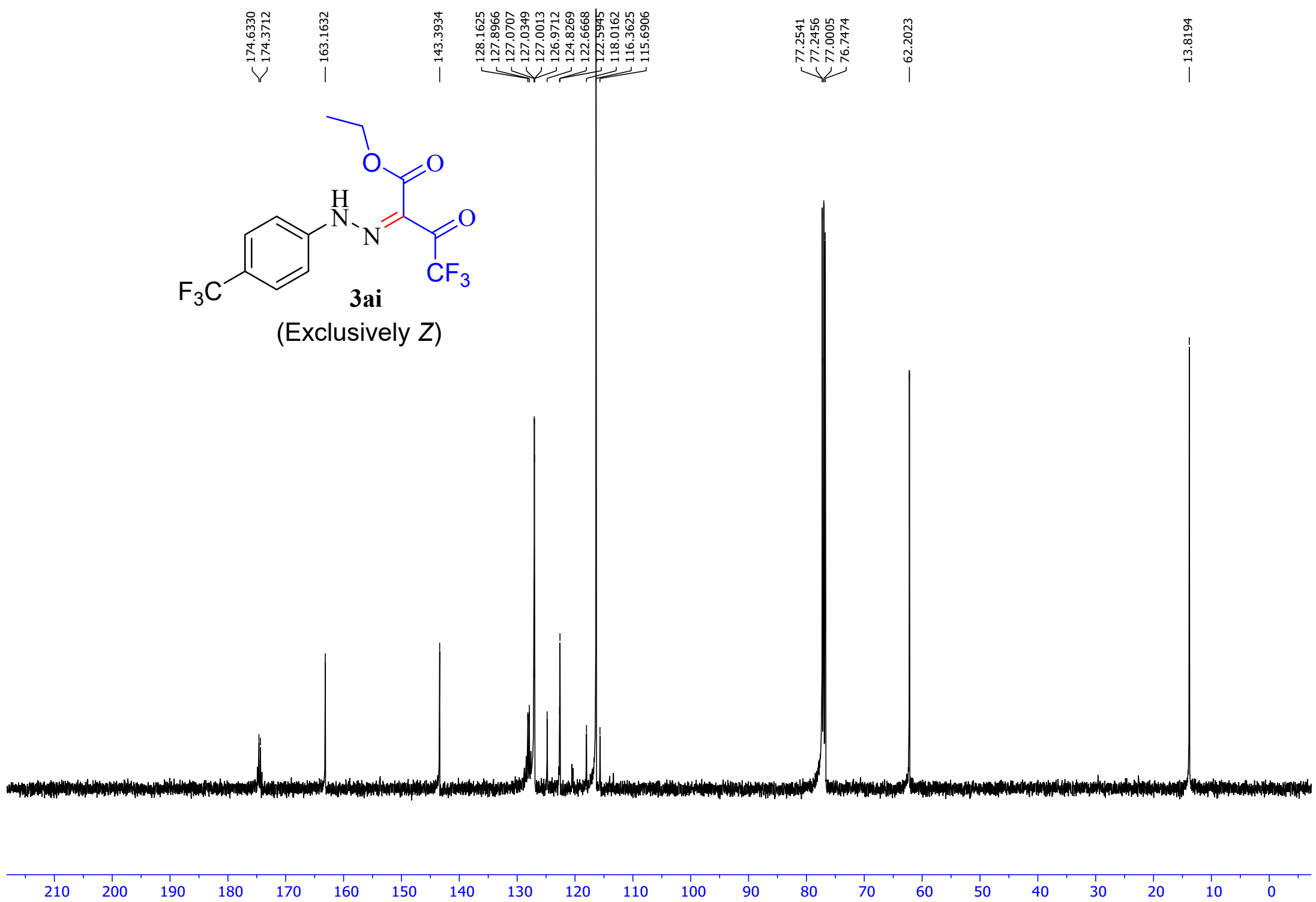
174.6330
174.3712
163.1632
143.3934
128.1625
127.8966
127.0707
127.0349
127.0013
126.9712
124.8269
122.6668
122.5945
118.0162
116.3625
115.6906
77.2541
77.2456
77.0005
76.7474
62.2023
13.8194
3ai
(Exclusively Z)
f1 (ppm)

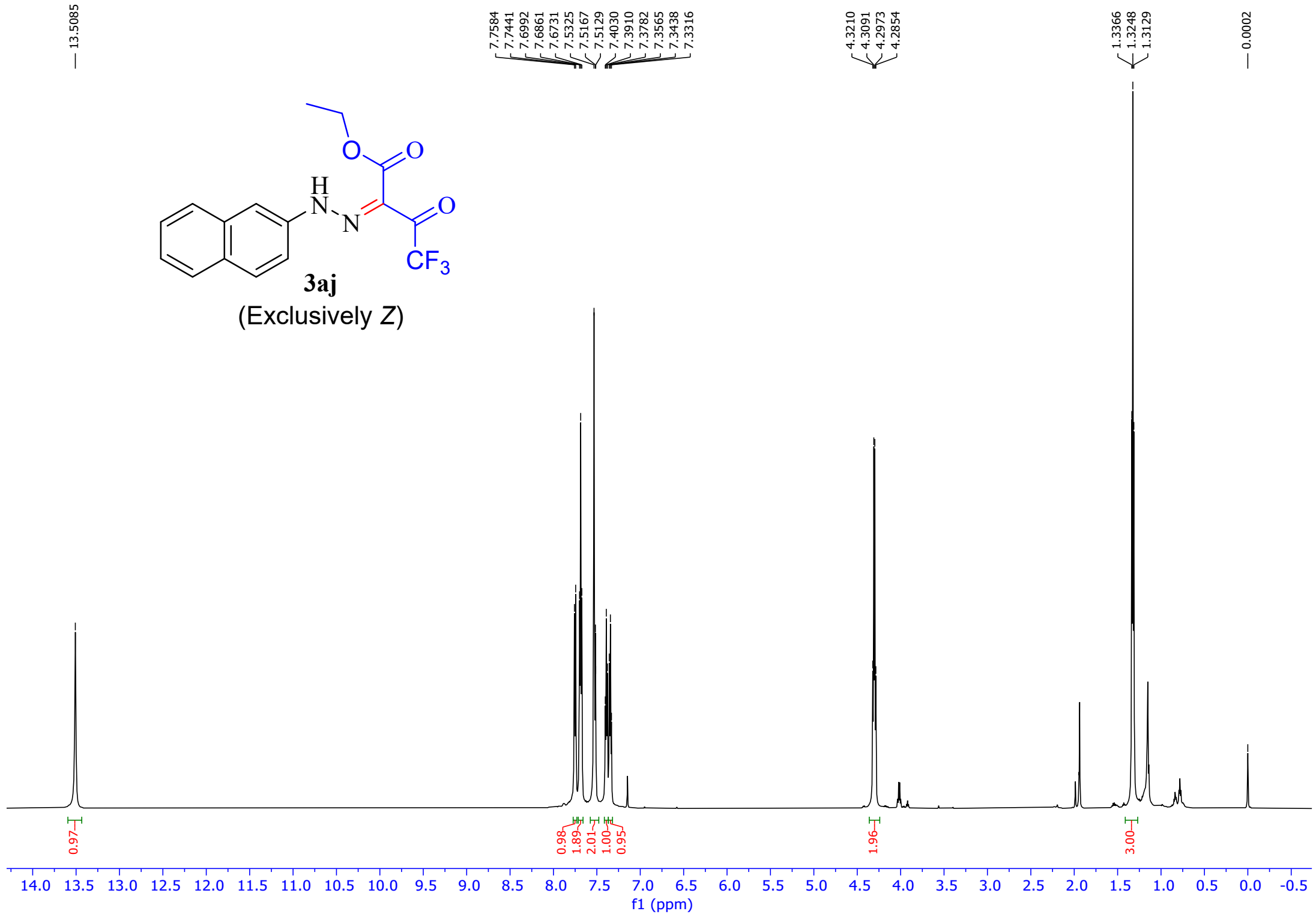

3aj
(Exclusively *Z*)

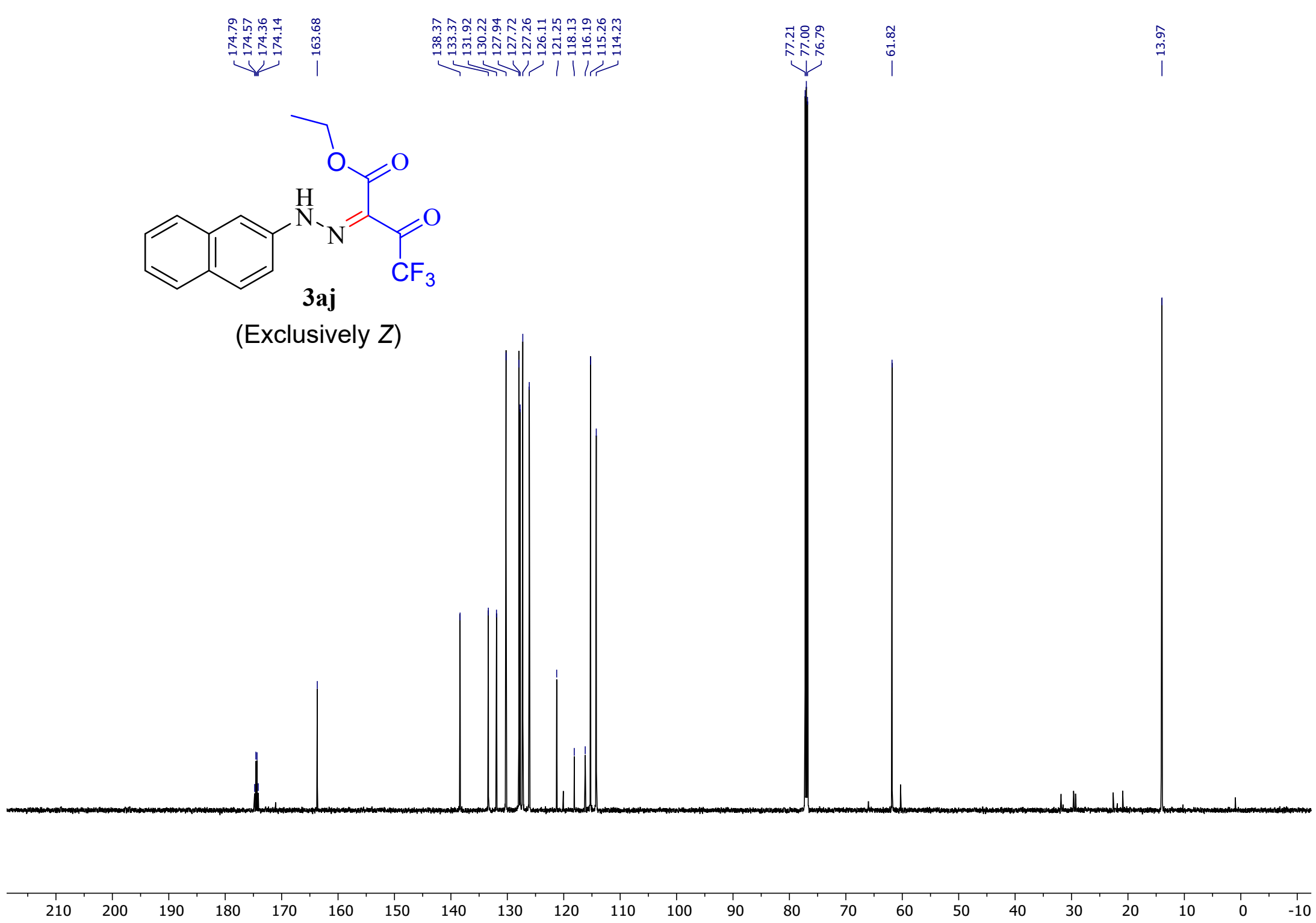

3aj
(Exclusively *Z*)

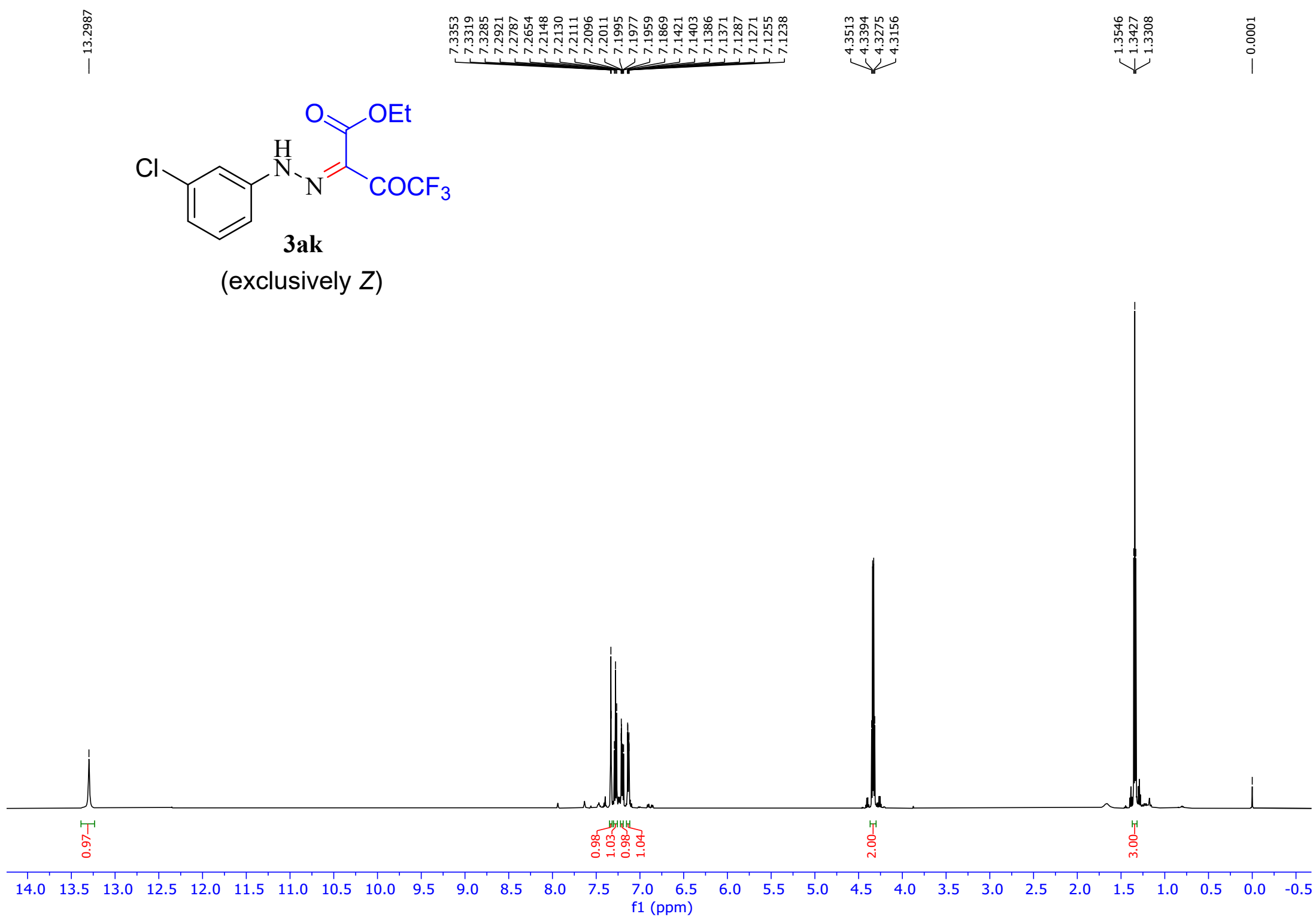
13.2987
7.3353 7.3319 7.3285 7.2921 7.2787 7.2654 7.2148 7.2130 7.2111 7.2096 7.2011 7.1995 7.1977 7.1959 7.1869 7.1421 7.1403 7.1386 7.1371 7.1287 7.1271 7.1255 7.1238
4.3513 4.3394 4.3275 4.3156
1.3546 1.3427 1.3308
0.0001
O
OEt
Cl
H
N
N
COCF3
3ak
(exclusively Z)
0.97
0.98
1.03
0.98
1.04
2.00
3.00
f1 (ppm)

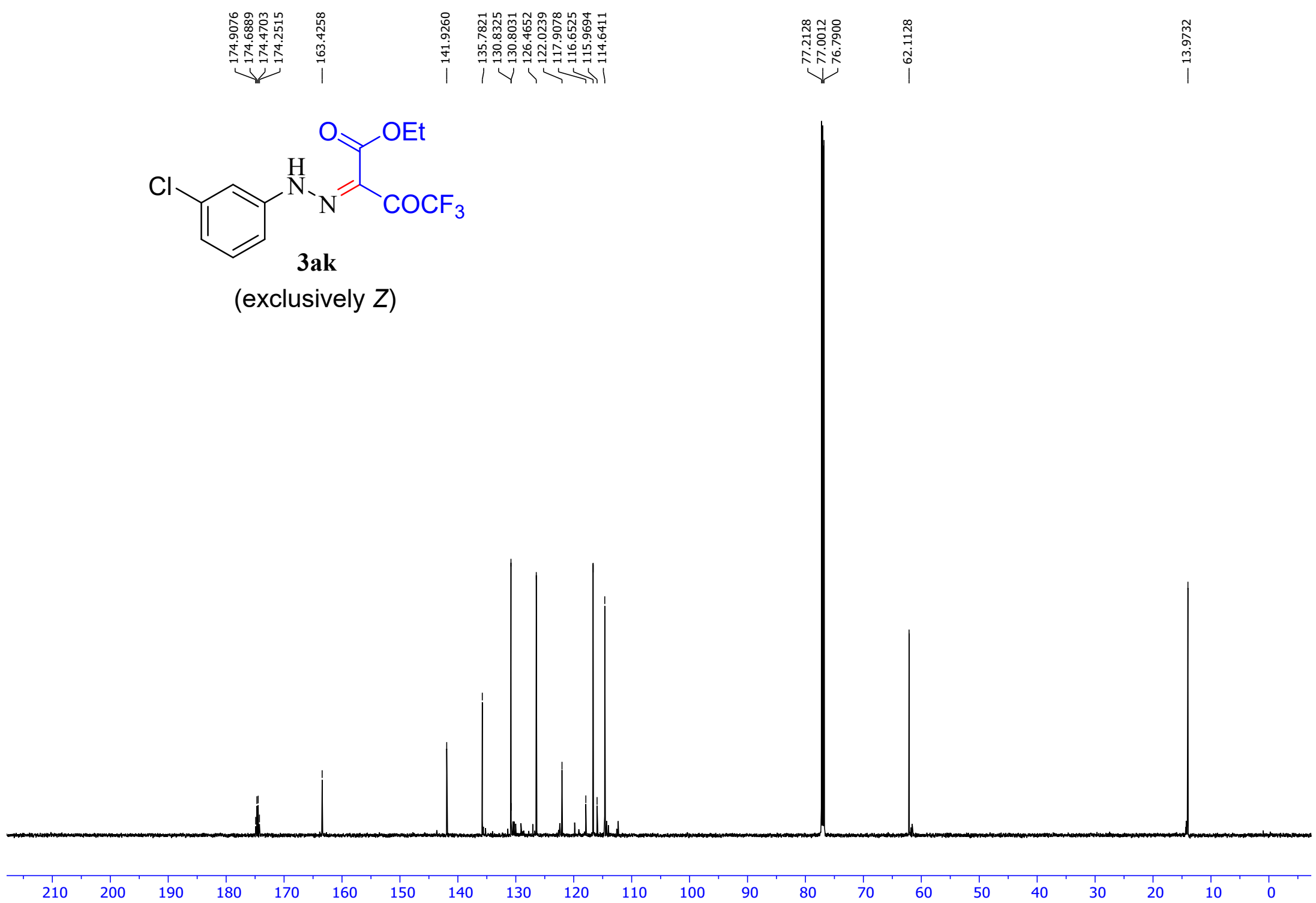
174.9076 174.6889 174.4703 174.2515
163.4258
141.9260
135.7821 130.8325 130.8031 126.4652 122.0239 117.9028 116.4525 115.9694 114.6411
77.2128 77.0012 76.7900
62.1128
13.9732
O
OEt
Cl
H
N
N
COCF3
3ak
(exclusively Z)
f1 (ppm)

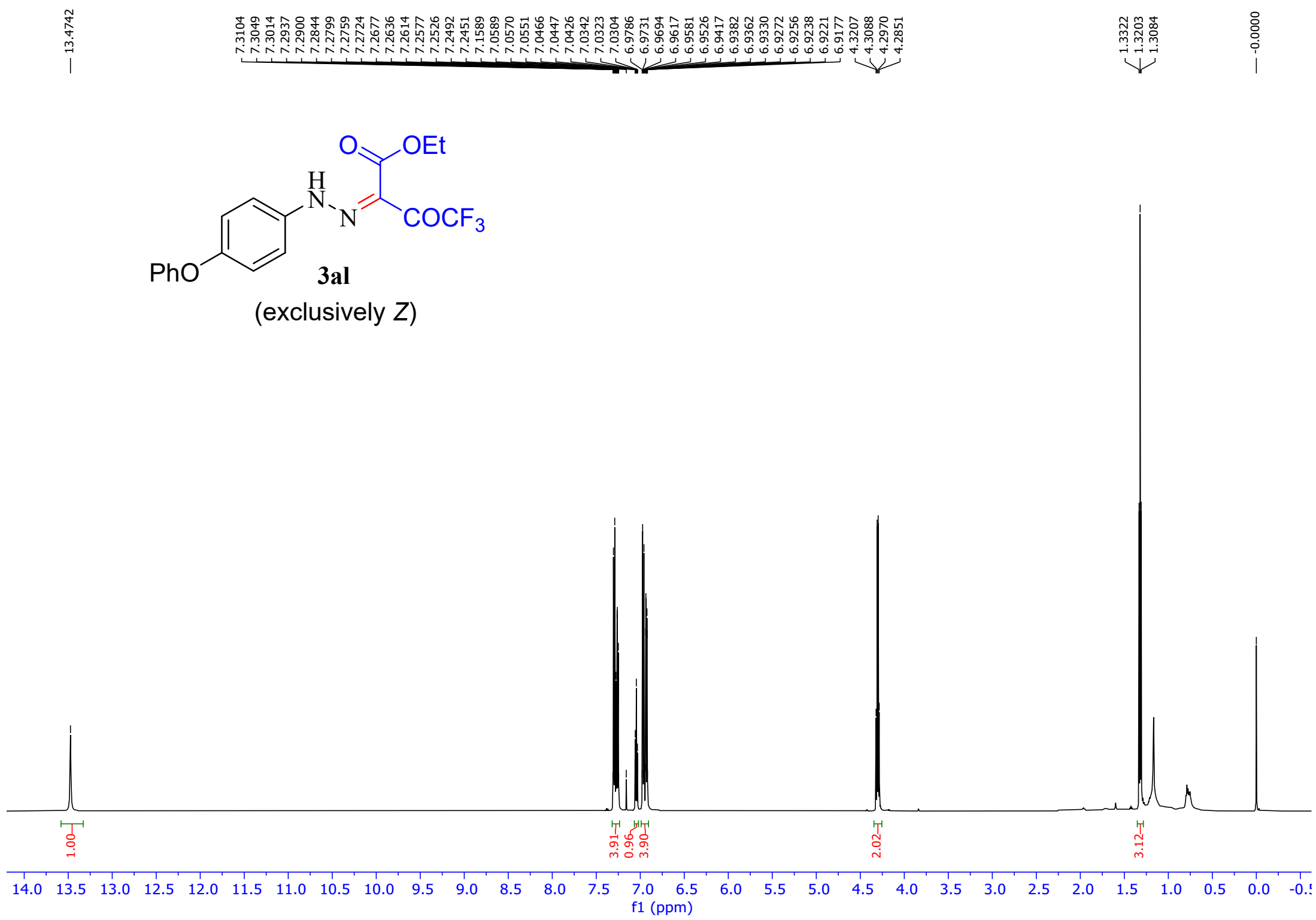
13.4742
7.3104
7.3049
7.3014
7.2937
7.2900
7.2844
7.2799
7.2759
7.2724
7.2677
7.2636
7.2614
7.2577
7.2526
7.2492
7.2451
7.1589
7.0589
7.0570
7.0551
7.0466
7.0447
7.0426
7.0342
7.0323
7.0304
6.9786
6.9731
6.9694
6.9617
6.9581
6.9526
6.9417
6.9382
6.9362
6.9330
6.9272
6.9256
6.9238
6.9221
6.9177
4.3207
4.3088
4.2970
4.2851
1.3322
1.3203
1.3084
-0.0000
O
OEt
H
N
N
COCF3
PhO
3al
(exclusively Z)
1.00
3.91
0.96
3.90
2.02
3.12
f1 (ppm)

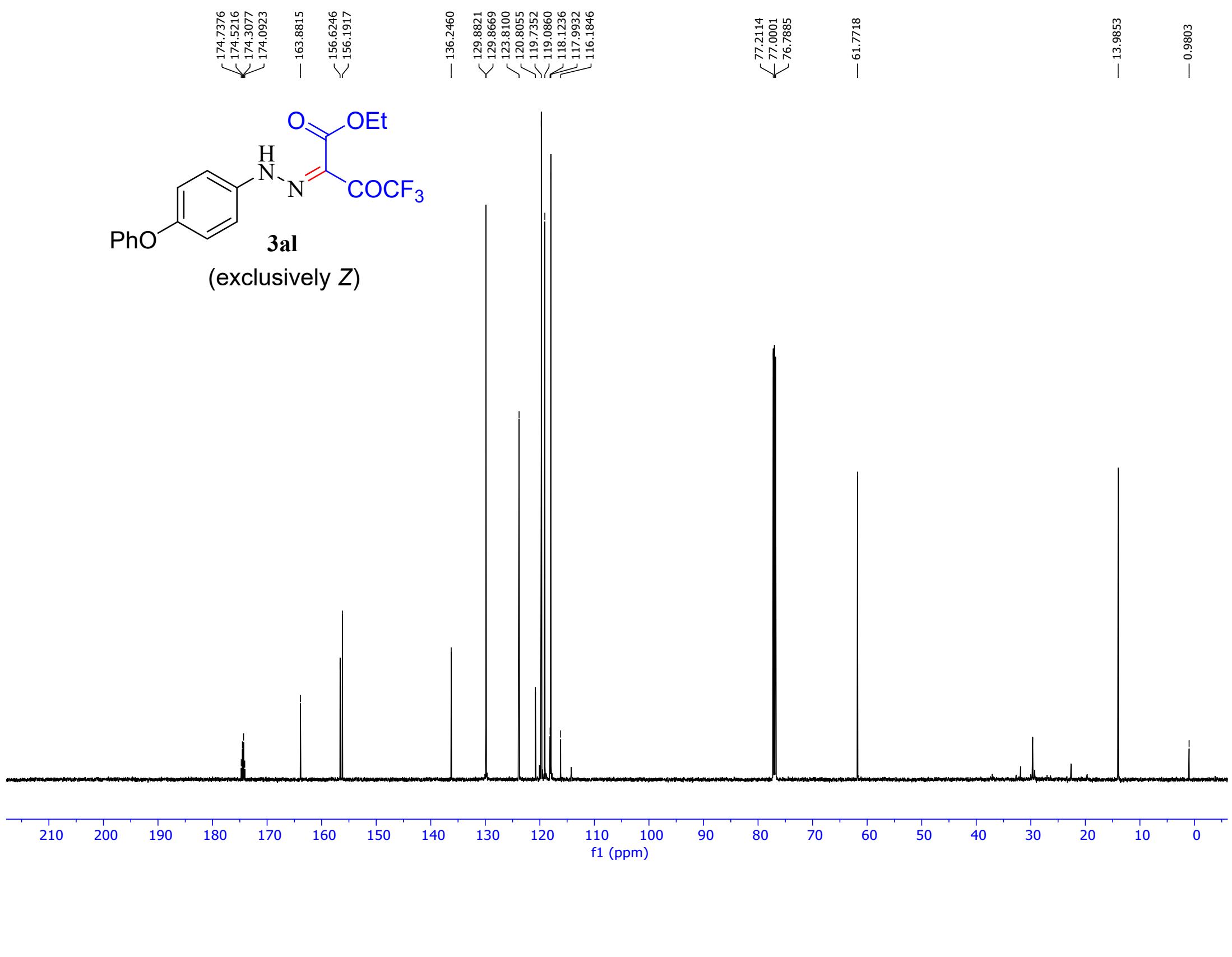
174.7376
174.5216
174.3077
174.0923
163.8815
156.6246
156.1917
136.2460
129.8821
129.8669
123.8100
120.8055
119.7352
119.0860
118.1236
117.9932
116.1846
77.2114
77.0001
76.7885
61.7718
13.9853
0.9803
O
OEt
H
N
N
COCF3
PhO
3al
(exclusively Z)
f1 (ppm)

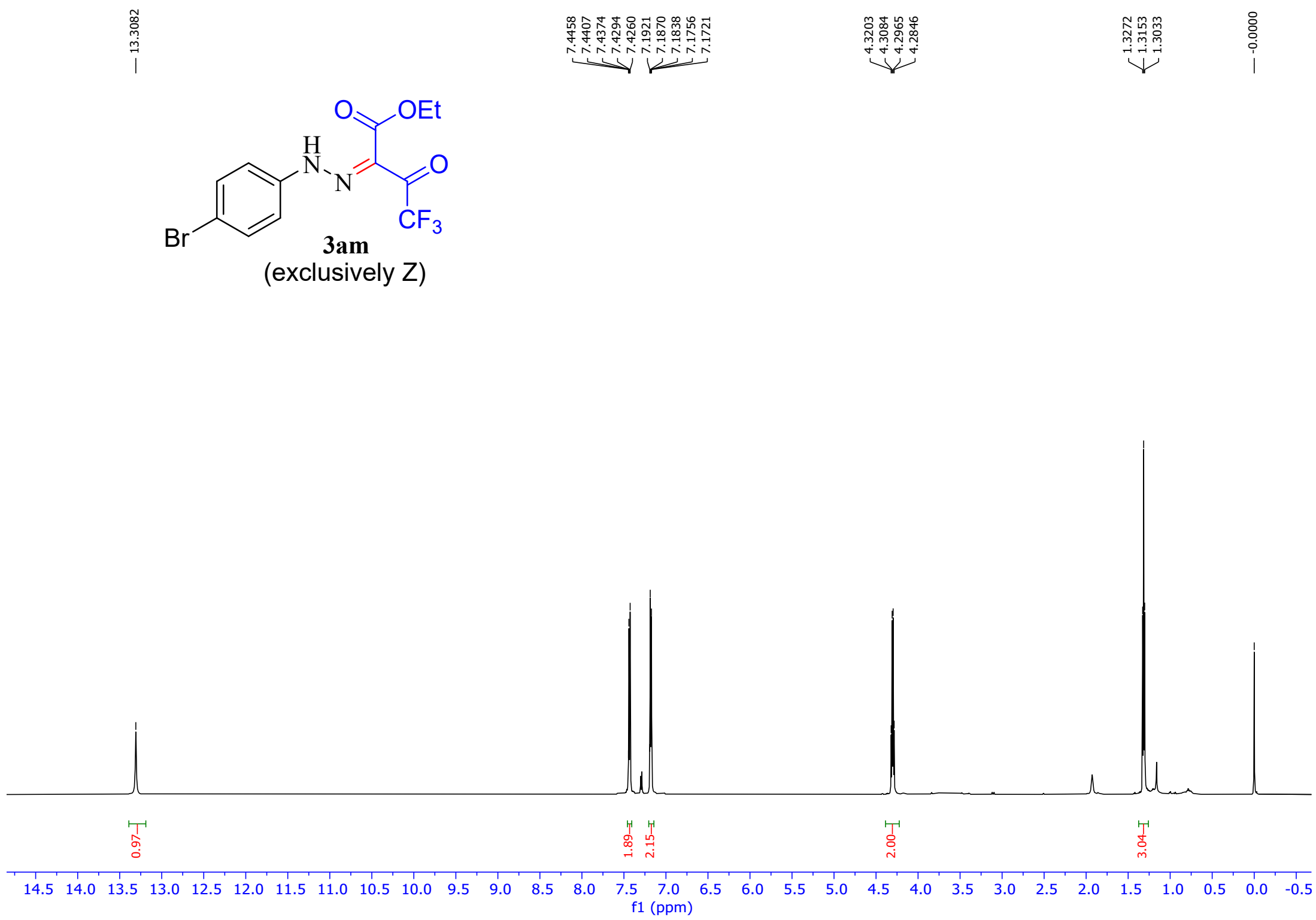
13.3082
7.4458
7.4407
7.4374
7.4294
7.4260
7.1921
7.1870
7.1838
7.1756
7.1721
4.3203
4.3084
4.2965
4.2846
1.3272
1.3153
1.3033
-0.0000
OEt
O
O
H
N
N
Br
CF3
3am
(exclusively Z)
0.97
1.89
2.15
2.00
3.04
f1 (ppm)

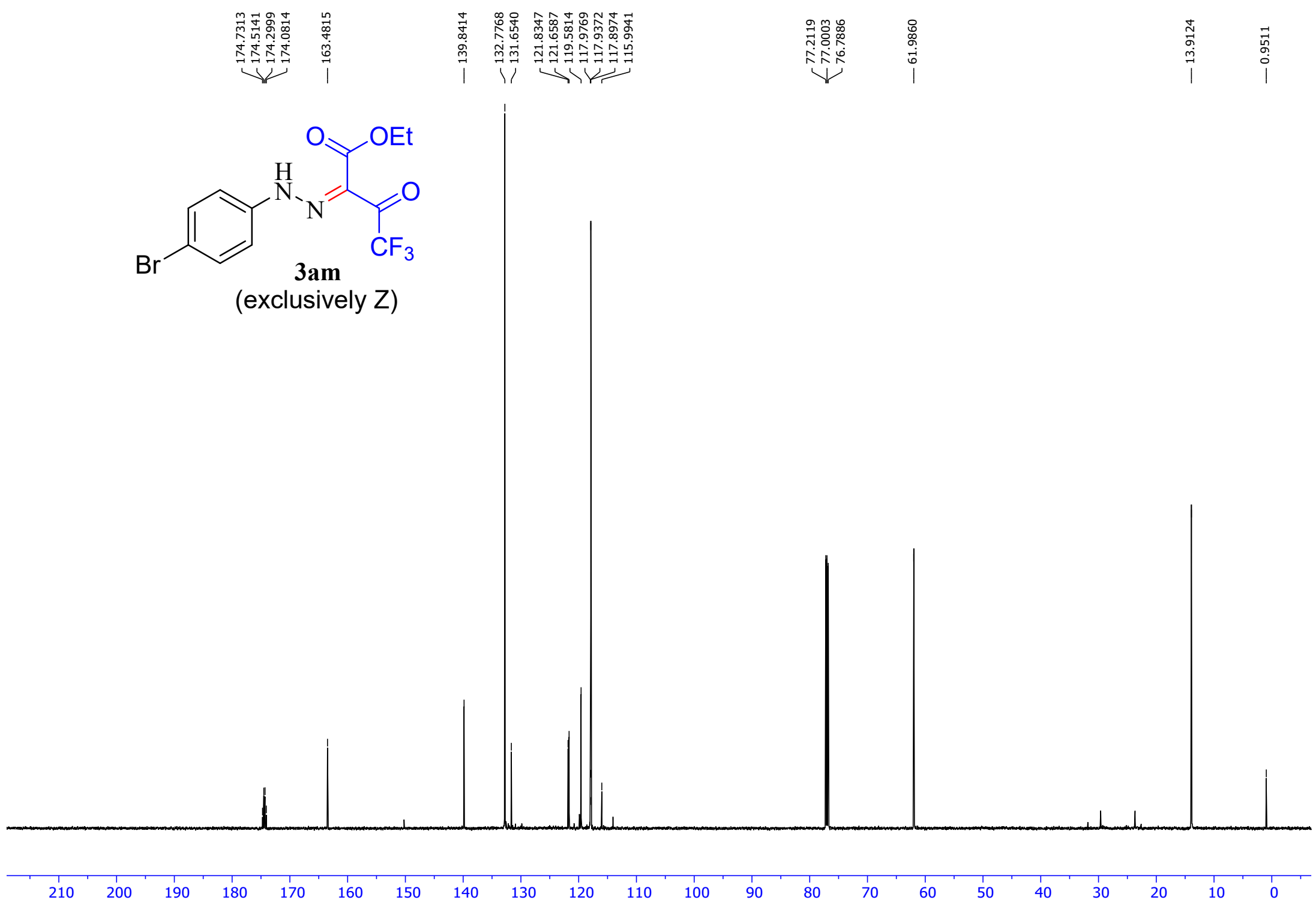
174.7313
174.5141
174.2999
174.0814
163.4815
139.8414
132.7768
131.6540
121.8347
121.6587
119.5814
117.9769
117.9372
117.8974
115.9941
77.2119
77.0003
76.7886
61.9860
13.9124
0.9511
OEt
O
O
H
N
N
Br
CF3
3am
(exclusively Z)
f1 (ppm)

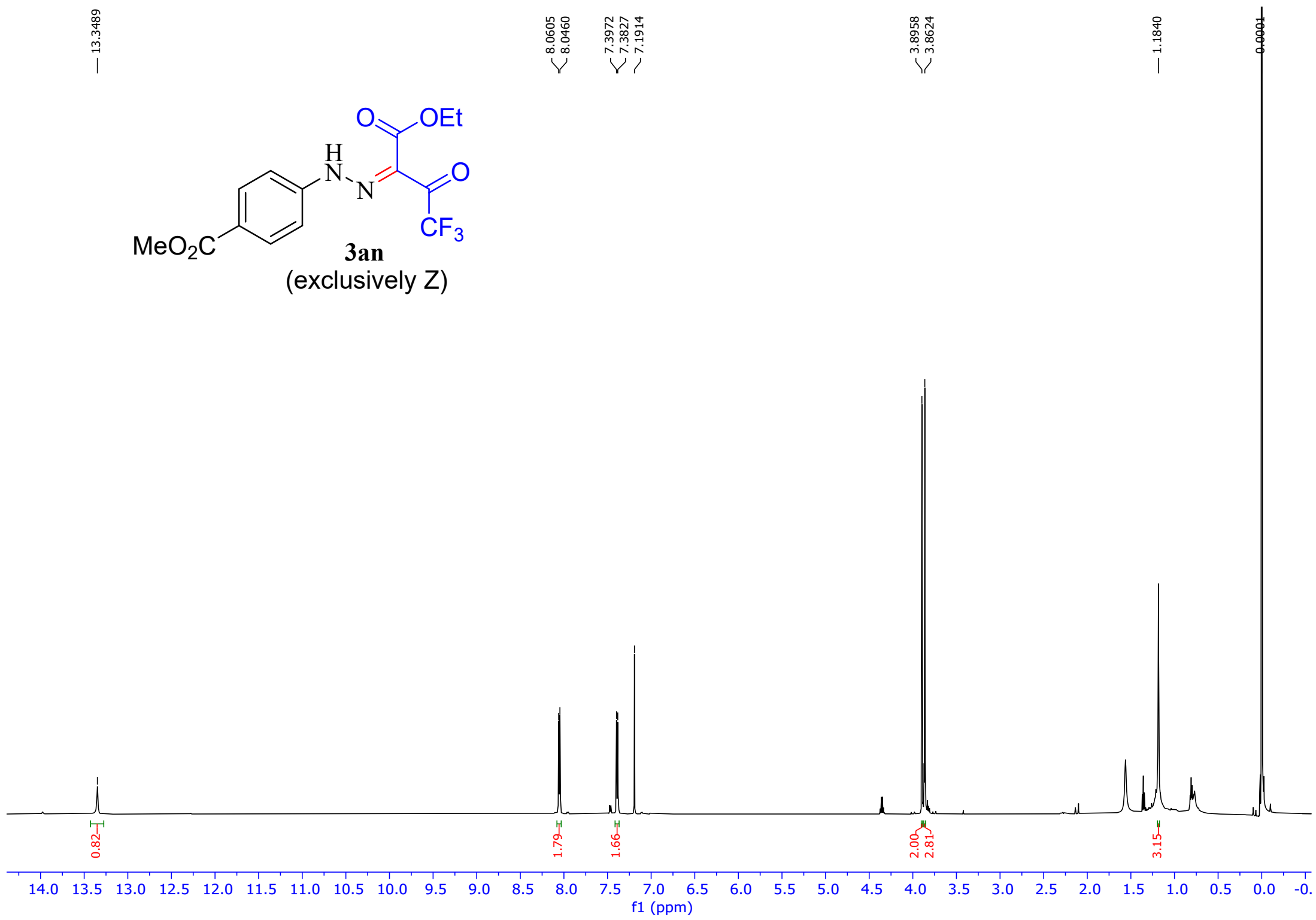
13.3489
8.0605
8.0460
7.3972
7.3827
7.1914
3.8958
3.8624
1.1840
0.0001
OEt
H
N
N
O
O
CF3
MeO2C
3an
(exclusively Z)
0.82
1.79
1.66
2.00
2.81
3.15
f1 (ppm)

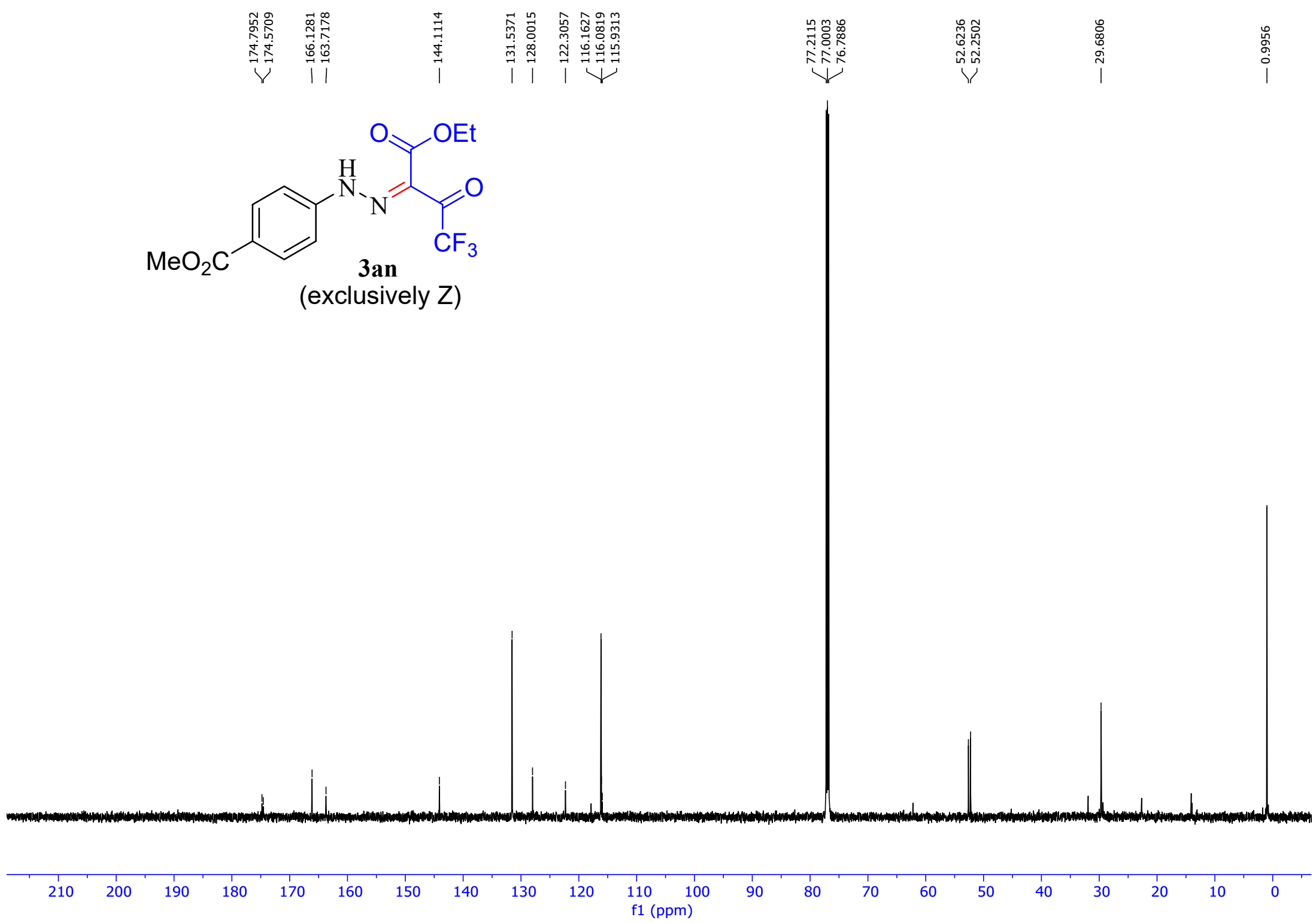
174.7952
174.5709
166.1281
163.7178
144.1114
131.5371
128.0015
122.3057
116.1627
116.0819
115.9313
77.2115
77.0003
76.7886
52.6236
52.2502
29.6806
0.9956
OEt
H
N
N
O
O
CF3
MeO2C
3an
(exclusively Z)
f1 (ppm)

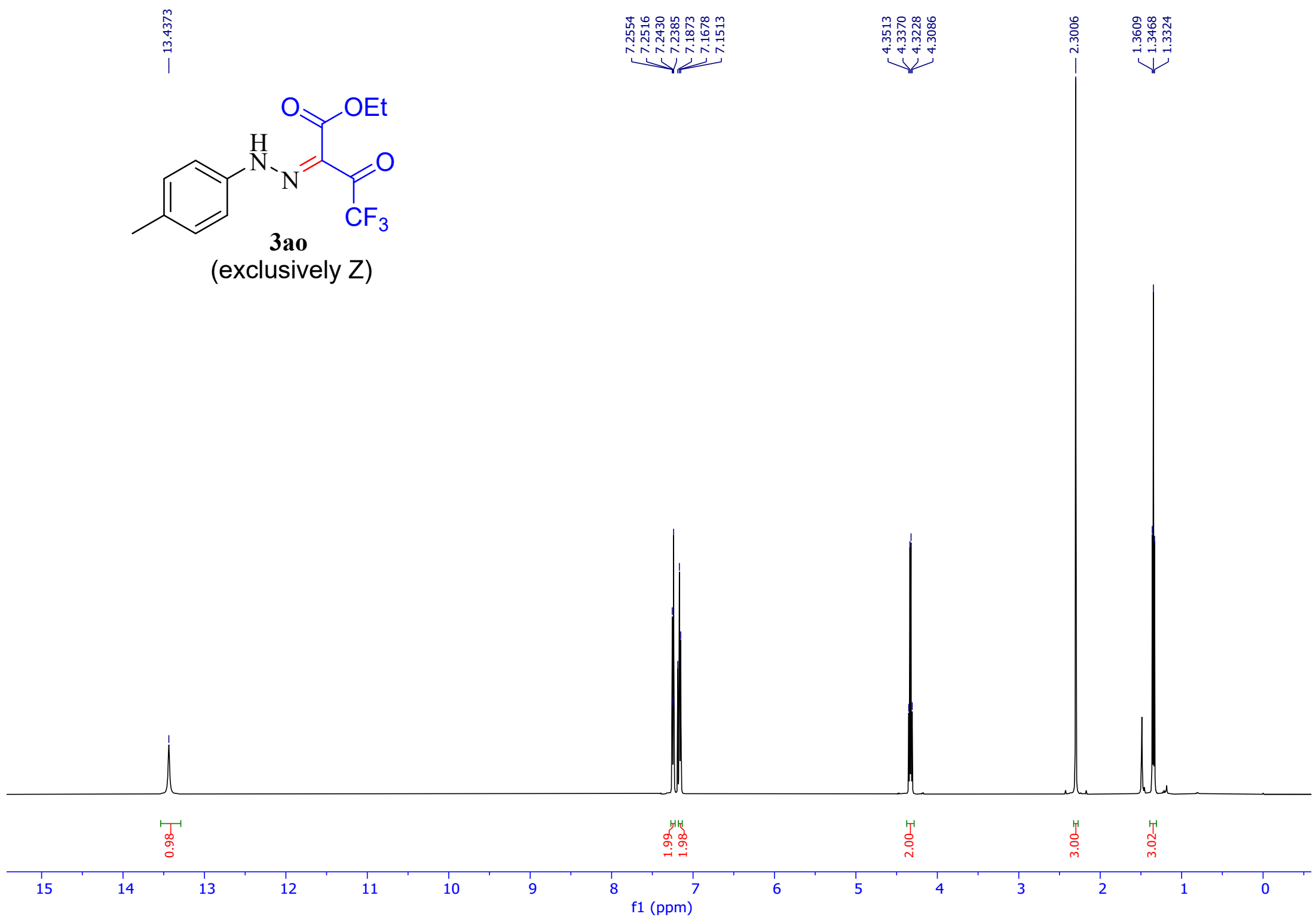
13.4373
7.2554
7.2516
7.2430
7.2385
7.1873
7.1678
7.1513
4.3513
4.3370
4.3228
4.3086
2.3006
1.3609
1.3468
1.3324
OEt
H
N
N
O
O
CF_3
3ao
(exclusively Z)
0.98
1.99
1.98
2.00
3.00
3.02
15 14 13 12 11 10 9 8 7 6 5 4 3 2 1 0
f1 (ppm)

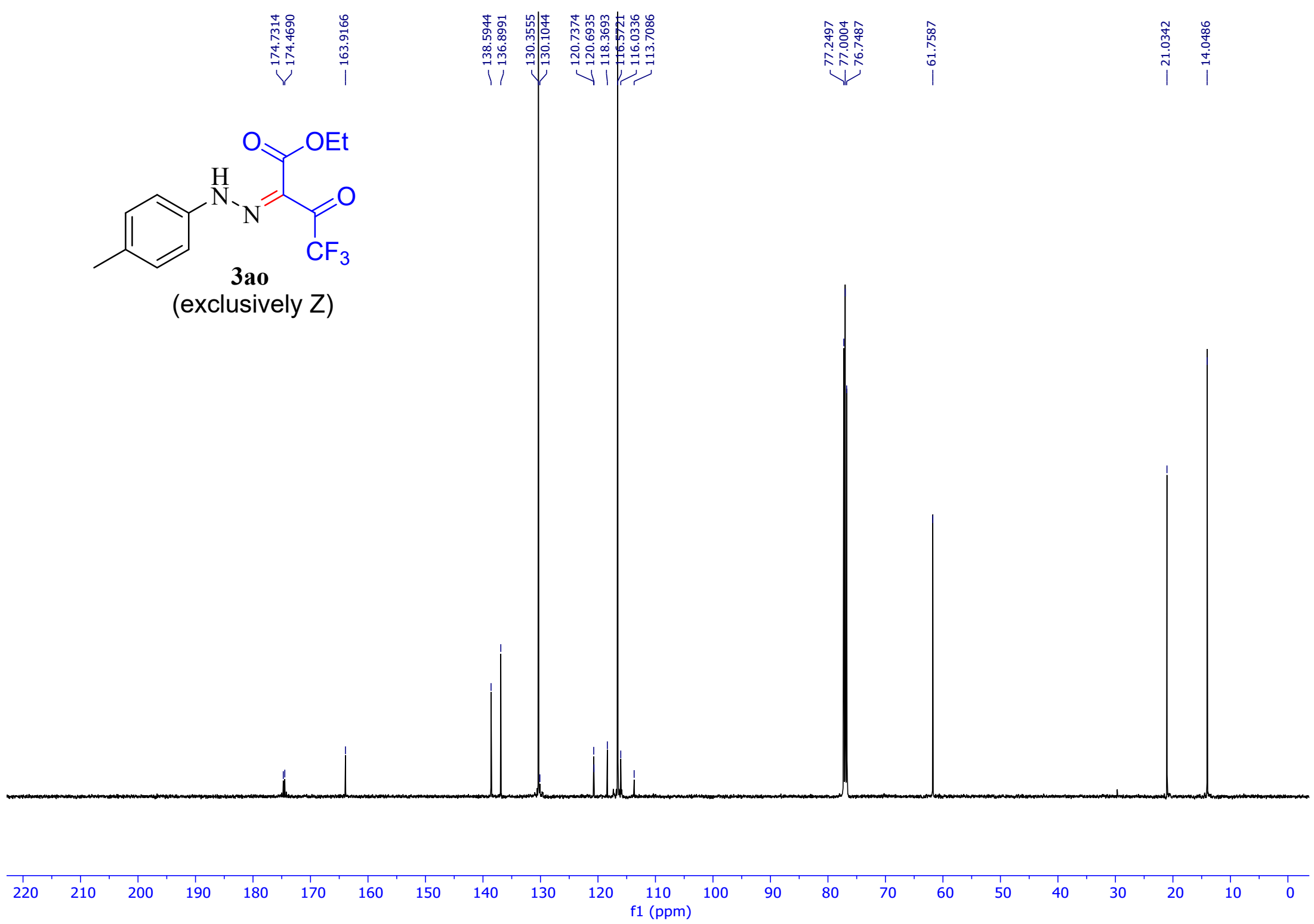
174.7314
174.4690
163.9166
138.5944
136.8991
130.3555
130.1044
120.7374
120.6935
118.3693
116.5721
116.0336
113.7086
77.2497
77.0004
76.7487
61.7587
21.0342
14.0486
OEt
H
N
N
O
O
CF_3
3ao
(exclusively Z)
220 210 200 190 180 170 160 150 140 130 120 110 100 90 80 70 60 50 40 30 20 10 0
f1 (ppm)

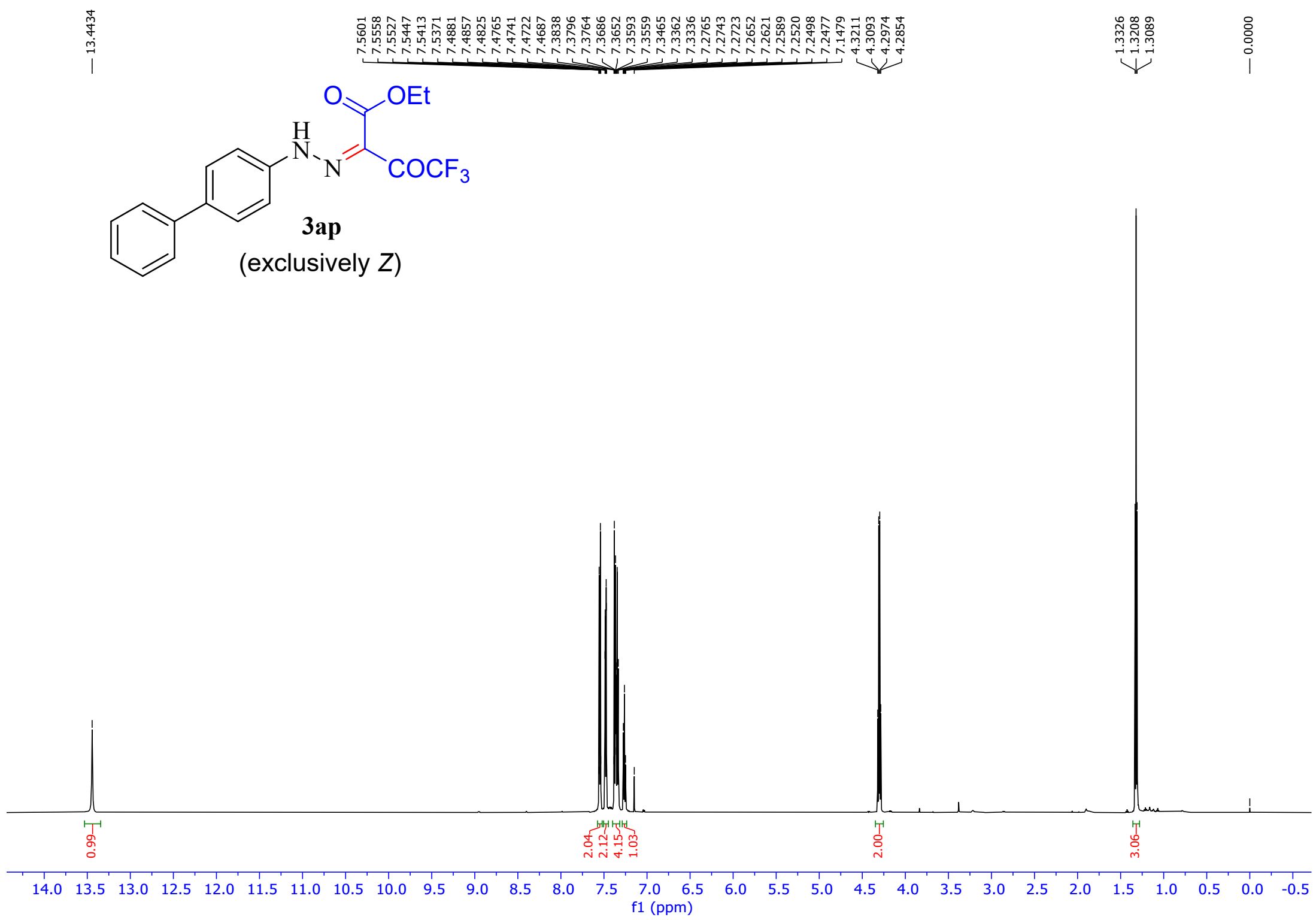
13.4434
7.5601 7.5558 7.5527 7.5447 7.5413 7.5371 7.4881 7.4857 7.4825 7.4765 7.4741 7.4722 7.4687 7.3838 7.3796 7.3764 7.3686 7.3652 7.3593 7.3559 7.3465 7.3362 7.3336 7.2765 7.2743 7.2723 7.2652 7.2621 7.2589 7.2520 7.2498 7.2477 7.1479
4.3211 4.3093 4.2974 4.2854
1.3326 1.3208 1.3089
0.0000
O
OEt
H
N
N
COCF3
3ap
(exclusively Z)
0.99
2.04
2.12
4.15
1.03
2.00
3.06
f1 (ppm)

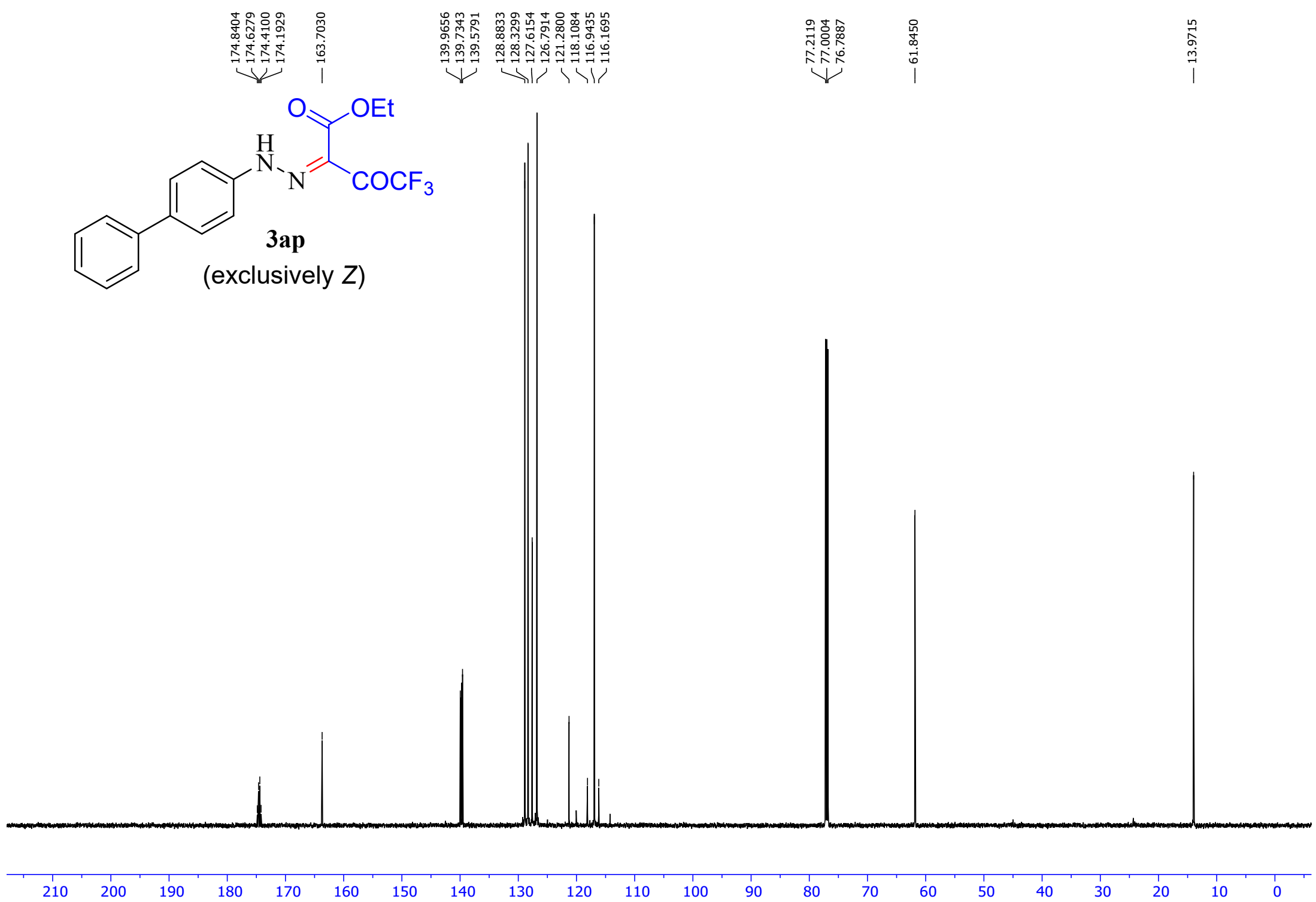
174.8404 174.6279 174.4100 174.1929
163.7030
139.9656 139.7343 139.5791
128.8833 128.3299 127.6154 126.7914 121.2800 118.1084 116.9435 116.1695
77.2119 77.0004 76.7887
61.8450
13.9715
O
OEt
H
N
N
COCF3
3ap
(exclusively Z)
f1 (ppm)

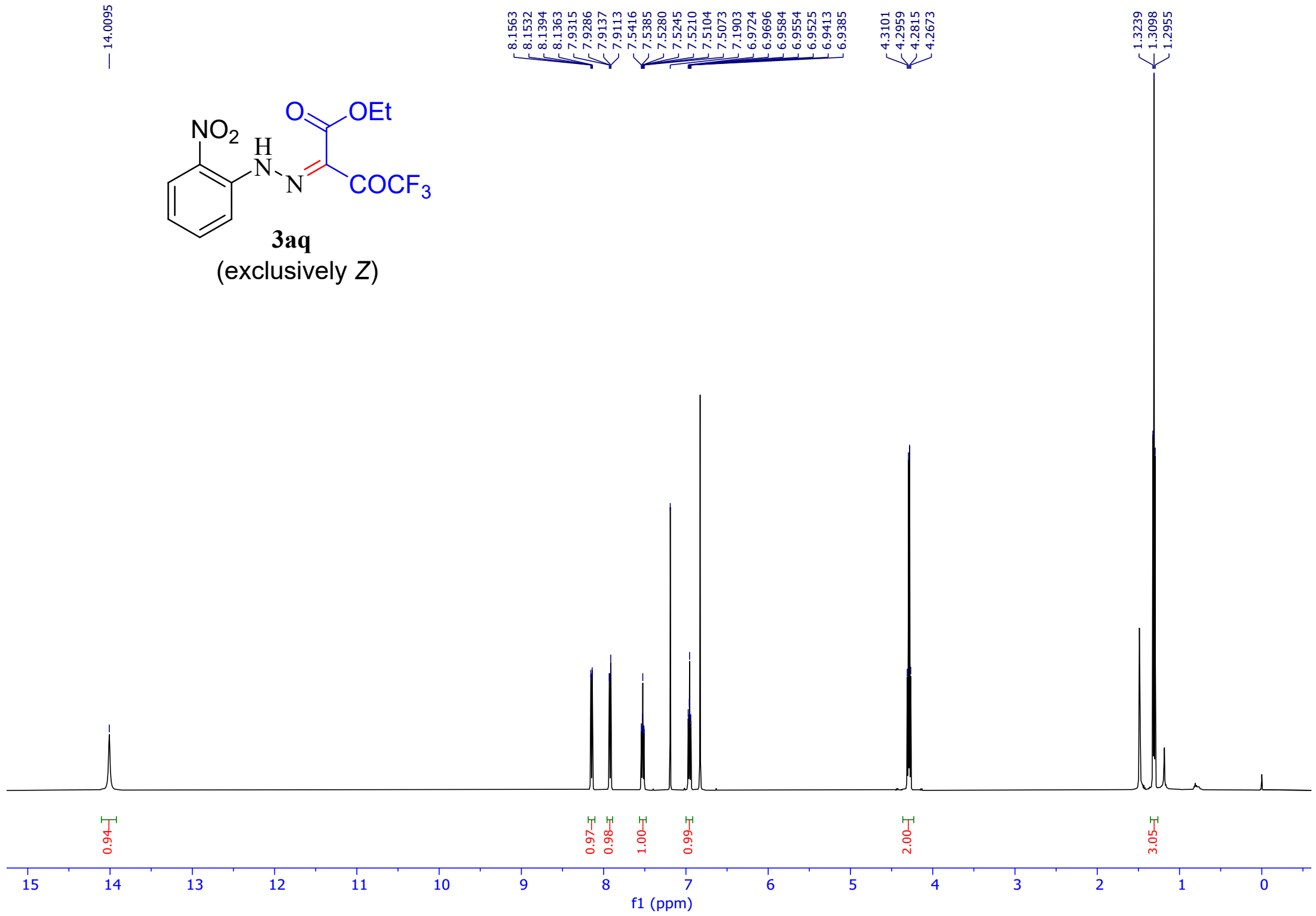
3aq
(exclusively Z)
f1 (ppm)

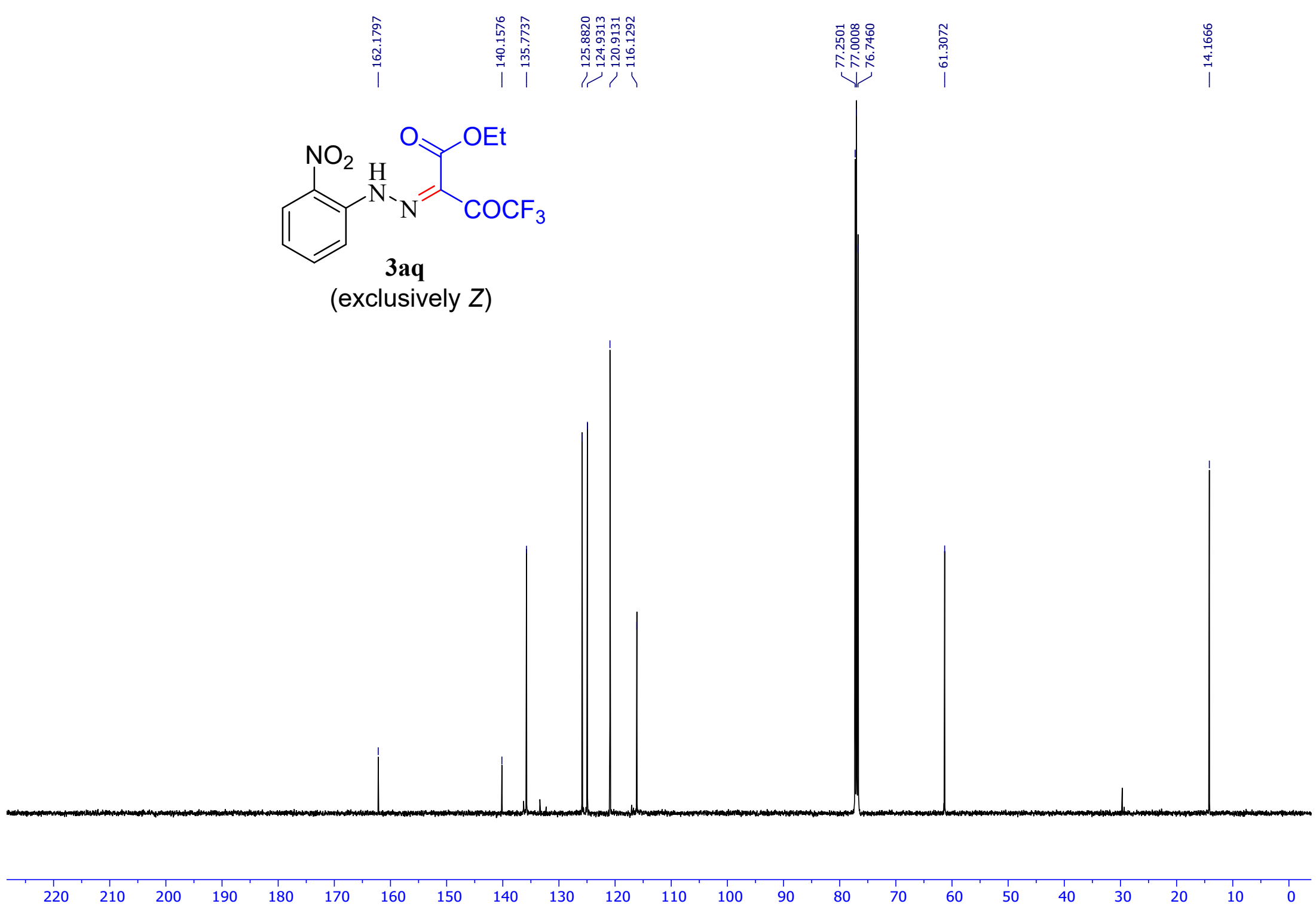
3aq
(exclusively Z)
f1 (ppm)

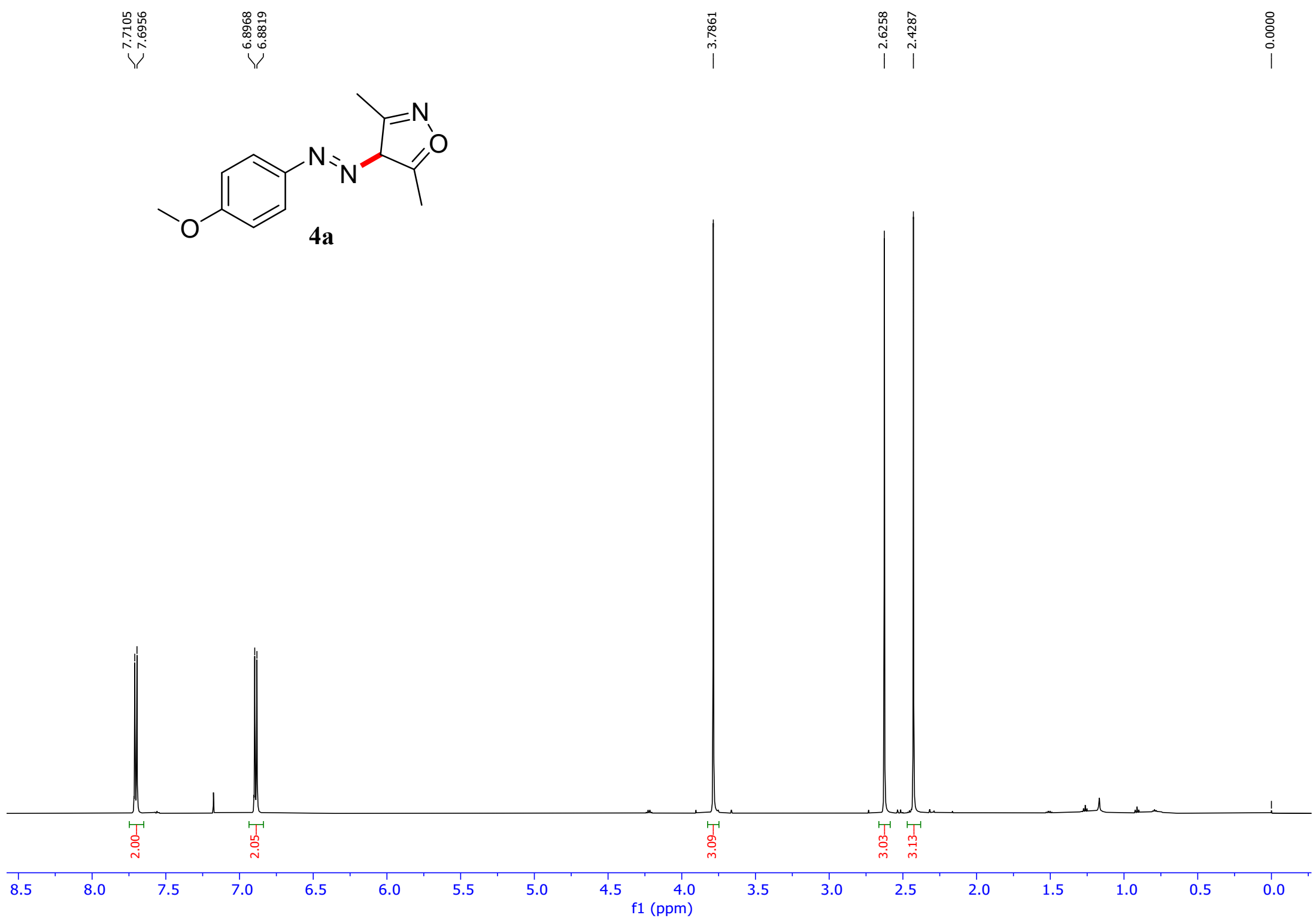
7.7105
7.6956
6.8968
6.8819
3.7861
2.6258
2.4287
0.0000
N
O
N
N
O
2.00
2.05
3.09
3.03
3.13
f1 (ppm)

4a

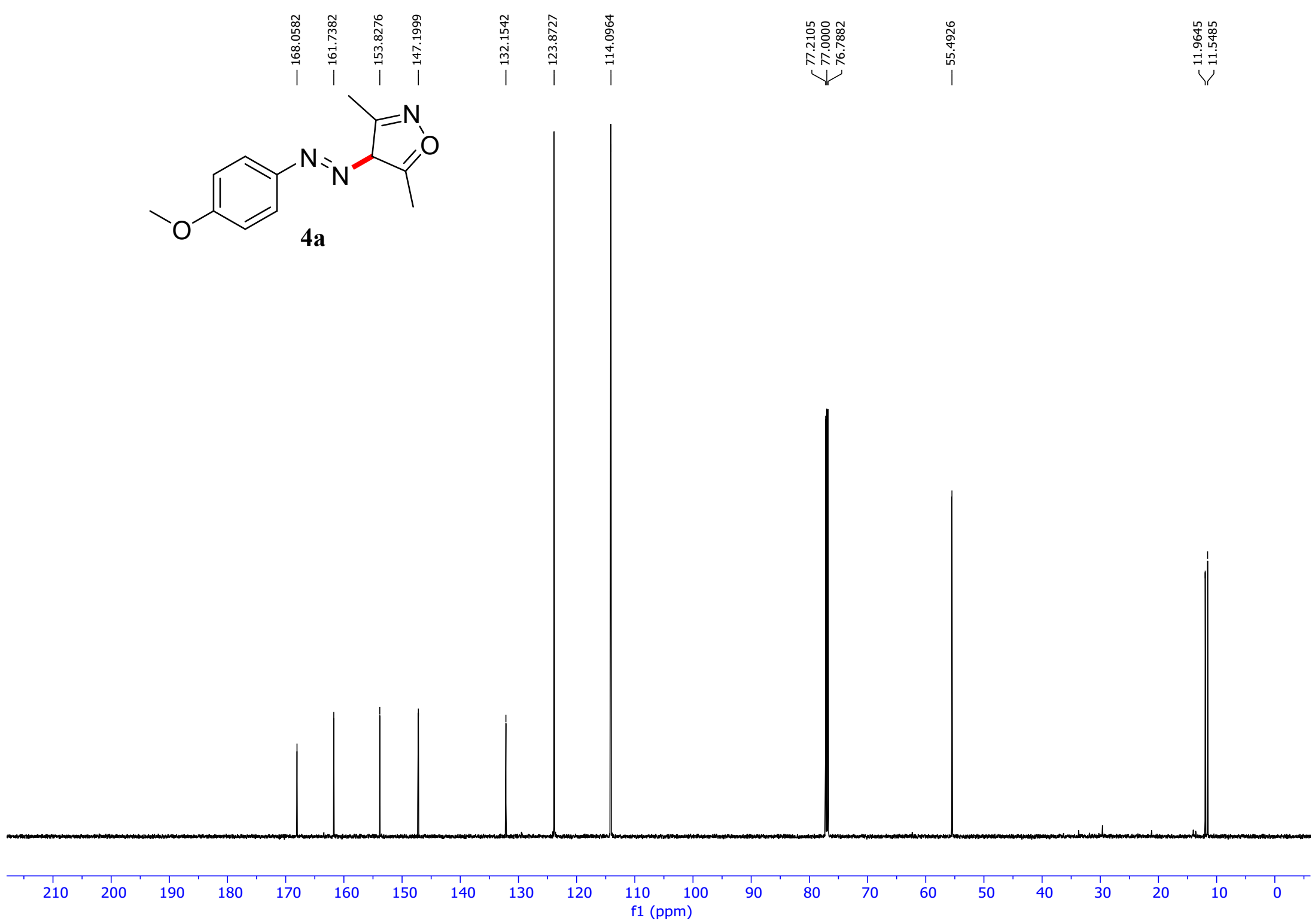
168.0582
161.7382
153.8276
147.1999
132.1542
123.8727
114.0964
77.2105
77.0000
76.7882
55.4926
11.9645
11.5485
N
O
N
N
O
f1 (ppm)

4a

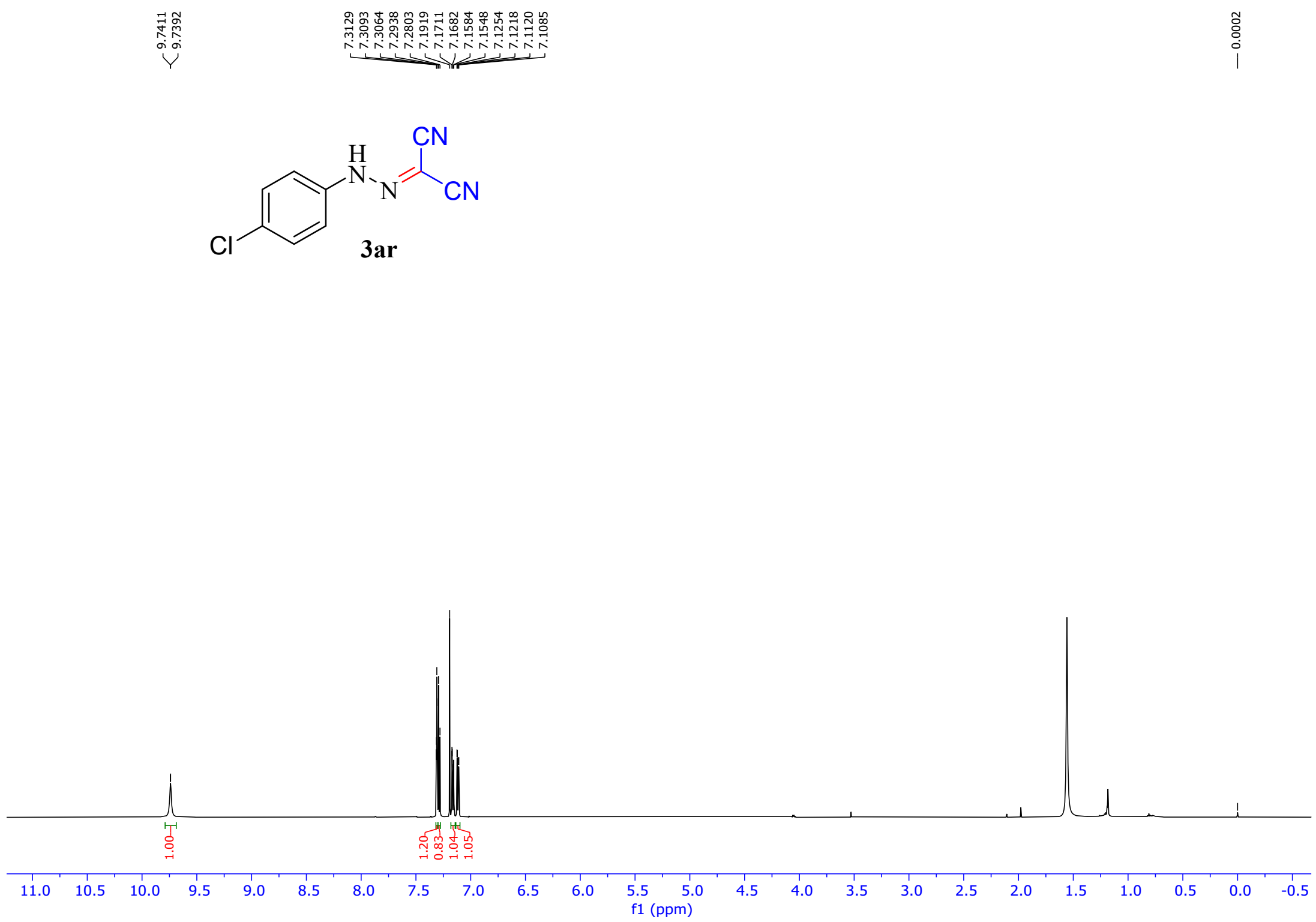
9.7411
9.7392
7.3129
7.3093
7.3064
7.2938
7.2803
7.1919
7.1711
7.1682
7.1584
7.1548
7.1254
7.1218
7.1120
7.1085
0.0002
H
N
N
CN
CN
Cl
3ar
1.00
1.20
0.83
1.04
1.05
f1 (ppm)

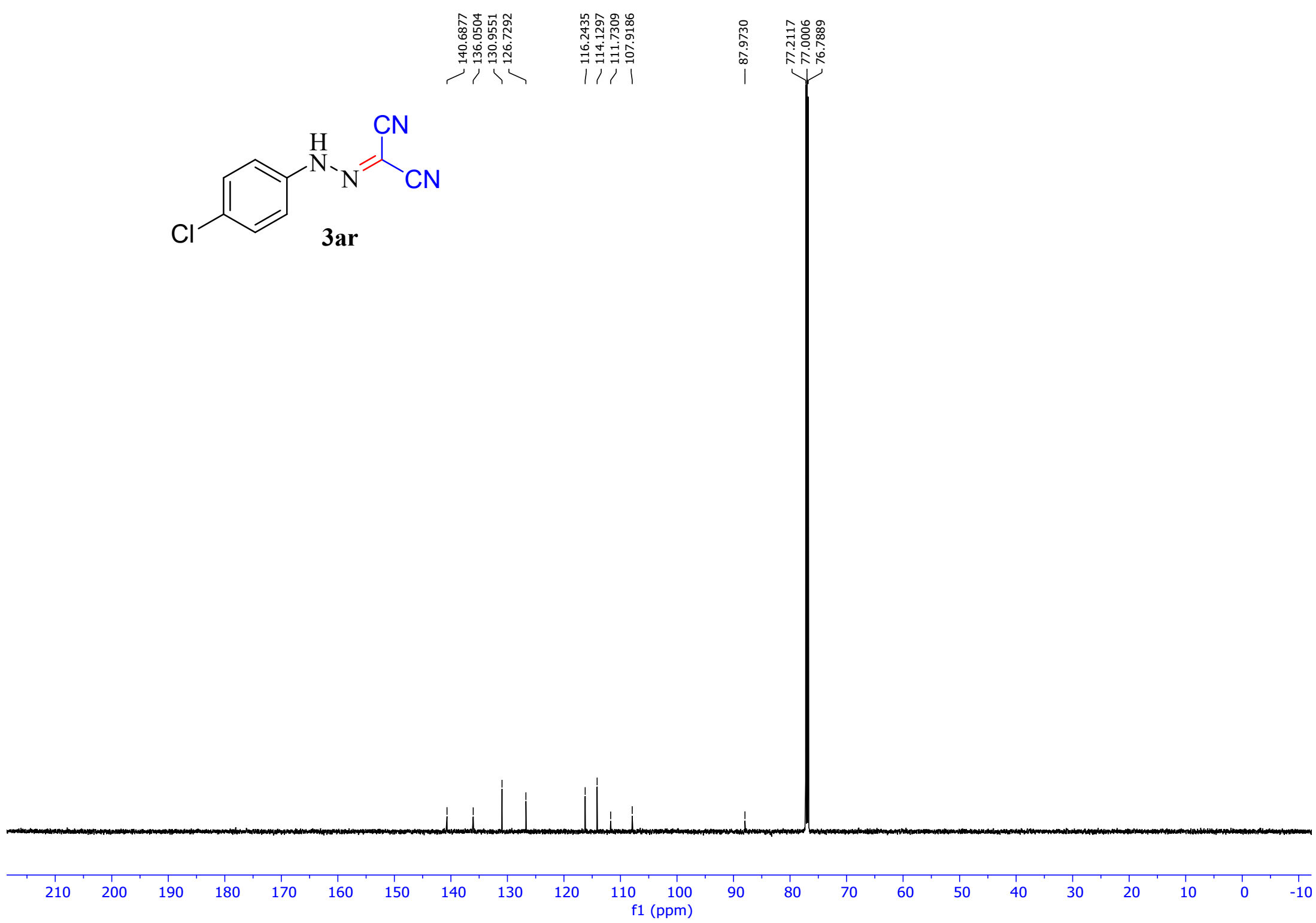
140.6877
136.0504
130.9551
126.7292
116.2435
114.1297
111.7309
107.9186
87.9730
77.2117
77.0006
76.7889
H
N
N
CN
CN
Cl
3ar
f1 (ppm)
